# Clinical Implications of Sub-grouping HER2 Positive Tumors by Amplicon Structure and Co-amplified Genes

**DOI:** 10.1038/s41598-019-55455-6

**Published:** 2019-12-11

**Authors:** Myriam Maoz, Michal Devir, Michal Inbar, Ziva Inbar-Daniel, Dana Sherill-Rofe, Idit Bloch, Karen Meir, David Edelman, Salah Azzam, Hovav Nechushtan, Ofra Maimon, Beatrice Uziely, Luna Kadouri, Amir Sonnenblick, Amir Eden, Tamar Peretz, Aviad Zick

**Affiliations:** 1 Sharett Institute of Oncology, Hebrew University-Hadassah Medical Center, Jerusalem, Israel; 2 Department of Pathology, Hebrew University-Hadassah Medical Center, Jerusalem, Israel; 3 The Oncology Division, Tel Aviv Sourasky Medical Center, Tel Aviv, Israel; 4 Department of Cell & Developmental Biology, Institute of Life Sciences, The Hebrew University of Jerusalem, Edmond J. Safra Campus, Givat Ram, Jerusalem, Israel

**Keywords:** Cancer genomics, Oncogenes

## Abstract

*ERBB2* amplification is a prognostic marker for aggressive tumors and a predictive marker for prolonged survival following treatment with HER2 inhibitors. We attempt to sub-group HER2+ tumors based on amplicon structures and co-amplified genes. We examined five HER2+ cell lines, three HER2+ xenographs and 57 HER2+ tumor tissues. *ERBB2* amplification was analyzed using digital droplet PCR and low coverage whole genome sequencing. In some HER2+ tumors *PPM1D*, that encodes WIP1, is co-amplified. Cell lines were treated with HER2 and WIP1 inhibitors. We find that inverted duplication is the amplicon structure in the majority of HER2+ tumors. In patients suffering from an early stage disease the *ERBB2* amplicon is composed of a single segment while in patients suffering from advanced cancer the amplicon is composed of several different segments. We find robust WIP1 inhibition in some HER2+ *PPM1D* amplified cell lines. Sub-grouping HER2+ tumors using low coverage whole genome sequencing identifies inverted duplications as the main amplicon structure and based on the number of segments, differentiates between local and advanced tumors. In addition, we found that we could determine if a tumor is a recurrent tumor or second primary tumor and identify co-amplified oncogenes that may serve as targets for therapy.

## Introduction

*ERRB2* encodes HER2, a member of the epidermal growth factor receptors (EGFR). HER2 dimerization, with other receptors of the EGFR family, initiates a signaling cascade leading to cell proliferation^1^. *ERBB2* amplification, defined as multiple copies of a DNA segment containing the *ERBB2* gene, is found in tumors^2^ and *ERBB2* amplified/ HER2 positive (HER2+) cancers are treated as a unique clinical entity due to course of disease and to treatment options. *ERBB2* amplification is a prognostic marker for aggressive breast tumors^3^ and a predictive marker for prolonged survival of breast^4^, gastric^5^ and colon^6^ cancer patients treated with HER2 inhibitors.

Identification of *ERBB2* amplification is performed using fluorescence *in situ* hybridization (FISH)^7^, and immunohistochemistry (IHC) for HER2 overexpression^8^. These methods are the gold standard and are routinely used in clinical care. Further characterization of DNA amplification can be performed using digital droplet PCR (ddPCR) and low coverage whole genome sequencing (lcWGS). DdPCR is a robust and precise method for enumerating the copy number (CN) of a specific DNA segment^9^. LcWGS identifies DNA amplifications and deletions throughout the genome as well as amplicon structure (AS)^10^ but also suffers from bias in CN enumeration due to variable efficacy in library preparation and DNA sequencing in different parts of the genome^11^, combining these methods can detail an amplicon CN and AS. Identifying the AS and other genes that are amplified simultaneously as separate events in parallel to *ERBB2*, co-amplified genes, may shed light on pathological mechanisms driving *ERBB2* amplification and provide clinical insight as well as additional treatment options.

Three principal amplicon structures were described in tumor amplified DNA: inverted duplication (ID), tandem repeat (TR) and double minute (DM)^12^. In ID one DNA segment is connected to the same segment in an inverted orientation, telomeric end to telomeric end and centromeric end to centromeric end. In TR, a DNA segment is connected to the same segment as a tandem repeat, the telomeric end of one segment is linked to the centromeric end of a second segment. A DM is composed of several DNA segments from different parts of the genome that are oriented randomly. A DM can be found either as an extra-chromosomal DNA fragment or as part of a chromosome^13^. An *ERBB2* amplicon with an ID was described in the breast cancer cell line HCC1954 model^12^ as well as in breast cancer patients^14,15^. In other tumors, a TR of *ERBB2* segment linked by an inversion to 17q21.3 was associated with a *BRCA1* loss, leading to a DM structure^16^. In HER2+ breast cancer patients co-amplification of *EGFR*^17^, *FGFR1*^18^ and *MYC*^19^ is a poor prognostic marker.

In this work, we attempt to further investigate the *ERBB2* amplicon in HER2+ tumors, based on AS and co-amplified genes using ddPCR and lcWGS. We describe the *ERBB2* AS of 40 HER2+ tumors and the clinical course of the disease. We find that in the majority of HER2+ tumors the AS is a single segment ID. In addition, in early stage cancer the *ERBB2* amplicon is composed of a single segment, while in advanced stage cancer it is composed of several different segments. We also found that co-amplification of *PPM1D*, which encodes wildtype p53-induced phosphatase 1 (WIP1), could identify a sub-group of HER2+ cell lines that are sensitive to treatment with a WIP1 inhibitor.

## Results

### Patient characteristics

Table 1 reports the characteristics of 53 patients suffering from 57 HER2+ tumors, in 47 tumors HER2 status was identified using IHC, in six tumors using FISH, in one using FoundationOne and in three tumors HER2 status was not known. In these three tumors the patients are carriers of a germline *TP53* mutation. DNA was extracted from the primary tumor (n = 46), local recurrences or distant metastasis (n = 11). Tumors were either naive to chemotherapy (n = 45), or previously treated (n = 12).

**Table 1:** HER2 positive cancer patient characteristics.

Median Age at Diagnosis (range)	49 (22–83)
**Patient gender**	N (%)
Female	51 (96%)
Male	2 (4%)
**Patient ethnicity**	N (%)
Arab	4 (8)
Ashkenazi Jew	22 (42)
Sephardic Jew	17 (32)
Other	10 (19)
**Primary tumor Site**	N (%)
Breast	53 (93)
Gastric	3 (5)
Colon	1 (2)
**Tumor Stage**	N (%)
I	13 (23)
II	23 (40)
III	7 (12)
IIIC-IV	11 (19)
Recurrent	3 (5)
**Tumor Pathology**	N (%)
Adenocarcinoma	4 (7)
Atypical medullary carcinoma	1 (2)
Invasive duct carcinoma	49 (86)
Invasive lobular carcinoma	2 (4)
Invasive micropapillary carcinoma	1 (2)
**Tumor ER statues**	N (%)
+	33 (63)
−	19 (37)
N/A	5
**Tumor HER2 identification**	
FISH	6 (10)
IHC	47 (90)
*TP53* carrier	3
FoundationOne	1

### ID is the AS in the majority of *ERBB2* amplicons

**Figure 1:**
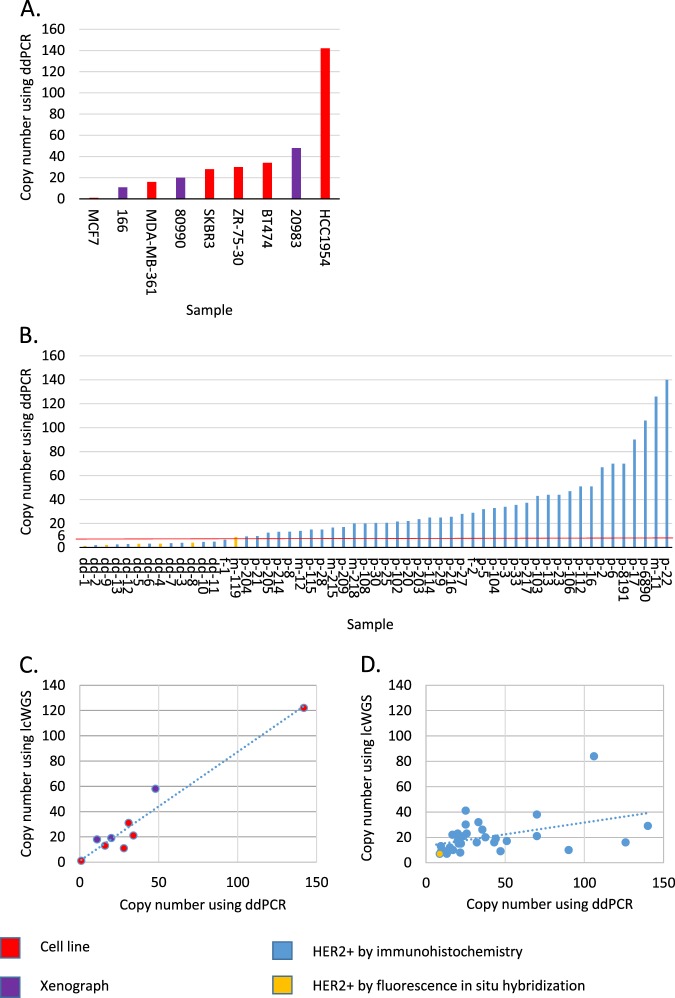
*ERBB2* copy number in study samples. We measured *ERBB2* CN using ddPCR and lcWGS in six samples derived from cell lines, colored red; three xenographs, colored purple (panel A); 55 HER2+ tumors, colored blue and six FISH positive tumors, colored orange. 42 tumors were found ddPCR positive, using a cut-off of six copies, and were further examined using lcWGS (panel B). In samples derived from cell lines and xenographs the correlation between ddPCR and lcWGS is strong, with a linear regression represented by the dotted line with a R2 of 0.94 (panel C). In 42 samples derived from FFPE two ddPCR positive samples are negative when measured using lcWGS, with a CN of two. In the 40 remaining samples the correlation between tests is weaker, with a linear regression represented by the dotted line with a R^2^ of 0.19 (panel D).

We performed ddPCR on a HER2- cell line (MCF7), HER2+ cell lines (BT474^20^, HCC1954^12^, MDA-MB-361^7^, SKBR3^21^, ZR-75-30^22^) and in three HER2+ xenographs (166; 20983; 80990). We found that in the HER2- cell line *ERBB2* gene is not amplified and in the HER2+ cell lines and xenographs *ERBB2* is found in more than six copies (Fig. 1A).

DNA was successfully extracted in 55/57 FFPE samples and ddPCR was performed. In 42 positive samples, with more than six copies of the *ERBB2* gene, the average CN is 37 with a median of 27 (range: 7–140), in range of previously described *ERBB2* CN in HER2+ breast cancer using FISH^7^. In 13 samples, we identified less than six *ERBB2* gene copies. In 6/13 samples with less than six *ERBB2* gene copies a ratio of 1.8 *ERBB2* gene to control gene or more was found. The concordance between HER2+ samples defined by immunohistochemistry and FISH to ddPCR is 85%, similar to previous reports testing the utility of *ERBB2* ddPCR in FFPE HER2+ tissue^23^. Samples with less than six copies included 5/6 FISH positive samples perhaps due to a low *ERBB2* CN in the sampled tissue (Fig. 1B).

LcWGS allows both CNV and AS detection. To determine the AS in samples that harbor a high CN of *ERBB2*, we sequenced samples that contain more than six copies of *ERBB2* according to ddPCR. As expected, we found no evidence for *ERBB2* amplification in the HER2- cell line (MCF7) while in the HER2+ cell lines *ERBB2* was found in more than six copies (Supplementary Table 1). Analyzing the *ERBB2* AS we identified an ID composed of a single segment in the HCC1954 cell line and a ID composed of a several segments in the SKBR3 cell line, as previously described^12,24^ validating our method for AS assignment. Our analysis could determine AS for other HER2+ cell lines: ID in BT474, and DM in MDA-MB-361, and ZR-75–30. In three HER2+ xenographs obtained from Champions^©^ (166R; 20983L; 80990L) a DM AS is identified (Supplementary Table 1).

Of 42 HER2+ tumor samples (based on ddPCR), lcWGS detected *ERBB2* amplification in 40 samples (Table 2). The average CN is 19, with a median of 16 (range: 6–84) and median amplicon size of 1.4 Mb (range: 0.2–14.1). The minimally amplified region in these tumors spans 105,000 bp and includes the *TCAP*, *PMNT*,*PGAP3*, *ERBB2*, *MIEN1* and *GRB7* genes, as previously described^15^. When comparing ddPCR and lcWGS *ERBB2* CN we found a strong correlation in cell lines and xenographs (Fig. 1C) and a weaker correlation in FFPE tissue (Fig. 1D).

**Table 2:** HER2+ tumor samples characteristics.

Sample^#^	Primary site	Stage	ER	*ERRB2* Amplicon CN: WGS/ddPCR	*ERBB2* Amplicon structure	Segment^#^
m-218	Colon	IV	N/A	17/20	DM	3
m-215	Breast	IV	−	22/17	DM	6
p-103* #1	Breast	LR	+	16/43	DM	2
m-119	Breast	IV	+	7/9	DM	10
p-6890	Gastric	IV	N/A	84/106	ID	18
p-104* #2	Gastric	IIIA	N/A	19/33	ID	1
p-8191	Gastric	IIIC	N/A	38/70	ID	2
p-2	Breast	IIA	−	21/67	ID	1
p-6	Breast	IIIC	−	20/70	ID	1
p-20	Breast	IIB	−	15/22	ID	1
p-22	Breast	IIA	−	29/140	ID	1
p-23	Breast	LR	−	29/44	ID	1
p-108* #2	Breast	LR	−	22/20	ID	1
p-114*	Breast	IIA	−	39/25	ID	3
p-216	Breast	IA	−	23/27	ID	1
p-5 #4	Breast	IIB	+	16/32	ID	1
m-12	Breast	IV	+	16/14	ID	4
p-13	Breast	IIA	+	19/44	ID	1
p-27	Breast	IIA	+	29/28	ID	1
p-29* #3	Breast	IV	+	30/25	ID	7
p-30	Breast	IA	+	12/21	ID	1
p-33 #4	Breast	IIB	+	26/36	ID	1
p-112	Breast	IIA	+	17/51	ID	1
p-203	Breast	IIB	+	21/24	ID	1
p-204	Breast	IIIA	+	13/9	ID	1
p-205	Breast	IIIA	+	9/12	ID	1
p-217	Breast	IV	+	20/37	ID	1
p-102* #1	Breast	IA	?	8/22	ID	1
p-16	Breast	IIA	−	15/51	n/a	1
p-21	Breast	IIB	−	9/10	n/a	1
p-25	Breast	IIB	−	15/21	n/a	1
p-28	Breast	IA	−	12/15	n/a	1
p-106	Breast	IIA	−	9/47	n/a	1
p-115	Breast	IIA	−	12/15	n/a	1
p-214	Breast	IIA	−	7/13	other	1
p-3	Breast	IIA	+	13/34	n/a	1
p-8	Breast	IA	+	9/13	n/a	1
m-11*#3	Breast	IV	+	16/126	n/a	1
p-17	Breast	IIA	+	10/90	n/a	1
p-209	Breast	IIB	+	10/17	other	1

*Sample of a germline *TP53* mutation carrier.

^#^Sample from the same patient.

p- sample from primary tumor; m- sample from metastatic tumor.

Legend

Sample – sample name.

Primary site – organ from which the cancer originated.

Stage – Stage of the patient at diagnosis according to the American Joint Committee on Cancer, 7th edition.

ER – Estrogen receptor (ER) expression.

ERRB2 Amplicon CN - WGS/ddPCR – Copy number of ERBB2 as measured by whole genome sequencing (WGS) and digital droplet PCR (ddPCR).

ERBB2 Amplicon structure – Structure of the ERBB2 amplicon, either double minute (DM) or inverted duplication (ID).

Segment # – Number of DNA segments composing the ERBB2 amplicon.

AS was determined in 28 samples: 22 as ID i.e. the majority of SVs identified in all the segments of the amplicon are of the ID type, four as DM i.e. the majority of SVs identified in all the segments of the amplicon are of the DM type, and four samples categorized as “other”. By manual inspection of the “other” samples we could further identify an ID AS in one sample (p-217) and a DM AS in a second sample (m-218). In 23 tumors the ER status is known, 60% are ER+. The rate of AS identification and the percent of samples determined as ID or DM was similar in the ER+ and ER- groups (Supplementary Tables 2, 3 & Supplementary Fig. 1).

In patient samples the *ERBB2* amplicon is composed of a single segment in 19 samples, all with ID AS. In nine samples the *ERBB2* amplicon is composed of several segments (2–18). The additional segments composing the *ERBB2* amplicon are from the chromosome 17 q-arm in all samples, in one sample in addition to segments from the 17 q-arm an additional segment from chromosome 17 p-arm is found (p-6890) and in another sample additional segments are from chromosome 17 q-arm, chromosome 10 and chromosome 20 (p-29). In the nine samples where the *ERBB2* amplicon is composed of several segments five have an ID AS and four a DM AS. In 4/5 samples with an ID AS the centromeric end of the *ERBB2* segment links to another segment and in one sample the telomeric end of the *ERBB2* segment links to another segment.

In 12 samples no AS was identified, out of which seven are ER- and five ER+. In nine samples no SV data was found in the *ERBB2* amplicon area, in two samples the SVs identified are internal translocations spanning tens of nucleotides in the middle of the *ERBB2* amplicon area and in one sample the SVs are between the *ERBB2* amplicon and genomic areas that are not amplified. In samples where no AS is identified the average CN is 11 as opposed to 22 in samples where the AS is identified. No difference in the tumor cells percentage is found in samples where an AS was identified or not. Perhaps other factors in tissue preparation make SV detection more difficult. This is demonstrated when analyzing a primary breast tumor and a bone metastasis from the same patient, samples p-29 and m-11. Both samples shared identical CNV but we identified AS only in the primary breast sample, perhaps due to the difficulty of obtaining good quality DNA in sufficient amount from the bone tissue.

### The *ERBB2* amplicon is composed of a single segment in localized tumors and from multiple segments in advanced tumors

**Figure 2:**
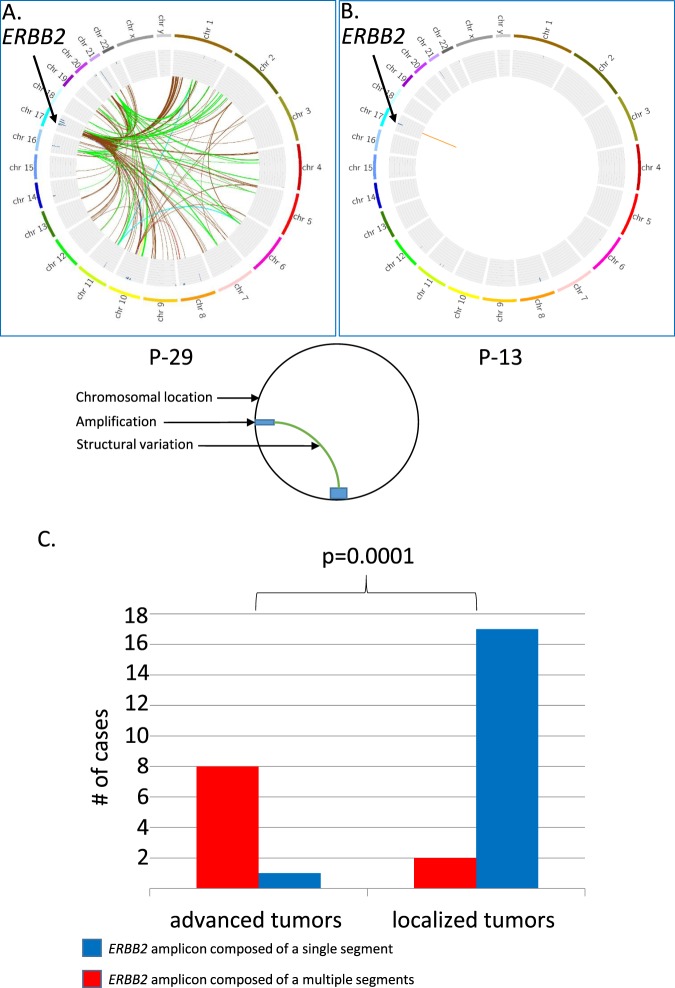
*ERBB2* amplicon structure is different in patient with localized and advanced disease. FAST analysis of low coverage whole genome sequencing (lcWGS) of two primary breast ER+/HER2+ tumors is visualized using Circus. The external ring represents chromosomal location; the inner ring shows areas of amplification as blue bars. Colored lines represent structural variations (SV), all the SV in an amplicon are colored with the same color. Although both (P-29 and P-13) are primary breast ER+/HER2+ tumors, P-29 was resected from a patient suffering from a metastatic disease as a palliative measure and P-13 was resected from a patient with a localized disease with curative intent. In P-29 the *ERBB2* amplicon is composed of several segments (Panel A) and in P-13 of a single segment (Panel B). The *ERBB2* amplicon is composed of a single segment, colored blue, in 17/19 patients with localized tumors, and is composed of multiple segments, colored red, in 8/9 patients with advanced tumors, Fisher exact test, two sided p = 0.0001 (Panel C).

All patients in the study with localized disease, stage I-IIIA, are without evidence of disease during our follow-up and all patients with advanced disease, stage IIIC & IV, responded or had stable disease when treated with anti-HER2 regimens (supplementary Table 2). We chose to focus on the difference in the *ERBB2* amplicon between patients with localized vs. advanced tumors. Of 28 tumors where an *ERBB2* AS is found 19 suffered from a localized disease and nine from advanced disease (Table 2). No difference is found between the mean *ERBB2* CN between localized and advanced tumors, 37 vs. 41 respectively, independent *t-*test is not significant, in line with previous study demonstrating that *ERBB2* CN is not a prognostic marker in HER2+ tumors^25^. On the other hand the *ERBB2* amplicon is composed of a single segment in 17/19 patients with localized tumors, and is composed of multiple segments in 8/9 patients with advanced tumors (Table 2). The deviation from the null hypothesis that a tumor with an *ERBB2* amplicon composed of one segment is found independently in patients suffering from localized or advanced tumors was tested using the Fisher exact test and is rejected with two sided p = 0.0001 (Fig. 2).

### In cases of two primary HER2+ tumors *ERBB2* AS in each primary tumor is distinct

**Figure 3:**
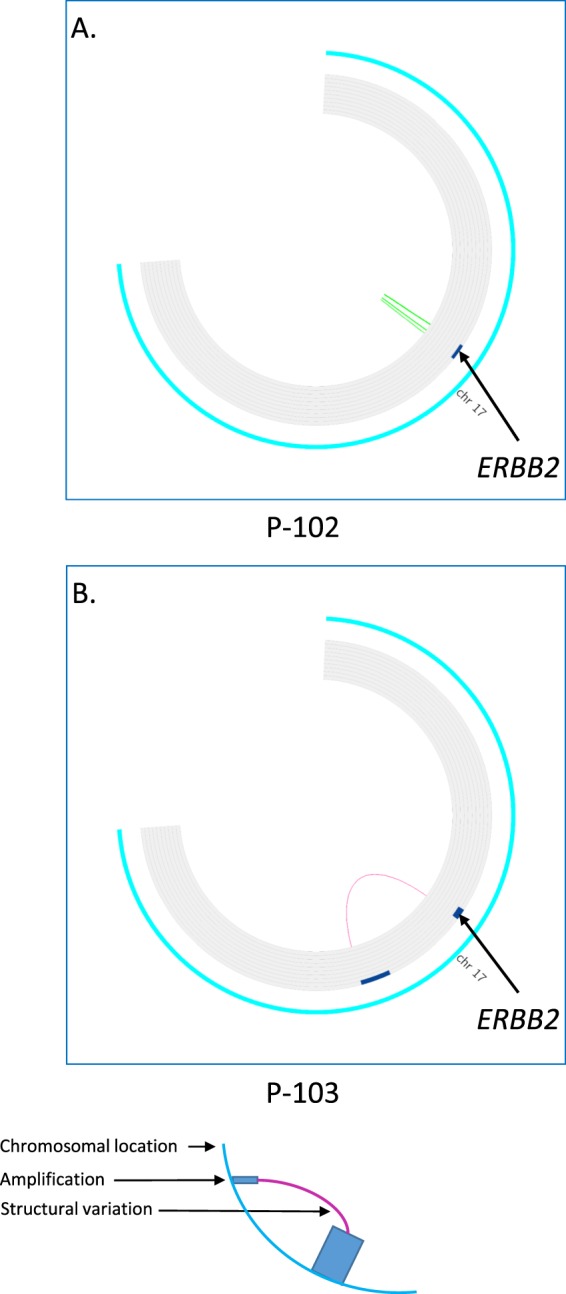
Difference in *ERBB2* amplicon in two breast tumors from the same patient. FAST analysis of low coverage whole genome sequencing (lcWGS) of two primary ER+/HER2+ and ER?/HER2+ breast tumors is visualized using Circus. The external ring represents chromosomal location on chromosome 17, the inner ring shows areas of amplification as blue bars. Colored lines represent structural variations (SV). Both tumors (P-102 and P-103) are primary HER2+ breast cancer. In P-102 the *ERBB2* amplicon spans chr17:37,125,000-38,715,000, has eight copies and has an inverted duplication amplicon structure (panel A) while In P-103 the *ERBB2* amplicon spans chr17:36,960,000-38,070,000, has 16 copies and has a double minute amplicon structure (panel B).

In four patients two samples were available for analysis, three of these patients are carriers of a pathogenic germline *TP53* mutation^26^ that predisposes to multiple tumors^27^. In the first patient, sample p-102 is a primary breast tumor and sample p-103 is a recurrent breast cancer. The samples show a difference in the *ERBB2* amplicon size, CN and structure, suggesting that sample p-103 is a second primary tumor (Fig. 3). In the second patient, sample p-104 is a primary breast tumor and sample p-108 is a primary gastric tumor. The samples show a difference in the *ERBB2* AS, demonstrating that these are independent events. The third patient suffered from primary breast cancer with bone metastasis, sample p-29 is from the patient’s primary breast tumor and sample m-11 is from a bone metastasis. In both samples the *ERBB2* as well as the *RPS6KB1/PPM1D* amplicon size and location are identical. We found an AS in sample p-29 but not in sample m-11, this is most likely due to poorer sample quality of sample m-11. In contrast, in the fourth patient, who suffered from bilateral breast cancer, the samples (p-5 and p-33) show a difference in CNV and the *ERBB2* AS, reaffirming the diagnosis of two independent tumors.

### Some HER2+ cell lines with co-amplified *PPM1D* are inhibited by WIP1 inhibitor

**Figure 4:**
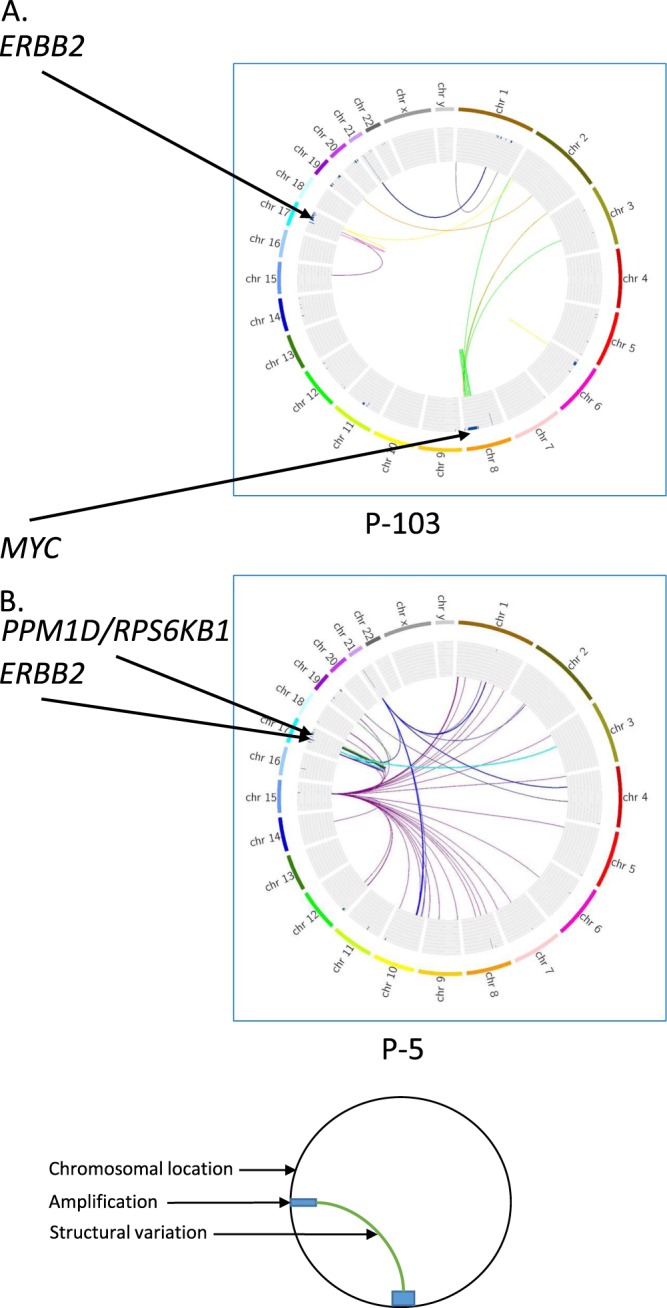
MYC, PPM1D and RPS6KB1 co-amplification with ERBB2. FAST analysis of low coverage whole genome sequencing (lcWGS) of two primary ER+/HER2+ breast tumors is visualized using Circus. The external ring represents chromosomal location; the inner ring shows areas of amplification as blue bars. Colored lines represent structural variations (SV), all the SV in an amplicon are colored with the same color. Although both tumors (P-5 and P-103) are primary breast ER+/HER2+ the co-amplified genes are different. In P-103 *MYC* co-amplification, colored green (panel A) while in P-5 *PPM1D* and *RPS6KB1* co-amplification, colored green (panel B).

We identified, 1–5 additional amplicons with a defined AS that harbor oncogenes, in 14 samples (Table 3), in the additional 26 samples *ERBB2* was the only amplified oncogene identified. These oncogenes are amplified in parallel to *ERBB2* and are found on a separate amplicon, for example *ERBB2* and *MYC* in sample p-103 or *ERBB2* and *PPM1D* in sample p-5 (Fig. 4). Previously described recurring co-amplified oncogenes include *MYC*^19^*, PPM1D*^28^ and *RPS6KB1*^29^, each found in three samples. The *HLF* oncogene is also co-amplified in three samples. *HLF* encodes a transcription factor that is part of the proline and acidic amino acid-rich family and acts as an oncogene in hepatocellular carcinoma^30^.

**Table 3:** Co-amplified genes in HER2 positive cancers.

*ADRM1*													X		2.5%
*APC*														X	2.5%
*CCND1*										X					2.5%
*CDK4*								X							2.5%
*CTTN*										X					2.5%
*EIF3H*					X						X				5%
*FGF19*										X					2.5%
*FGF3*										X					2.5%
*FGF4*										X					2.5%
*HIST1H3B*						X									2.5%
*HLF*								X	X				X		7.5%
*IGF1R*									X						2.5%
*MACC1*												X			2.5%
*MAK*						X									2.5%
*MAP2K3*													X		2.5%
*MAPK7*							X								2.5%
*MSI2*	X						X								5%
*MYC*		X			X									X	7.5%
*NF1*			X												2.5%
*PPM1D*	X			X					X						7.5%
*RPS6KB1*	X			X					X						7.5%
*RSPO2*					X										2.5%
*SPOP*									X						2.5%
*TSHZ2*										X					2.5%
*TWIST*												X			2.5%
*ZNF217*										X					2.5%
	p-5	p-21	p-25	p-29	p-103	p-108	p-114	p-115	p-203	p-204	p-216	p-8191	m-12	m-215	Percentage of amplified samples per gene

**Figure 5:**
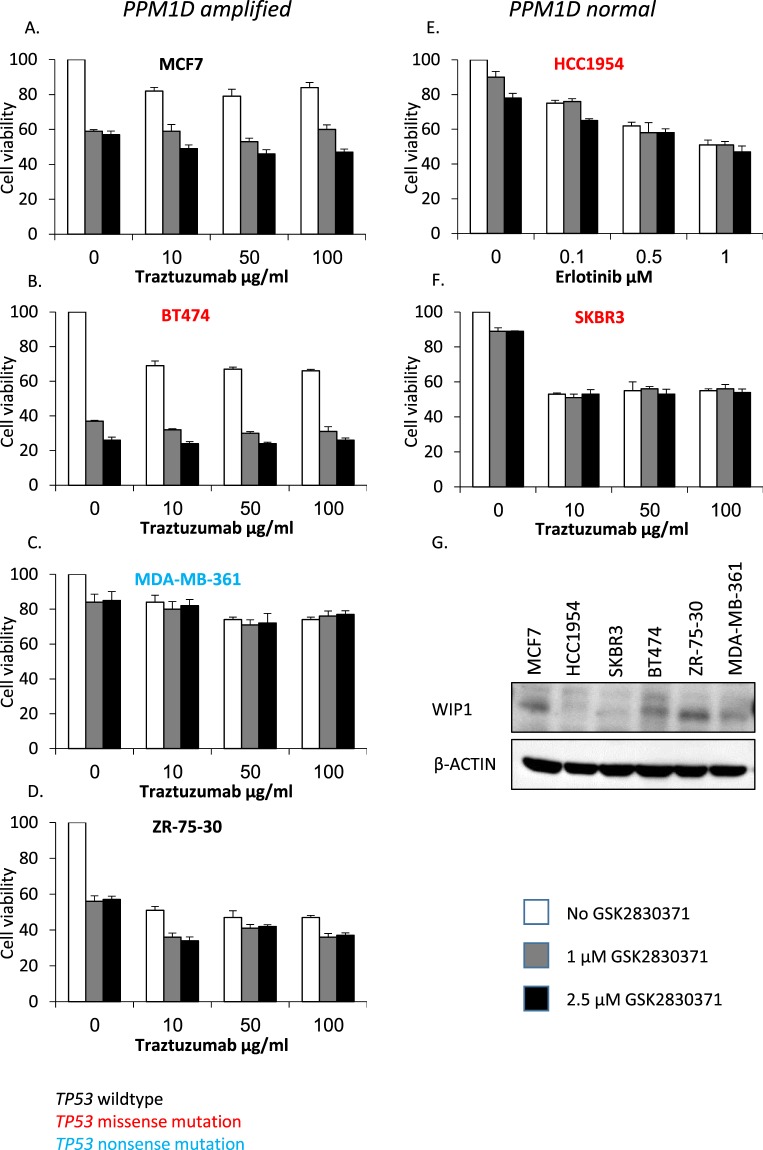
WIP1 and HER2 co-inhibition in different cell lines. We measured cell viability (y-axis) of cell lines with (MCF7, BT474, MDA-MB-361 and ZR-75-30) or without (HCC1954 and SKBR3) *PPM1D* amplification. Some cell lines are *TP53* wildtype (MCF7 and ZR-75-30), colored black; some harbor a *TP53* missense mutation (HCC1954, SKBR3 and BT474), colored red and one harbors a *TP53* nonsense mutation (MDA-MB-361), colored blue. Cell lines were treated with HER2 inhibitors and with GSK2830371, a WIP1 inhibitor. Cell viability decreased by WIP1 inhibition in some of the *PPM1D* amplified cell lines (pane A, B and D) but not in *PPM1D* normal cell lines (pane E and F) and a *PPM1D* amplified, *TP53* nonsense mutated cell line (pane C). We measured WIP1 levels by immunoblotting, elevated WIP1 protein levels were found in cell lines with *PPM1D* amplification compared to cell lines without *PPM1D* amplification (pane G.).

We hypothesized that the *PPM1D* and *ERBB2* co-amplification will have a predictive value for the response to combined treatment with HER2 and WIP1 inhibitors. We tested WIP1 inhibition using cell lines with *PPM1D* amplification and normal *ERBB2* CN (MCF7); with normal *PPM1D* CN and *ERBB2* amplification (HCC1954, SKBR3); and with *ERBB2* and *PPM1D* amplifications (BT474, MDA-MB-361, ZR-75-30). Some cell lines are *TP53* wild type (MCF7 and ZR-75-30) and some are *TP53* mutated (HCC1954, SKBR3, BT474 and MDA-MB-361)^31^ (Supplementary Table 1). We found as previously described that HER2 inhibitors, either trastuzumab and in HCC1954 erlotinib, efficiently inhibited the *ERBB2* amplified cell lines but not the cell line with normal *ERBB2* CN. We found that treating *PPM1D* amplified, *ERBB2* normal, *TP53* wild type (MCF7) cells with the WIP1 inhibitor, GSK2830371 at 1 μM and 2.5 μM, inhibited cell proliferation to 60% as previously described^32^. Treatment of two *PPM1D* amplified, *ERBB2* amplified cell lines one harboring a *TP53* mutation, (BT474) and one with wild type *TP53 (*ZR-75-30) with GSK2830371 at 1 μM and 2.5 μM inhibited cell proliferation to 30% and 60% respectively. The *PPM1D* amplified, *ERBB2* amplified, *TP53* mutated cell line (MDA-MB-361) and cell lines with normal *PPM1D* CN (HCC1954 and SKBR3) were not inhibited by GSK2830371 (Fig. 5A–F). Protein expression of WIP1 is higher in *PPM1D* amplified cell lines (MCF7, BT474, ZR-75-30 and MDA-MB-361) than in cell lines with normal *PPM1D* copy number (HCC1954 and SKBR3) (Fig. 5G).

## Discussion

In this work, we describe the *ERBB2* AS in 40 HER2+ tumors. We tested five HER2+ cell lines, three HER2+ xenographs and 57 HER2+ FFPE tumor samples. When testing HER2+ cell lines and xenographs using lcWGS a DM AS is found in most samples. In HCC1954 a single segment ID AS is found as previously described^12^, in SKBR3^28^ and BT474 an ID AS composed of several segments is found. In HER2+ tumor samples the common AS is ID. In most cases, the amplicon is composed of a single *ERBB2* containing segment while in a few cases, additional segments from the q-arm of chromosome 17 link to the *ERBB2* segment’s centromeric end. This may reflect a pathological process whereby most amplicons originate in a break-fusion-bridge mechanism^33^ leading to an ID AS. This is in line with the finding that in primary breast cancer, *ERBB2* amplification is found as an intra-chromosomal event^7^. This proposed order of events correlates with extent of disease: in patients suffering from a localized disease we found a single segment ID AS; in patients suffering from an advanced disease amplicons contain several segments. The cell lines and xenographs could be derived from advanced disease or may have undergone further selection.

Examining tumors using lcWGS can lead to further insight that could be clinically relevant. We found that characterizing HER2+ tumors using lcWGS could help determine if a tumor is a recurrent or a second primary tumor. We also identified co-amplification of oncogenes that may be clinically relevant. The most commonly clinically relevant co-amplified genes found are *MYC*, *PPM1D* and *RPS6KB1*, in three samples each. In 10% of HER2+ tumors *MYC* and *ERBB2* are co-amplified, serving as a poor prognostic factor^19^. Our results are in line with *MYC* amplification serving as a poor prognostic marker, of three patients with *MYC* and *ERBB2* co-amplification one suffered from metastatic disease, one from a local recurrence, and one from a localized disease. *MYC* is a transcription factor that regulates cell proliferation. Although no targets for direct inhibition were found, inhibition of transcription using BET inhibitors and modifying epigenetic context using HDAC inhibitors as well as inhibition of downstream pathways such as the PIK3CA pathway and CDK4/6 are currently studied^34^.

We find that *RPS6KB1* and *PPM1D* are located in the same amplicon, and that the *PPM1D/RPS6KB1* amplicon is different from the *ERBB2* amplicon. *RPS6KB1* encodes PS6K, a serine/threonine kinase that is a downstream target of mTOR^35^. PS6K phosphorylates the ribosomal protein S6 and is essential for transition to S-phase^36^. *RPS6KB1* amplification is found in both HER2+ and HER2- cell lines^28^. In HER2+ cell lines PS6K phosphorylation is a marker for resistance to trastuzumab treatment^37^. *PPM1D* and *ERBB2* co-amplification has been previously described^29^. *PPM1D* encodes wildtype p53-induced phosphatase 1 (WIP1) a protein serine/threonine phosphatase that promotes cell proliferation through downregulation of the DNA damage response and is amplified in various tumors^38,39^. Following DNA damage WIP1 is induced by wild type P53^40^, WIP1 de-phosphorylates MDM2 leading to increased P53 degradation^41^. WIP1 also de-phosphorylates key regulators of the DNA damage response such as ATM^42^. Thus WIP1 is a key player in the negative feedback loop of the P53 induced DNA damage response. WIP1 inhibitors are currently under development^39^. Specifically, HER2+ breast cancer cells were inhibited by GSK2830371 either alone or additively with a miR-21 inhibitor^43^ and breast cancer cells treated with a WIP1 inhibitor were sensitized to doxorubicin and to a MDM2 antagonist^32^.

We examined if co-amplification can identify a sub-group of HER2+ tumors where additional inhibitors could be effective. We show that in HER2+ cell lines WIP1 inhibitors effectively inhibits cell proliferation in two *PPM1D* amplified cell lines (BT474 & ZR-75-30) but not in another (MDA-MB-361). *TP53* is wild type in ZR-75-30 and mutated in BT474 and MDA-MB-361. An intact P53 pathway seems a prerequisite for WIP1 function and sensitivity to WIP1 inhibitors. While the P53 wild type ZR-75-30 and P53 mutated MDA-MB-361 cell lines fit this model the P53 mutated BT474 cell line does not. A possible explanation might be found in the different types of P53 mutations in MDA-MB-361 and BT474. MDA-MB-361 harbors a nonsense *TP53* mutation rendering the protein non-functional while BT474 harbors a missense mutation leading to an E285K mutation^44^. When expressed in *S. cerevisiae* the P53 E285K is active in a temperature sensitive manner^45^. Perhaps some P53 activity is retained in the BT474 cell line allowing P53 induction of WIP1 and conferring sensitivity to the WIP1 inhibitor. Further research is needed to better characterize the molecular pathways leading to sensitivity and resistance to WIP1 inhibitors in *PPM1D* and *ERBB2* co-amplified tumors in the context of different *TP53* mutations.

The main limit of this work is its retrospective nature that limits its ability to generalize our findings and exposes our population to bias such as survivorship bias. Additional limitations are the relatively small number of samples from advanced tumors and the lack of detailed analysis of the molecular mechanisms leading to sensitivity or primary resistance to WIP1 inhibitor in cell lines with *PPM1D/RPS6KB1* and *ERBB2* co-amplification. Further research including analysis of a larger number of patients, specifically with advanced disease is needed to validate the findings of this work. Analysis of large cohorts in a prospective manner is needed to understand if *ERBB2* AS can predict the benefit from anti-HER2 therapy. Understanding the molecular biology of WIP1 inhibitors could increase the potency of WIP1 inhibitors in a specific subsets of tumors with *PPM1D/RPS6KB1* and *ERBB2* co-amplification that may lead to improved cancer care.

To conclude, we propose that the initial mechanism of *ERBB2* amplification is break-fusion-bridge. This is based on the finding that in patients with a localized disease the AS is a single segment ID, while in patients with advanced disease an amplicon with ID AS composed of several segments is common. In addition, we found that characterizing HER2+ tumors using lcWGS could help determine if a tumor is a recurrent tumor or a second primary tumor, and identify co-amplified oncogenes that may predict the benefit of specific drug treatment.

## Methods

### Cell lines and xenographs

HCC1954 (ATCC Cat# CRL-2338, RRID:CVCL_1259) and ZR-75-30 (ATCC Cat# CRL-1504, RRID:CVCL_1661) were obtained from the American Type Culture Collection (ATCC) and maintained according to directions supplied with each cell line. The MCF7 (NCI-DTP Cat# MCF7, RRID:CVCL_0031) is a kind contribution of Prof. Ben-Porath, MDA-MB-361 (ATCC Cat# HTB-27, RRID:CVCL_0620) is a kind contribution of Prof. Lotem, SKBR3 (CLS Cat# 300333/p3803_SK-BR-3, RRID:CVCL_0033) is a kind contribution of Dr. Sonnenblick and BT474 (CLS Cat# 300131/p705_BT-474, RRID:CVCL_0179) is a kind contribution of Prof. Elkin (all of the Hebrew University, Hadassah medical center, Jerusalem, Israel). MDA-MB-361, BT474, HCC1954 and ZR-75-30 were cultured in RPMI-1640 medium (Biological Industries) supplemented with 10% FCS and 1% Penicillin/Streptomycin (P/S) and maintained at 37 C°, 5% CO2. The MCF7 cell line was cultured in Dulbecco’s Modified Eagle’s Medium (DMEM). Three HER2+ xenographs were obtained from Champions Oncology^©^ (166; 20983; 80990).

### Cell proliferation assay

Cells were seeded in 96 well culture plates at a density of 3 to 9 × 10^3^ cells/well depending on cell line. After overnight incubation the MCF7, BT474, MDA-MB-361, ZR-75-30 and SKBR3 cells were treated with either mock treatment, trastuzumab (Roche) as previously described^46,47^ or GSK2830371 (Sigma-Aldrich) alone or combined. The HCC1954 cells were treated with either mock treatment, erlotinib (Sigma-Aldrich) as previously described^48^ or GSK2830371 alone or combined. The drugs were added at a final concentration of 10 μgr/ml, 50 μgr/ml and 100 μgr/ml for trastuzumab; 0.1 μM, 0.5 μM and 1 μM for erlotinib; 1 μM and 2.5 μM for GSK2830371. Cell viability was measured on day 7 using the MTT proliferation assay kit (Promega, Madison, WI, USA) according to manufacturer instructions. The absorbance was measured at 595 nm on the Tecan sunrise microplate reader (Tecan Group Ltd, Mannedorf, Switzerland). The data shown are representative of at least three independent experiments and each treatment was performed in pentaplicate.

### Western blot analysis

Cells were solubilized for 20 min at 4° in lysis buffer containing 10 mM Tris-HCl, ph7.4, 150 mM NaCl, 1 mM EDTA, 1% Triton X-100, supplemented with a protease inhibitor cocktail including 5 mg/ml aprotinin, 1 mM phenylmethylsulfonylfluoride, PMSF, and 1 mM Na orthovanadate (Sigma, St. Louis, MO, USA). After centrifugation at 12000 rpm for 20 min the protein concentration was measured in the supernatants. Equal protein aliquots (50 μg) were loaded on a 12% SDS-PAGE, followed by transfer to an Immobilon-P membrane (Millipore, Bedford, MA, USA). Membranes were blocked and probed with the appropriate antibodies; anti WIP1 (F-10; mAb; sc-376257 Santa Cruz, Dallas Texas, USA, 1:500) and anti β-actin (Sigma Aldrich 1:5000). The membranes were washed and incubated with horseradish peroxidase - conjugated secondary antibody. The protein bands of interest were detected by the enhanced chemiluminescence (ECL) reagent (Pierce, Rockford, IL, USA).

### Patients and samples

The study design is a retrospective single institution study of sequential patients with HER2+ tumors. Tissue samples were obtained in accordance with the principles endorsed by the declaration of Helsinki and written informed consent was obtained from all subjects. Protocols were approved by the institutional review board of Hadassah-Hebrew University Medical Center. A board qualified pathologist determined the diagnosis and percent of tumor cells in the sample. 53 patients are included in this study, 43 patients with localized disease (American Joint Committee on Cancer 7^th^ edition (AJCC stage IA-IIIA), seven patients with advanced disease (AJCC stage IIIC-IV) and three patients with recurrent disease. Of 53 patients, four harbor two tumors, a total of 57 tumors were examined. DNA was successfully extracted from 55 tumors and tested using ddPCR, 42 tumors were found positive and were further tested using lcWGS. The 40 tumors where *ERBB2* is found, using lcWGS, in 6 or more copies are described.

### DNA extraction

We extracted DNA from cell lines and xenographs using the DNeasy blood and tissue kit (Qiagen) according to the manufacturer’s protocol. A qualified pathologist identified Formalin-fixed, paraffin-embedded (FFPE) tumor tissue blocks and sections were prepared, the tumor tissue was marked on hematoxylin and eosin stained slides. The tissue was scraped from unstained slides and the DNA extracted using the QIAamp DNA FFPE tissue kit (Qiagen) as described by the manufacturer. Briefly, we removed the paraffin and extracted the DNA using optimized lysis conditions followed by incubation at an elevated temperature to remove formalin crosslinking. The DNA was then bound to a silica based membrane column, washed and eluted. Extracted DNA concentration was measured using Nanodrop spectrophotometer ND-1000 (Thermo Scientific) and Qubit 2.0 fluorometer (Invitrogen).

### Evaluation of ERBB2 copy number (CN) with ddPCR

We analyzed the DNA samples using Digital Droplet PCR (Bio-Rad). The Taqman PCR reaction was prepared in a final volume of 20 μl. We mixed 20 ng of genomic DNA with ddPCR supermix (Bio-Rad), target *ERBB2* primers and probe (FAM), reference AP3B1 primers and probe (HEX) (Bio-Rad). 20 μl of the ddPCR reaction mix and 70 μl of the droplet generation oil (Bio-Rad) were loaded into 8 well cartridges (Bio-Rad) and placed into the droplet generator (Bio-Rad). We transferred the resulting droplets into a 96 well PCR plate. Thermal cycling conditions were 95 °C for 10 min (1 cycle), 94 °C for 30 sec and 60 °C for 60 sec (40 cycles), 98 °C for10 min (1 cycle) and 4 °C hold. After thermal cycling, we transferred the PCR reaction plate into the QX200 Droplet Reader (Bio-Rad) and quantified fluorescence in each droplet according to the manufacturer’s protocol. We performed CN analysis using the Quantasoft analysis software (Bio-Rad). The ratio between the concentration of the target *ERBB2* DNA and the reference *AP3B1* (2 copies) was used to calculate the *ERBB2* CN in each sample as described by the manufacturer. Values of 6 copies or more were considered positive.

### Whole genome library preparation

We fragmented genomic DNA to a target size of 300 bp on the Covaris M220. We prepared libraries using the NEBNext Ultra DNA Library Preparation Kit for Illumina and the Multiplex Oligos Kit for indexes (New England Biolabs), using 50–100 ng of genomic DNA input for each sample. The preparation involves end repair, dA tailing, adapter ligation, cleanup with Agencourt AMPure magnetic beads, PCR enrichment with incorporation of indexes and PCR cleanup. We quantified the final libraries using Qubit and library size was determined using the Agilent TapeStation 2200 as described by the manufacturer.

### Whole genome sequencing of the libraries

We denatured the libraries and loaded them on the Illumina NextSeq. 500 platform at a concentration of 1.8 pM. Paired-end sequencing was performed using the NextSeq. 500 High Output v2 kit (75 cycles) as described by the manufacturer, resulting in a median sequencing depth of 9X (3-15X).

### Bioinformatics workflow

**Figure 6:**
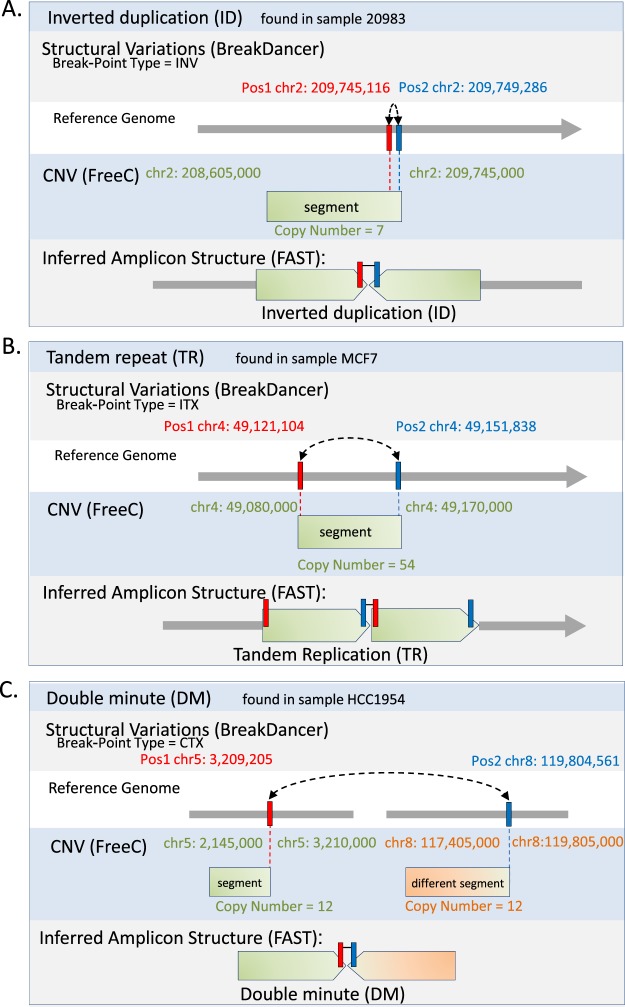
Identification of amplicon structure (AS) using copy number variation (CNV) and structural variation (SV). Following low coverage whole genome sequencing of tumor DNA we detected and analyzed structural variation (SV) using BreakDancer and enumerated copy number (CN) using Control-FREEC. The data was integrated and further analyzed using FindAmpliconSTructure: FAST, to identify regions with genomic amplification and infer amplicon structure (AS). (**A**) Identification of inverted duplication (ID) based amplification: A break-point of type Inversion (INV) was found by BreakDancer between Pos1 and Pos2 (chr2: 209,745,116 and chr2: 209,749,286). A segment (chr2:208,605,000-209,745,000 in 7 copies) was found by FreeC, the right edge of which coincides with Pos1 and Pos2. This combination delineates an amplicon structure of Inverted duplication (ID), in which the segment is repeated head-to-head. (**B**) Identification of tandem repeat (TR) based amplification: A break-point of type Internal-Translocation (ITX) was found by BreakDancer between Pos1 and Pos2 (chr4: 49,121,104 and chr4: 49,151,838). A segment (chr4: 49,080,000-49,170,000 in 54 copies) was found by FreeC, coinciding with Pos1 and Pos2, as shown. This combination delineates an amplicon structure of Tandem Repeat (TR), in which the segment is repeated head-to-tail. (**C**) Identification of double minute (DM) based amplification: A break-point of type inter-chromosomal translocation (CTX) was found by BreakDancer between Pos1 and Pos2 (chr5: 3,209,205 and chr8: 119,804,561). Two segments were found by FreeC (chr5: 2,145,000-3,210,000 in 12 copies, and chr8: 117,405,000-119,805,000 in 12 copies), one edge of each segment coincides with one of the break-points’ positions. This combination delineates an amplicon structure of Double Minute (DM), in which segments from different chromosomes are linked. The following link to a session in UCSC Browser^53^ shows the CN and SV tracks of samples.

We performed alignment of short paired-end sequences to the reference genome hg19 using BWA-ALN algorithm^49^. We removed potential PCR duplicates using the MarkDuplicates tool from Picard (http://broadinstitute.github.io/picard/). CN was enumerated using Control FREEC^50^ and structural variations were detected and analyzed using BreakDancer^51^ algorithm. In brief, Control FREEC takes as input the aligned reads and constructs a copy number frequency profile. The profile is then normalized, segmented and analyzed in order to assign the copy number to each genomic region. The results are available at https://datadryad.org/review?doi=doi:10.5061/dryad.32t0b4m. In brief, BreakDancer read pairs mapped to a reference genome with sufficient mapping quality are independently classified into six types: normal, deletion, insertion, inversion, intrachromosomal translocation and interchromosomal translocation. This classification process is based on (i) the separation distance and alignment orientation between the paired reads, (ii) the user-specified threshold and (iii) the empirical insert size distribution estimated from the alignment of each library contributing genome coverage. The algorithm then searches for genomic regions that anchor substantially more anomalous read pairs than expected on average. A putative structural variant is derived from the identification of one or more regions that are interconnected by at least two anomalous read pairs. A confidence score is estimated for each variant based on a Poisson model that takes into consideration the number of supporting anomalous read pairs, the size of the anchoring regions and the coverage of the genome. The dominant type of associated anomalous read pairs in a particular region determines the type of structural variant. The start and the end coordinates are defined as the inner boundaries of the constituent regions that are closest to the suspected breakpoints, and the size is estimated by subtracting the mean insert size from the average spanning distance in each library and then averaging across libraries. To define AS the output of FreeC and BreakDancer was further analyzed using FindAmpliconSTructure: FAST, https://github.com/zick-lab/FAST, a tool we developed (Fig. 6). In brief, FAST reads the CNV file from Control FREEC and SV file from BreakDancer and performs some preprocessing such as removing segments below a defined threshold of six CN, breakpoints with a score below 99, and unifies consecutive segments. Next, segments are indexed and their location is assigned, based on SV data, segments are connected to an amplicon. Based on segment location and SV, the amplicon type is determined as ID, DM or TR. The results are visualized using Circus^52^. The following link to a session in UCSC Browser^53^ shows the CN and SV tracks of samples.

### Statistical analysis

To test contingency we used Fisher’s exact test.

### Ethics approval and consent to participate

This study were approved by the Ethics Committee of the Hadassah Medical Center.

## Supplementary information

Title page and supplementary figure 1

Supplementary Tables
