# Author Correction: Clinical Implications of Sub-grouping HER2 Positive Tumors by Amplicon Structure and Co-amplified Genes

**DOI:** 10.1038/s41598-020-60492-7

**Published:** 2020-02-27

**Authors:** Myriam Maoz, Michal Devir, Michal Inbar, Ziva Inbar-Daniel, Dana Sherill-Rofe, Idit Bloch, Karen Meir, David Edelman, Salah Azzam, Hovav Nechushtan, Ofra Maimon, Beatrice Uziely, Luna Kadouri, Amir Sonnenblick, Amir Eden, Tamar Peretz, Aviad Zick

**Affiliations:** 1 Sharett Institute of Oncology, Hebrew University-Hadassah Medical Center, Jerusalem, Israel; 2 Department of Pathology, Hebrew University-Hadassah Medical Center, Jerusalem, Israel; 3 The Oncology Division, Tel Aviv Sourasky Medical Center, Tel Aviv, Israel; 4 Department of Cell & Developmental Biology, Institute of Life Sciences, The Hebrew University of Jerusalem, Edmond J. Safra Campus, Givat Ram, Jerusalem Israel

Correction to: *Scientific Reports* 10.1038/s41598-019-55455-6, published online 11 December 2019

A supplementary file containing Tables S1, S2 and S3 was omitted from the original version of this Article. This has been corrected in the HTML version of the Article; the PDF version was correct at time of publication.

