# Supplementary material for: Clinical Implications of Sub-grouping HER2 Positive Tumors by Amplicon Structure and Co-amplified Genes
Source: Sci Rep. 2019 Dec 11;9:18795. doi: 10.1038/s41598-019-55455-6 (PMC6906288; doi:10.1038/s41598-019-55455-6)
Supplement: Supplementary file 1 — Title page and supplementary figure 1 [file 41598_2019_55455_MOESM1_ESM.pdf]

Clinical Implications of Sub-grouping HER2 Positive Tumors by Amplicon Structure and Co-amplified Genes

Myriam Maoz, Michal Devir, Michal Inbar, Ziva Inbar-Daniel, Dana Sherill-Rofe, Idit Bloch, Karen Meir, David Edelman, Salah Azzam, Hovav Nechushtan, Ofra Maimon, Beatrice Uziely, Luna Kaduri, Amir Sonnenblick, Amir Eden, Tamar Peretz, Aviad Zick.

List of samples in order of appearance:

p-2
p-3
p-5
p-6
p-8
m-11
m-12
m-13
p-16
p-17
p-20
p-21
p-22
p-23
p-25
p-27
p-28
p-29
p-30
p-33
p-102
p-103
p-104
p-106
p-108
p-112
p-114
p-115
p-119
166
p-203
p-204
p-205
p-209
p-214
p-215
p-216
p-217
p-218
p-6890
p-8191
20983
80990
BT474
HCC1954
MCF7
MDA361
SKBR3
ZR-75-30

p-2

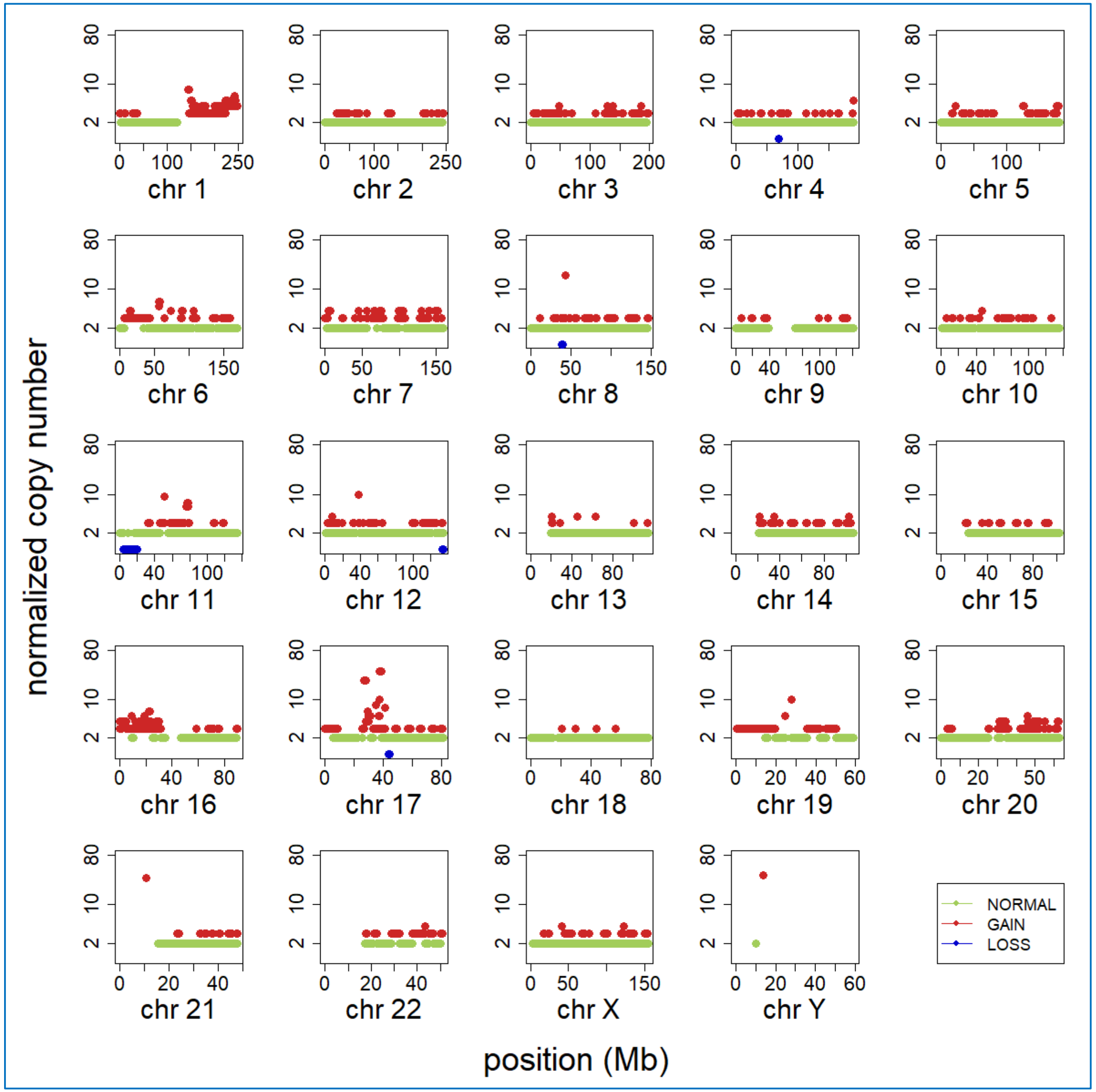

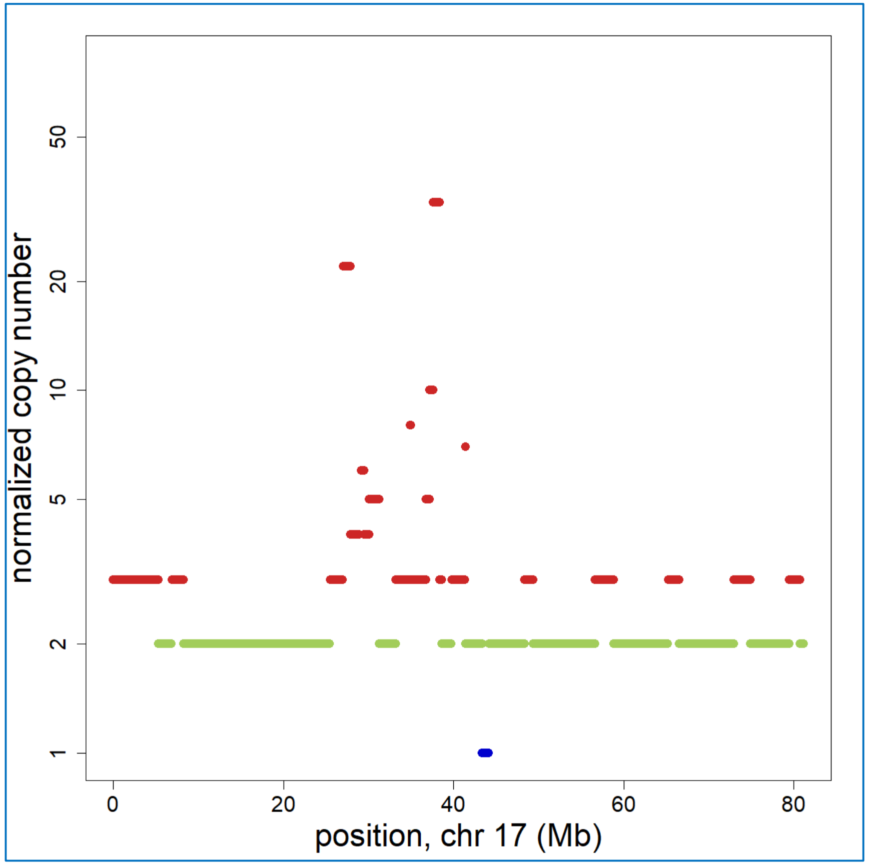

Control-FREEC

p-2

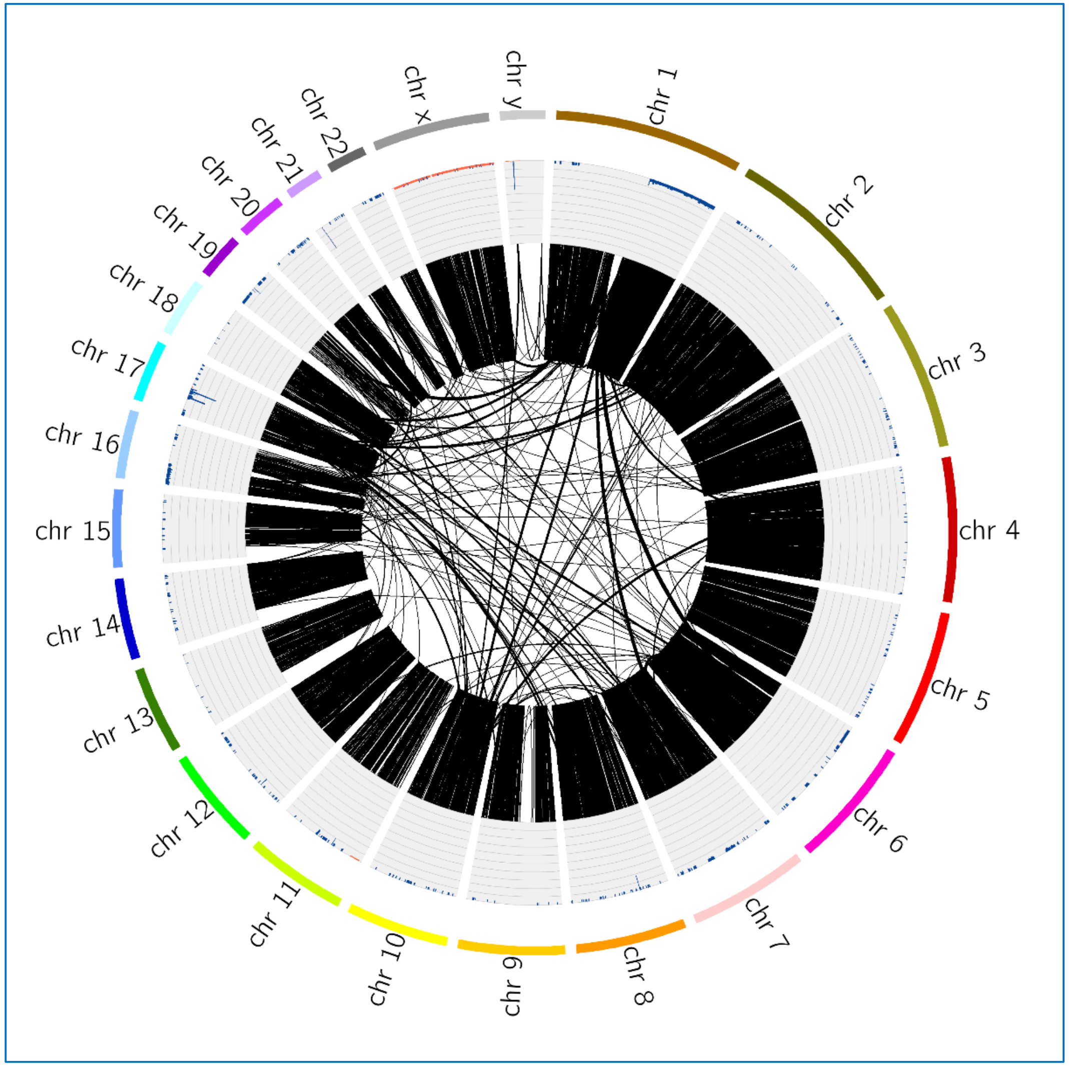

BreakDancer + Control-FREEC

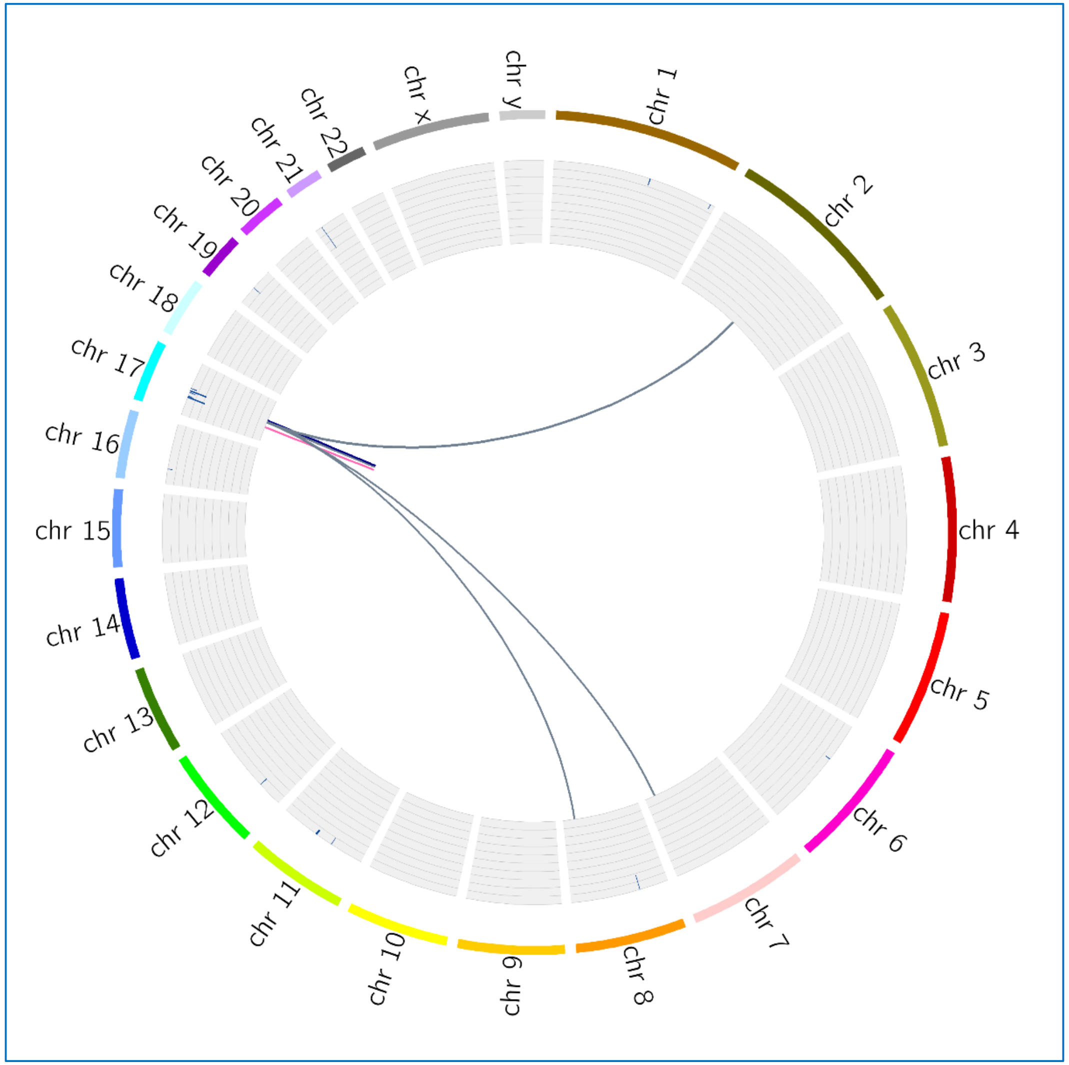

FAST – Whole Genome

p-2

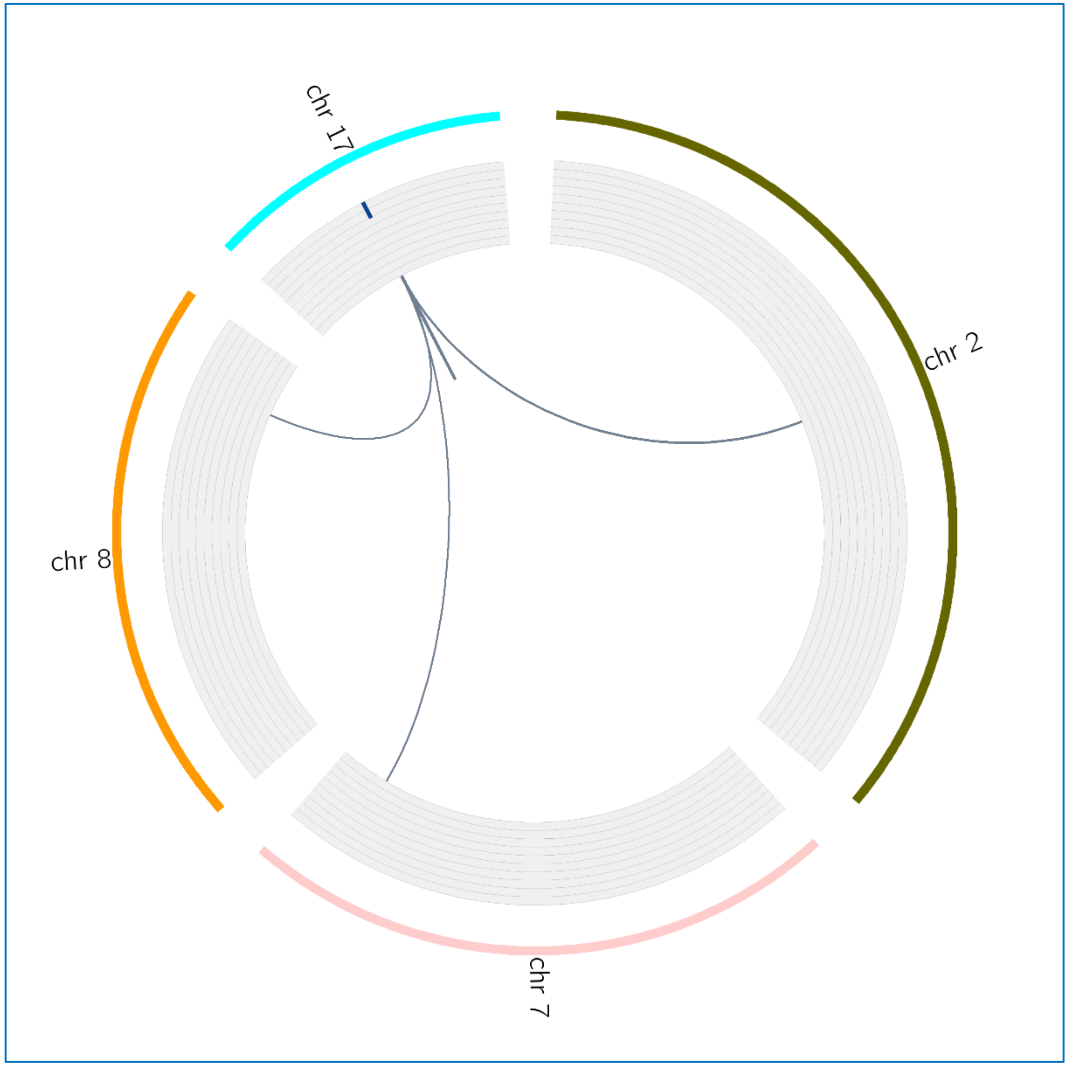

FAST – ERBB2 amplicon

p-3

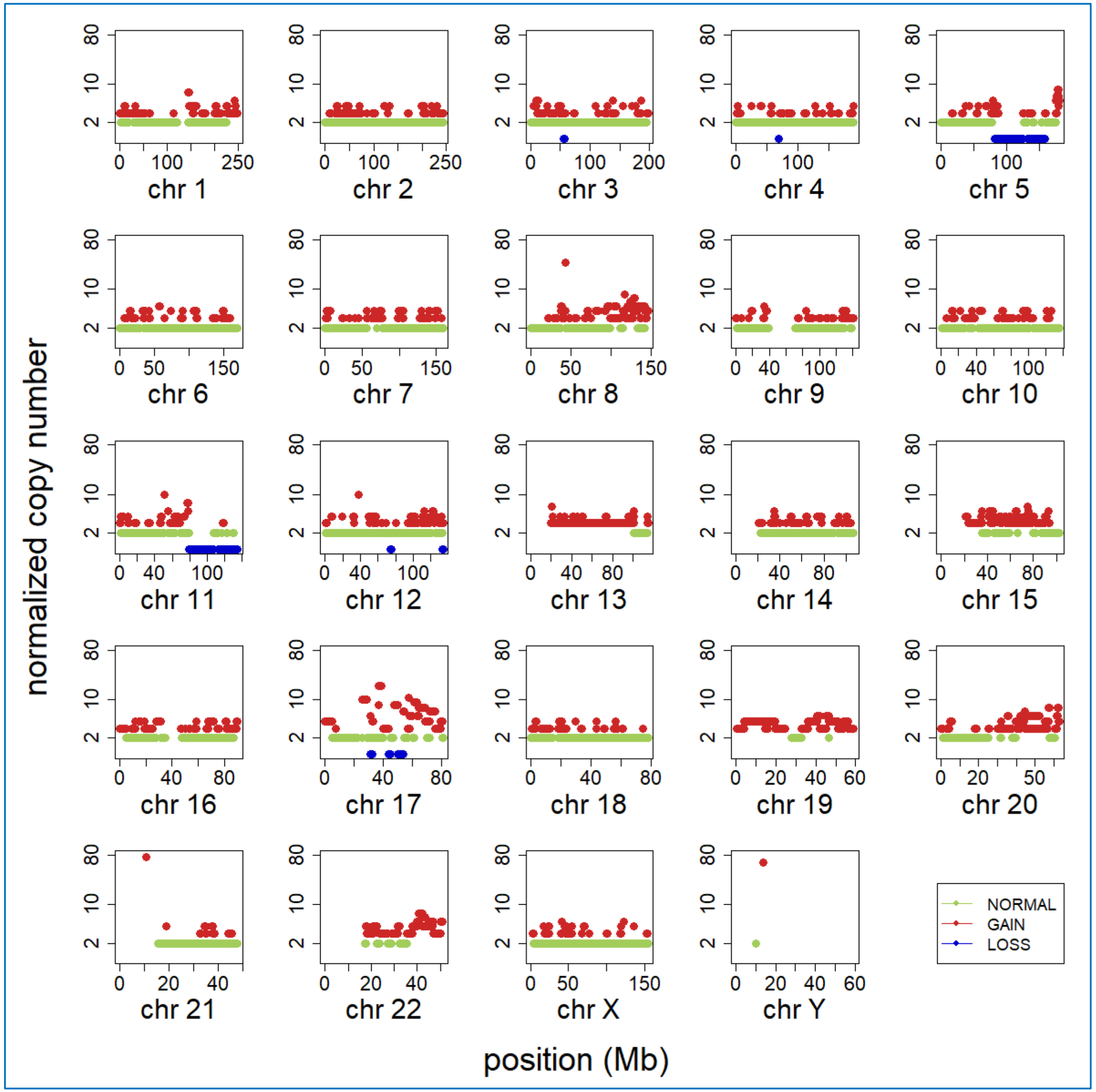

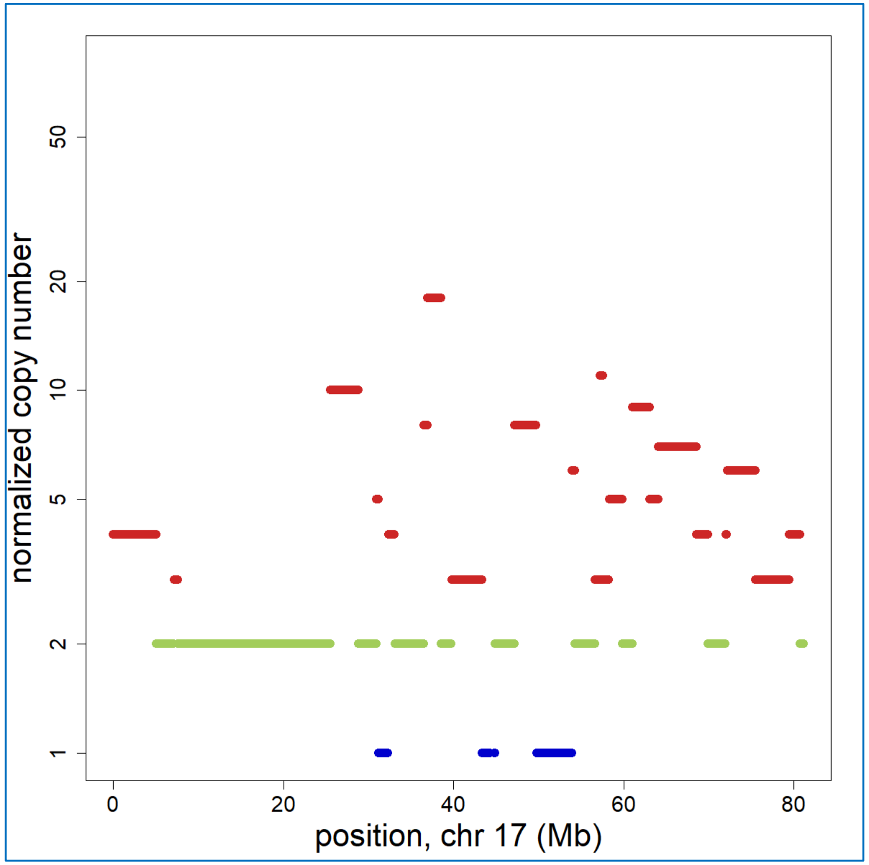

Control-FREEC

p-3

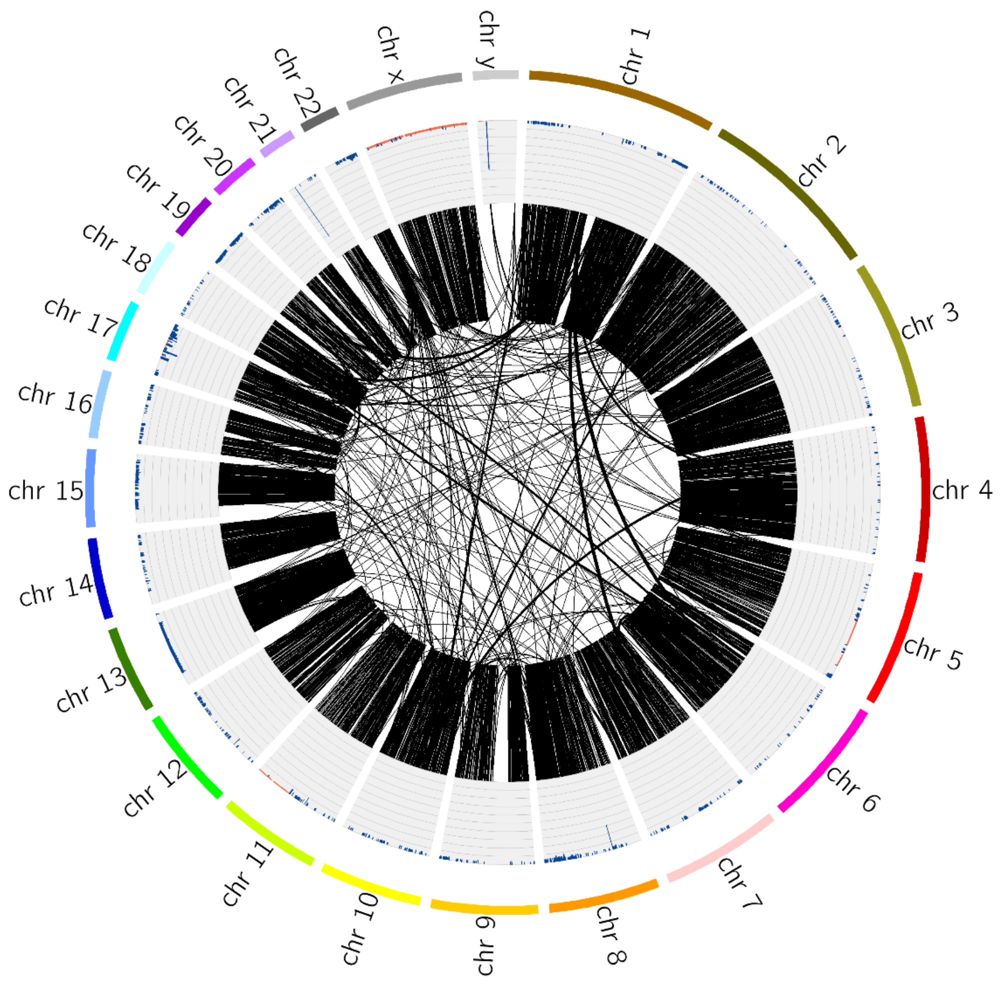

No FAST data for this sample

BreakDancer + Control-FREEC

FAST – Whole Genome

p-3

No FAST data for this sample

FAST – ERBB2 amplicon

p-5

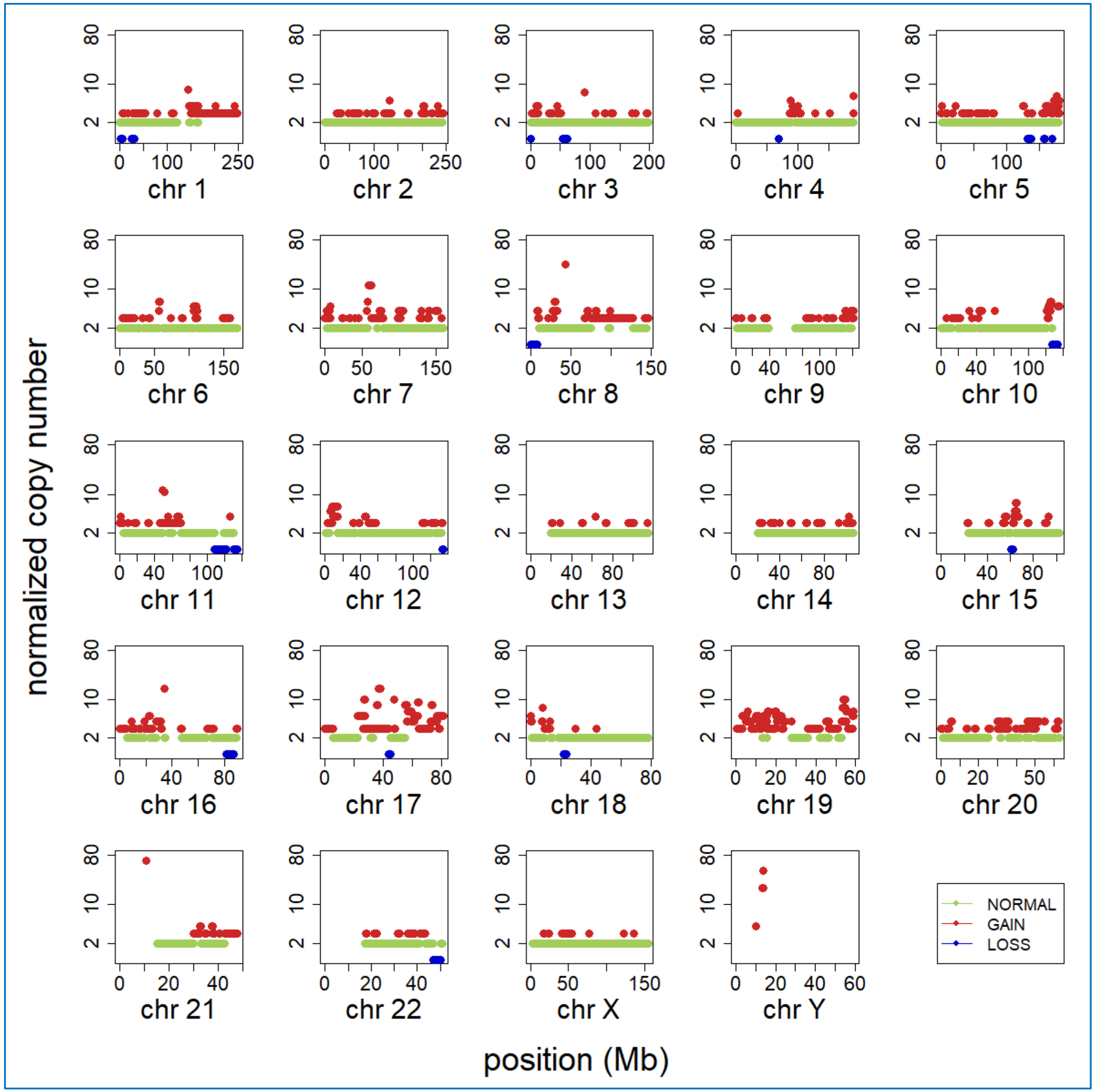

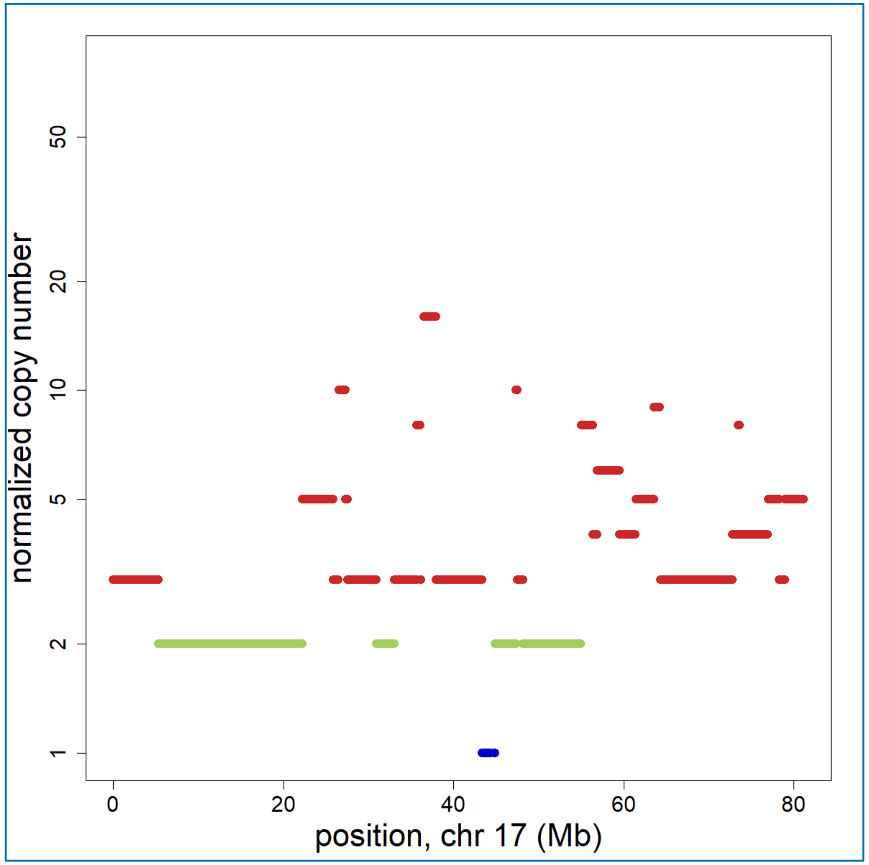

Control-FREEC

p-5

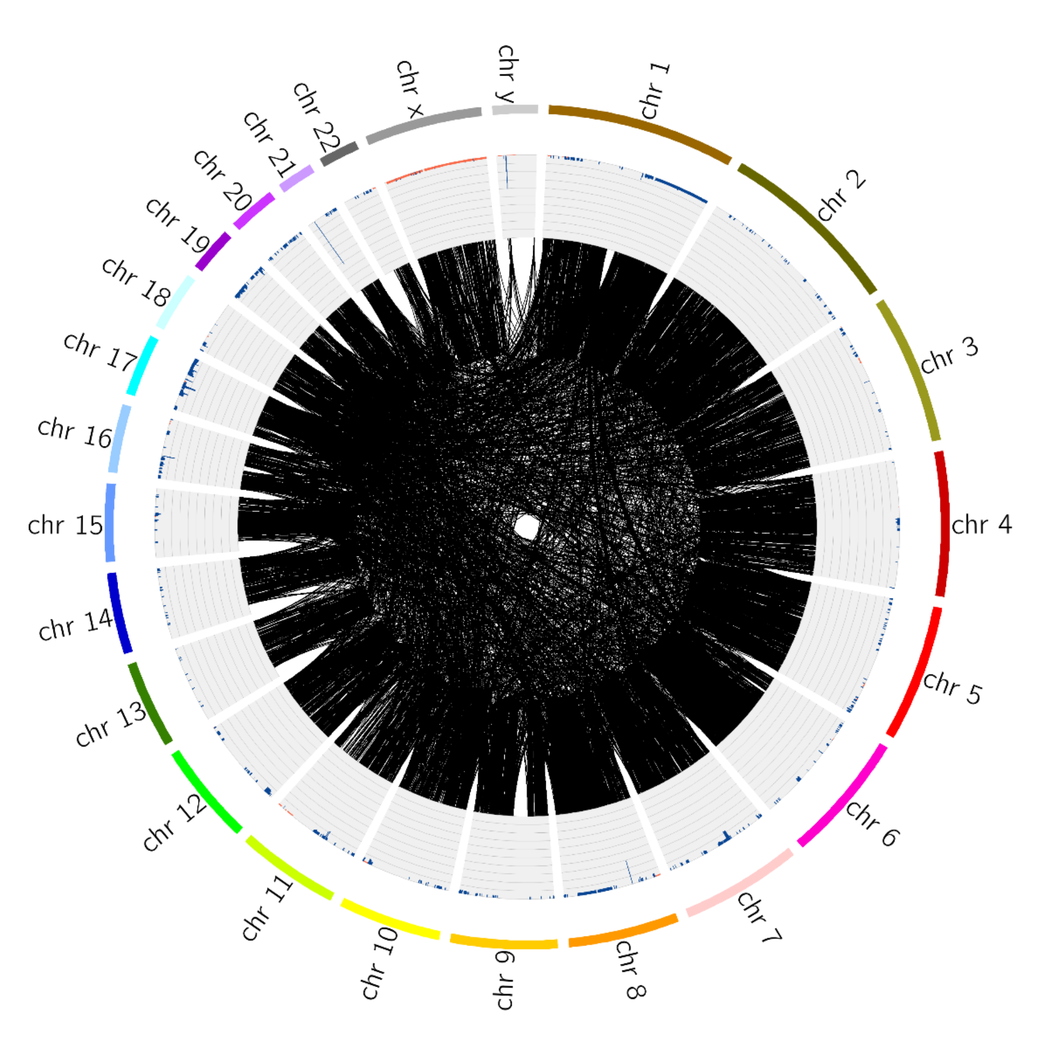

BreakDancer + Control-FREEC

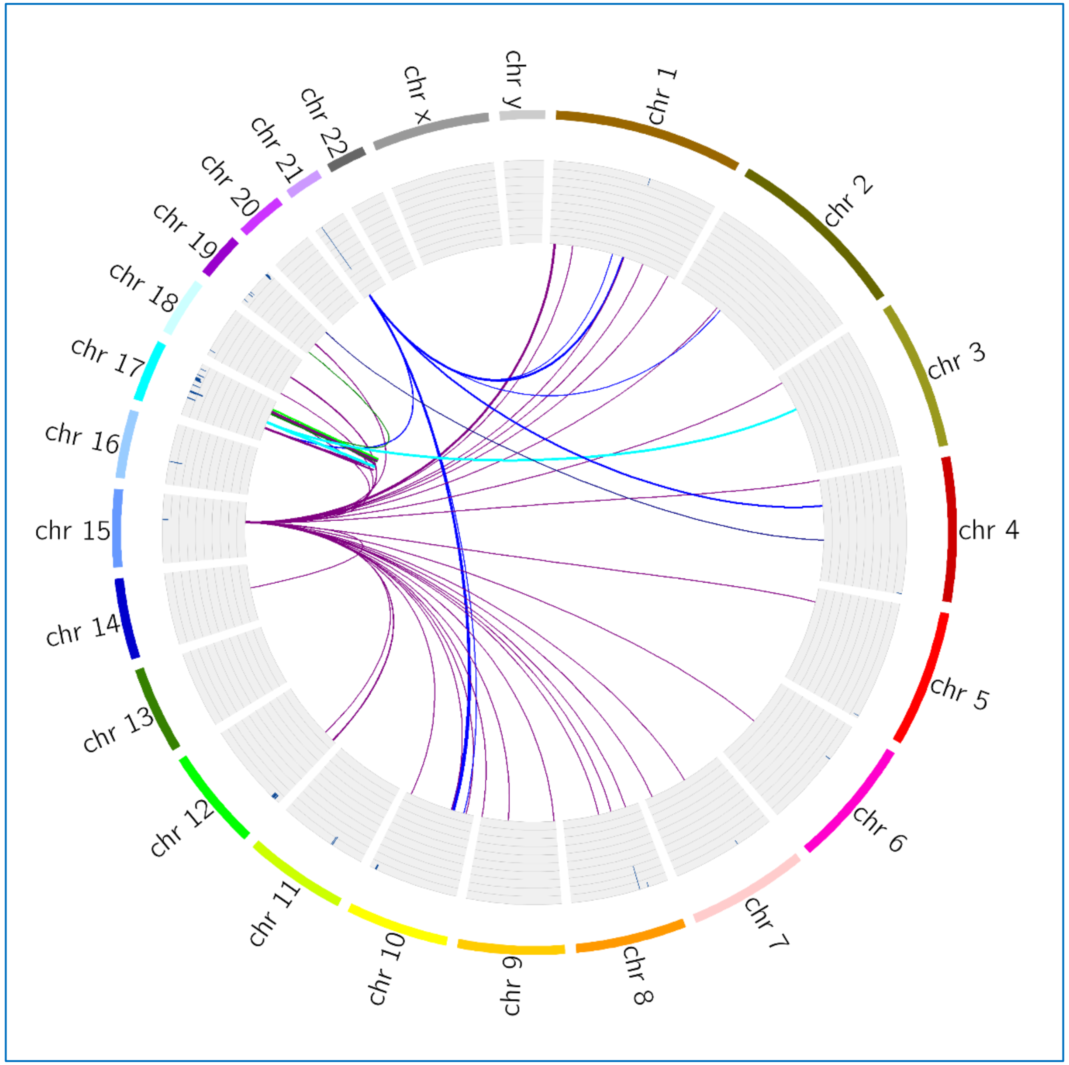

FAST – Whole Genome

p-5

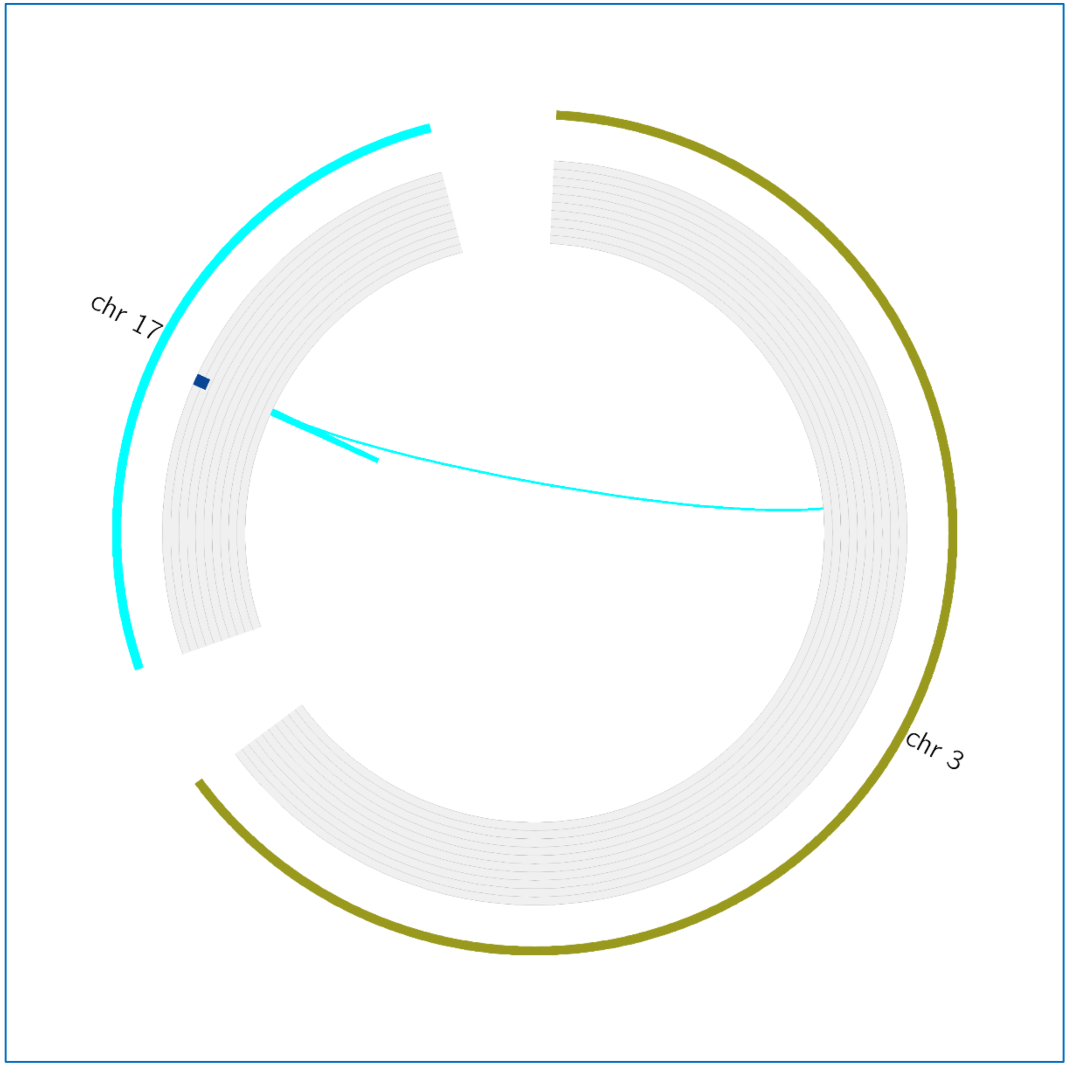

FAST – ERBB2 amplicon

p-6

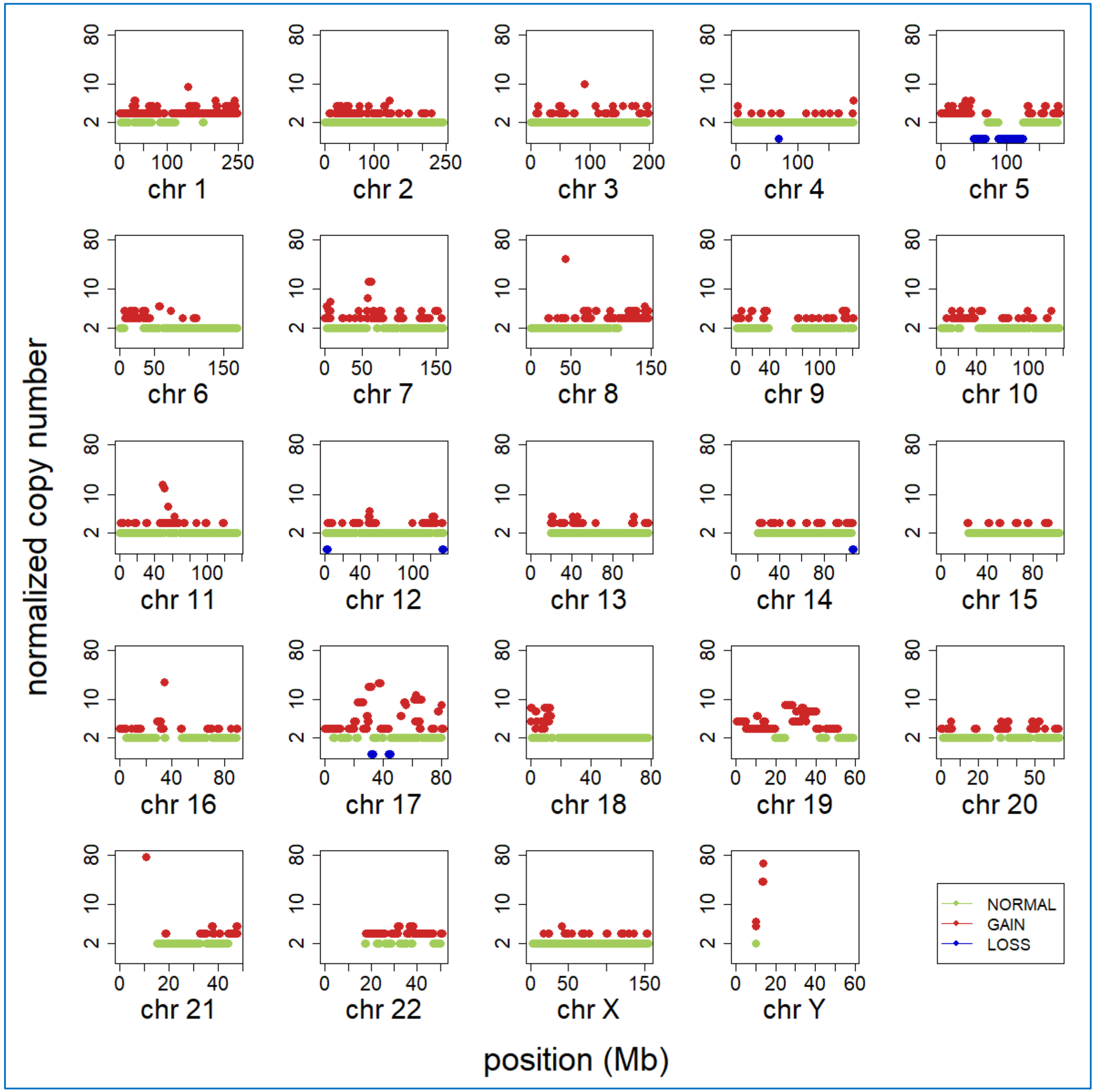

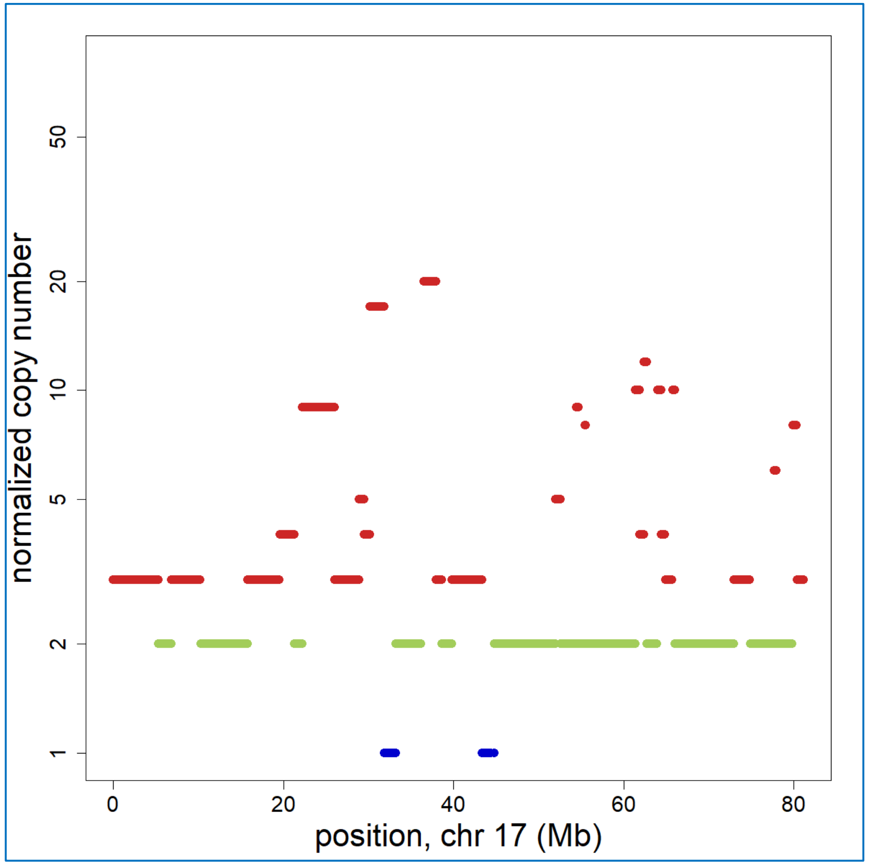

Control-FREEC

p-6

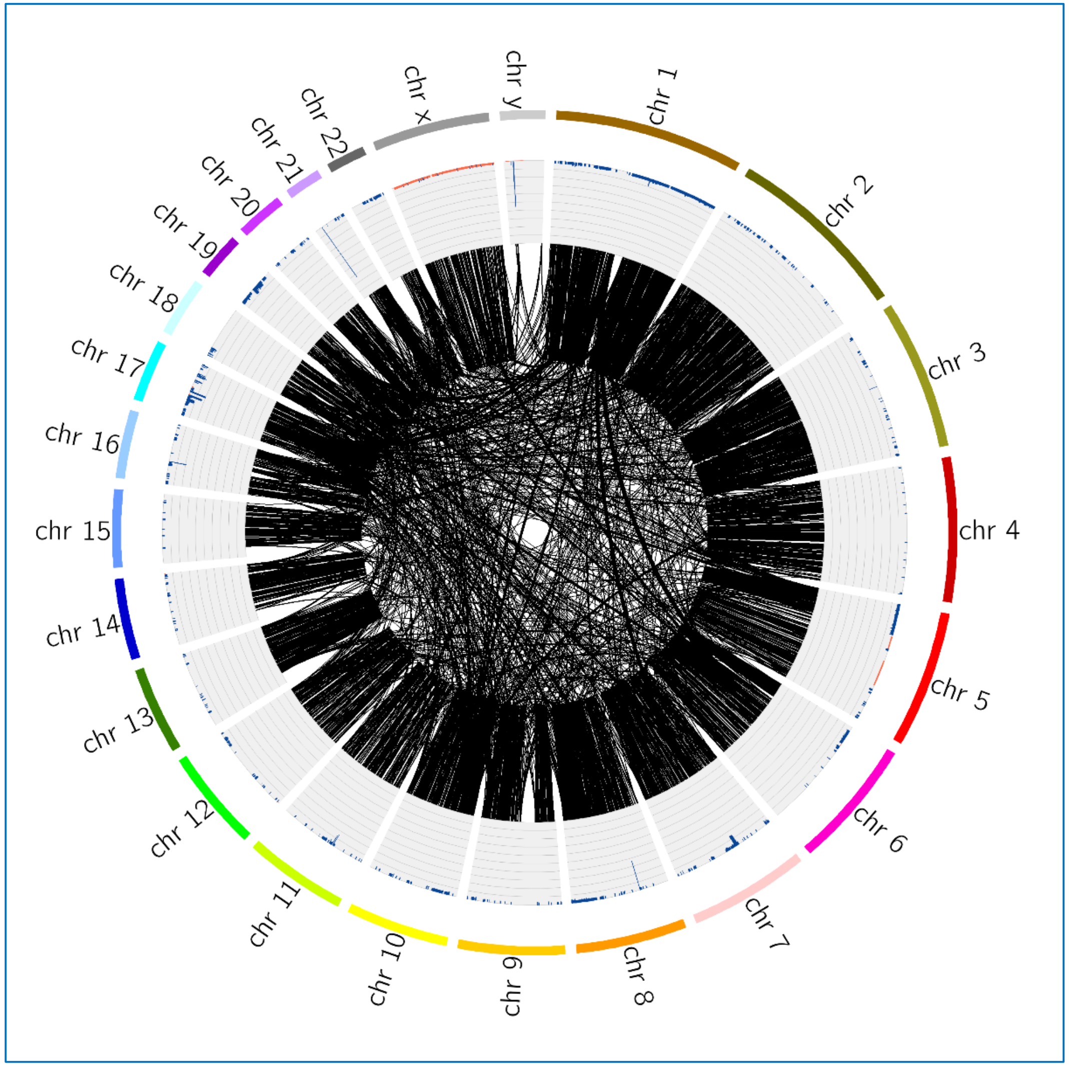

BreakDancer + Control-FREEC

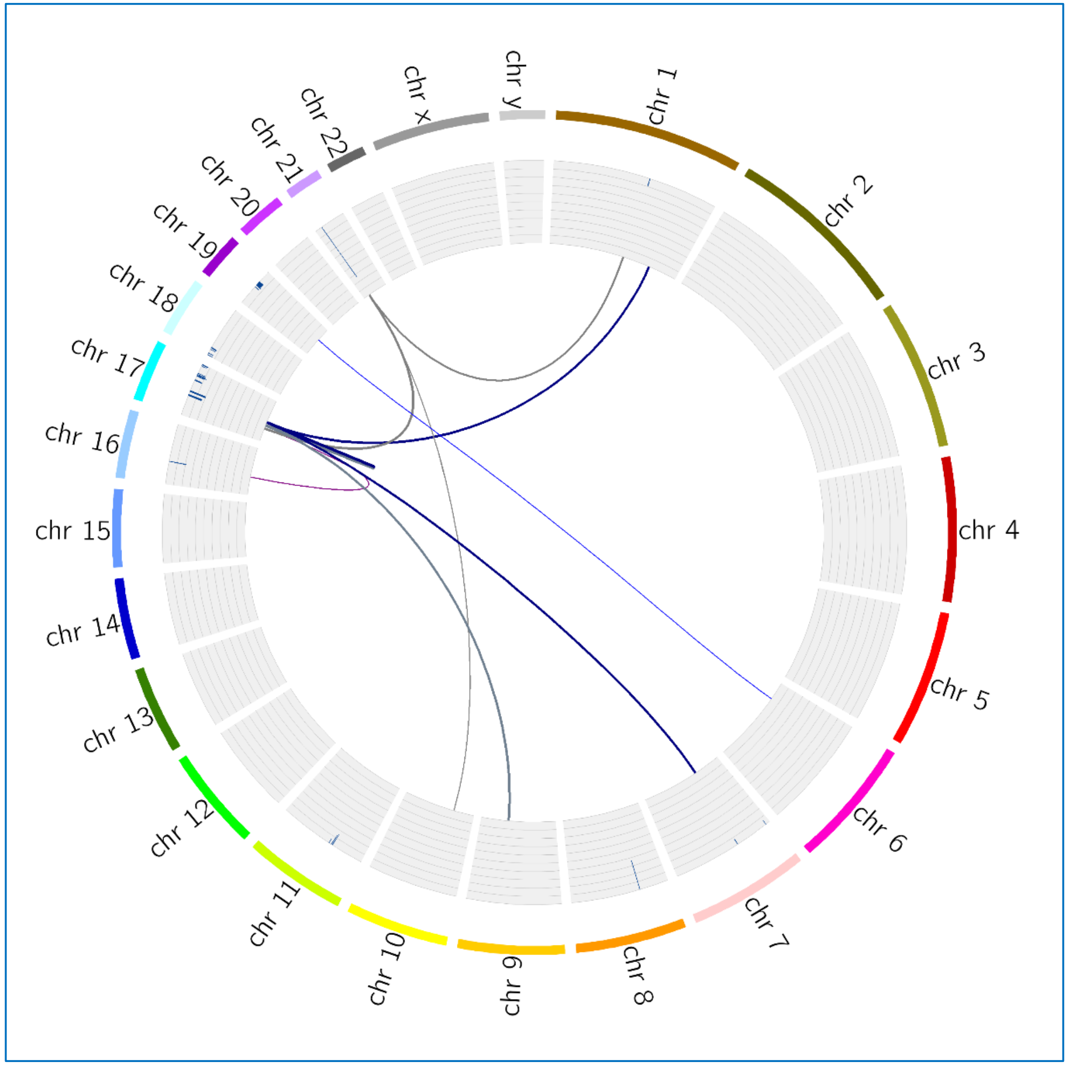

FAST – Whole Genome

p-6

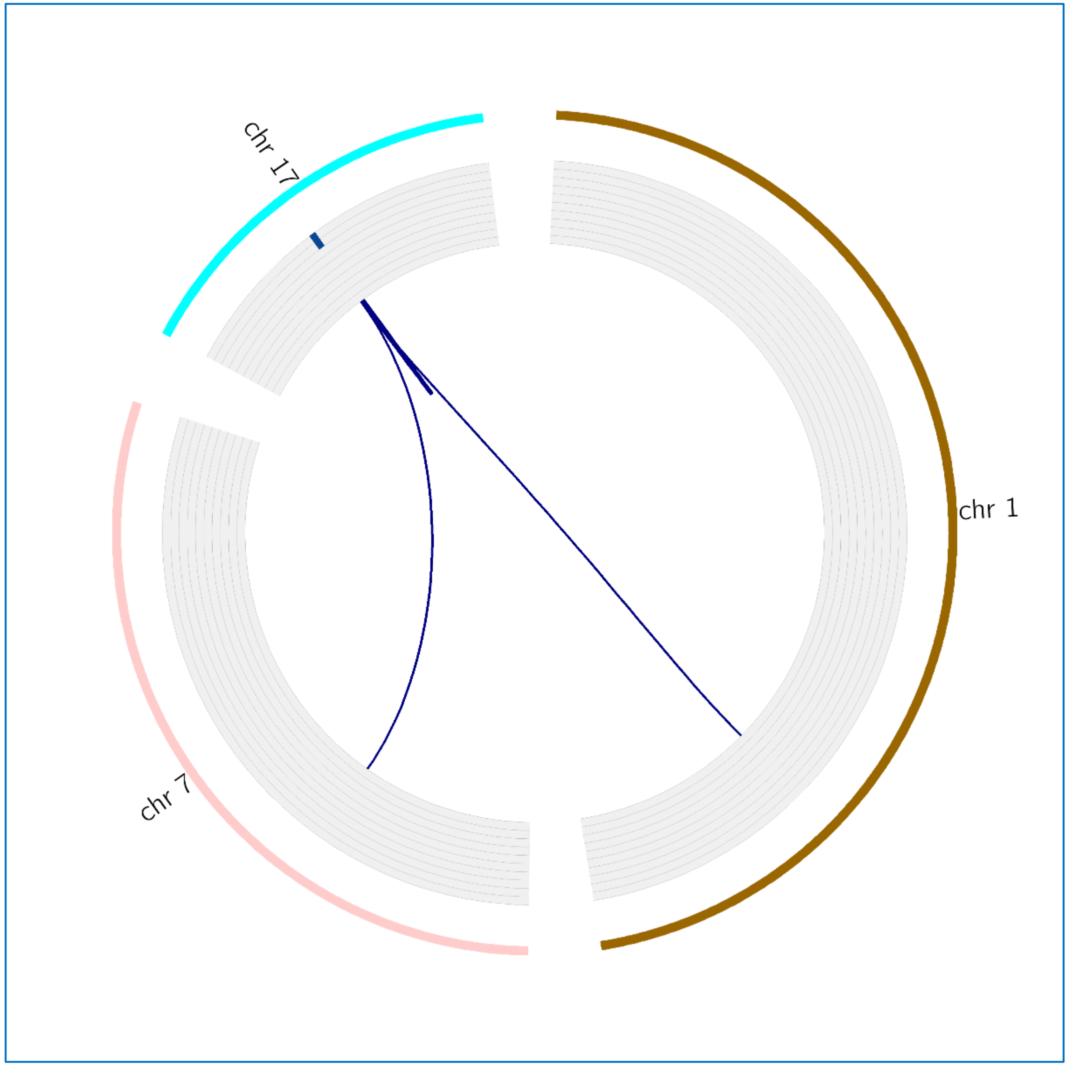

FAST – ERBB2 amplicon

p-8

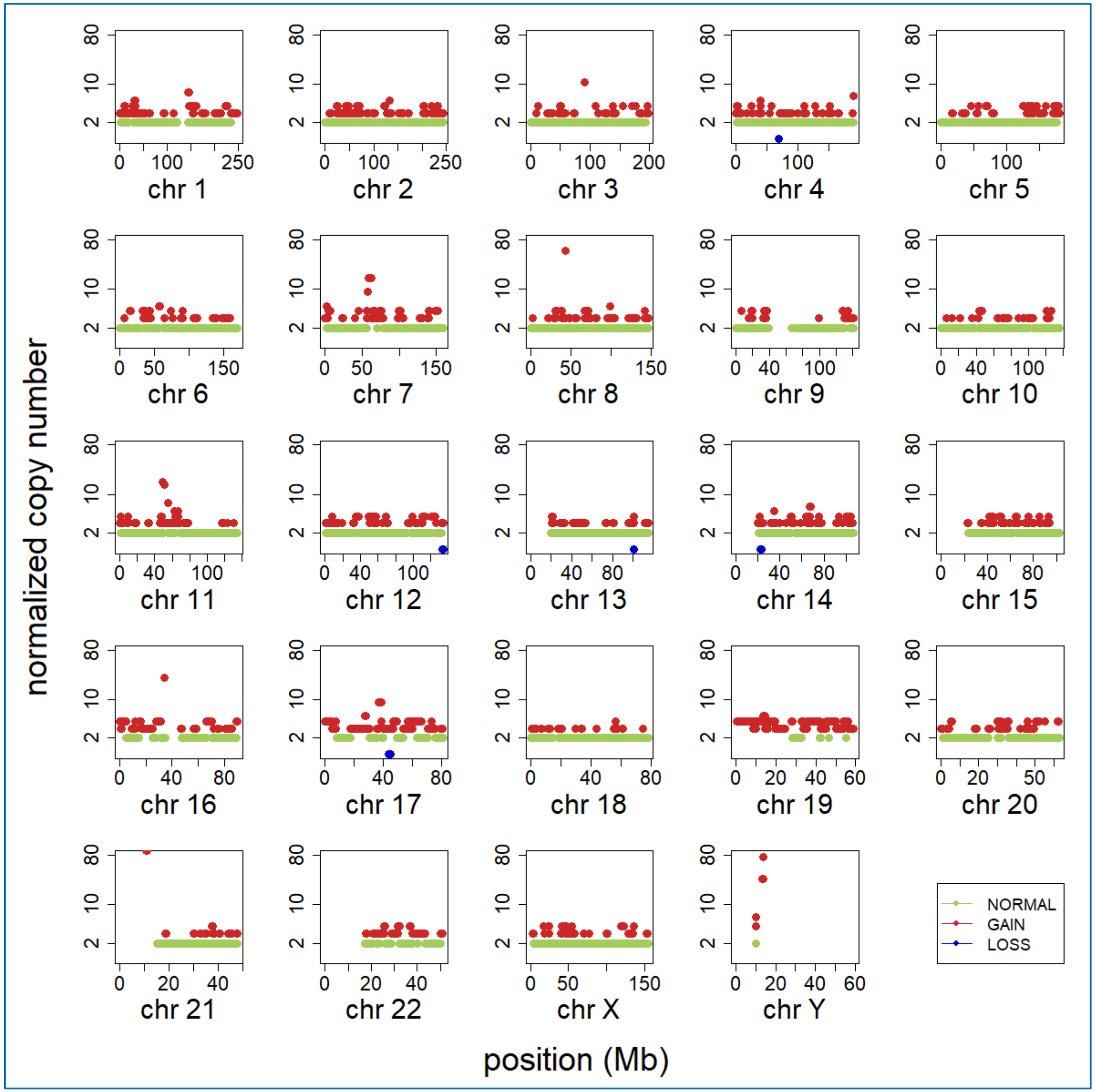

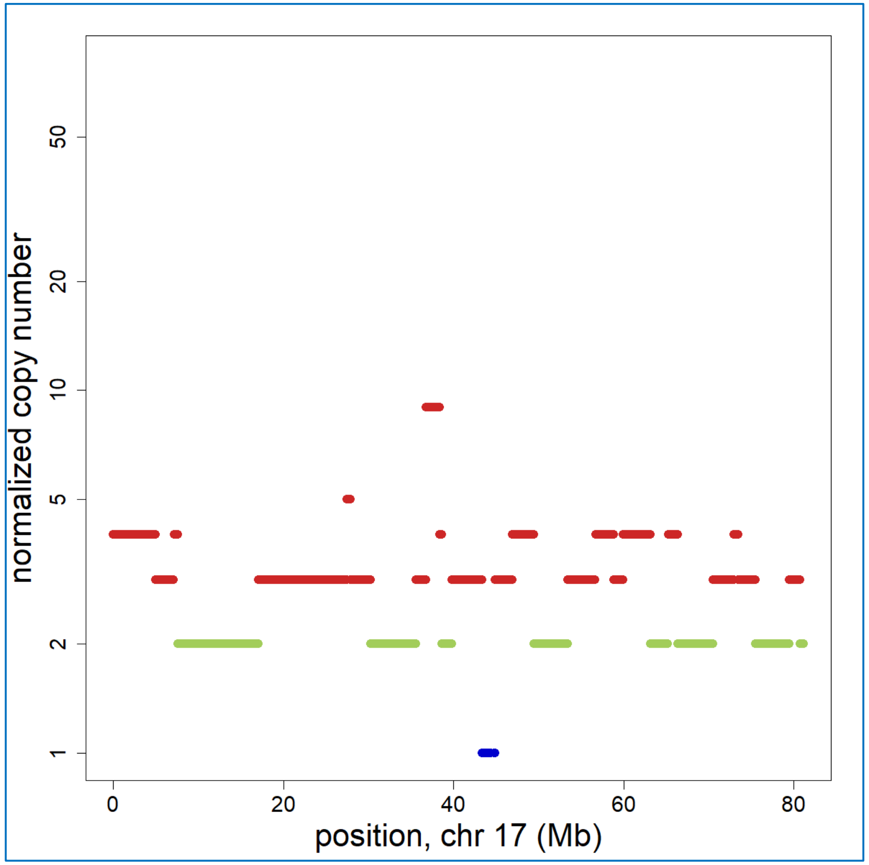

Control-FREEC

p-8

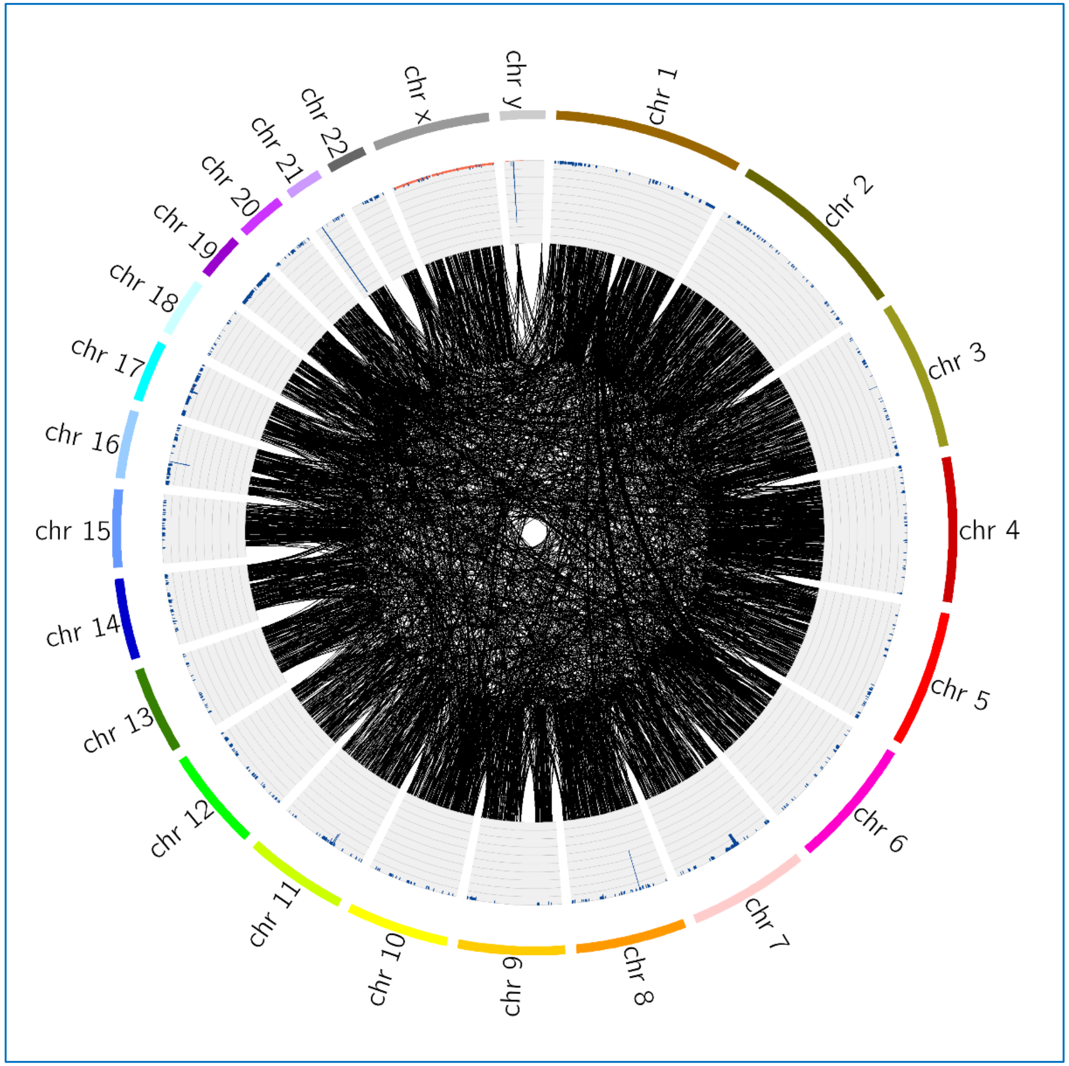

BreakDancer + Control-FREEC

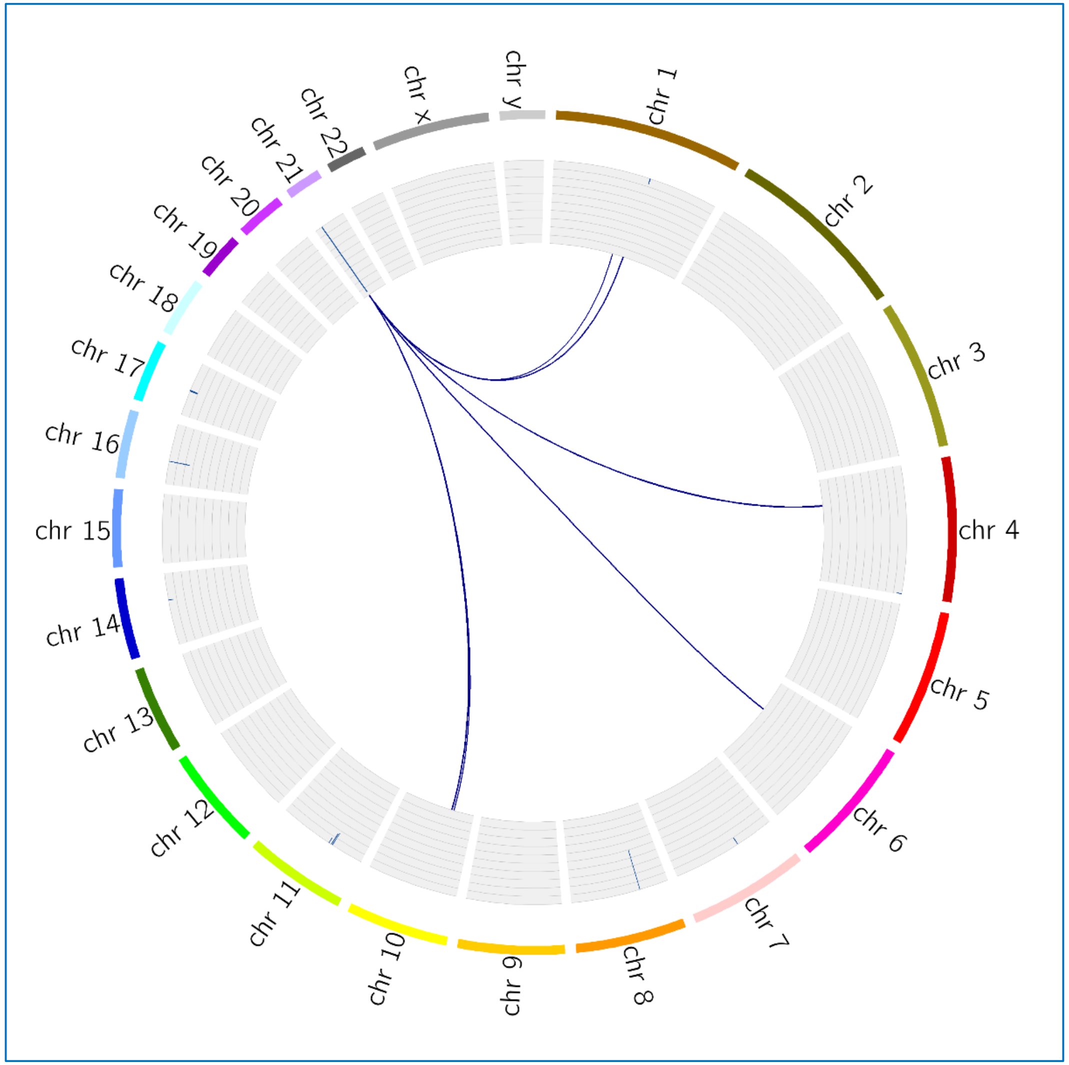

FAST – Whole Genome

p-8

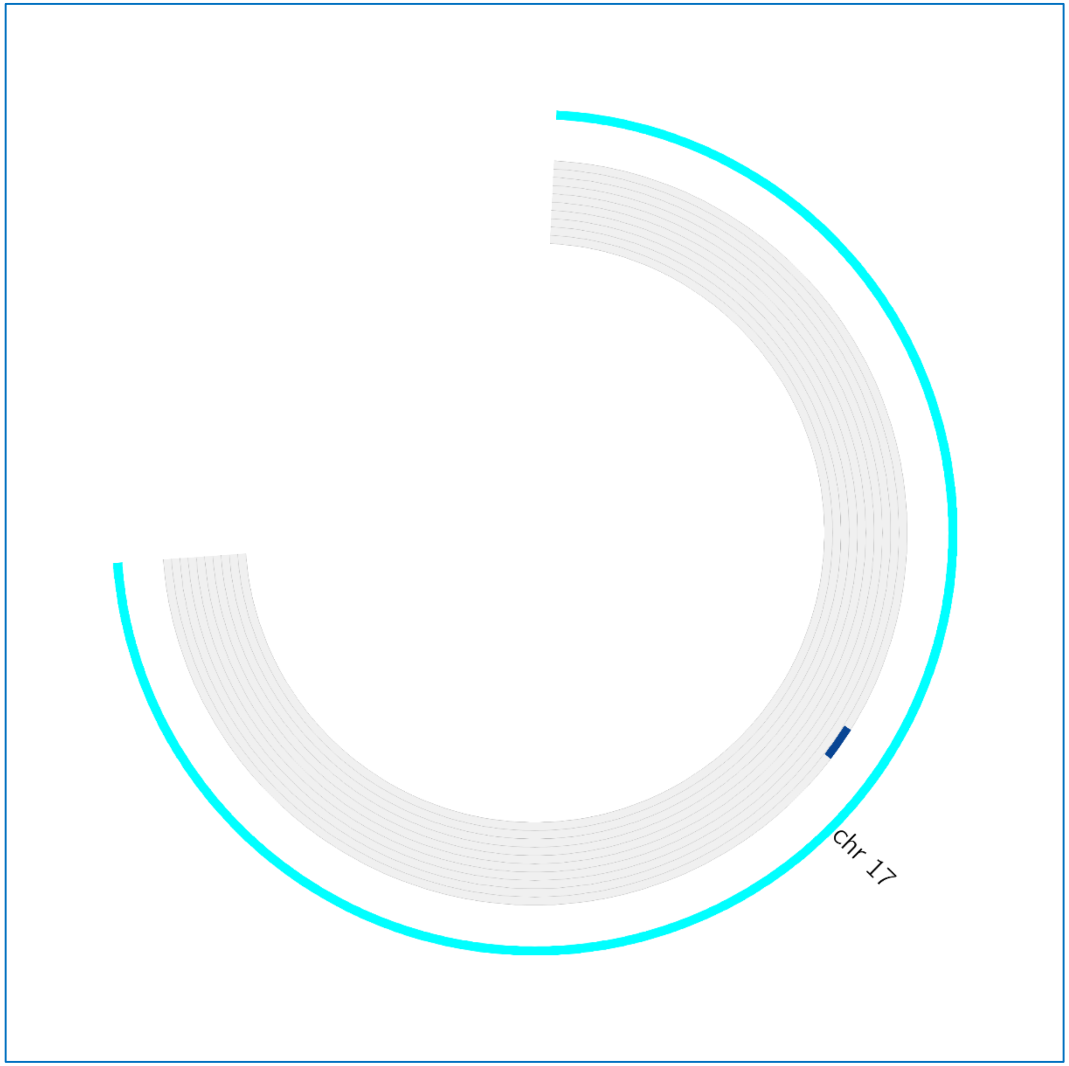

FAST – ERBB2 amplicon

m-11

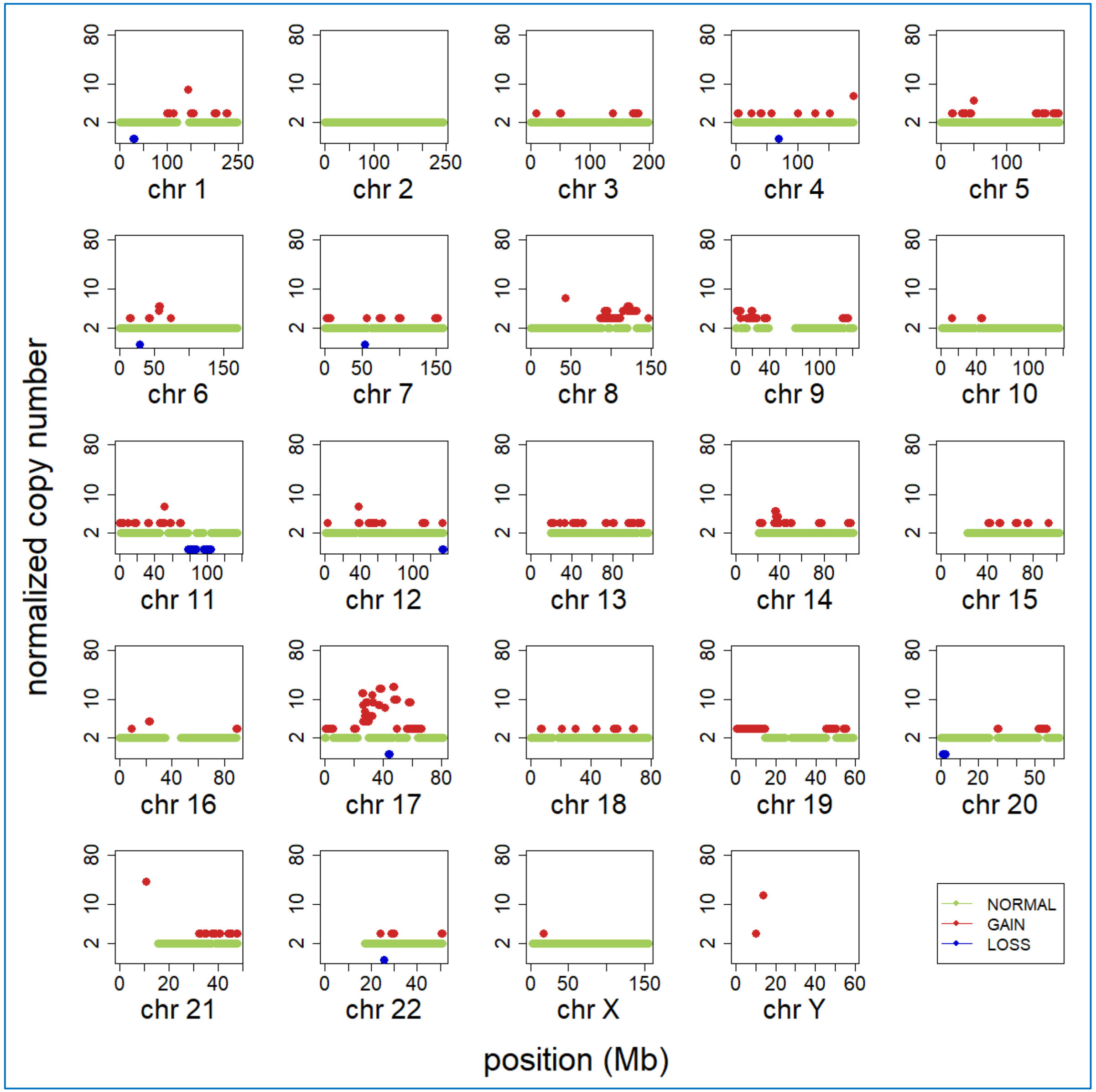

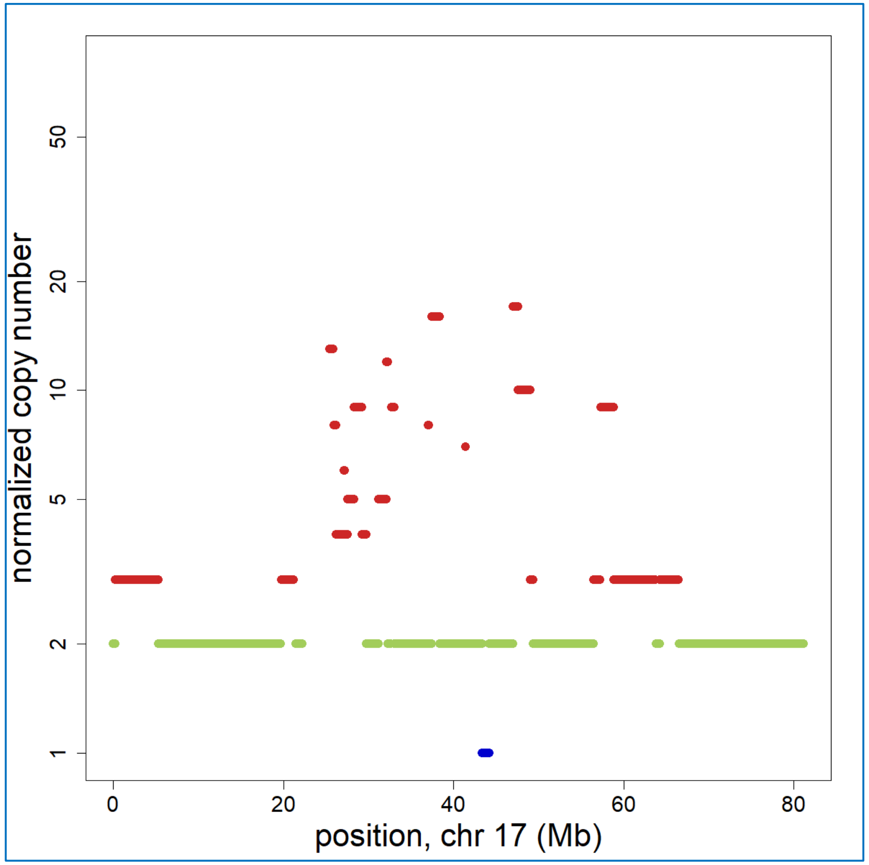

Control-FREEC

m-11

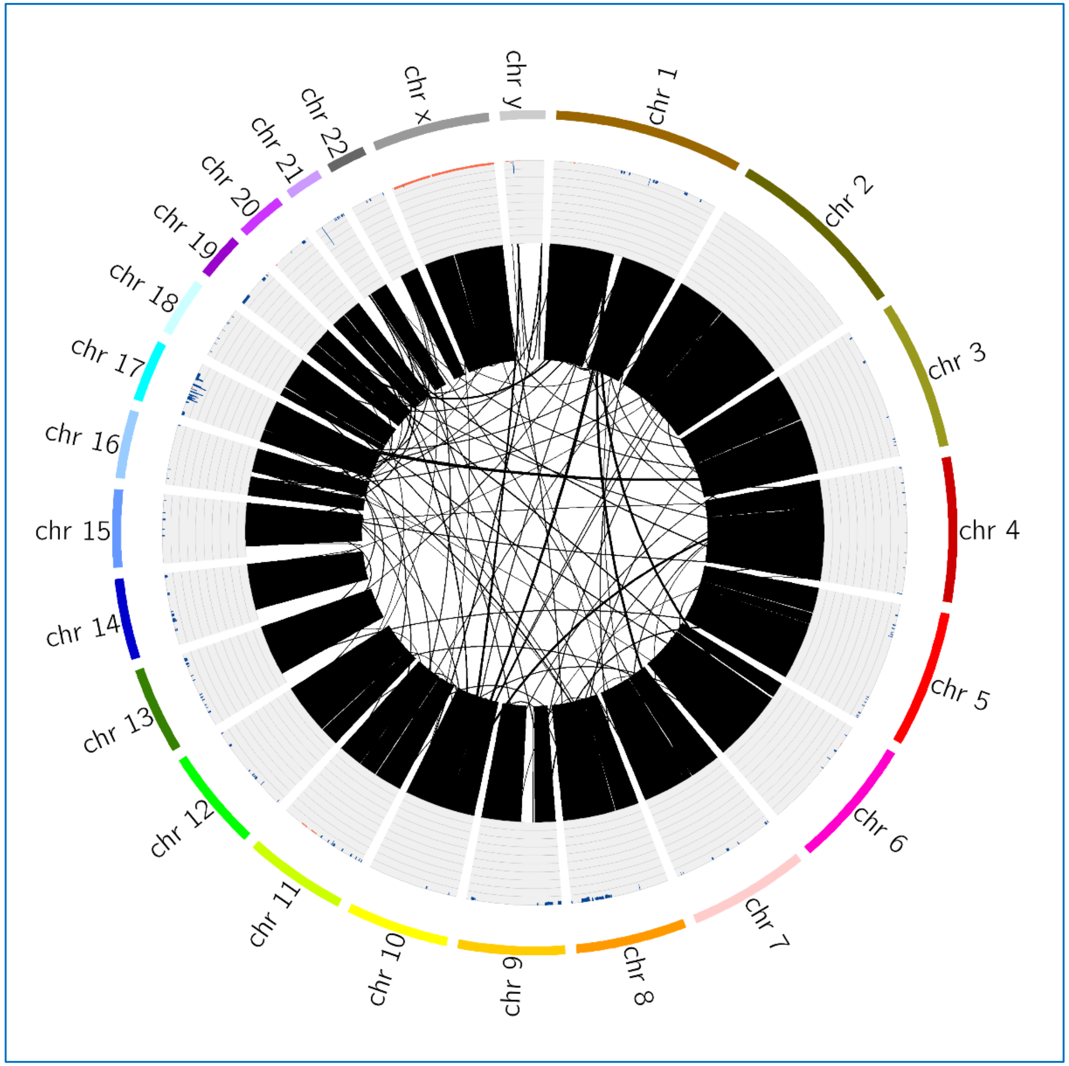

BreakDancer + Control-FREEC

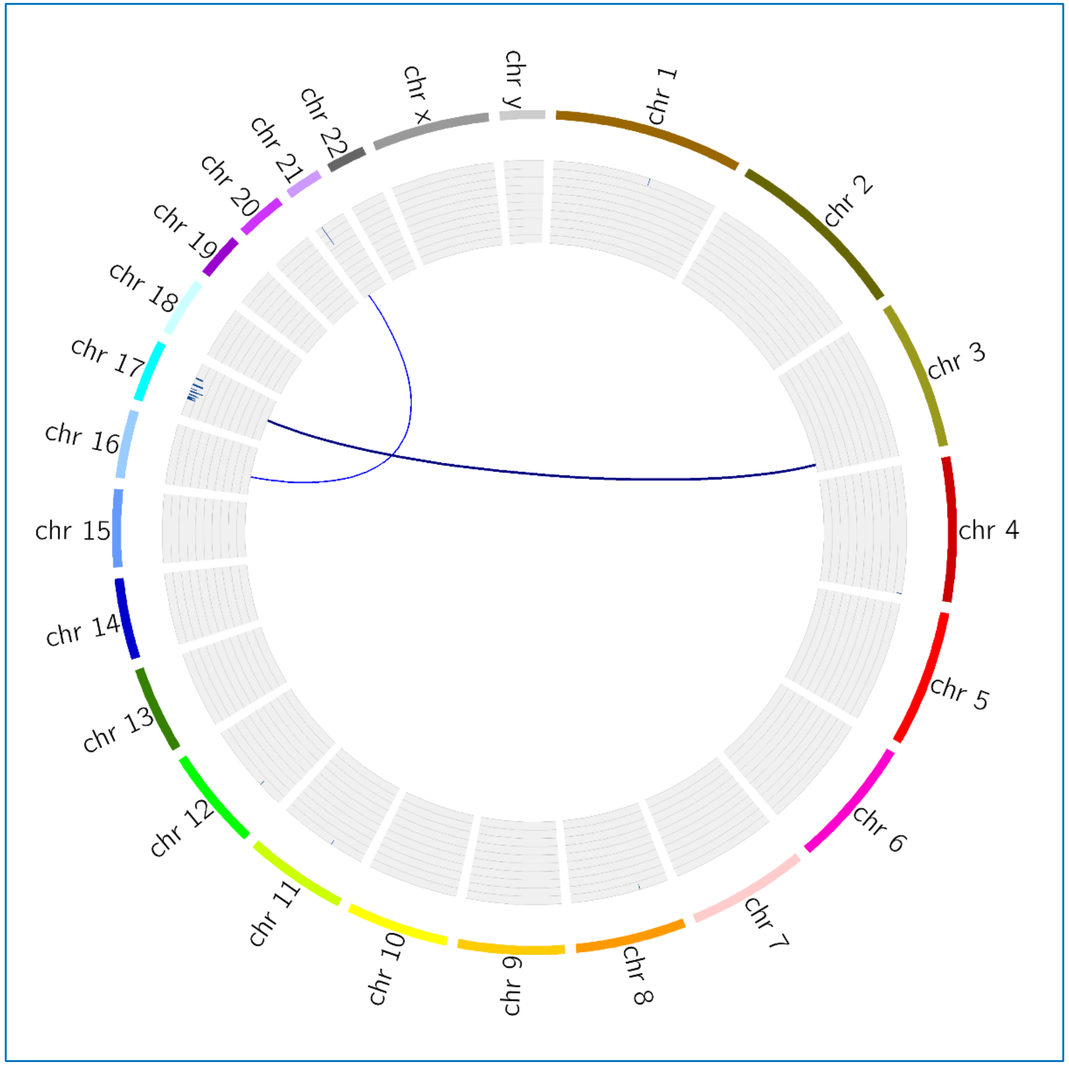

FAST – Whole Genome

m-11

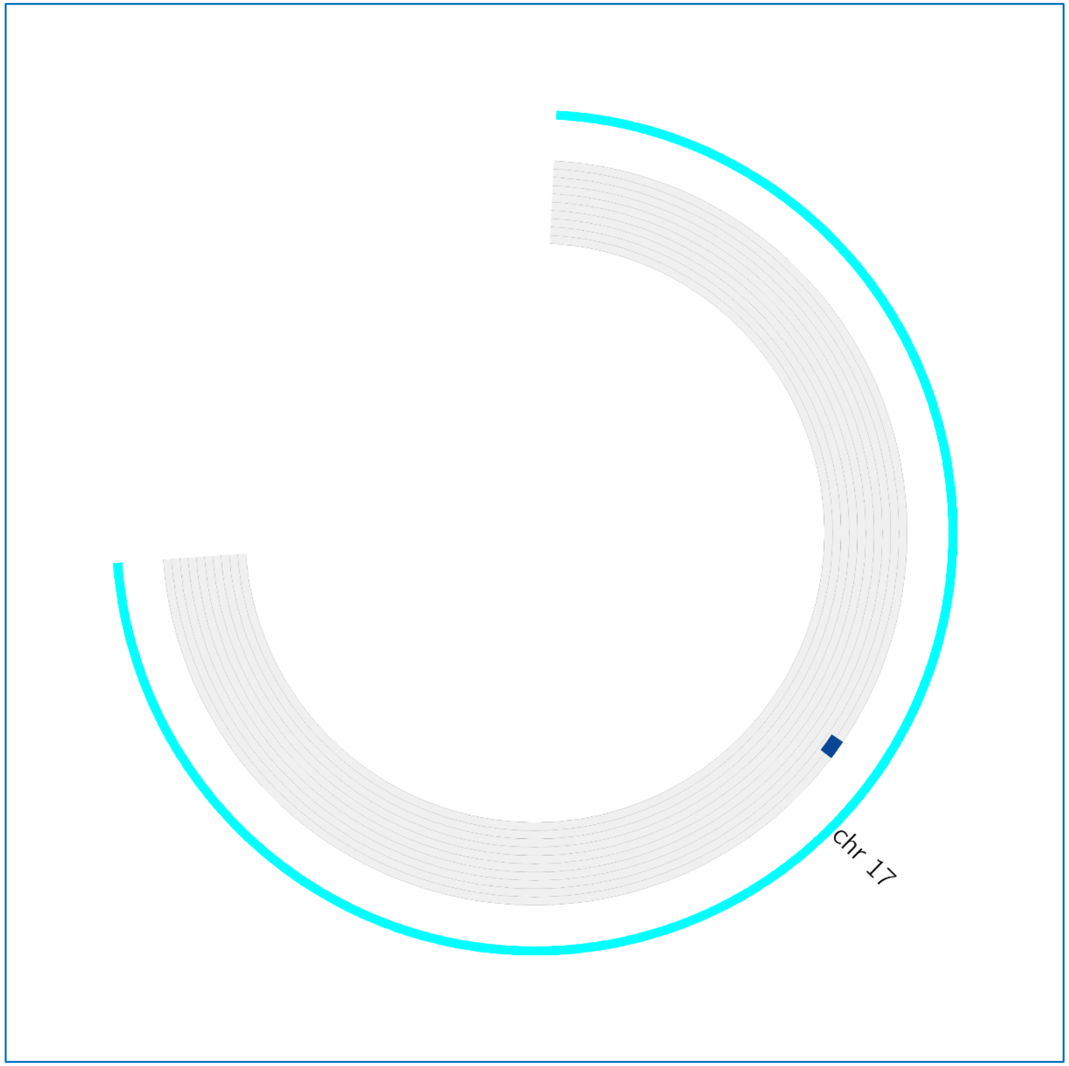

FAST – ERBB2 amplicon

m-12

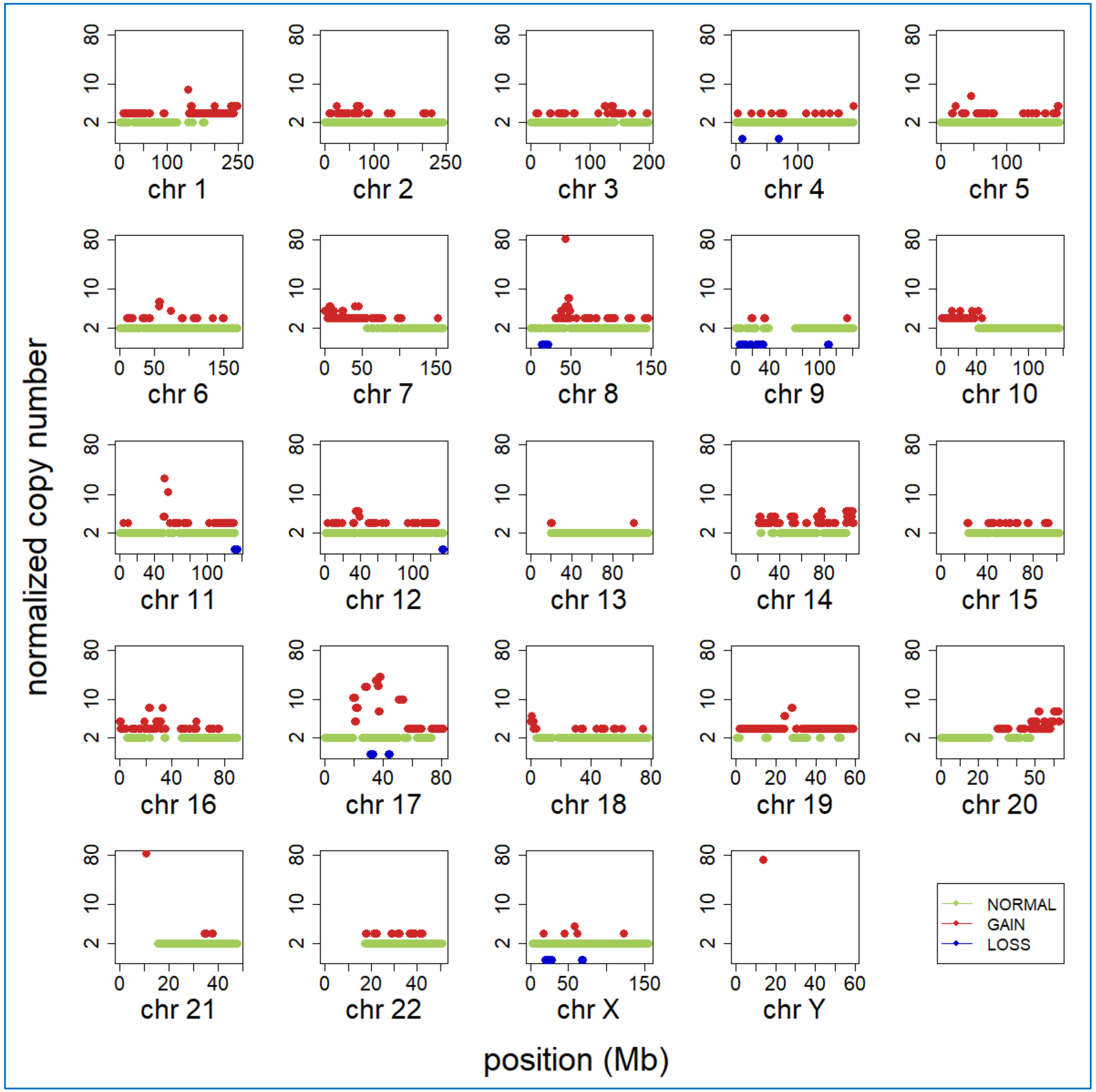

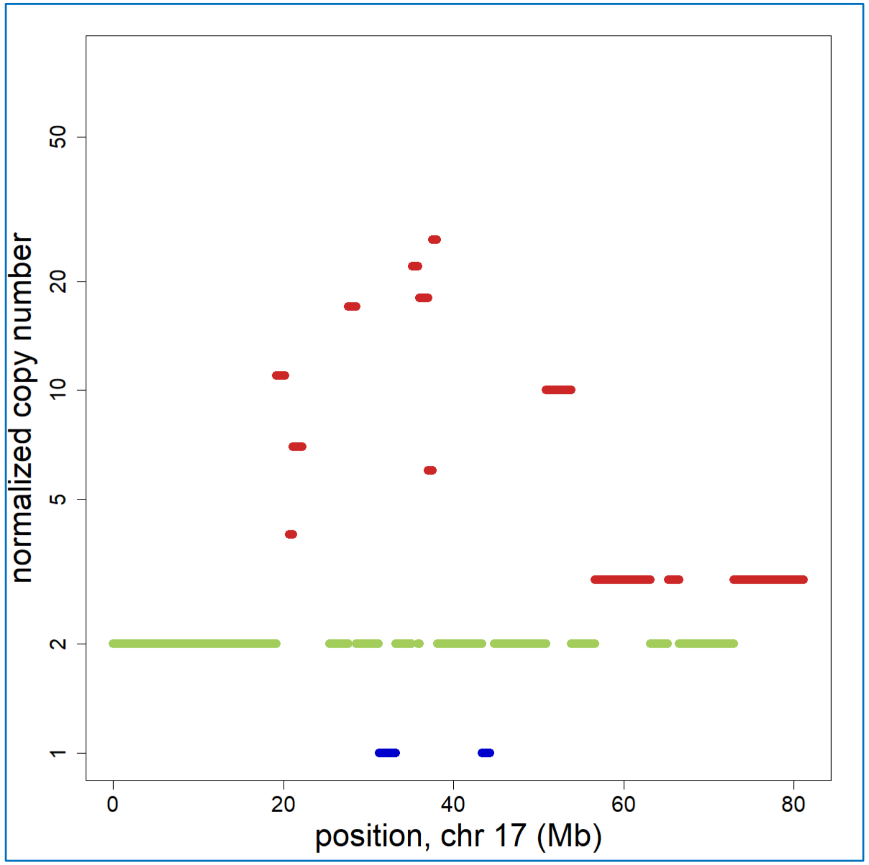

Control-FREEC

m-12

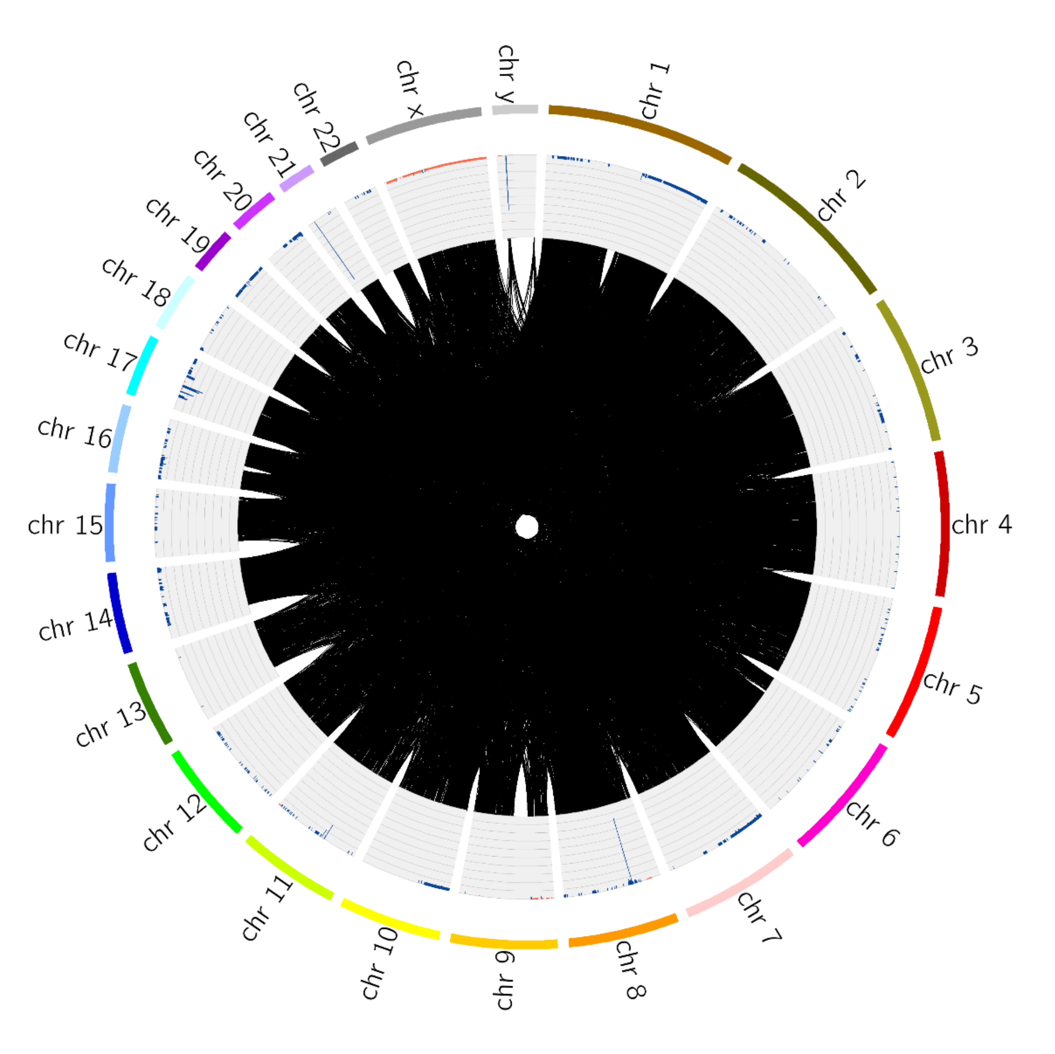

BreakDancer + Control-FREEC

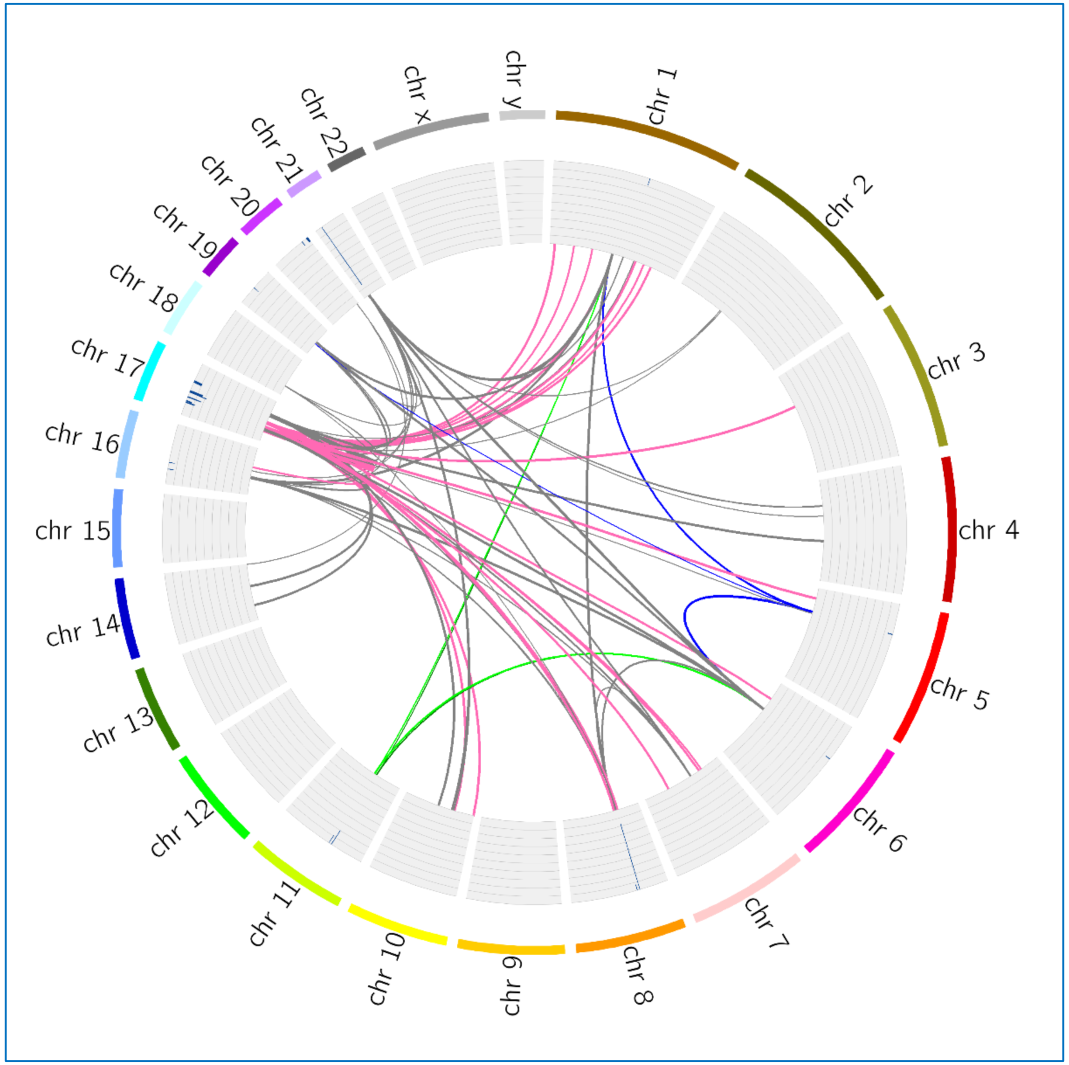

FAST – Whole Genome

m-12

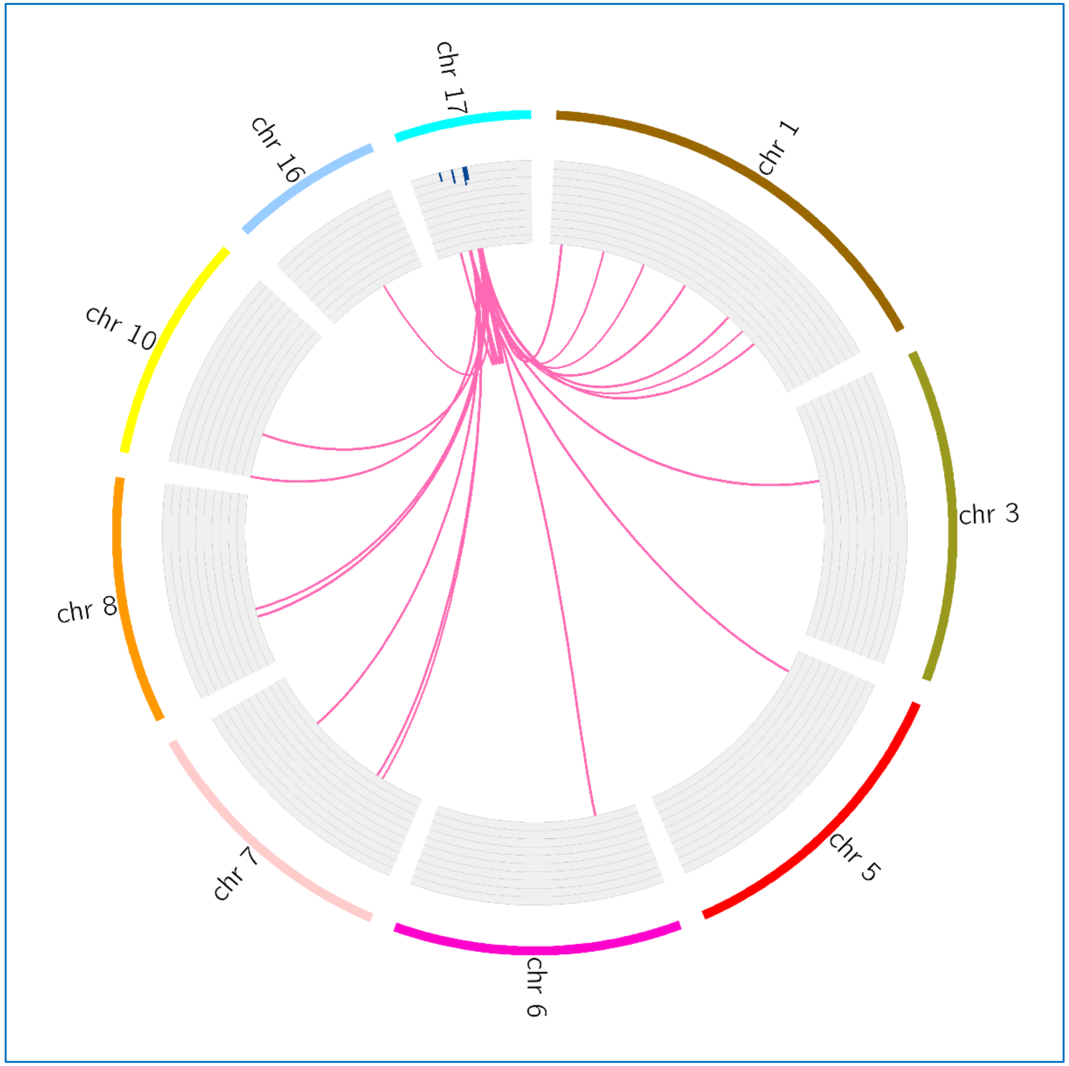

FAST – ERBB2 amplicon

m-13

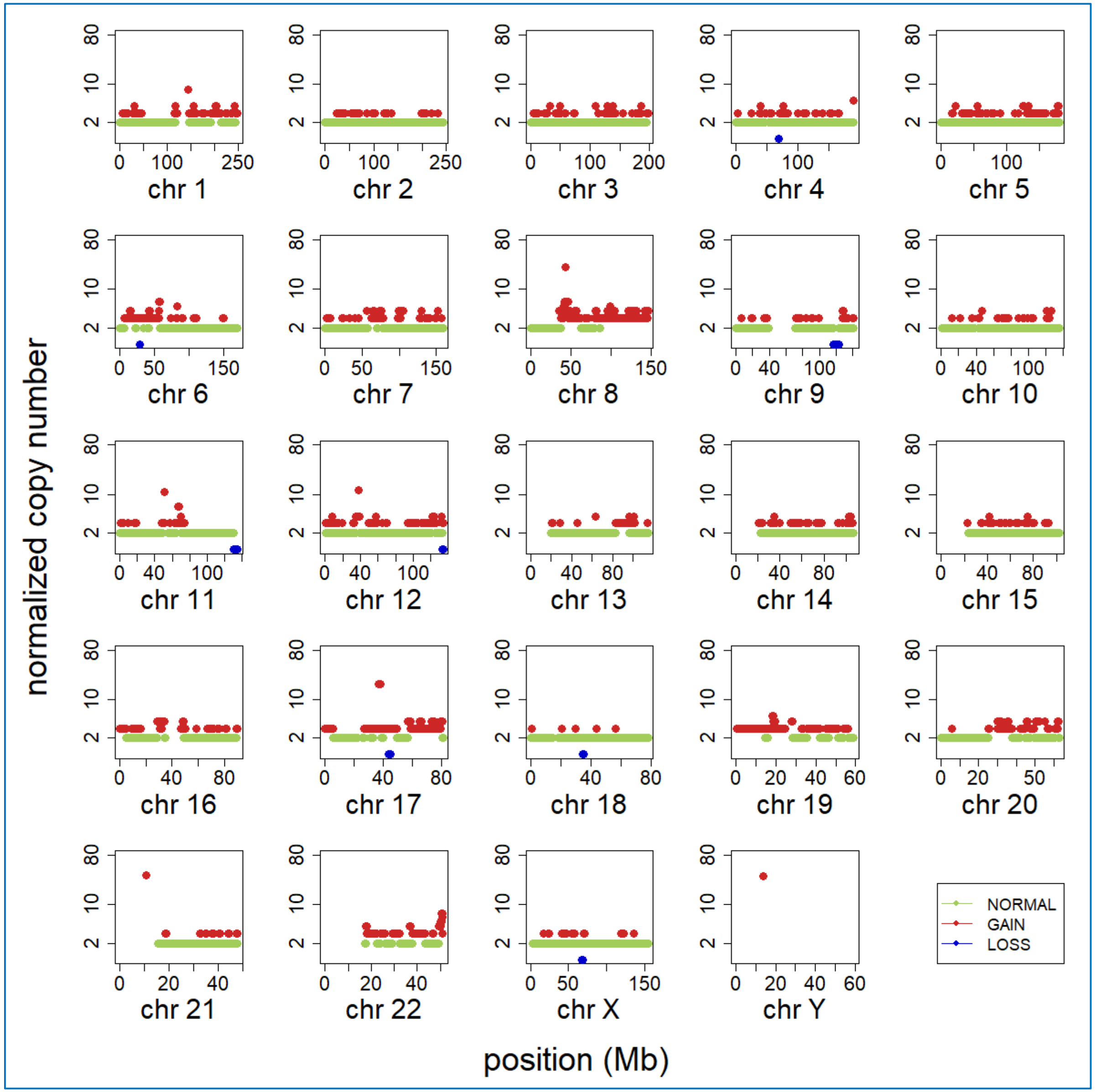

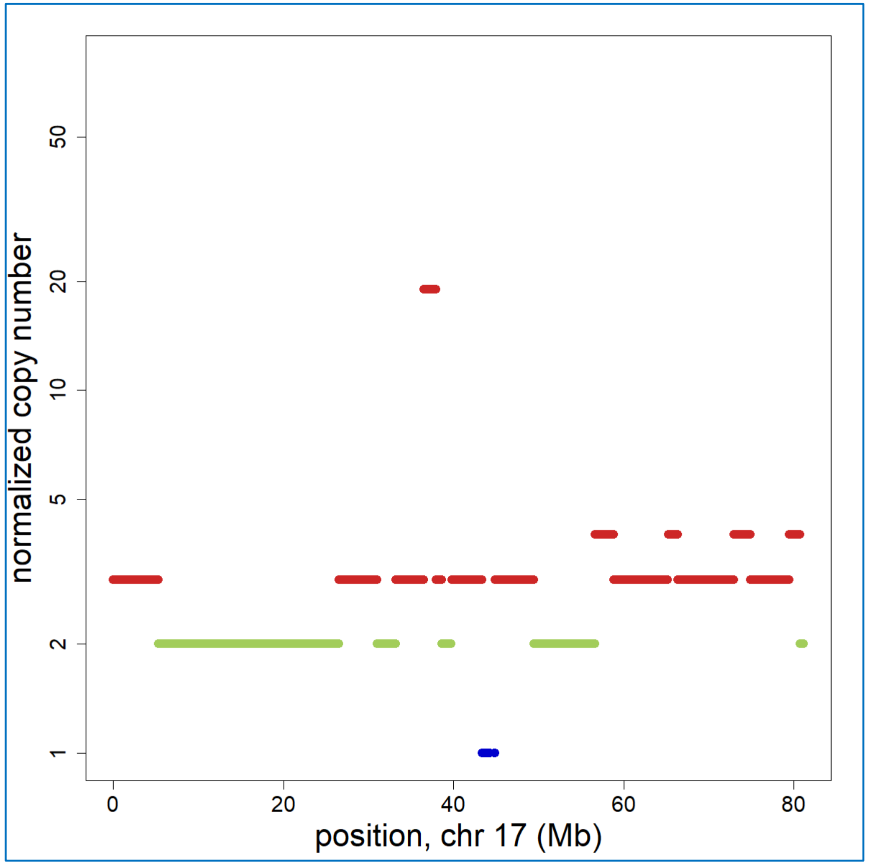

Control-FREEC

m-13

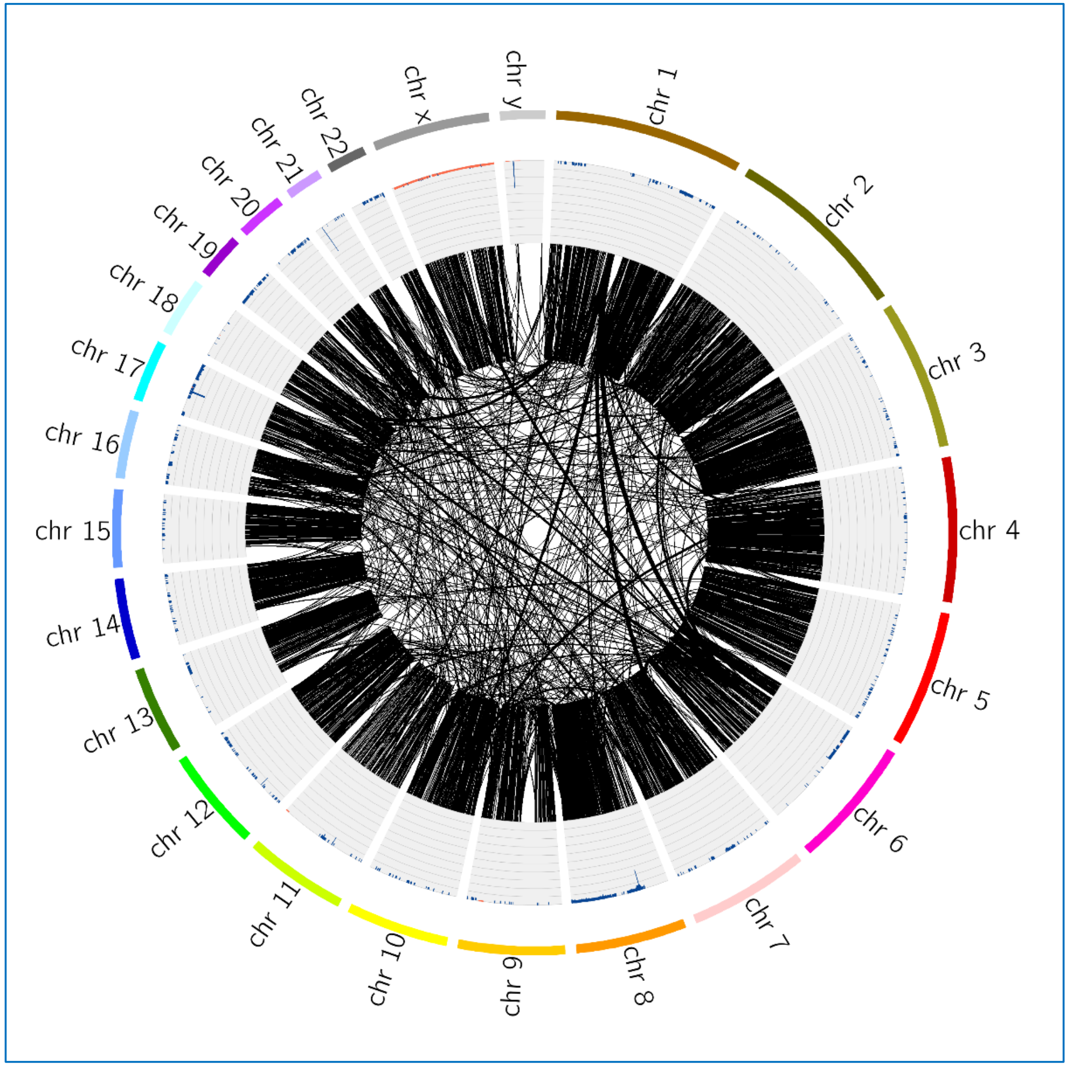

BreakDancer + Control-FREEC

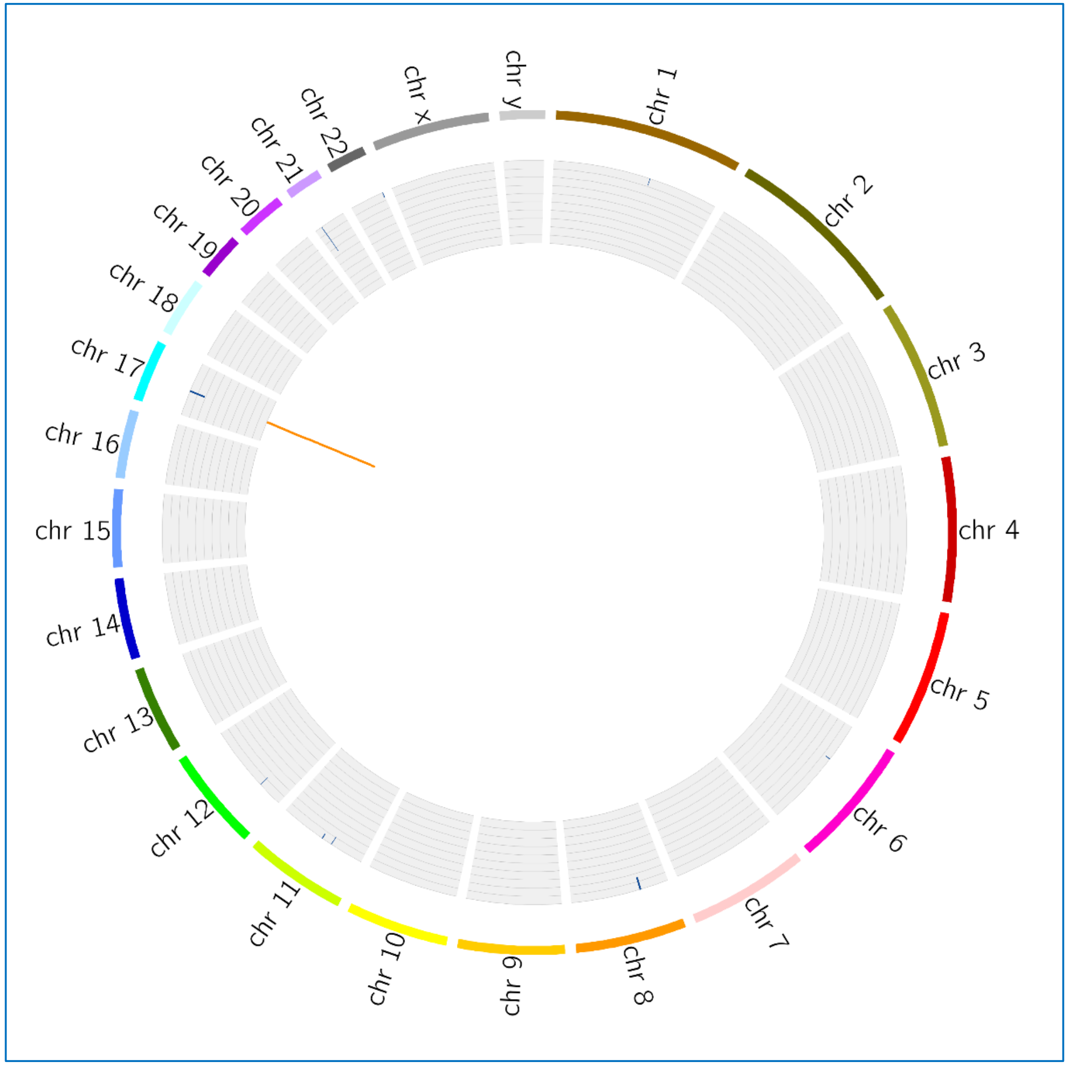

FAST – Whole Genome

m-13

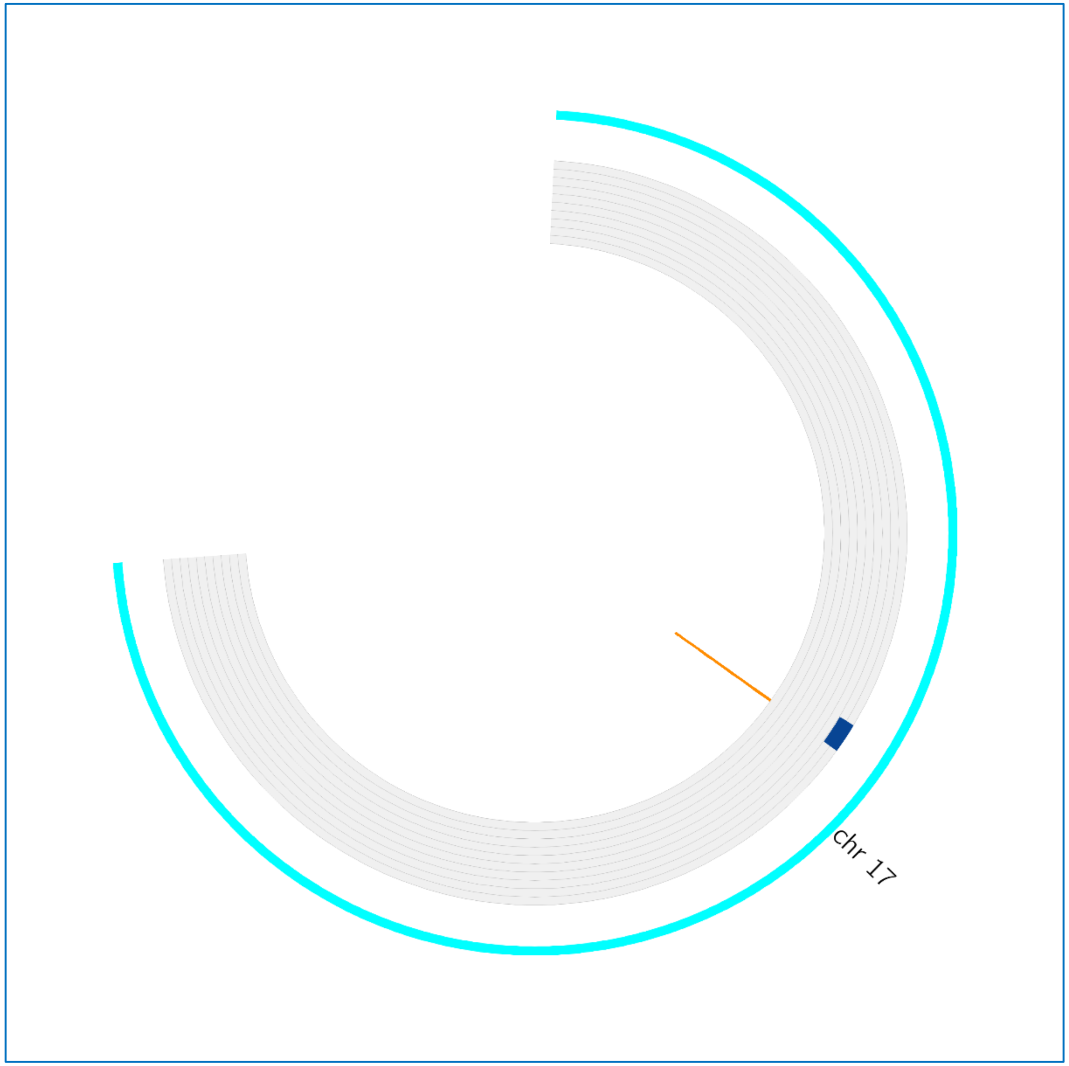

FAST – ERBB2 amplicon

p-16

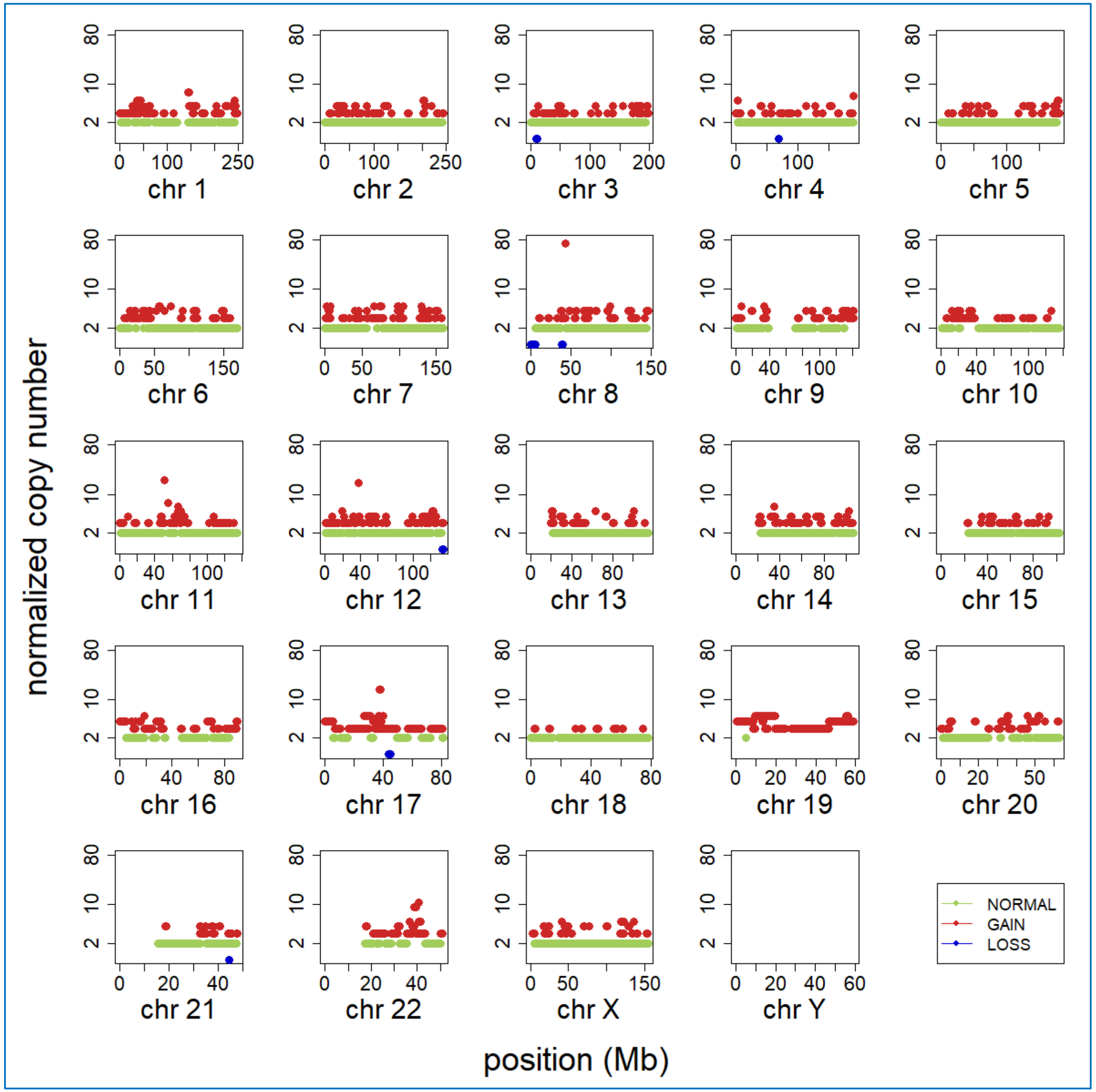

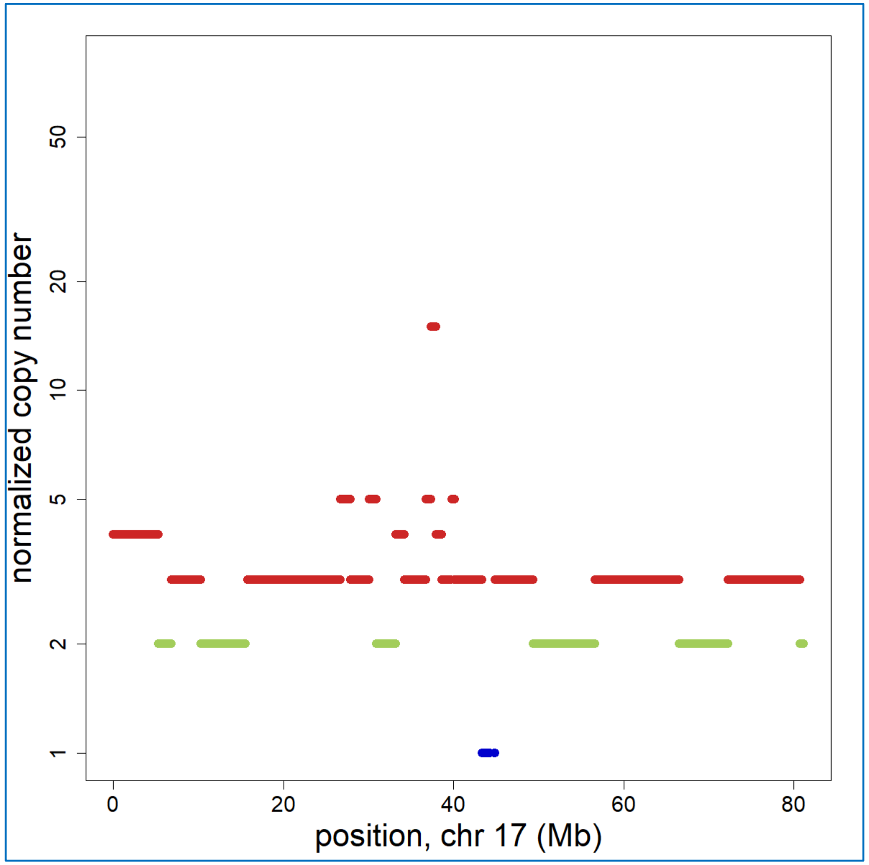

Control-FREEC

p-16

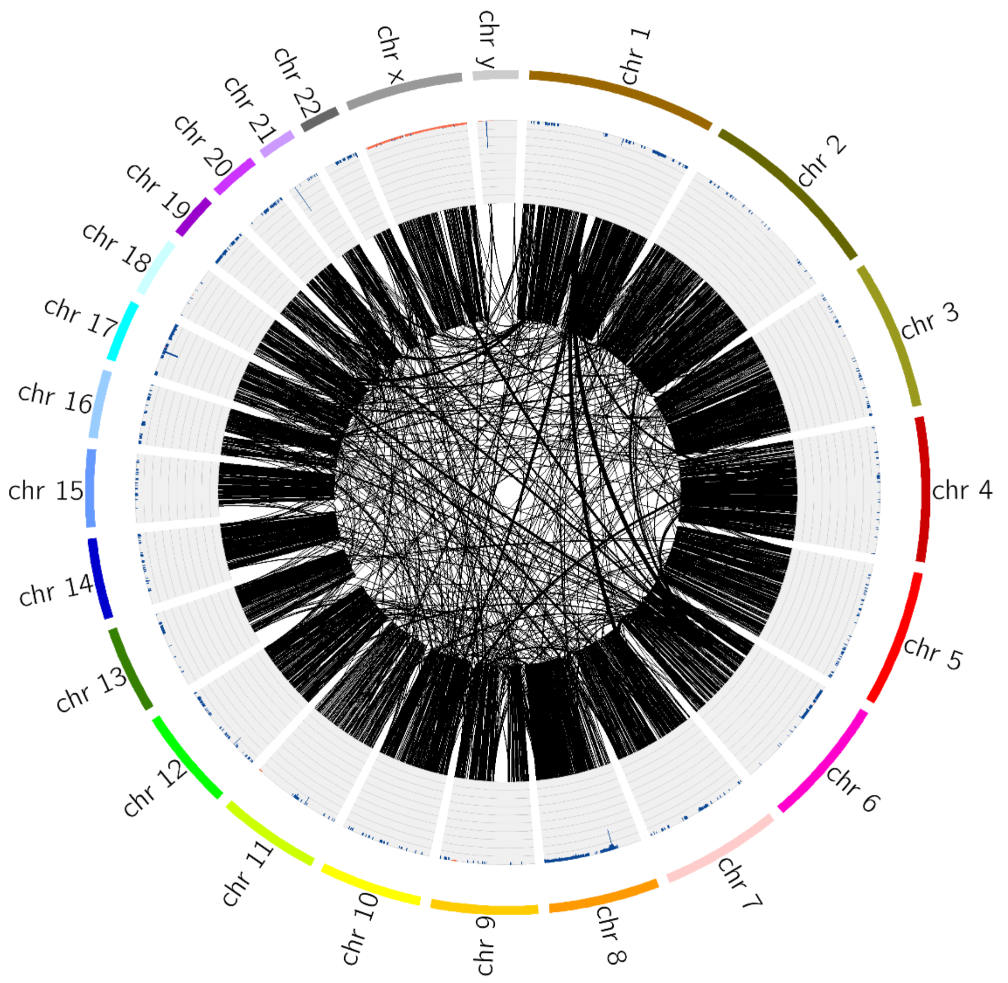

BreakDancer + Control-FREEC

No FAST data for this sample

FAST – Whole Genome

m-16

No FAST data for this sample

FAST – ERBB2 amplicon

p-17

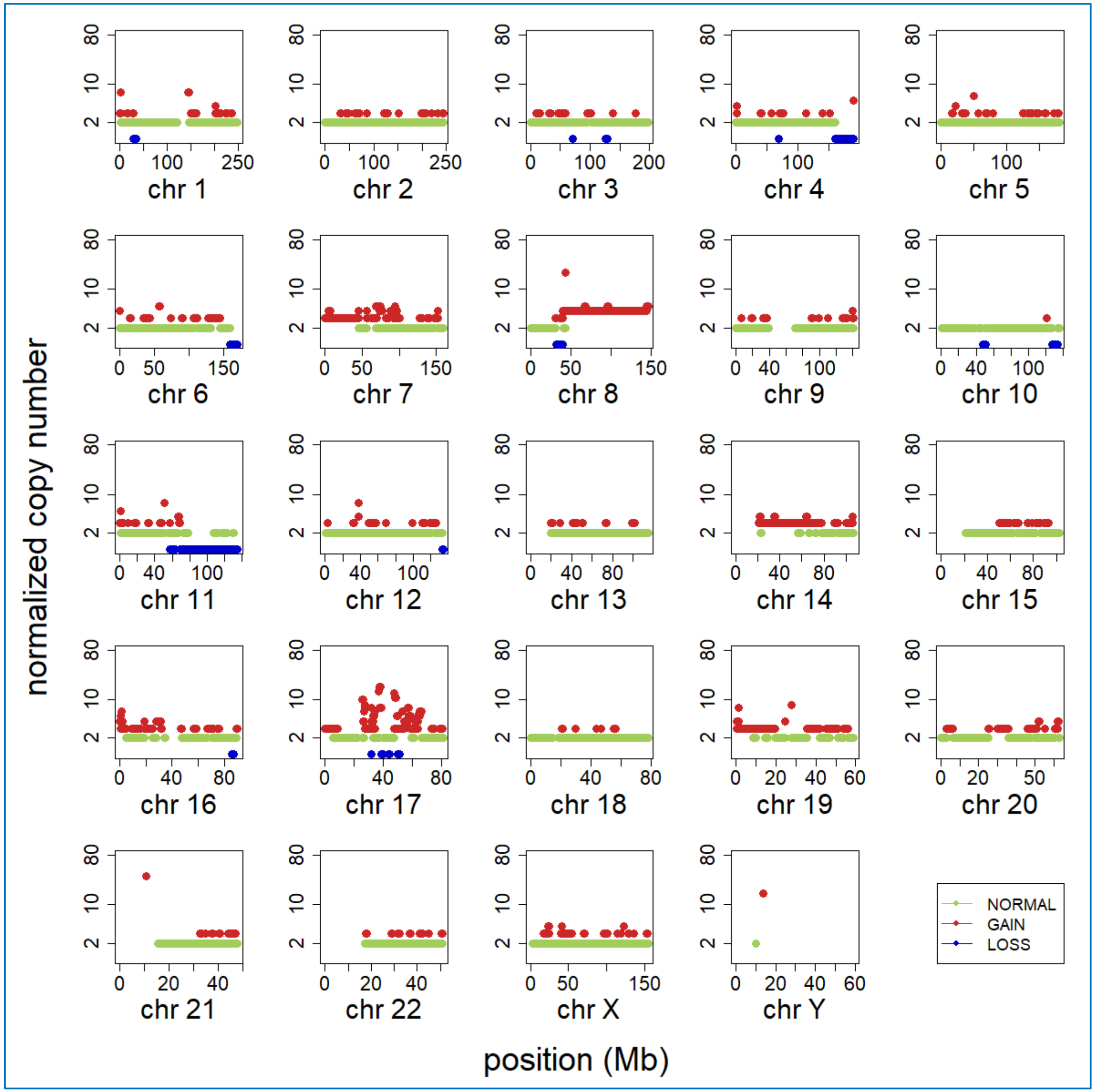

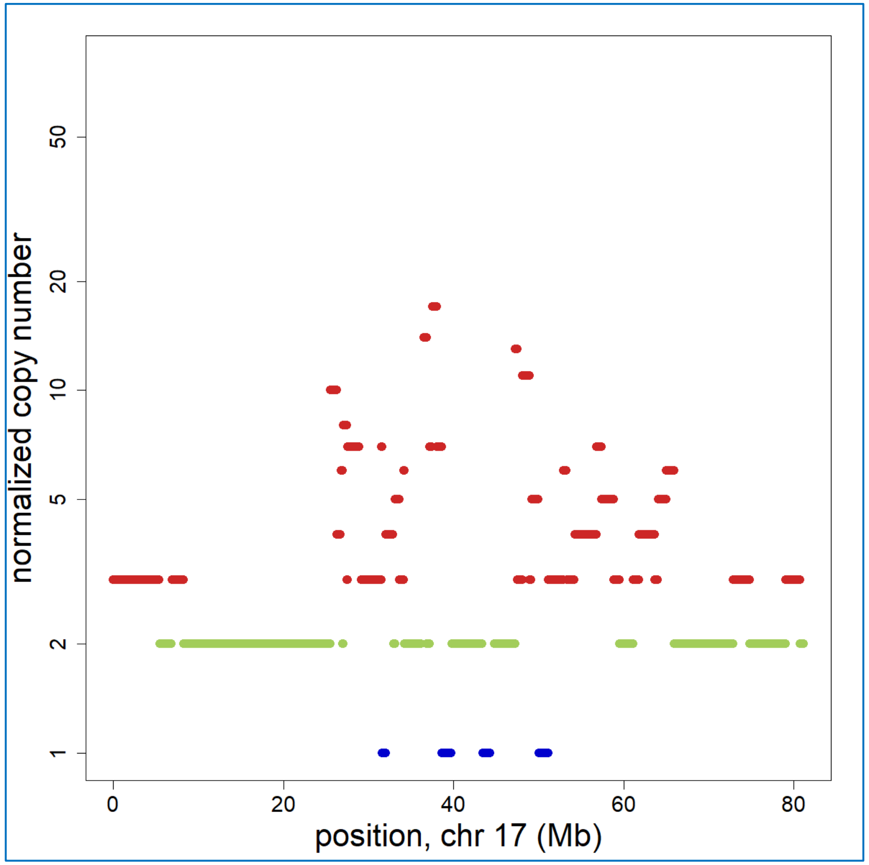

Control-FREEC

p-17

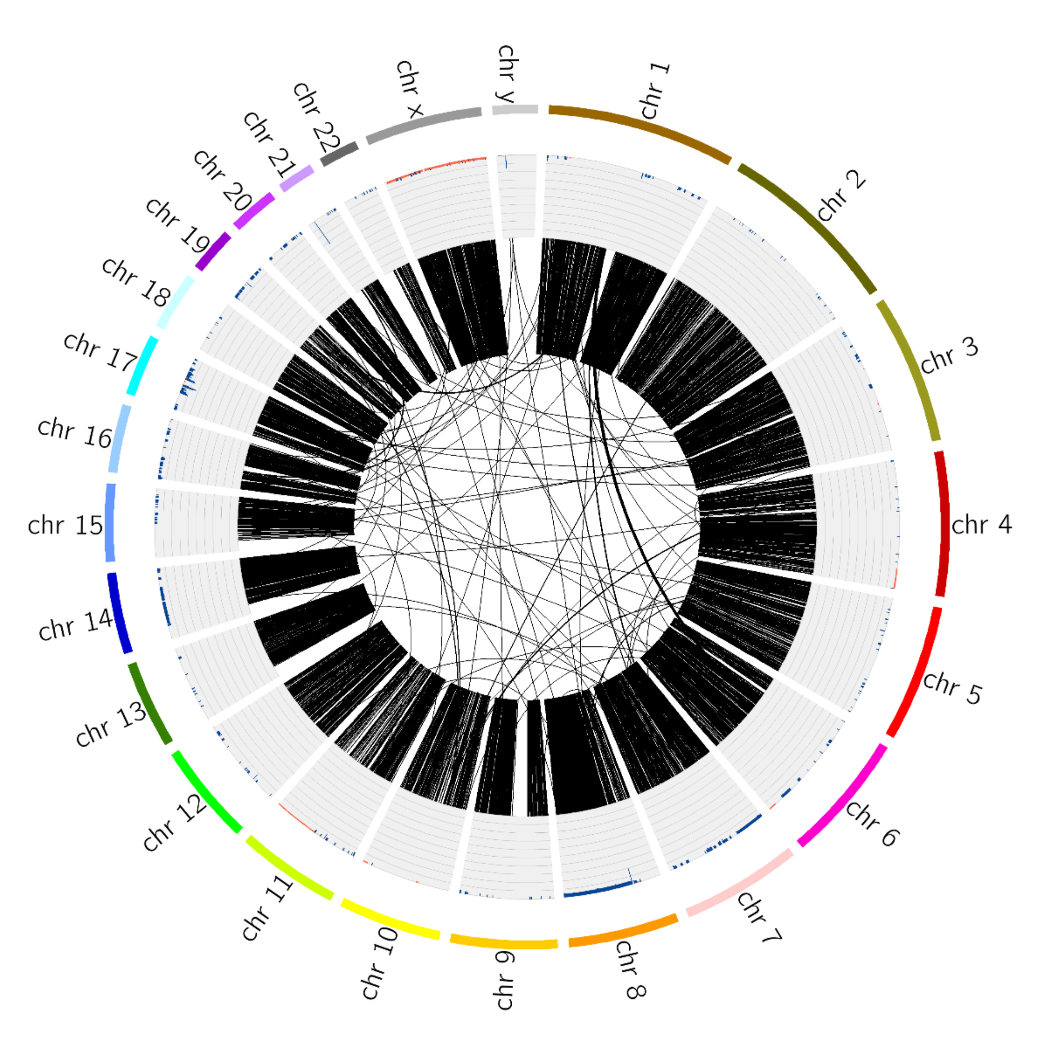

BreakDancer + Control-FREEC

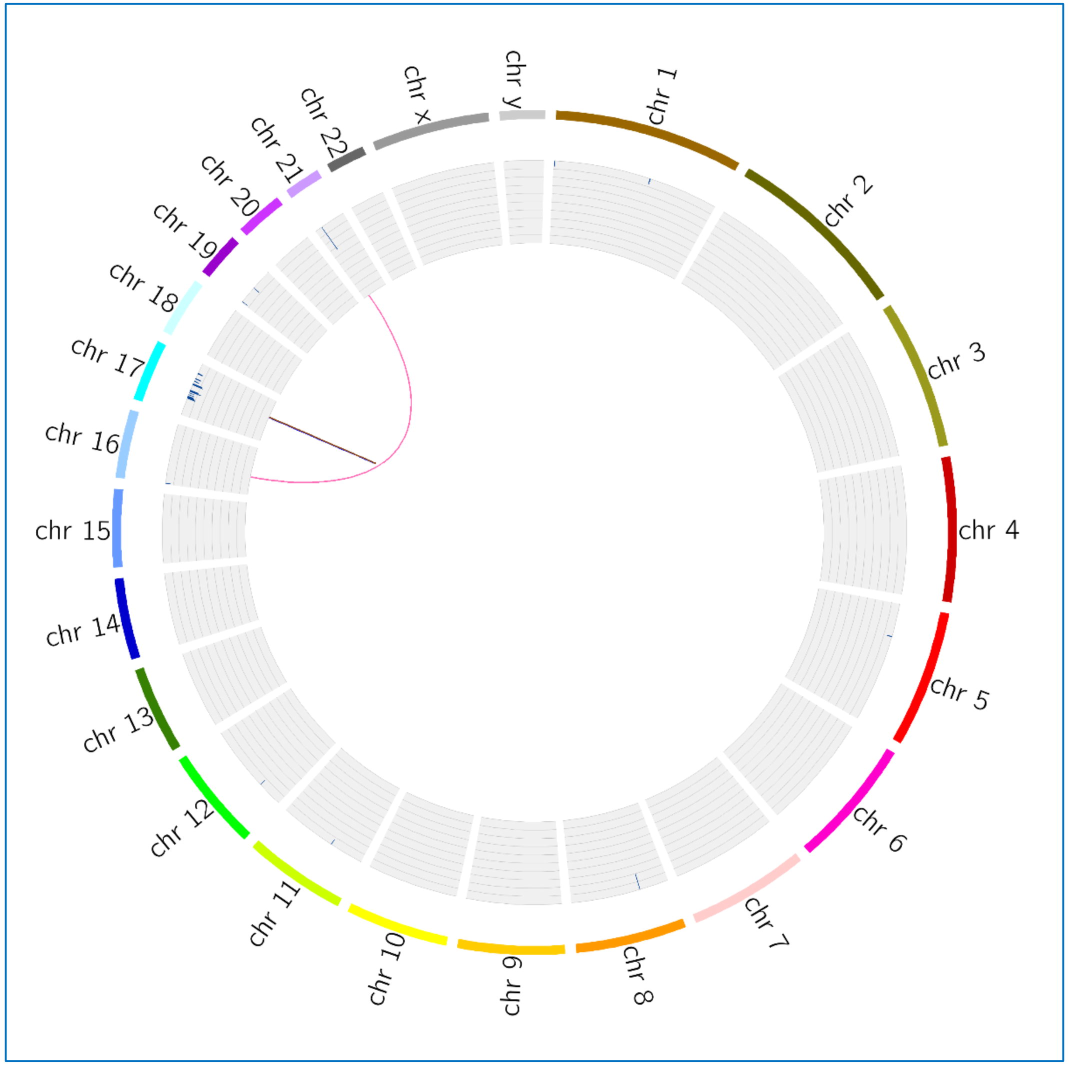

FAST – Whole Genome

p-17

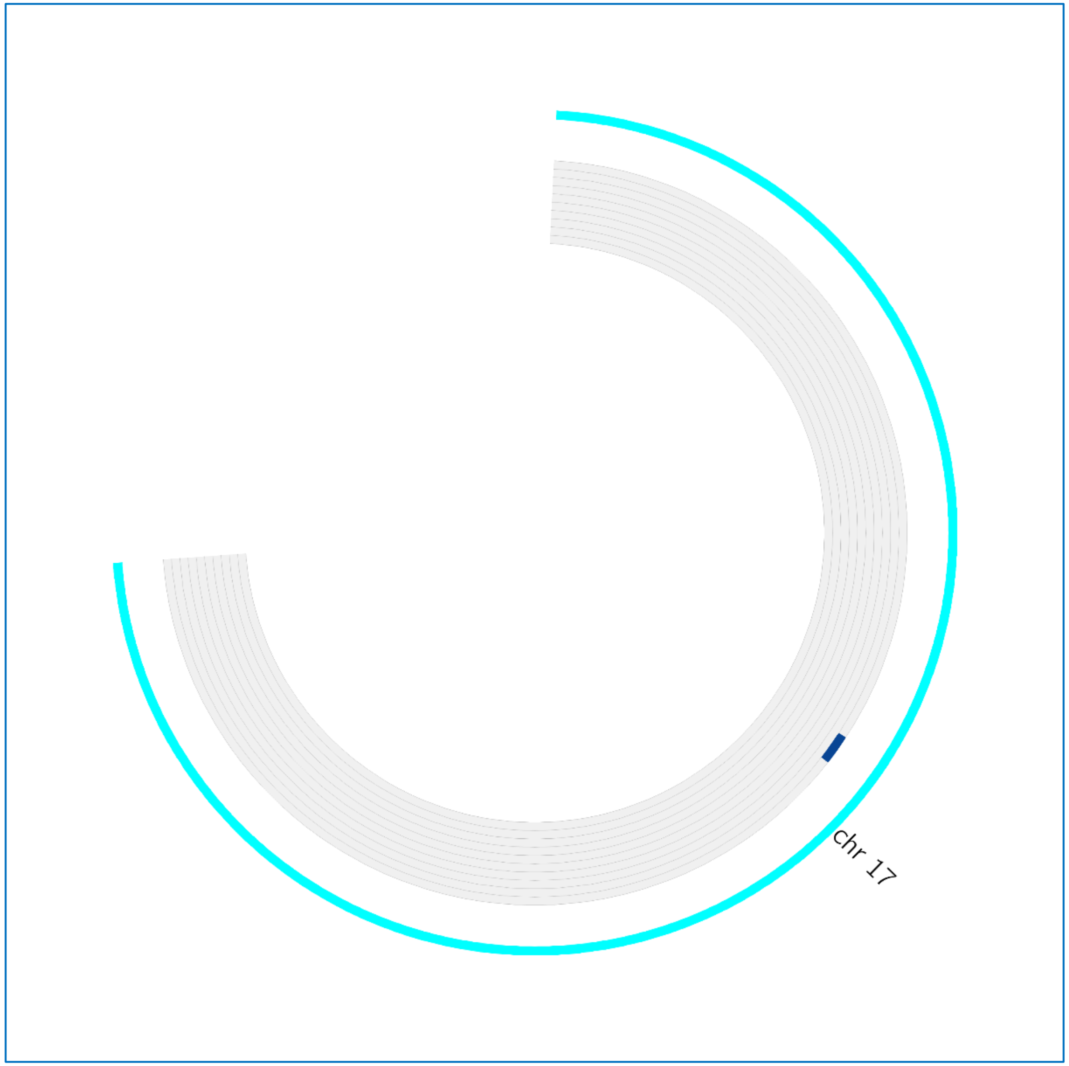

FAST – ERBB2 amplicon

p-20

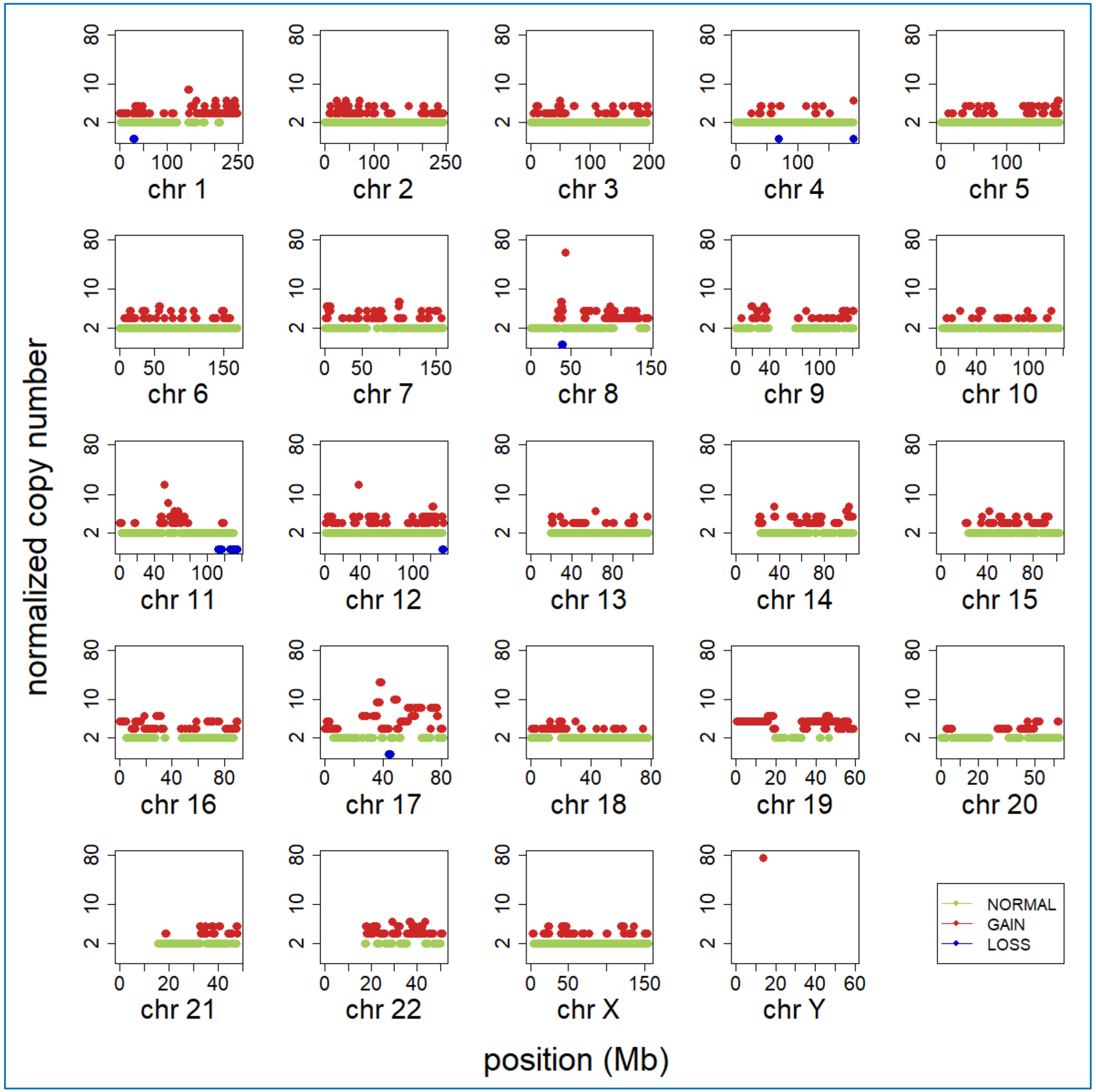

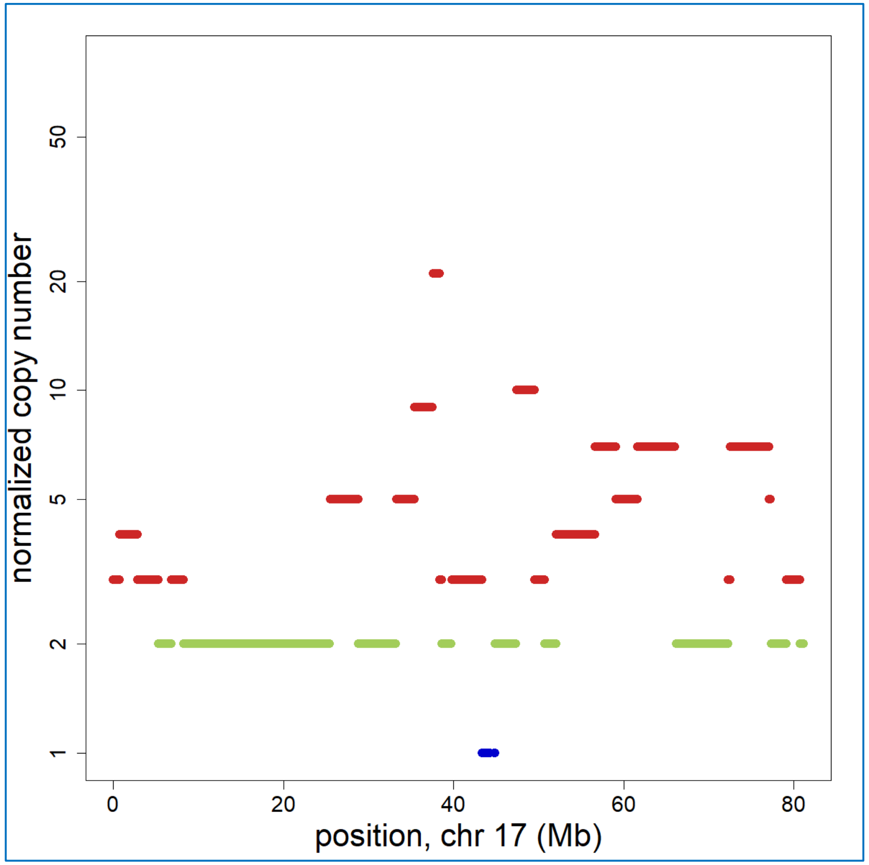

Control-FREEC

p-20

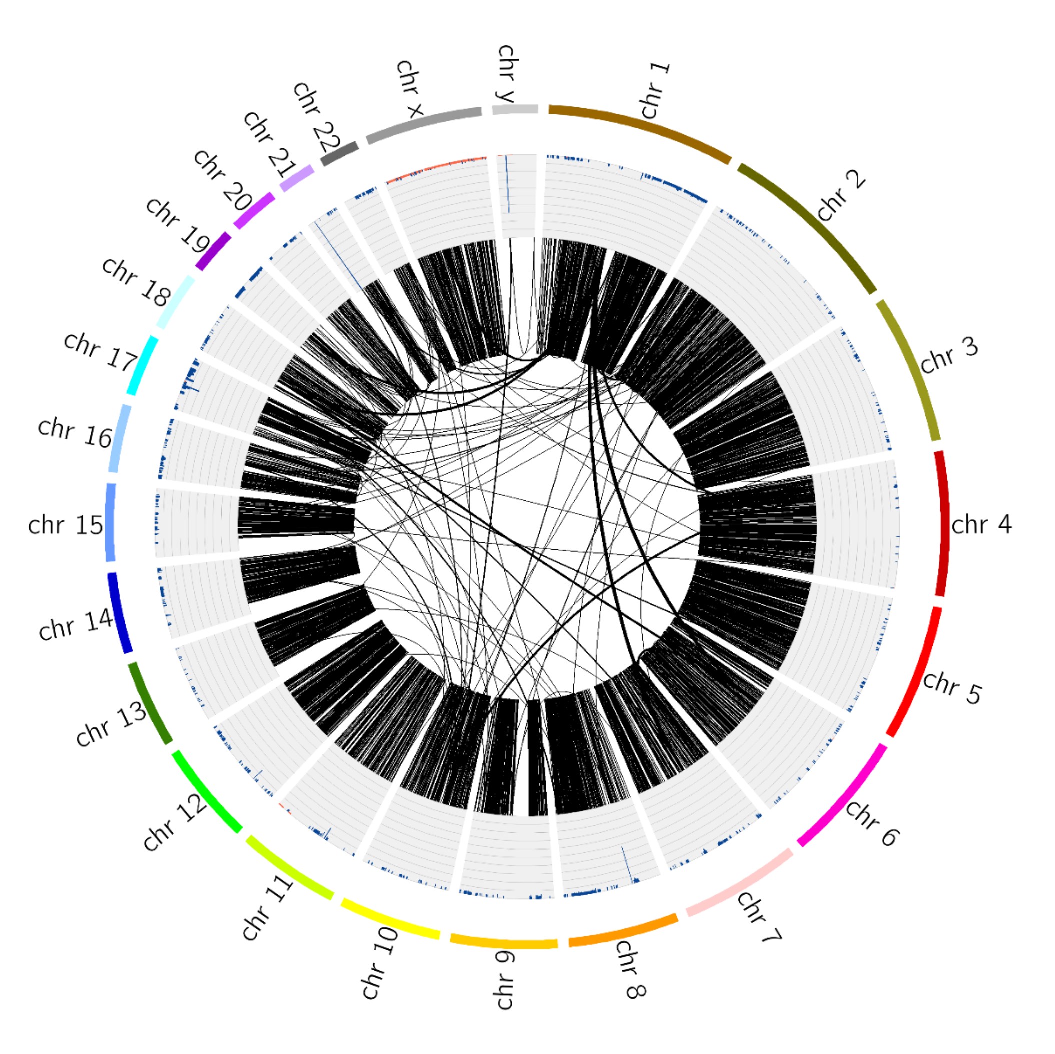

BreakDancer + Control-FREEC

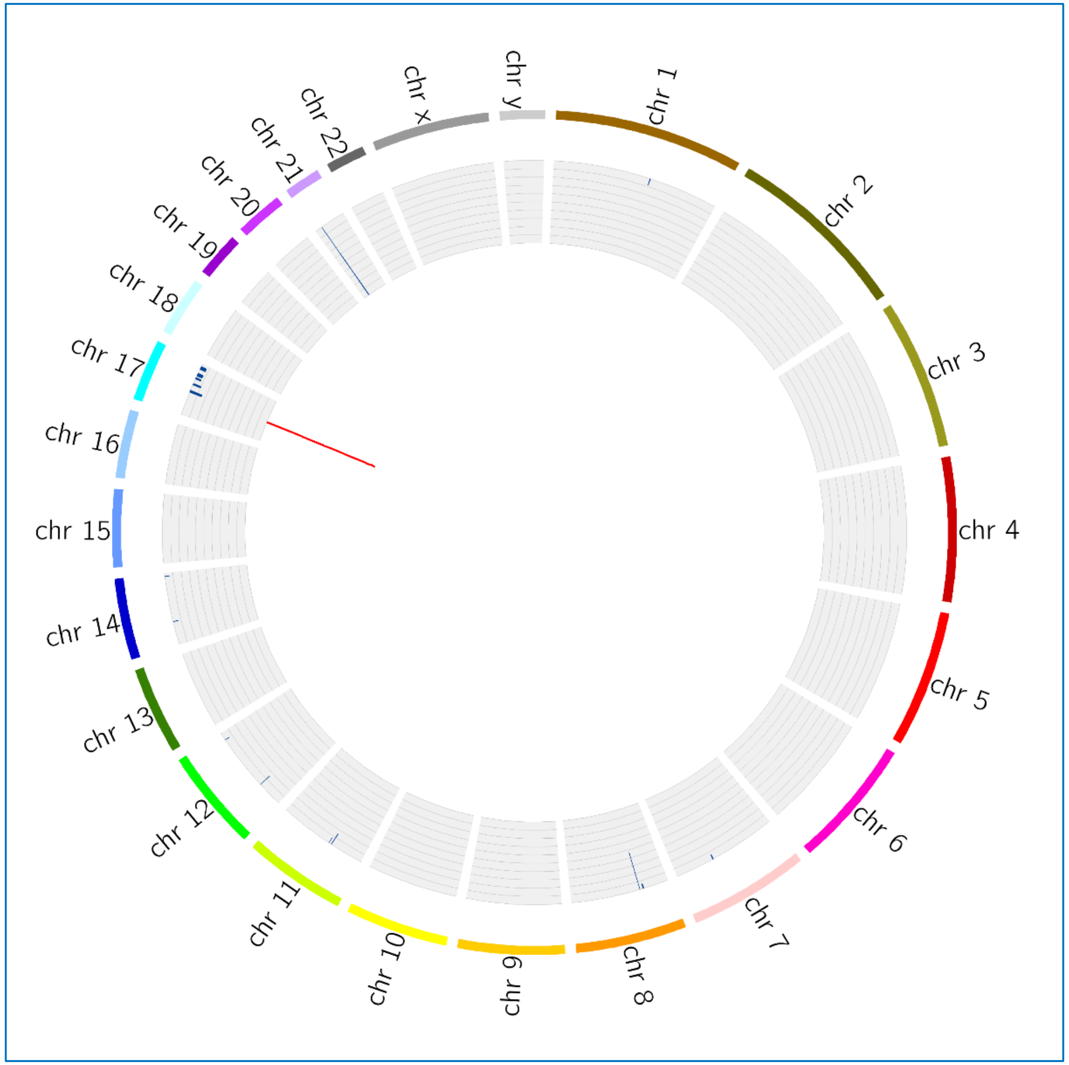

FAST – Whole Genome

p-20

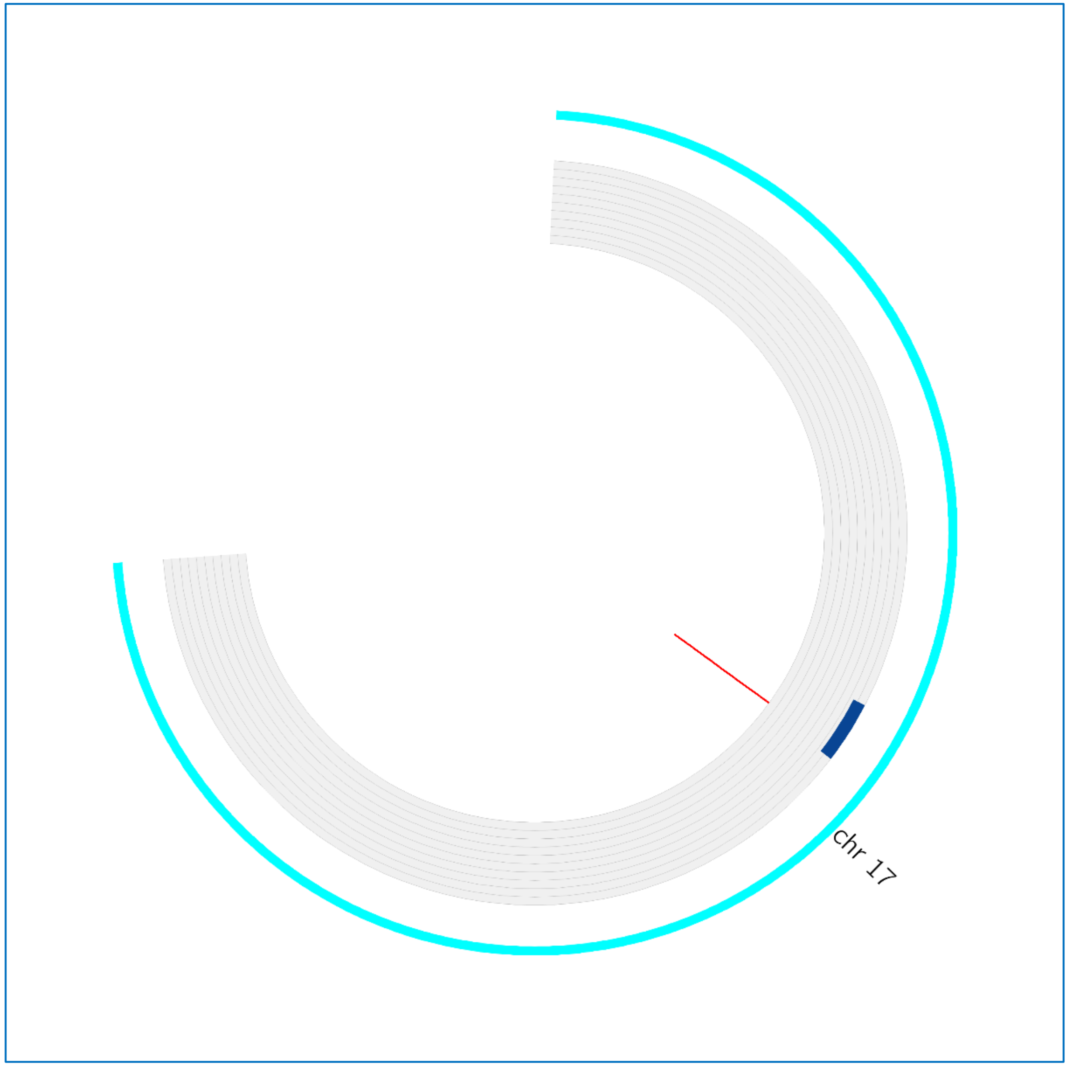

FAST – ERBB2 amplicon

p-21

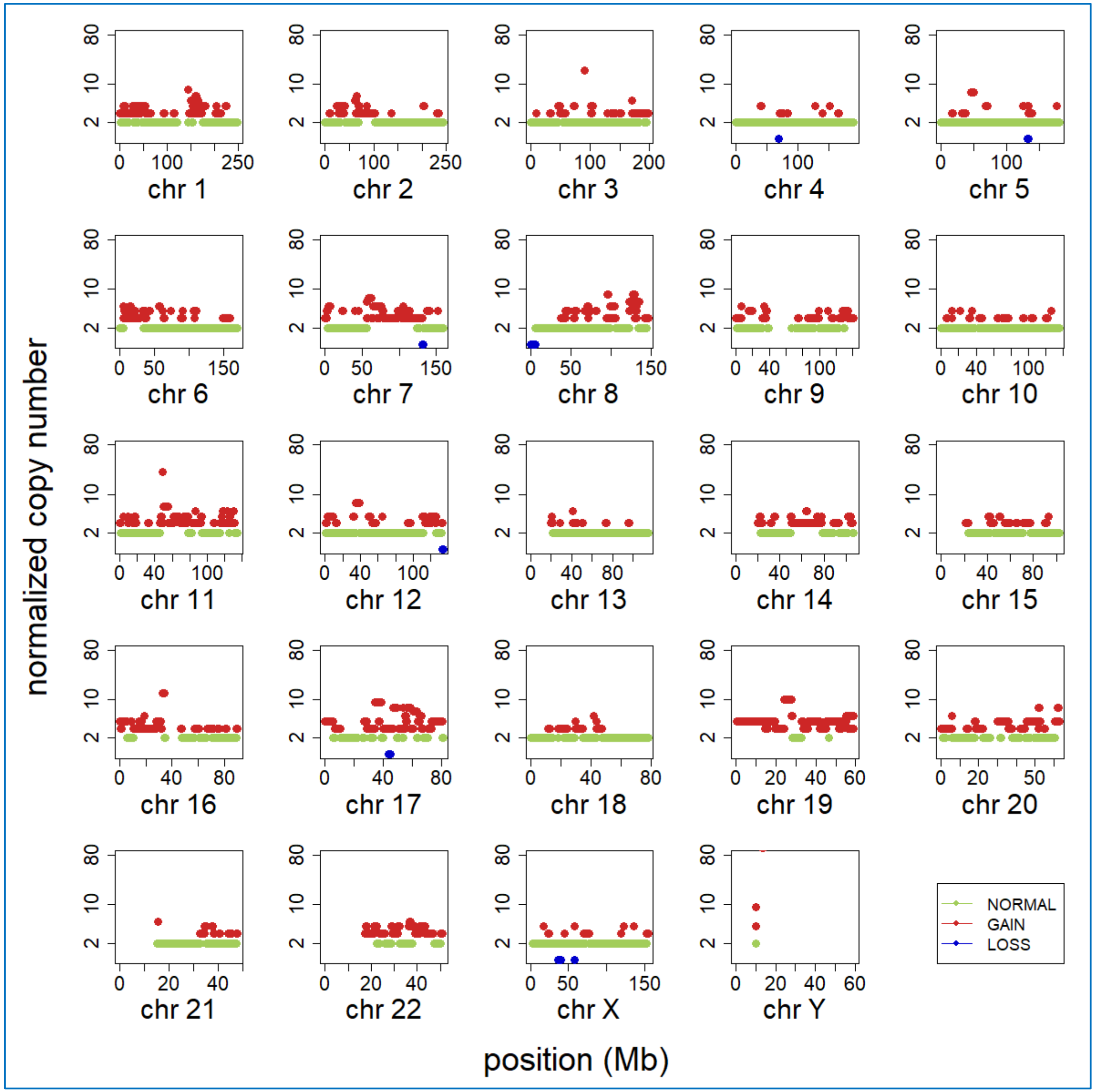

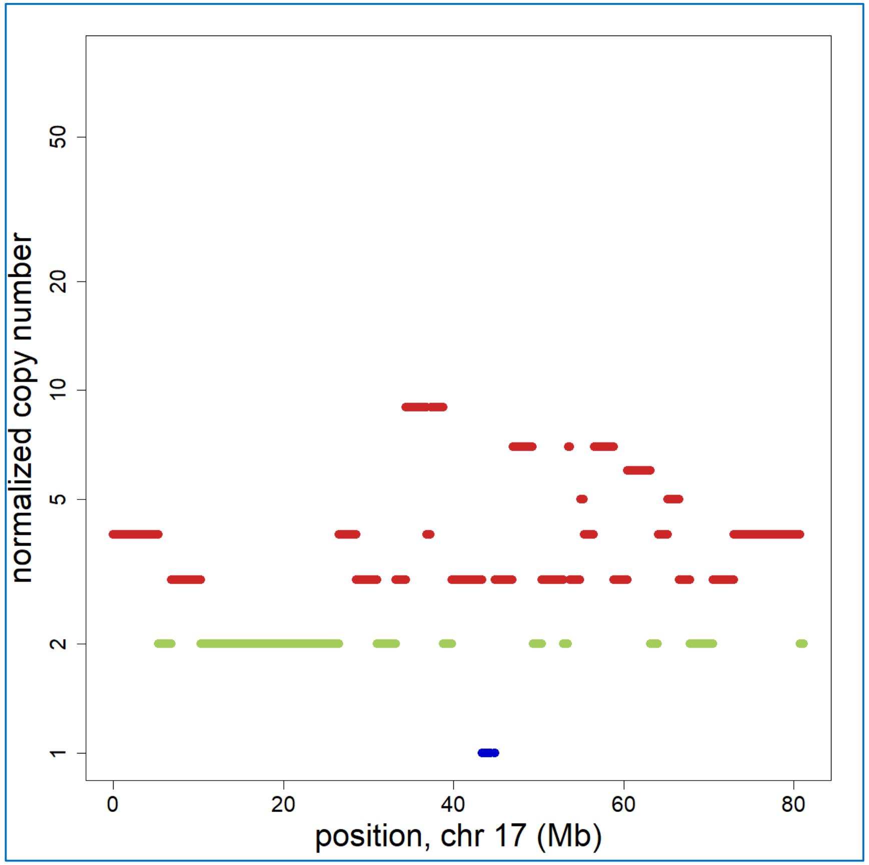

Control-FREEC

p-21

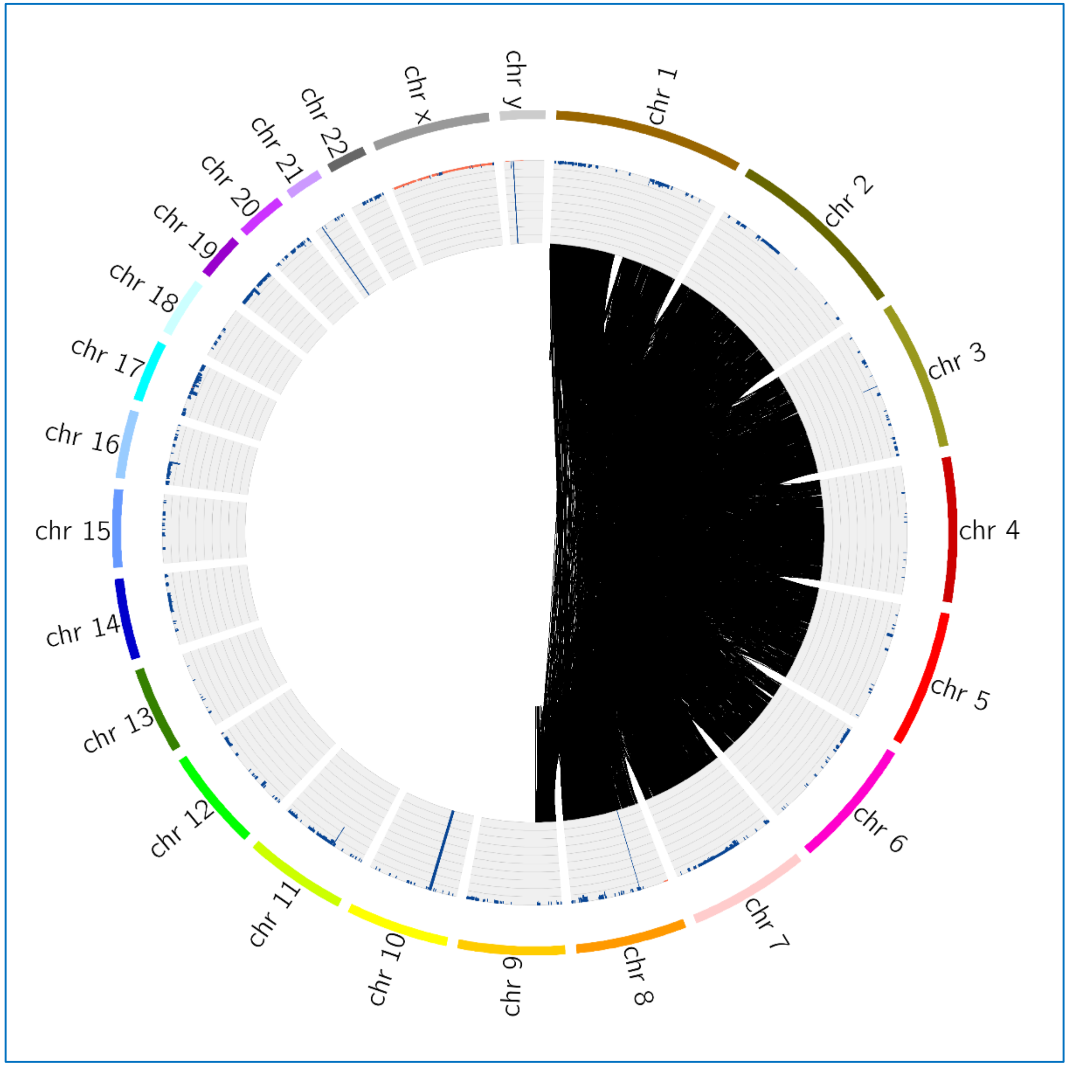

BreakDancer + Control-FREEC

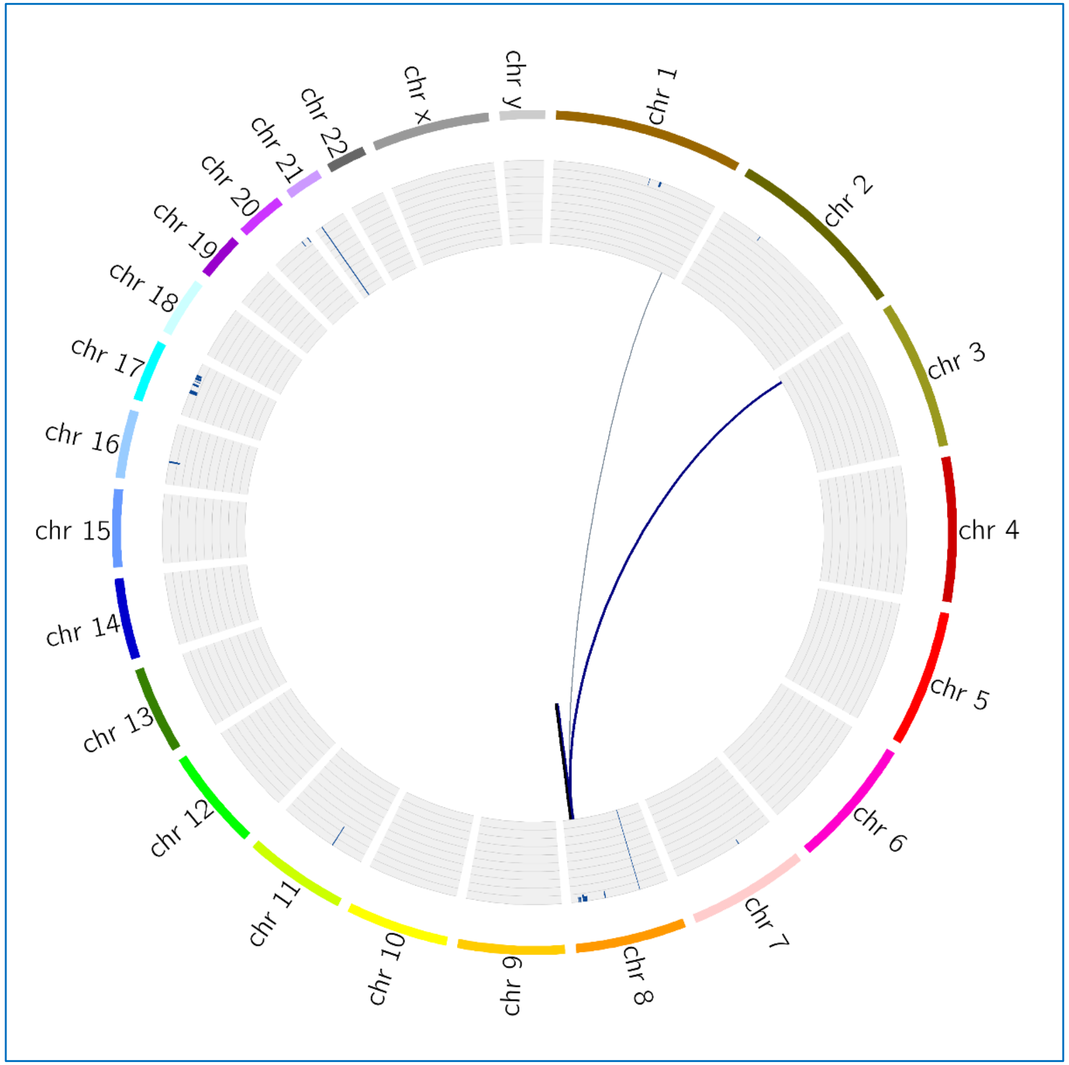

FAST – Whole Genome

p-21

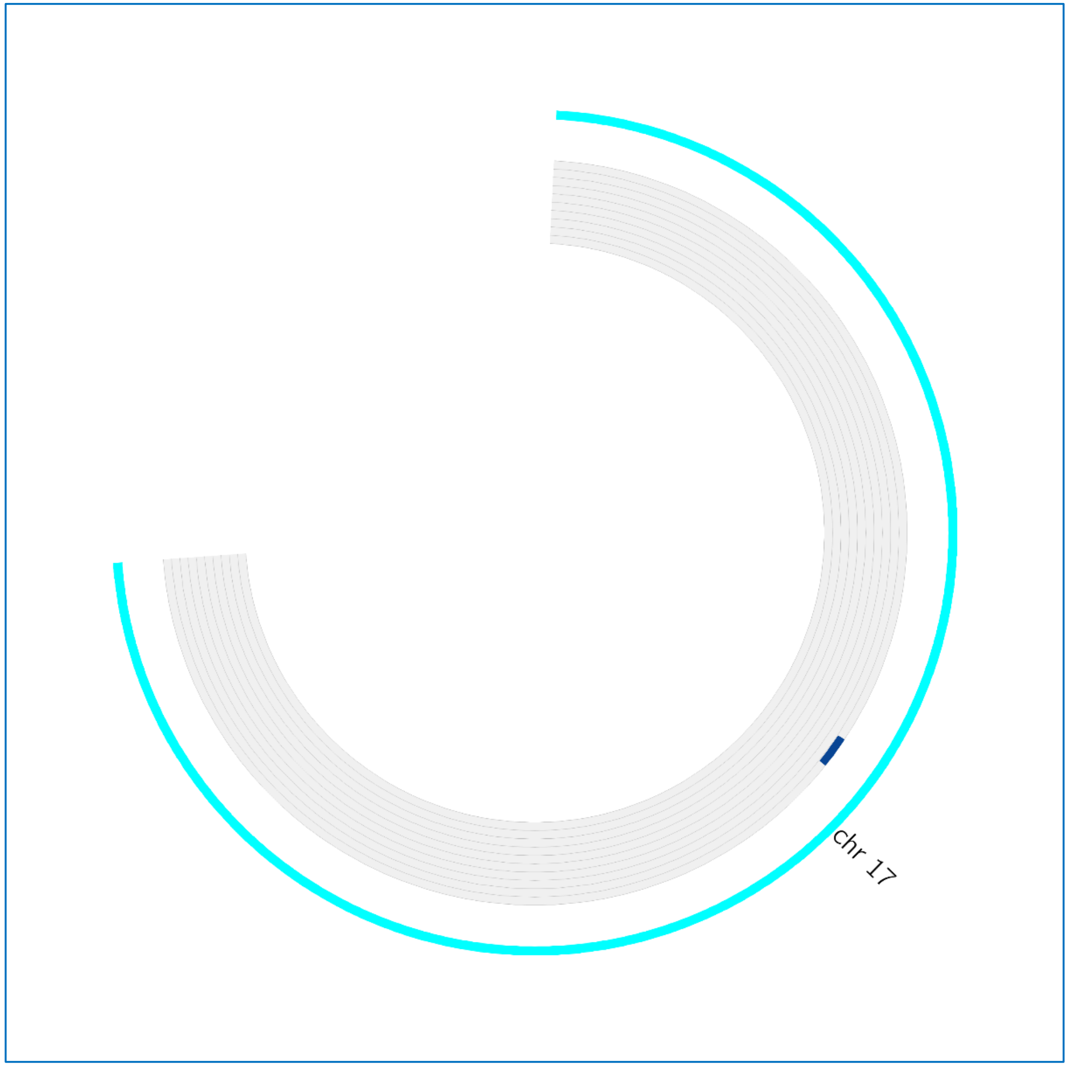

FAST – ERBB2 amplicon

p-22

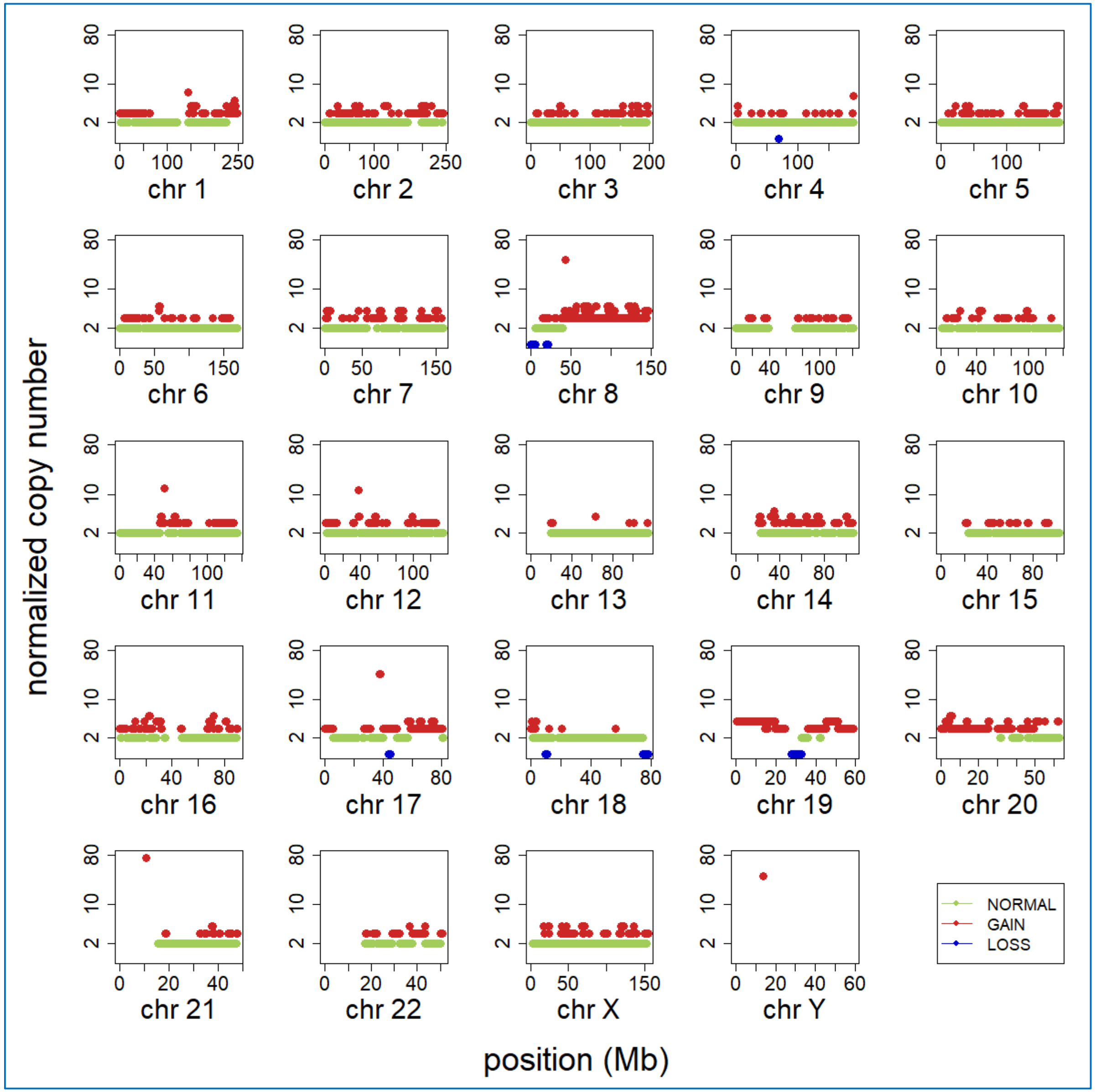

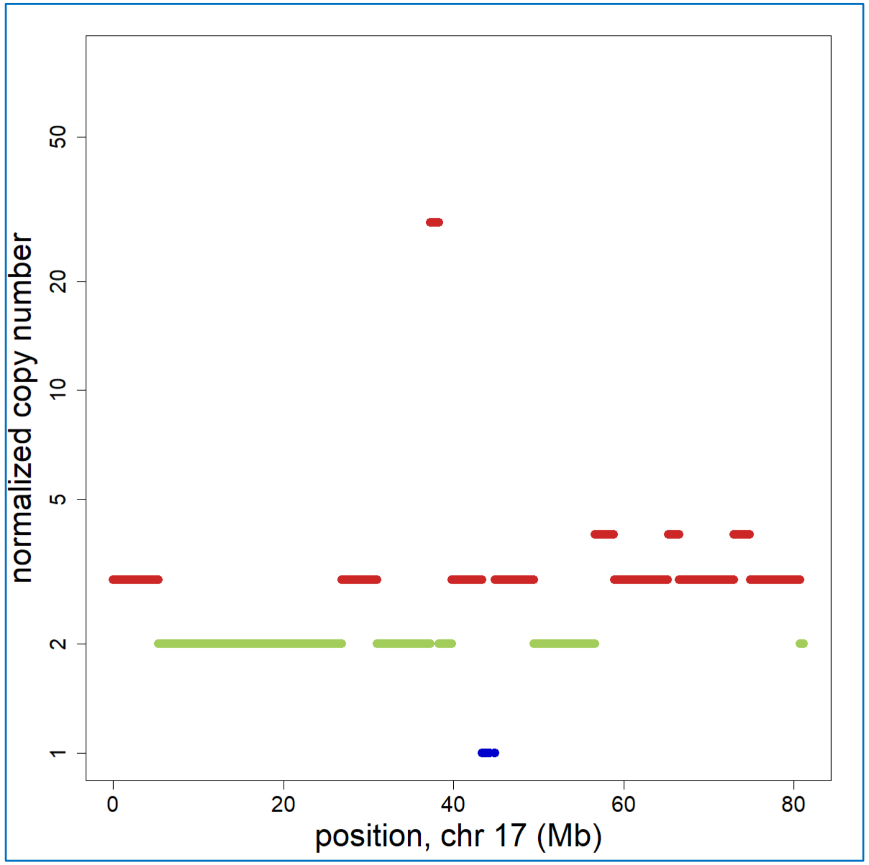

Control-FREEC

p-22

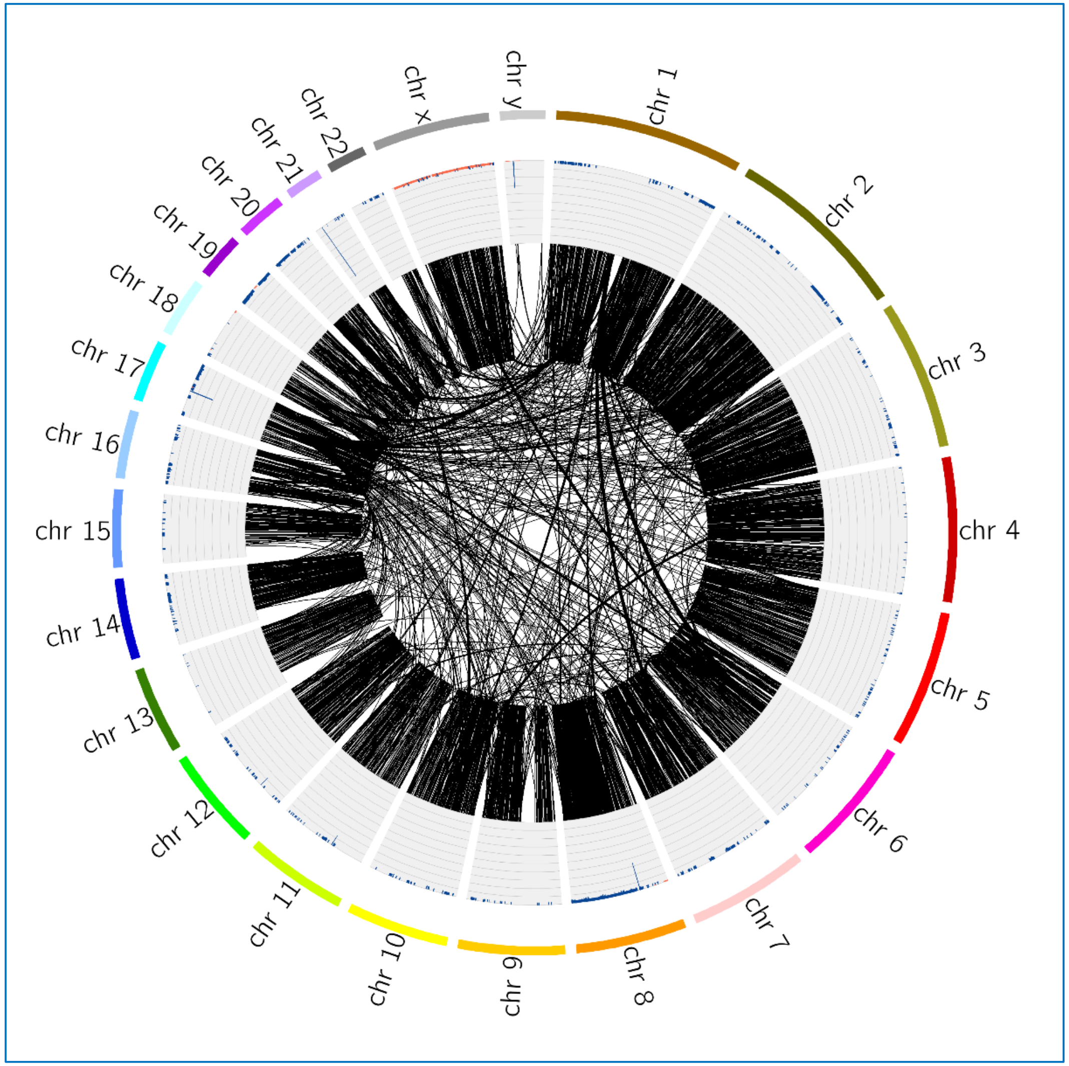

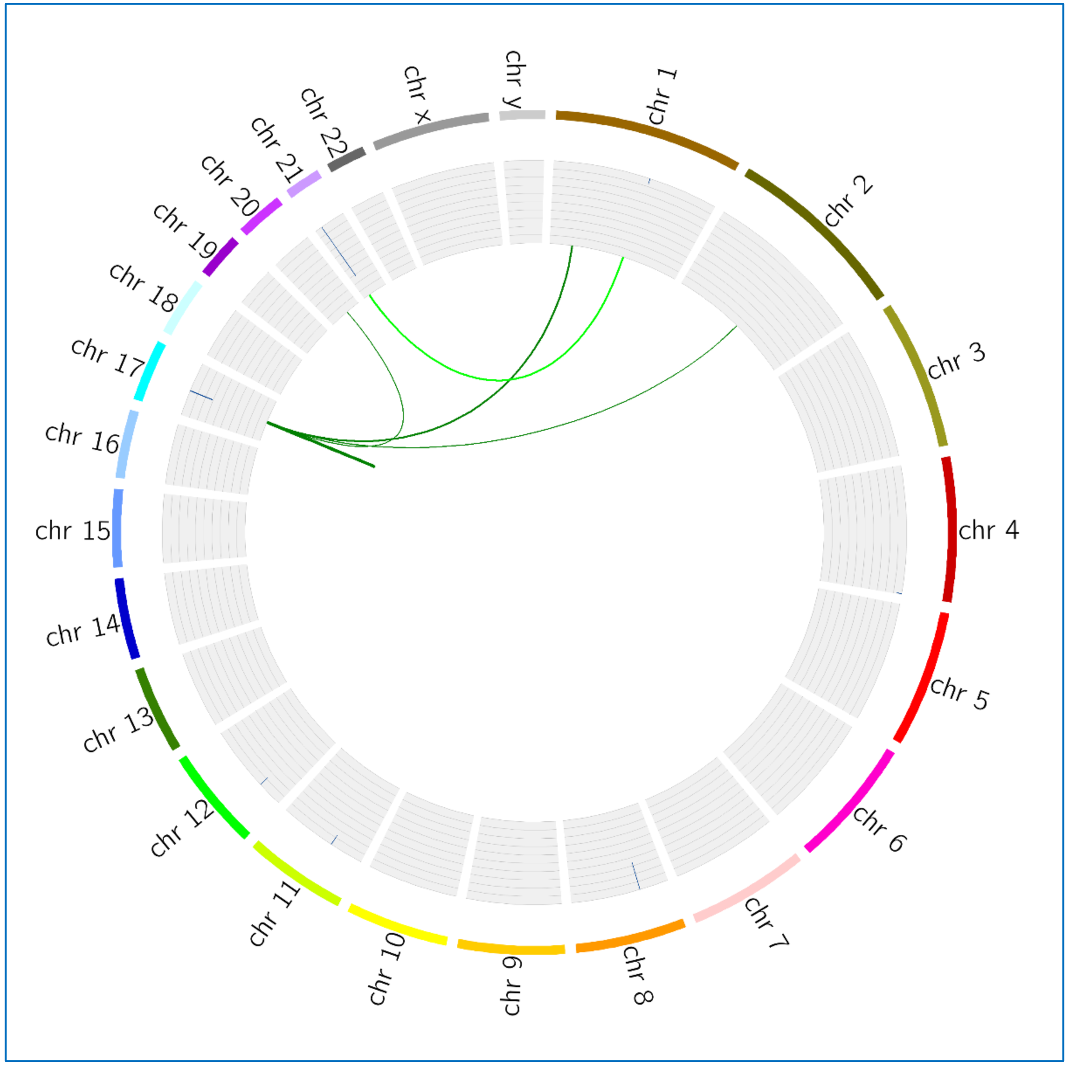

BreakDancer + Control-FREEC

FAST – Whole Genome

p-22

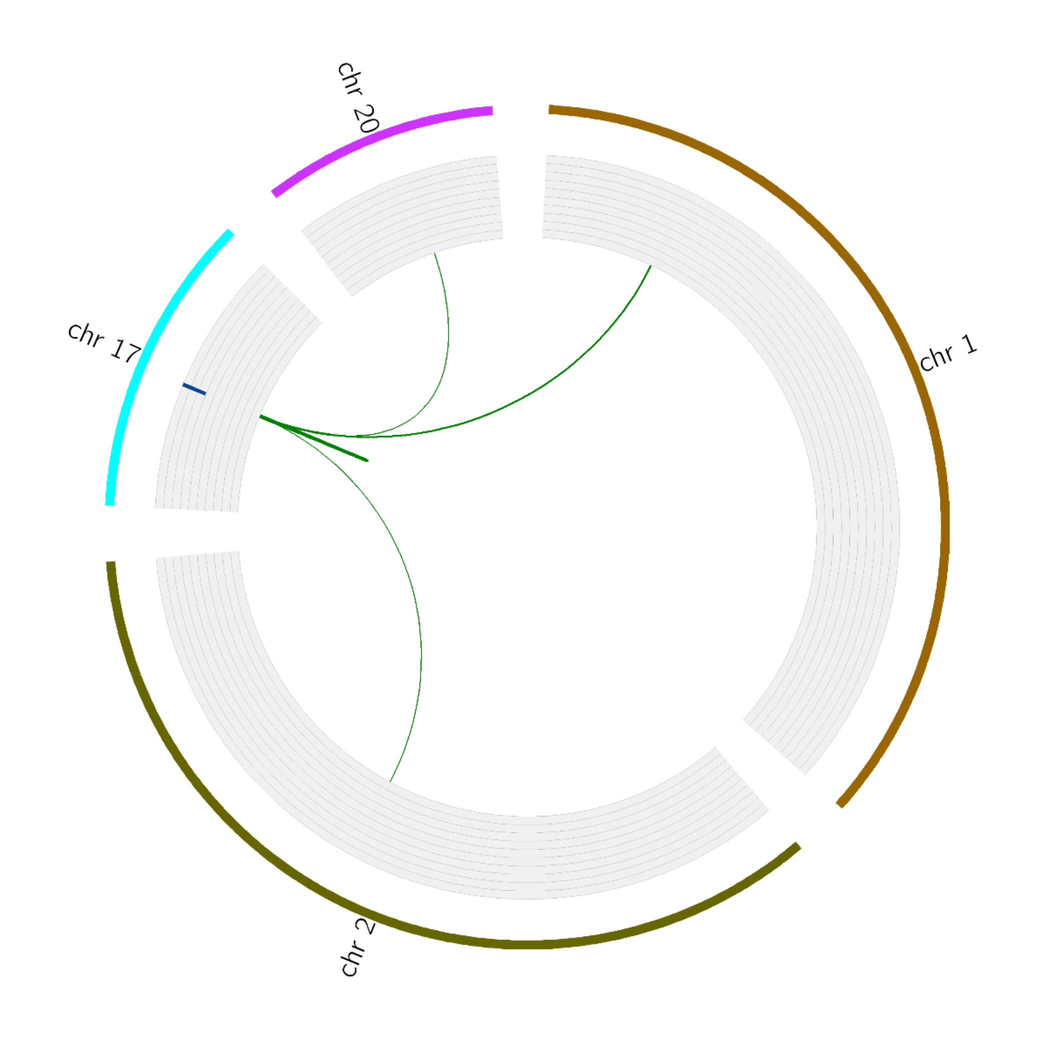

FAST – ERBB2 amplicon

p-23

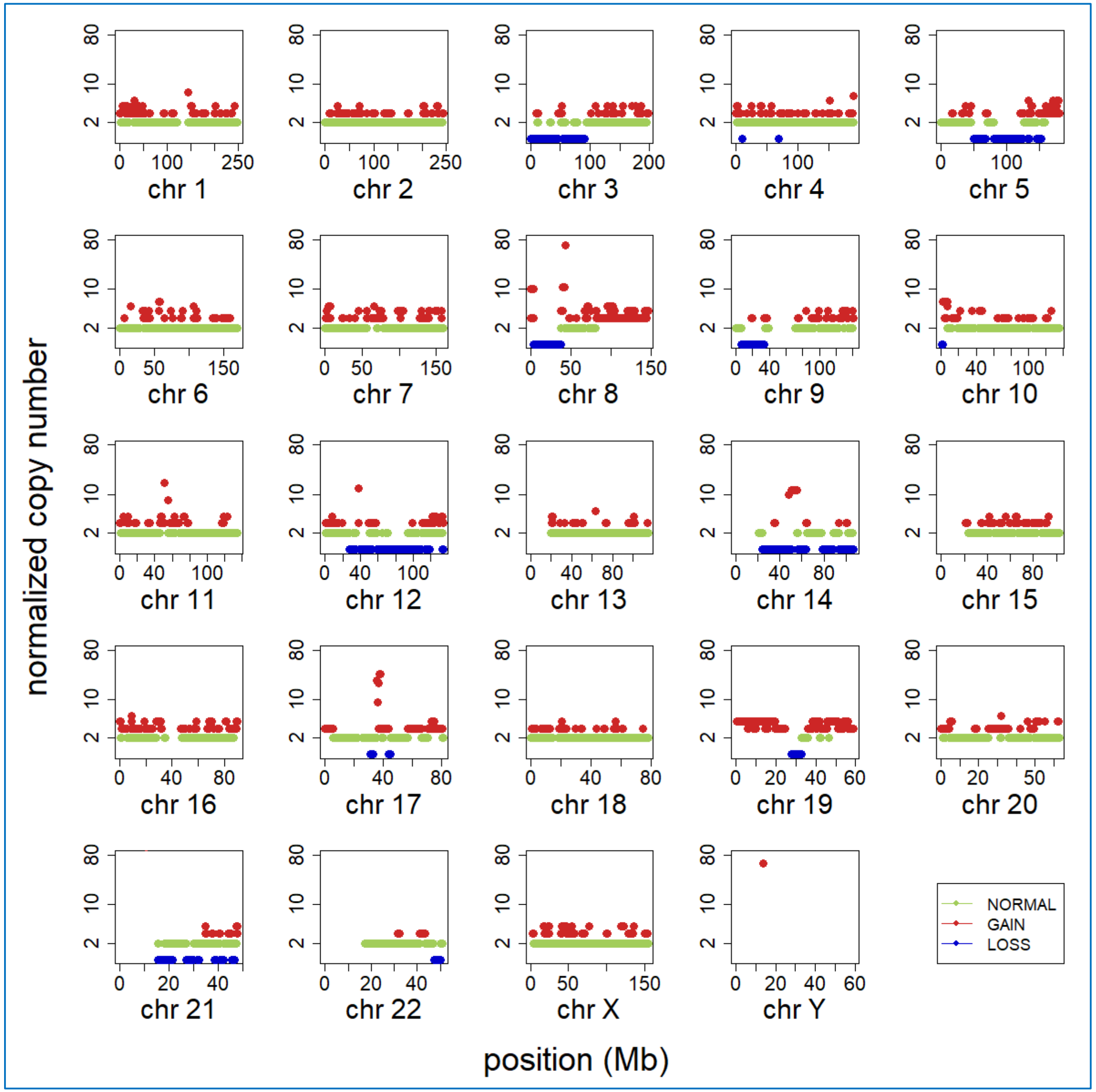

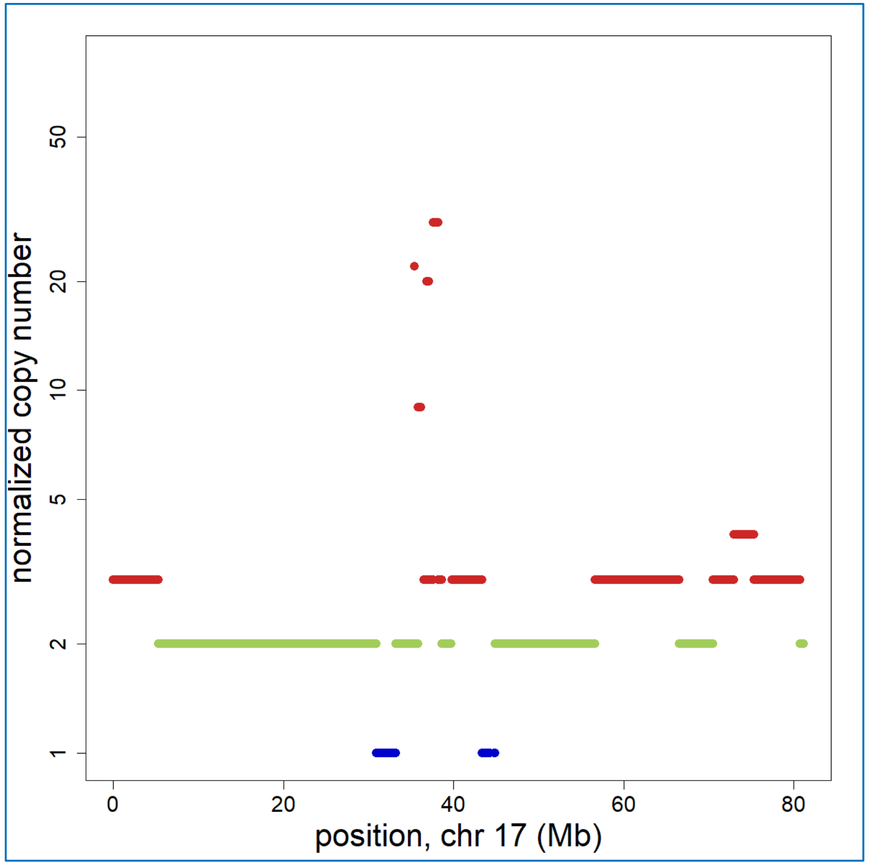

Control-FREEC

p-23

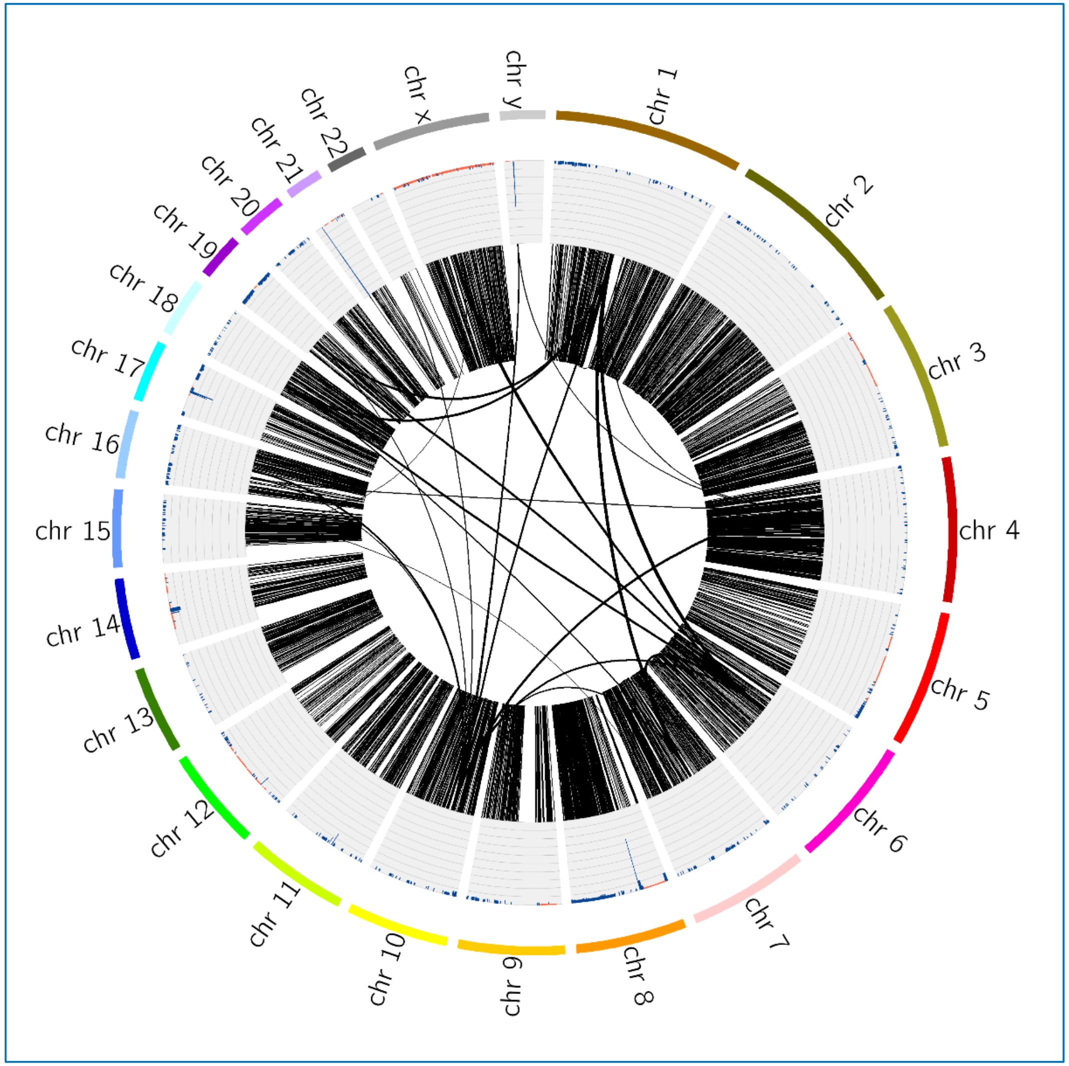

BreakDancer + Control-FREEC

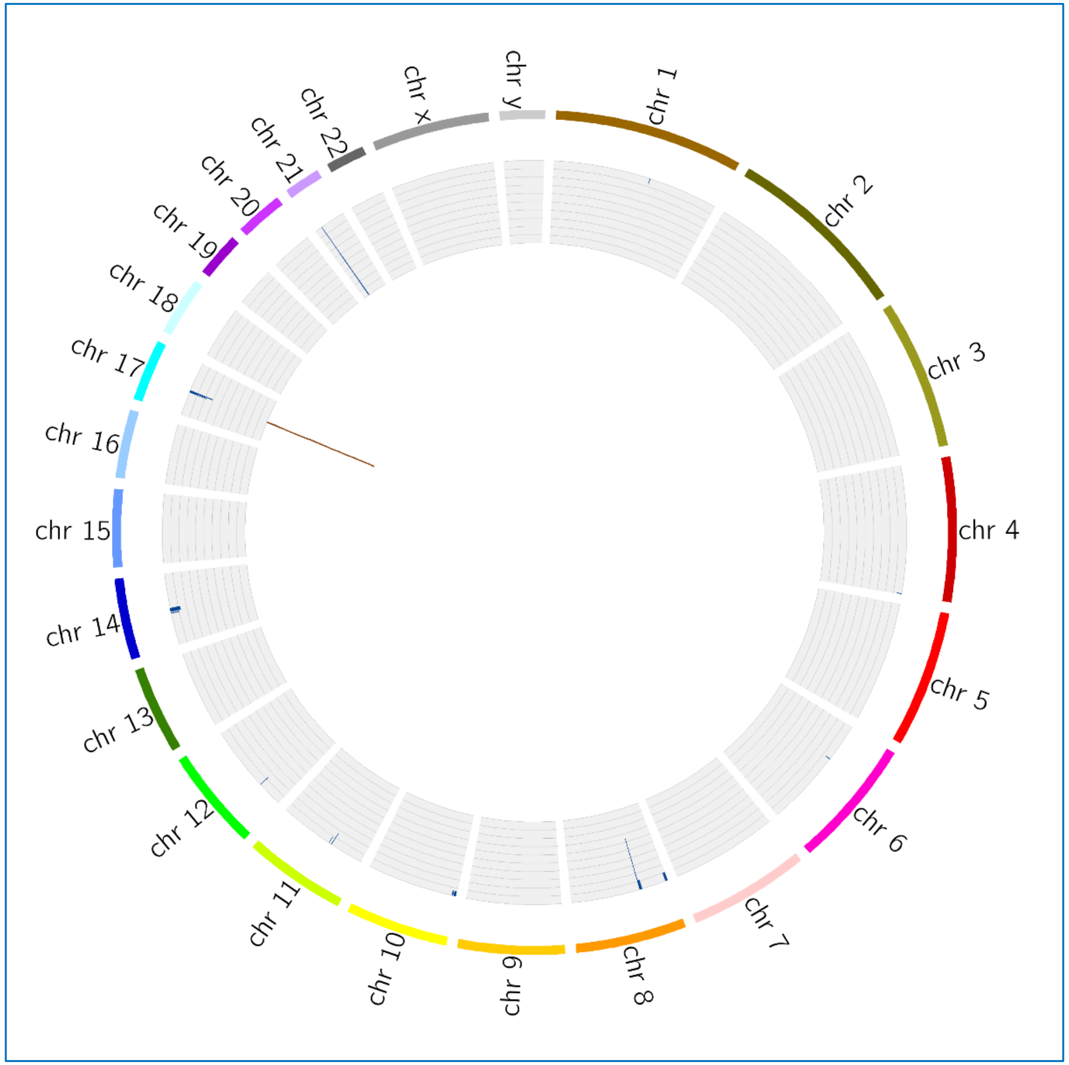

FAST – Whole Genome

p-23

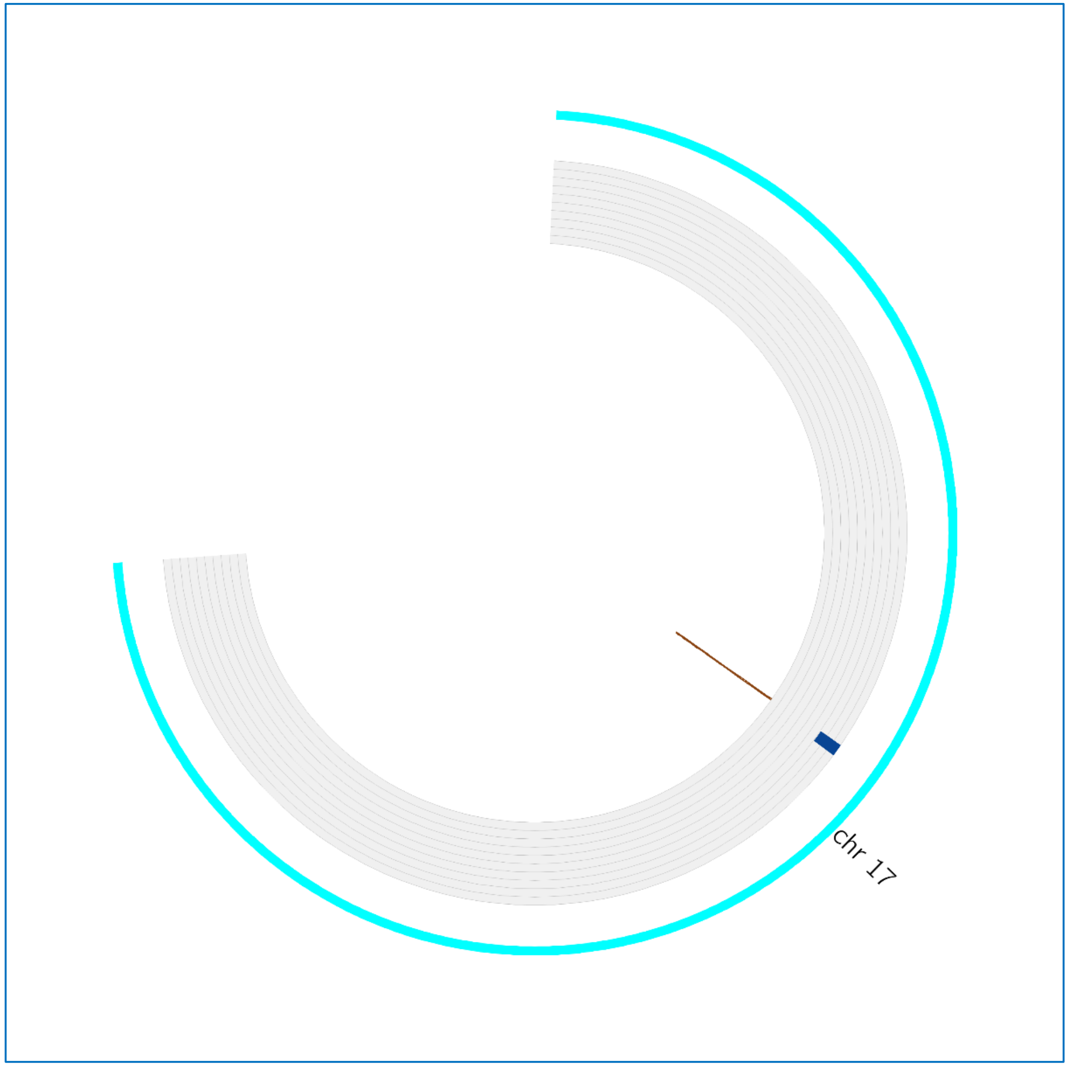

FAST – ERBB2 amplicon

p-25

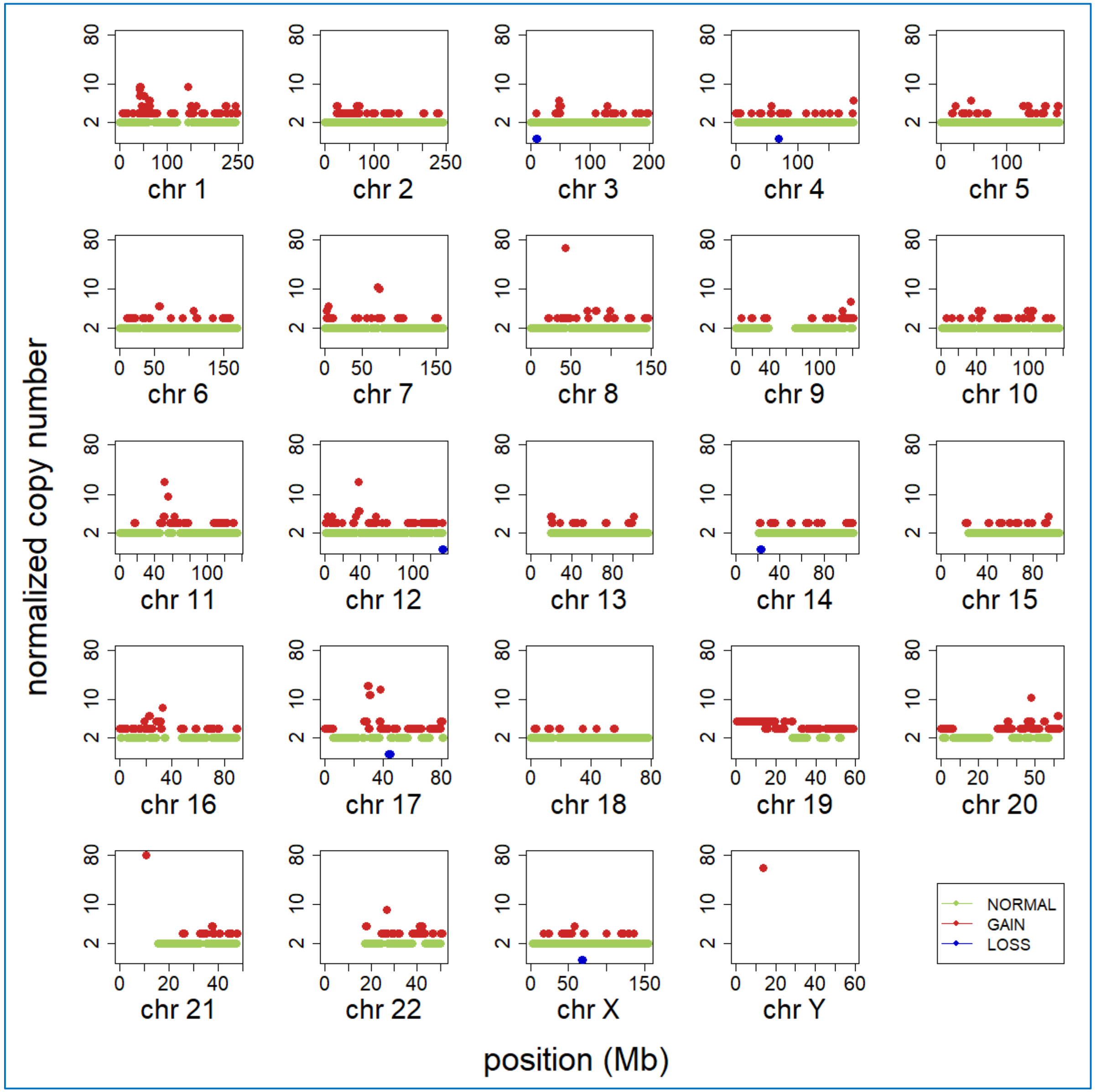

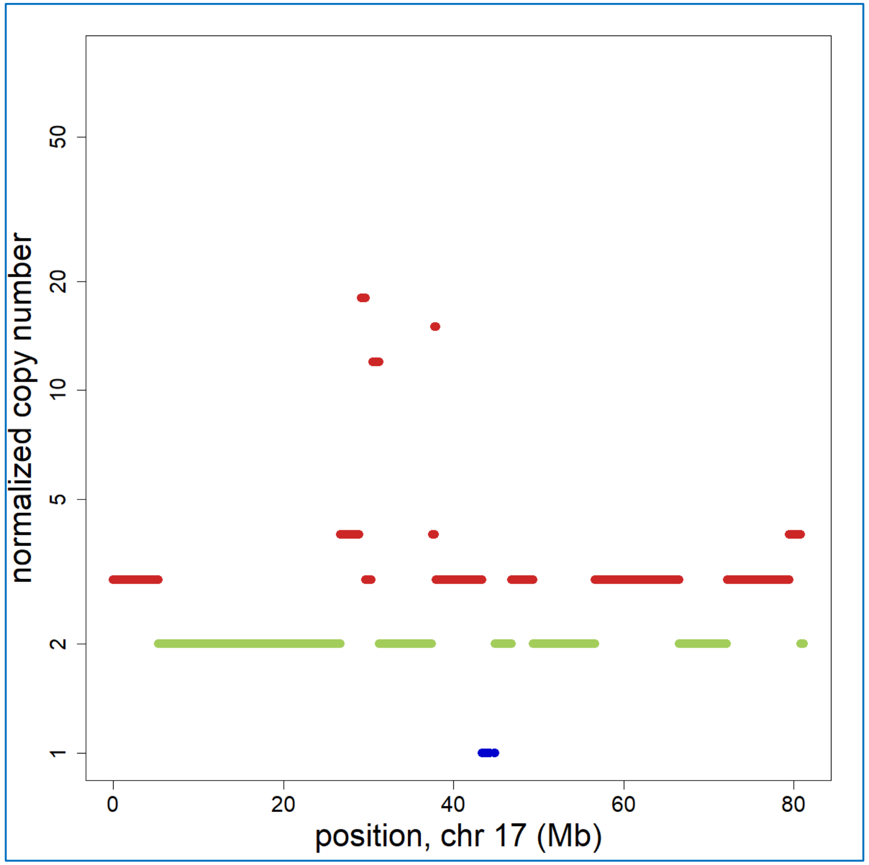

Control-FREEC

p-25

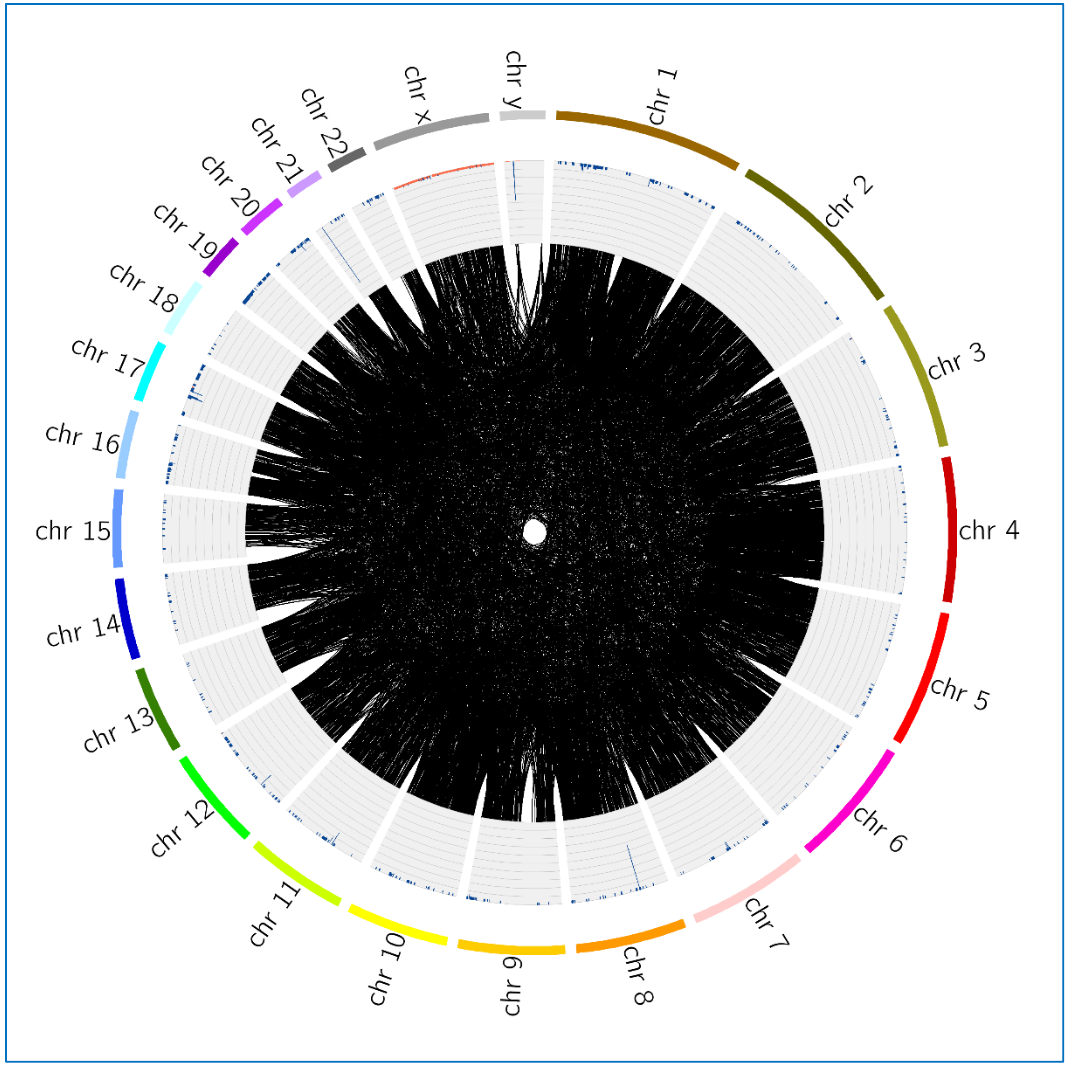

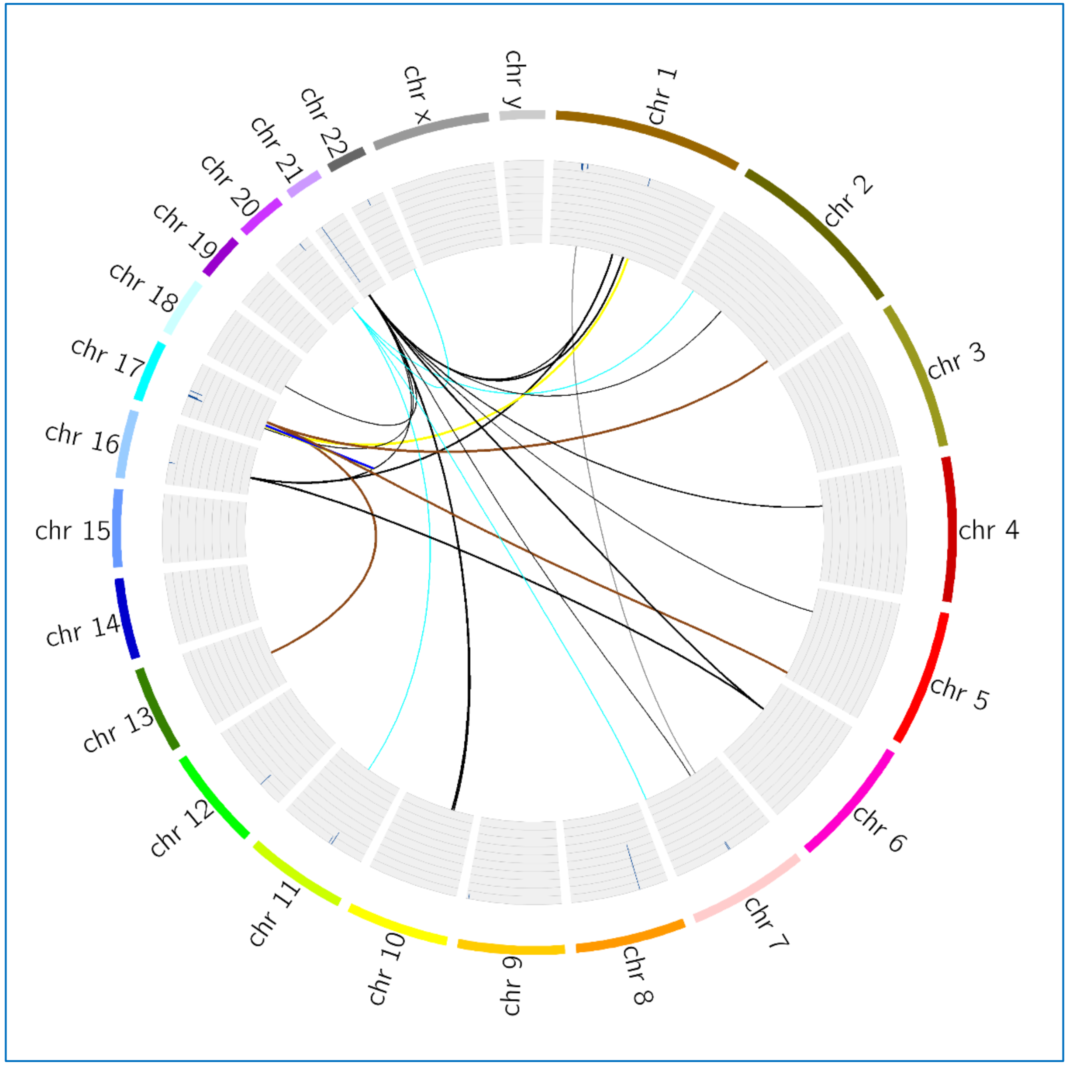

BreakDancer + Control-FREEC

FAST – Whole Genome

p-25

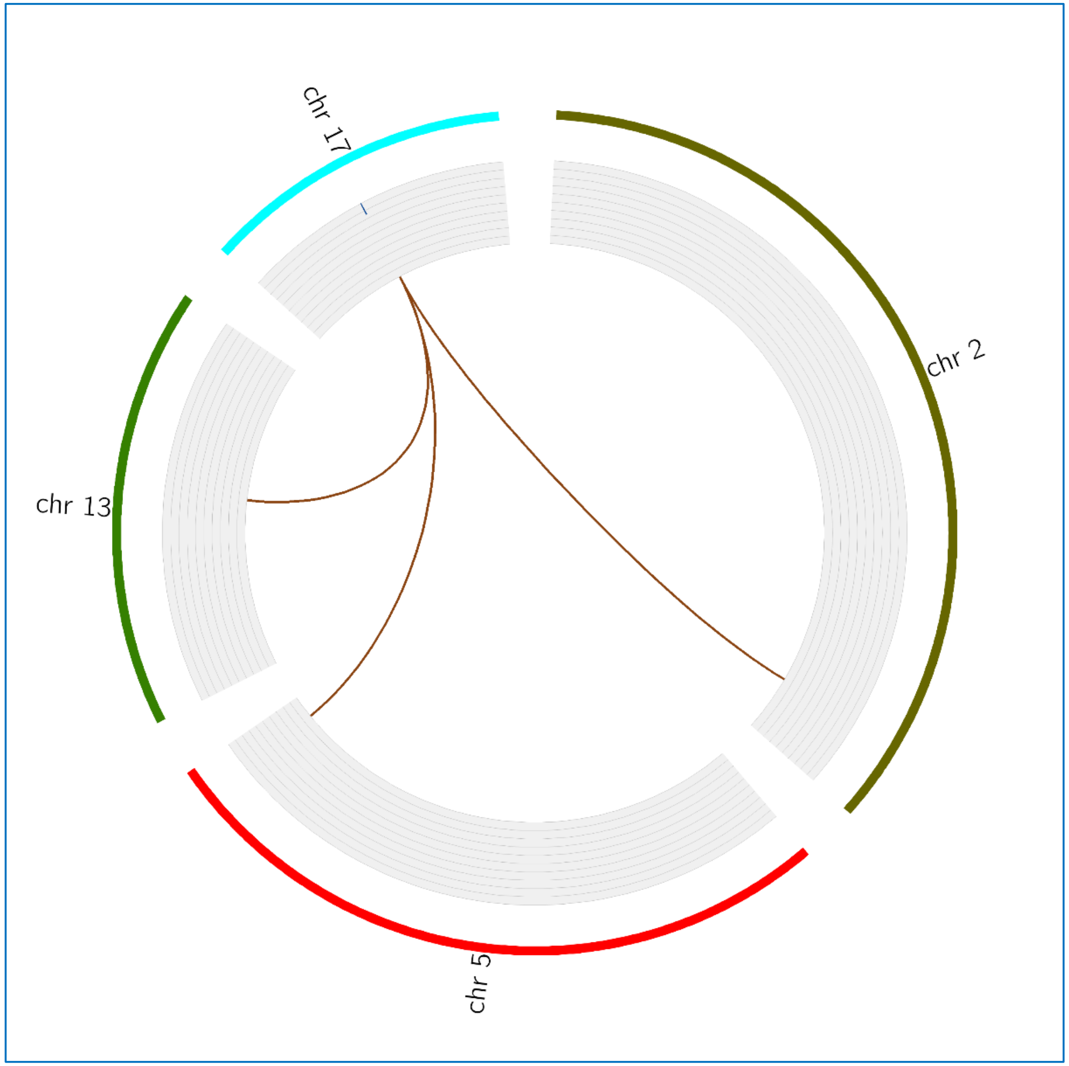

FAST – ERBB2 amplicon

p-27

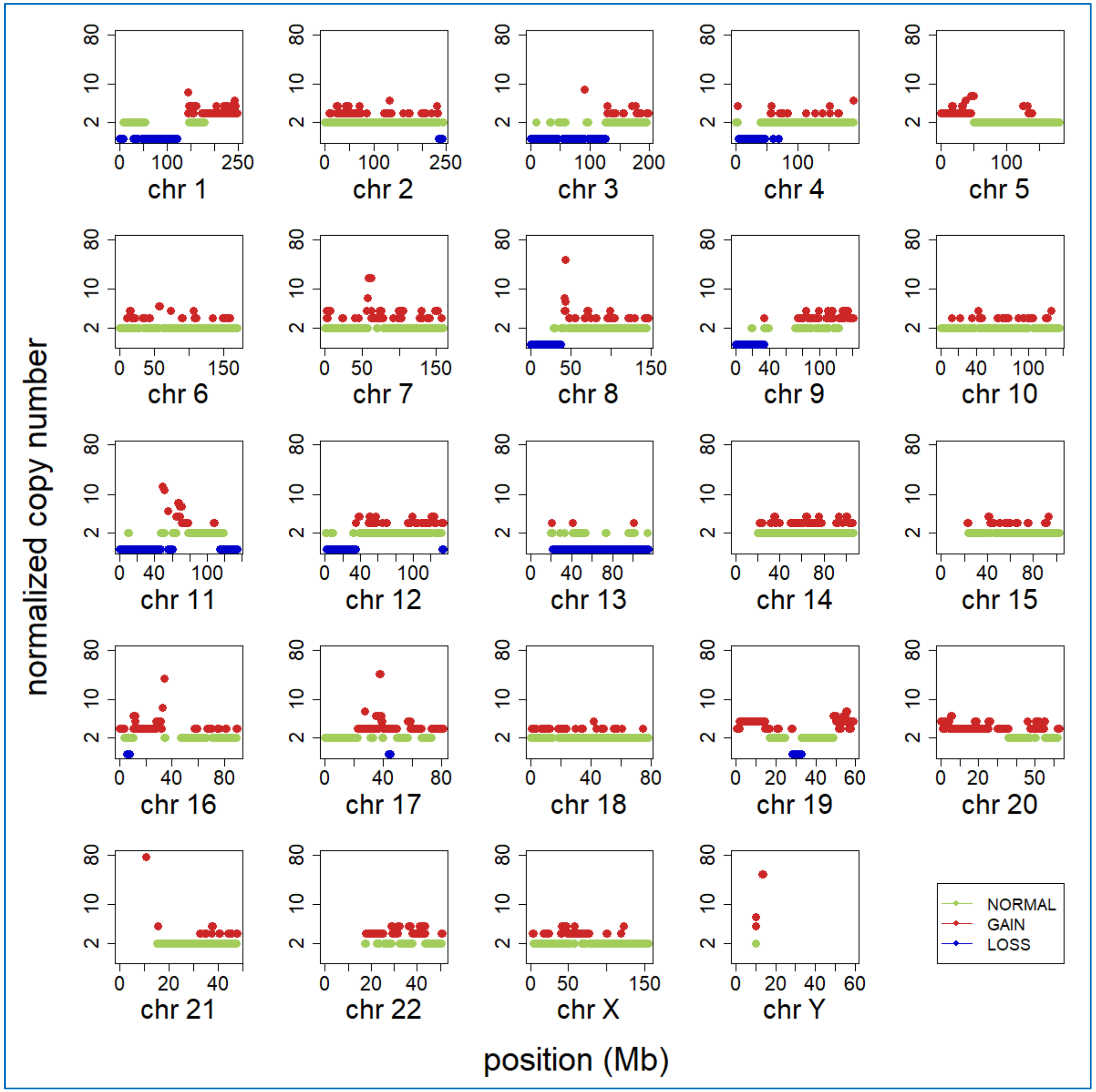

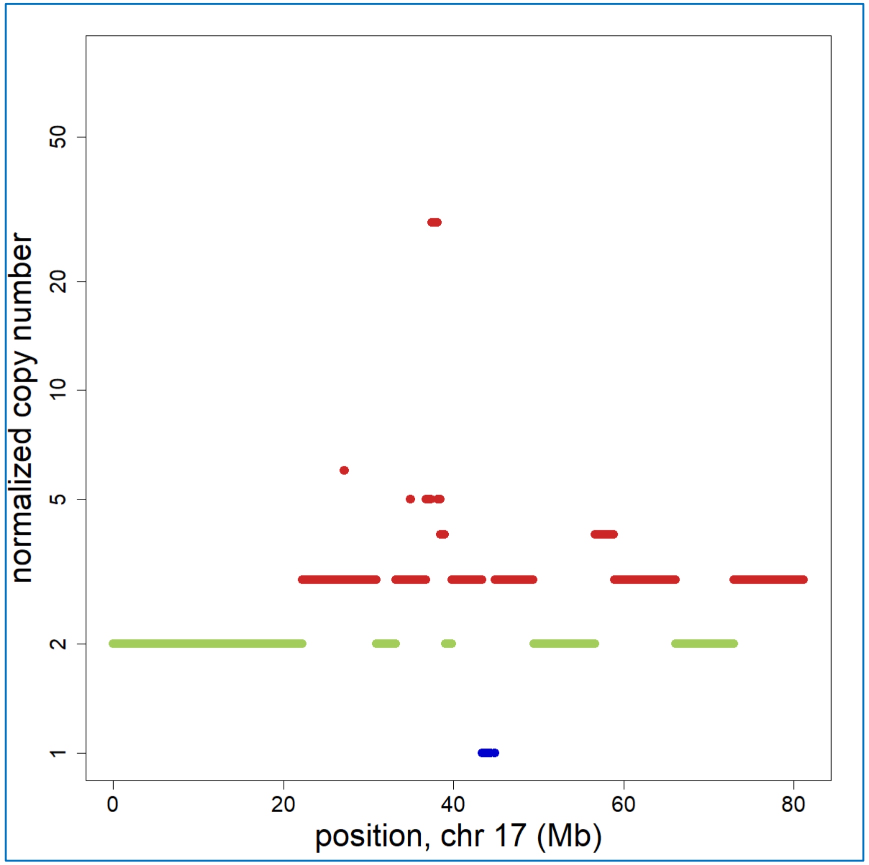

Control-FREEC

p-27

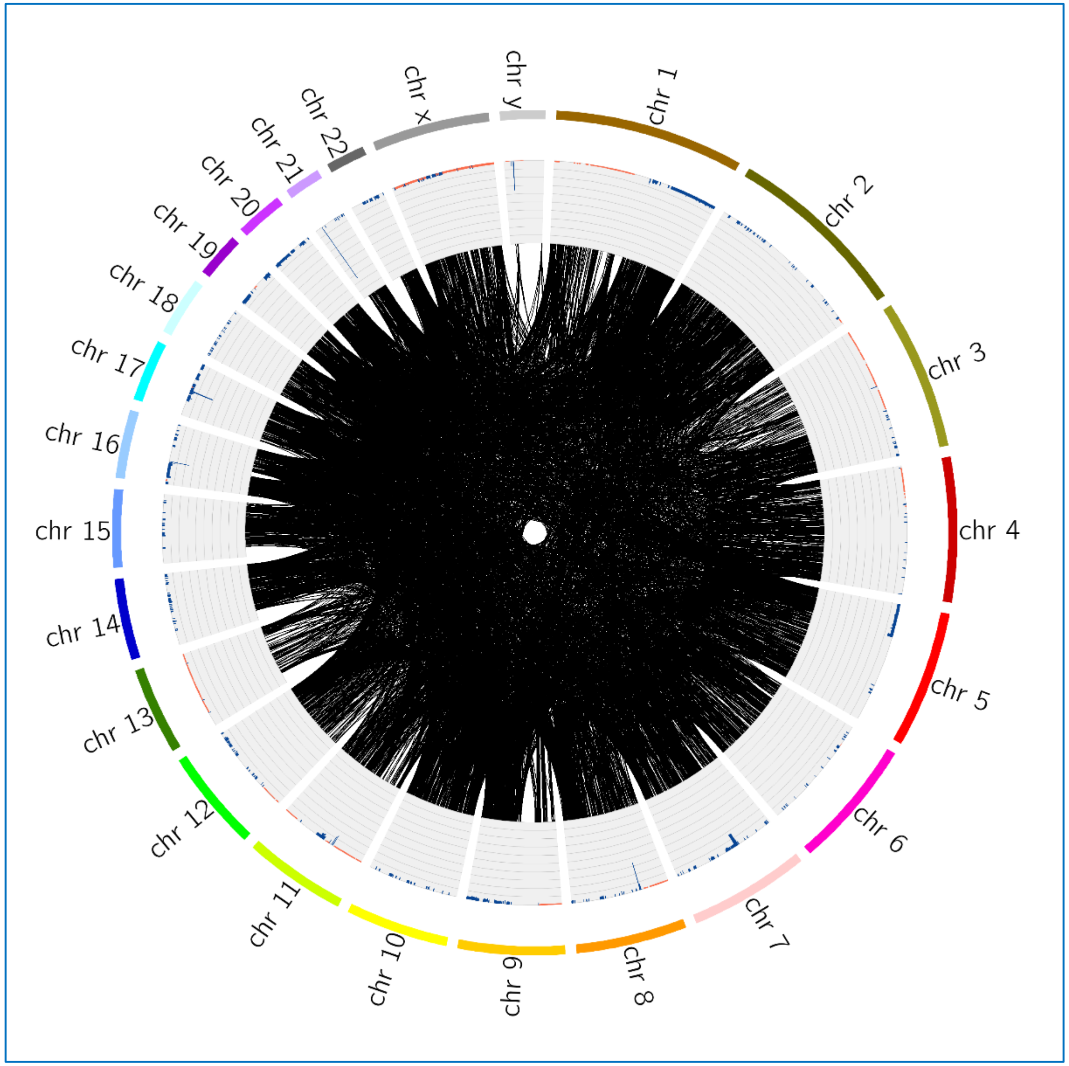

BreakDancer + Control-FREEC

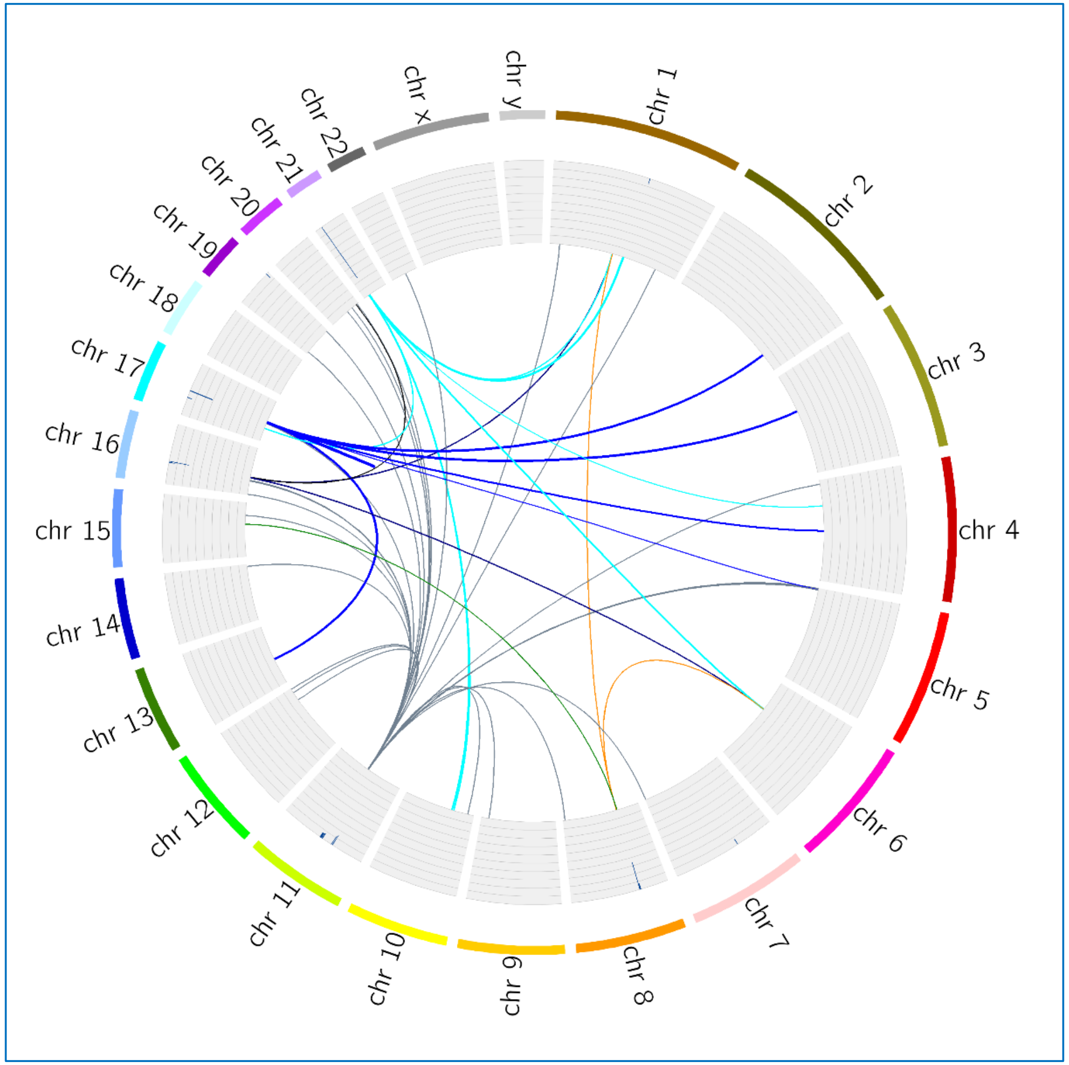

FAST – Whole Genome

p-27

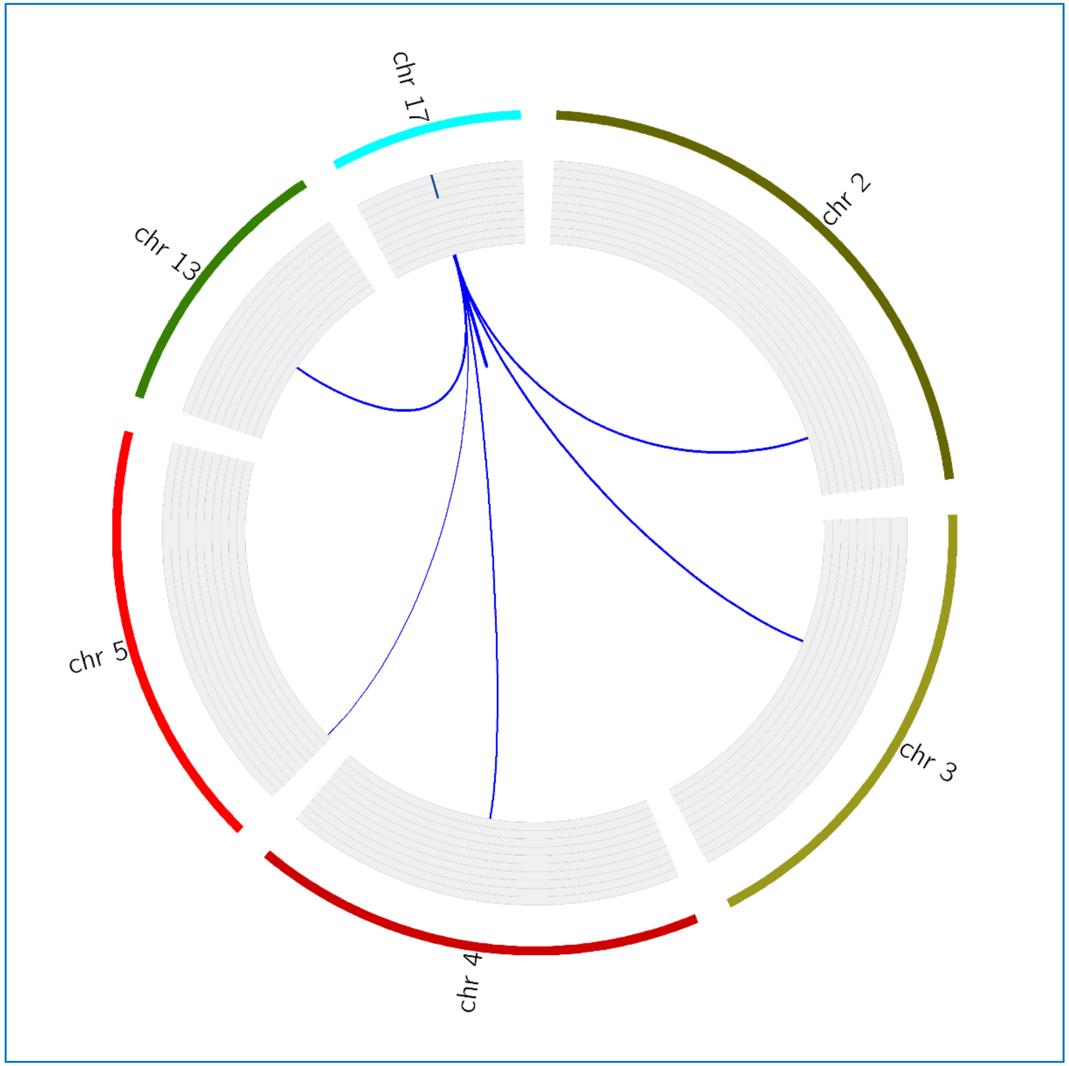

FAST – ERBB2 amplicon

p-28

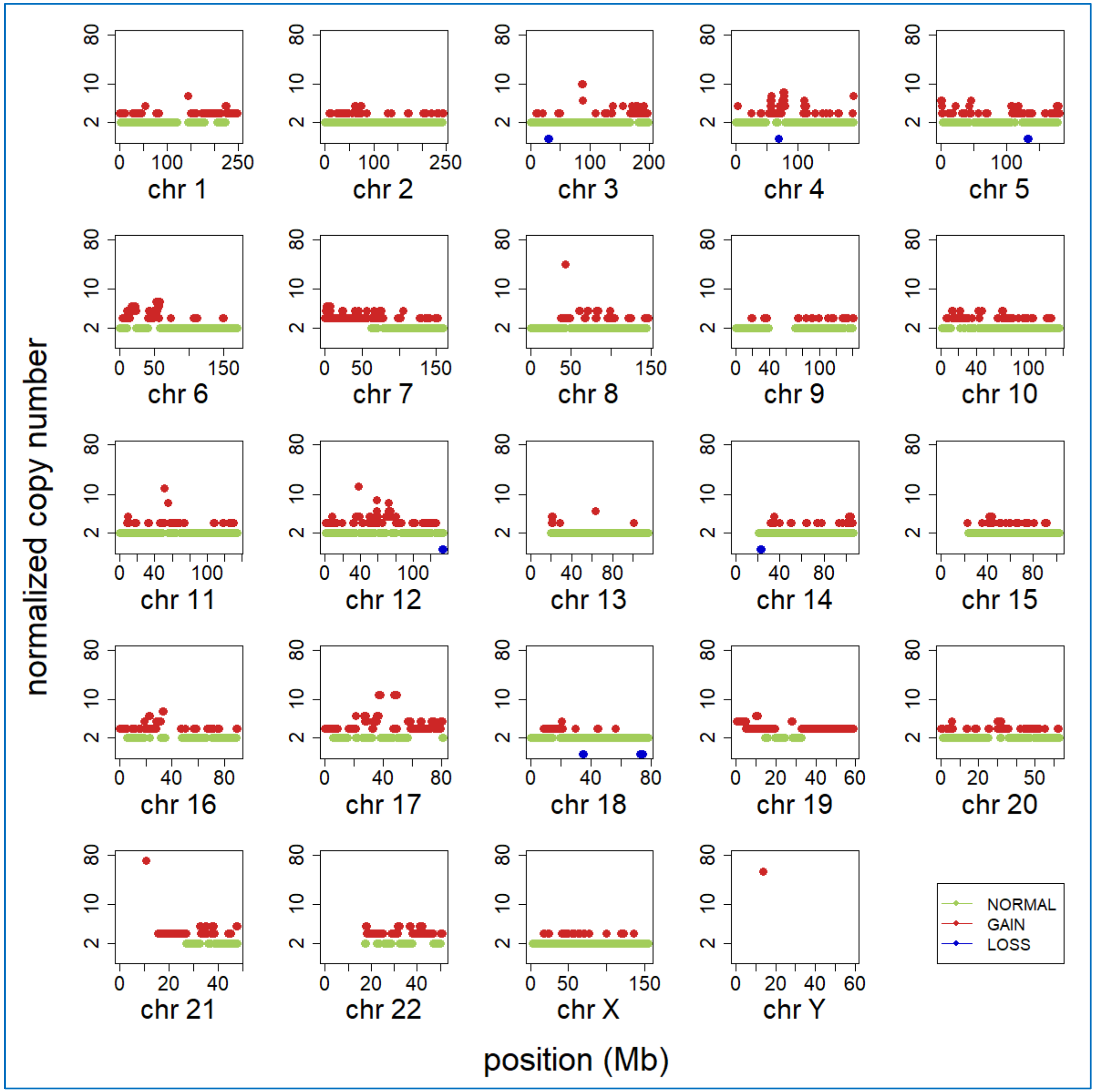

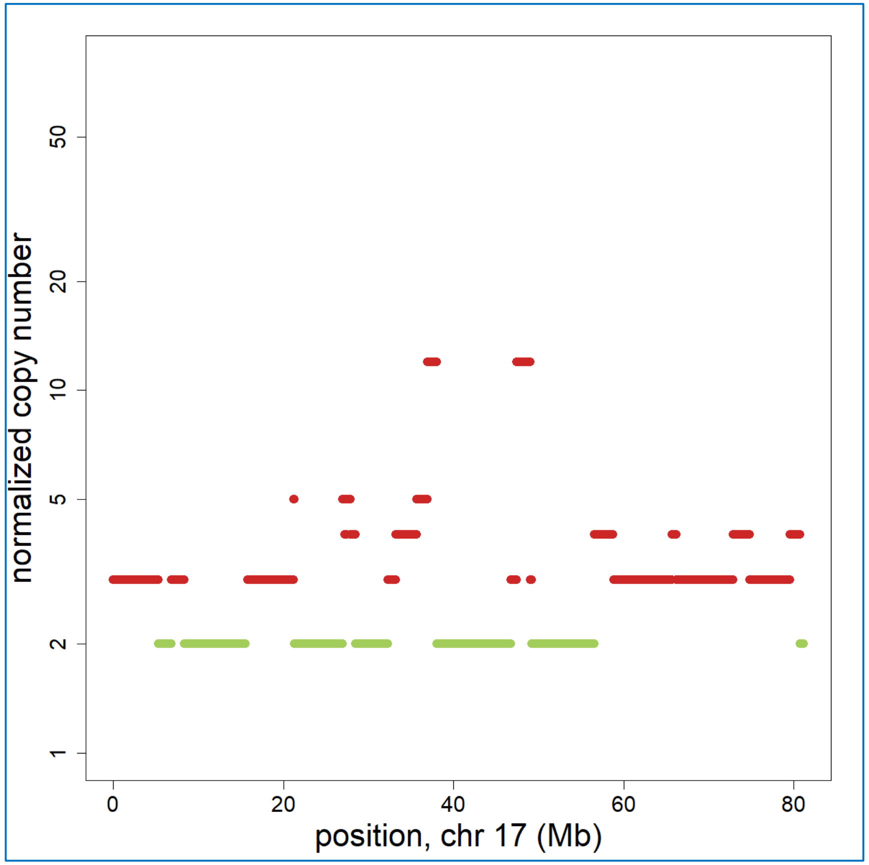

Control-FREEC

p-28

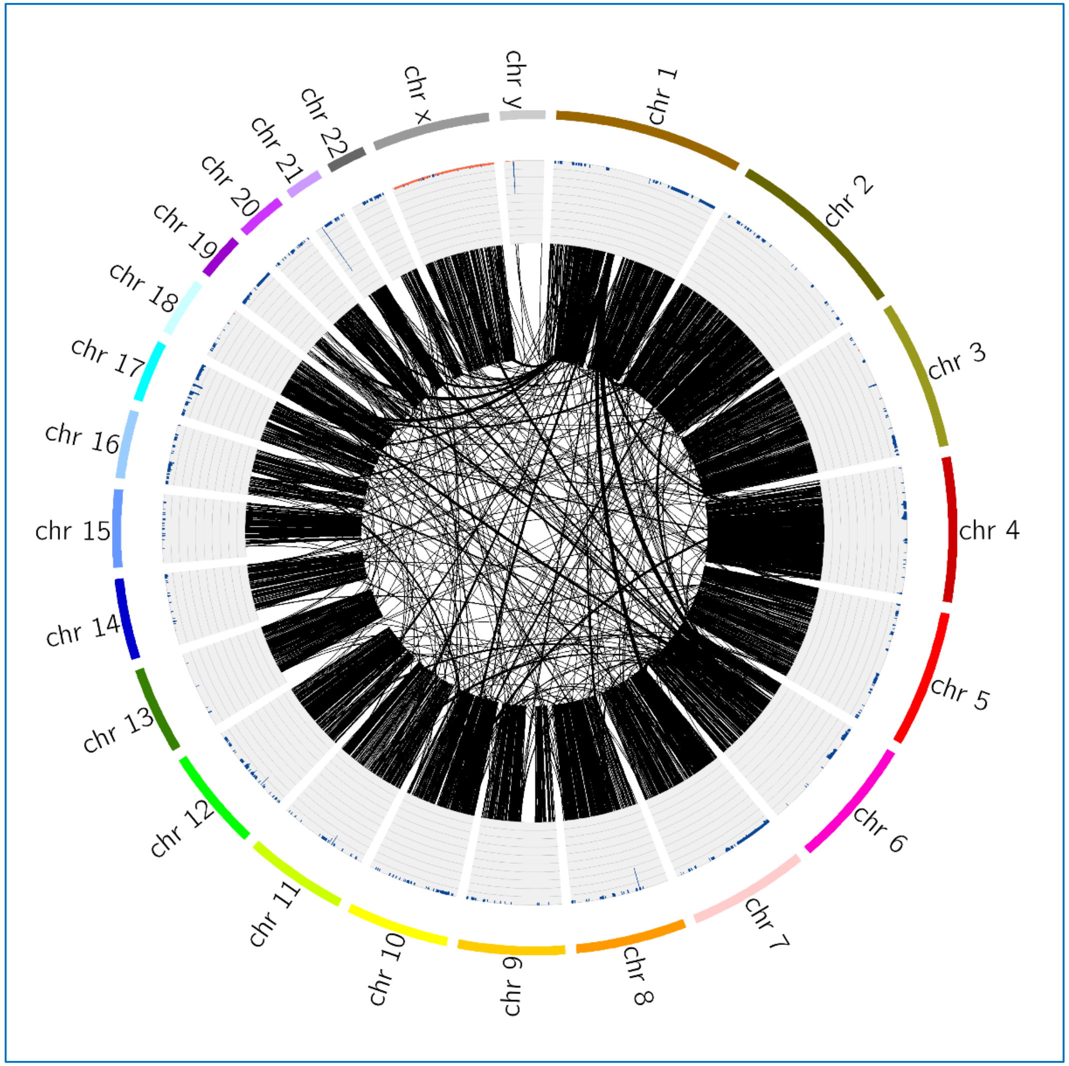

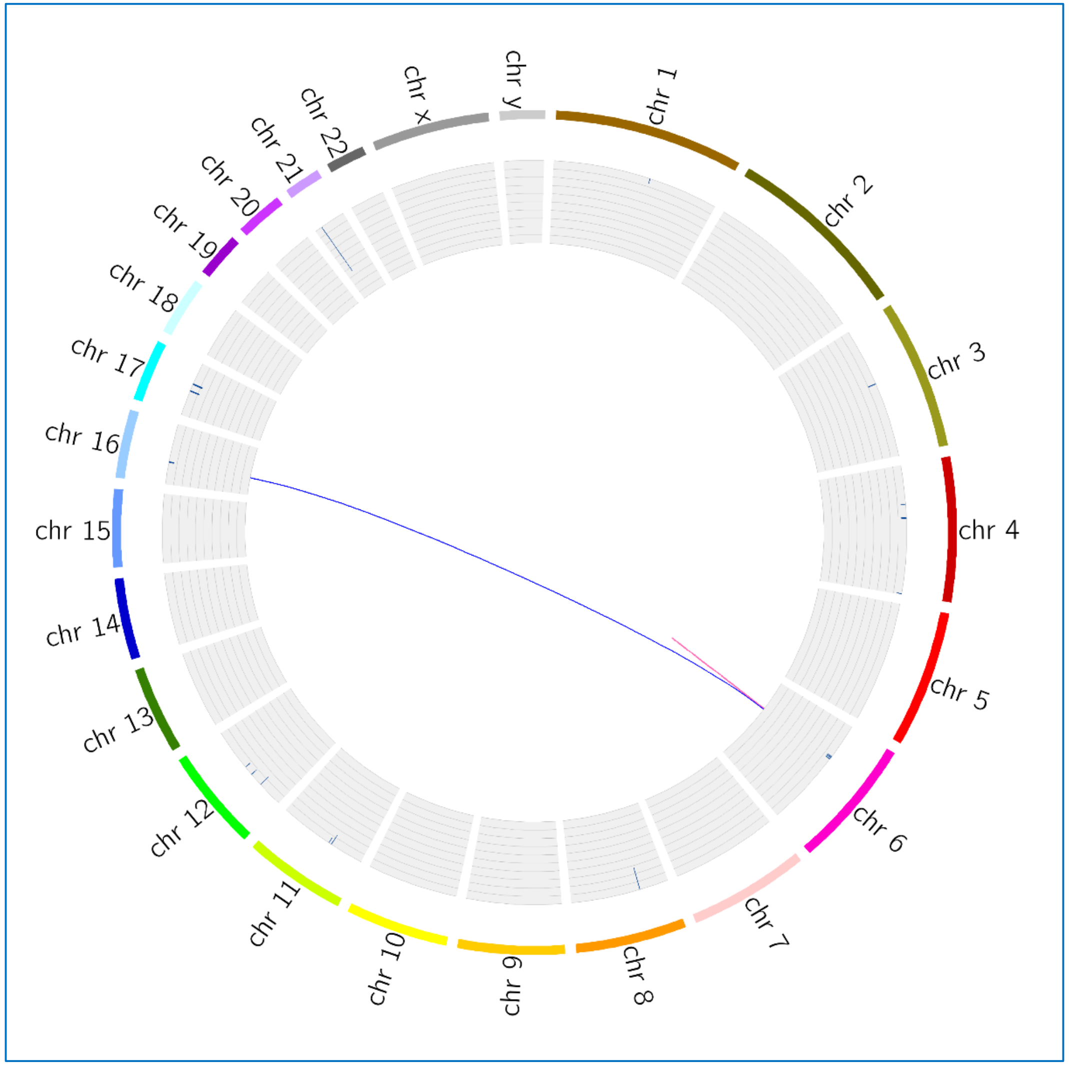

BreakDancer + Control-FREEC

FAST – Whole Genome

p-28

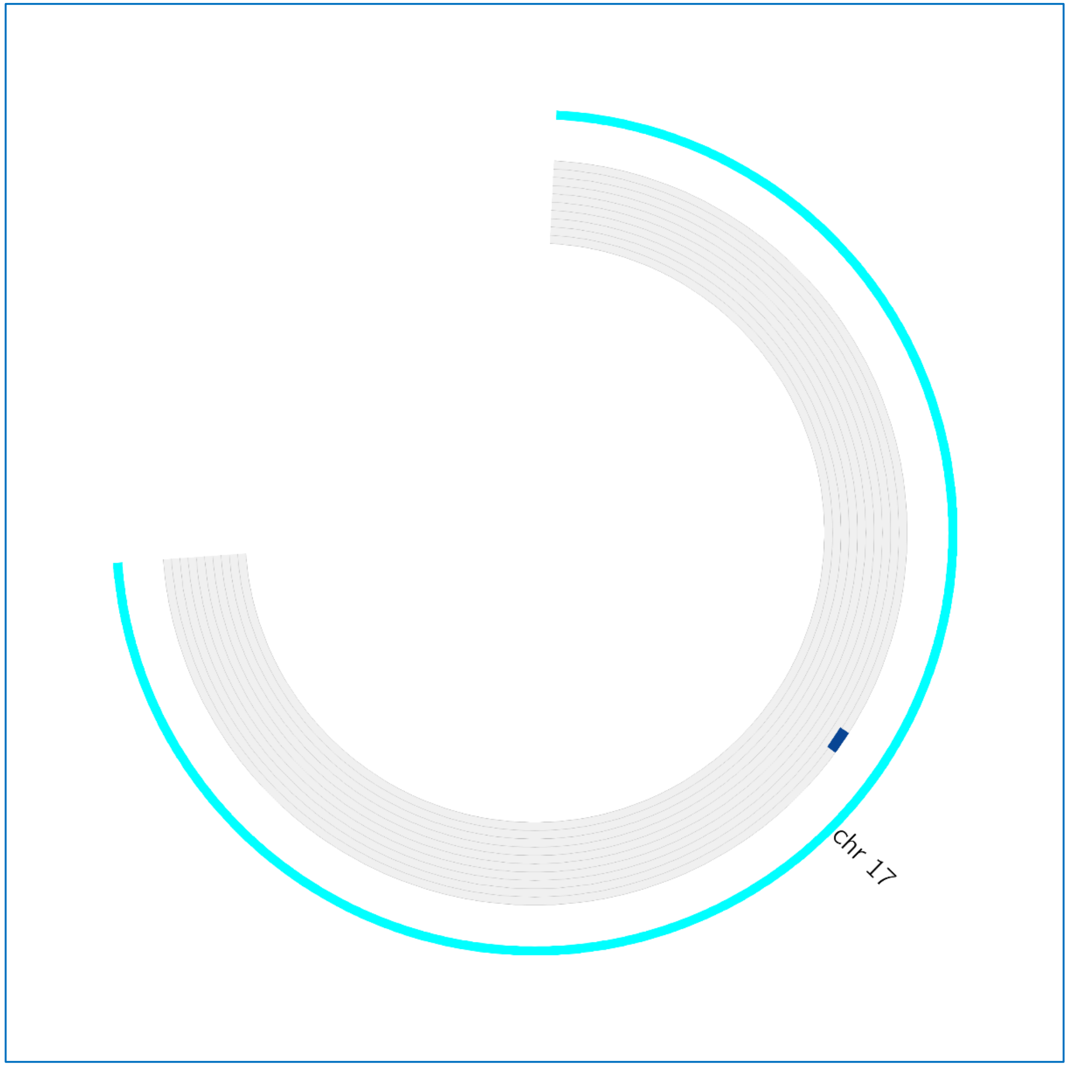

FAST – ERBB2 amplicon

p-29

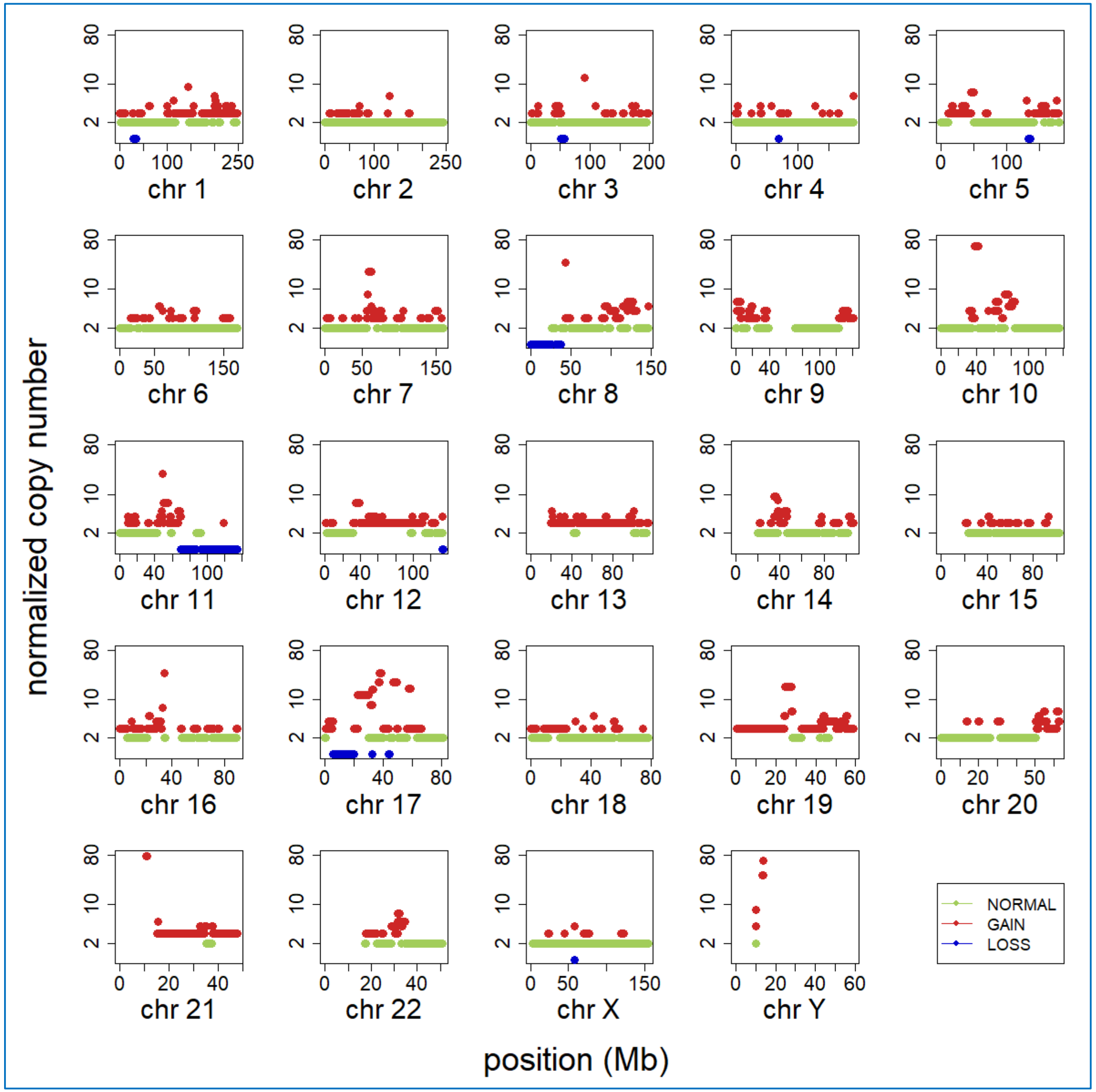

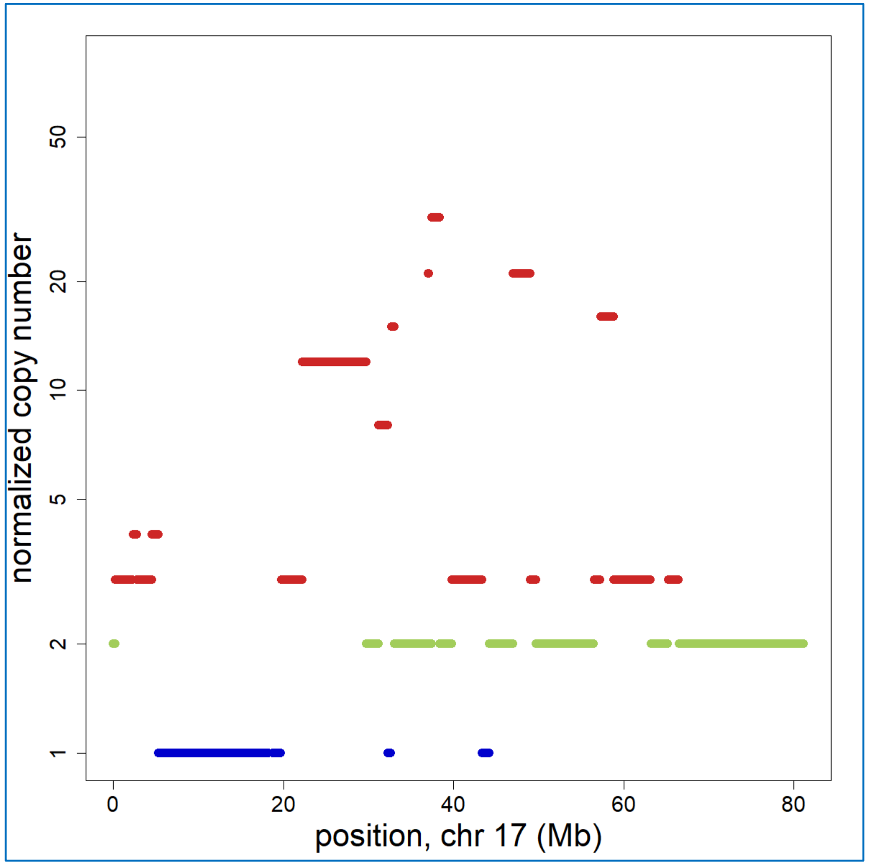

Control-FREEC

p-29

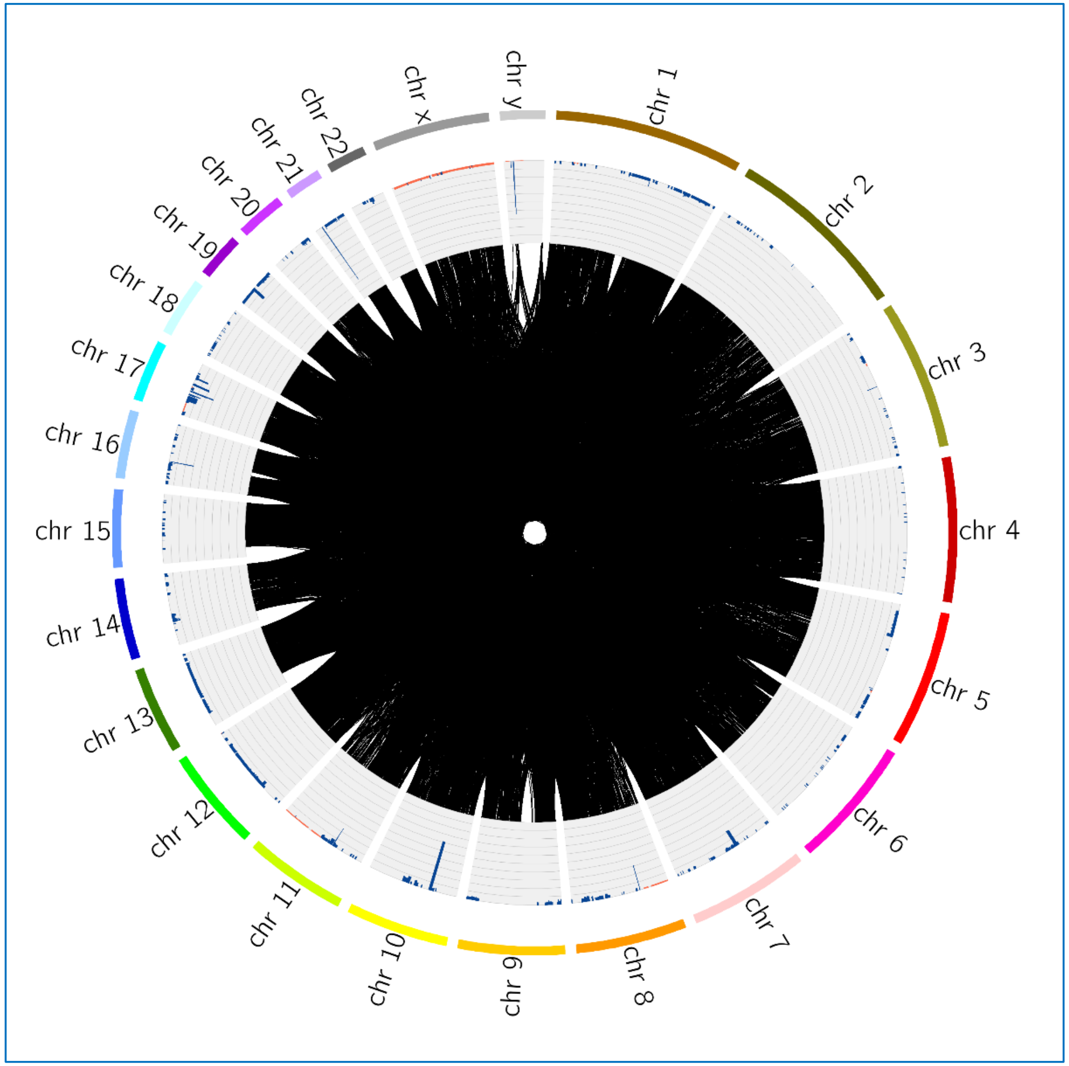

BreakDancer + Control-FREEC

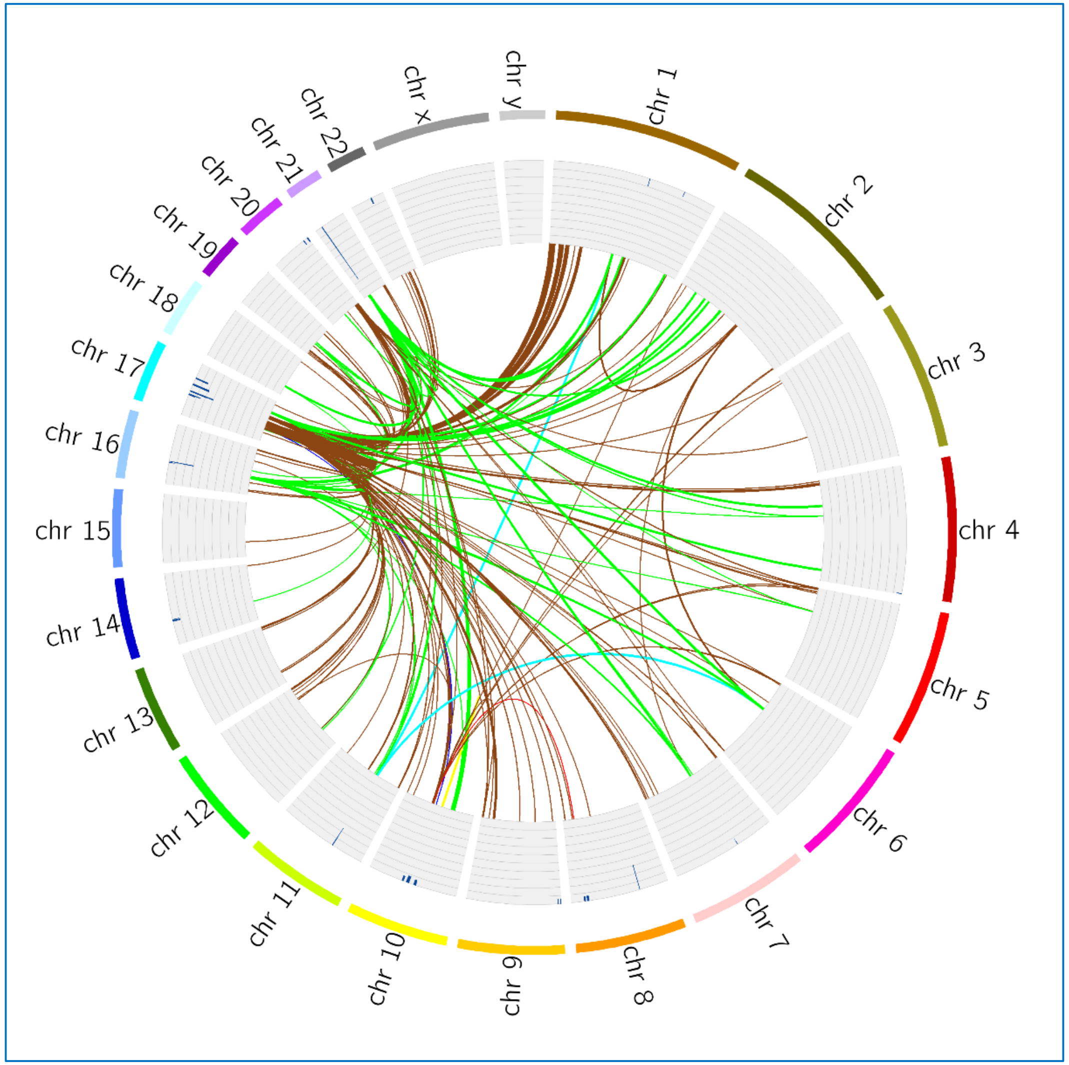

FAST – Whole Genome

p-29

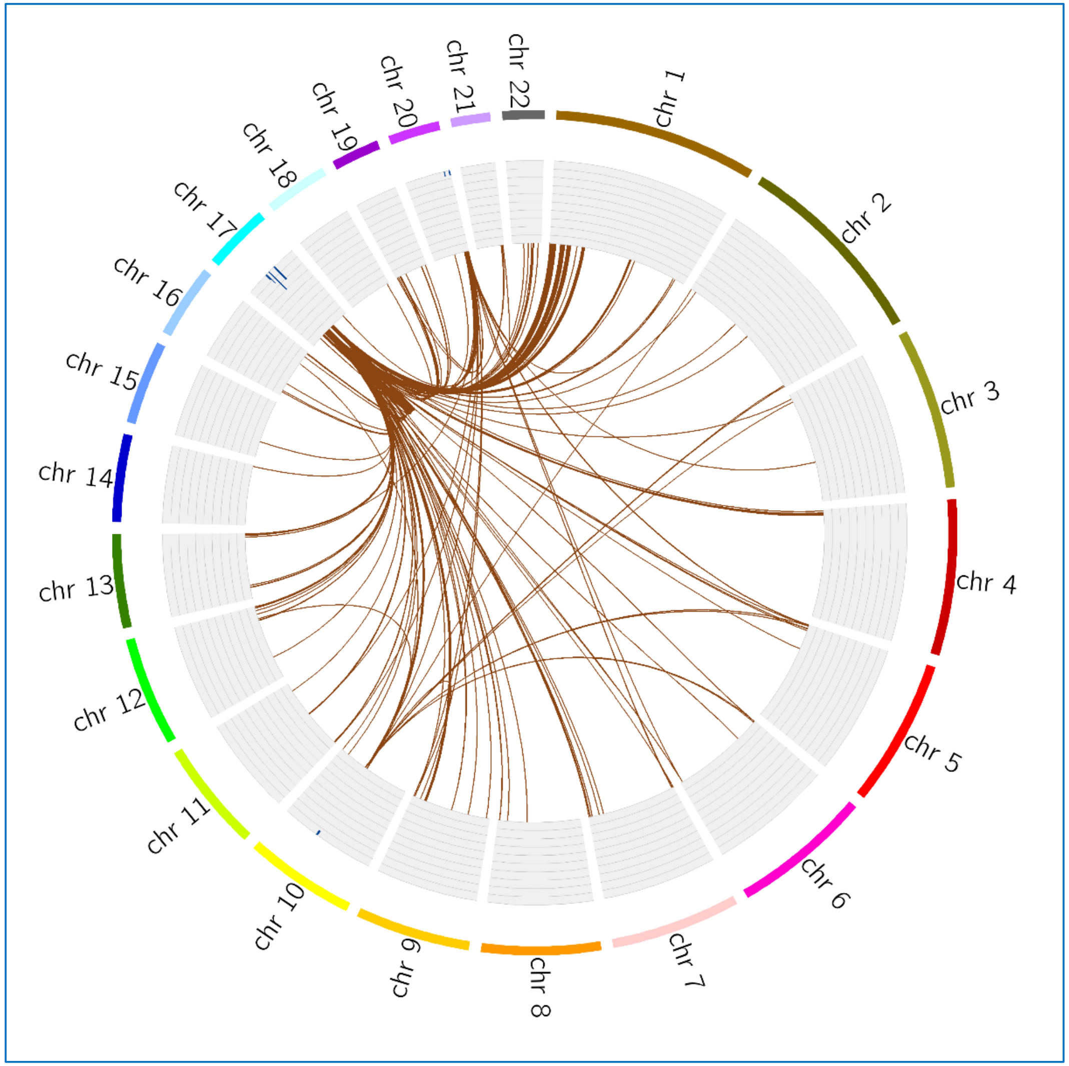

FAST – ERBB2 amplicon

p-30

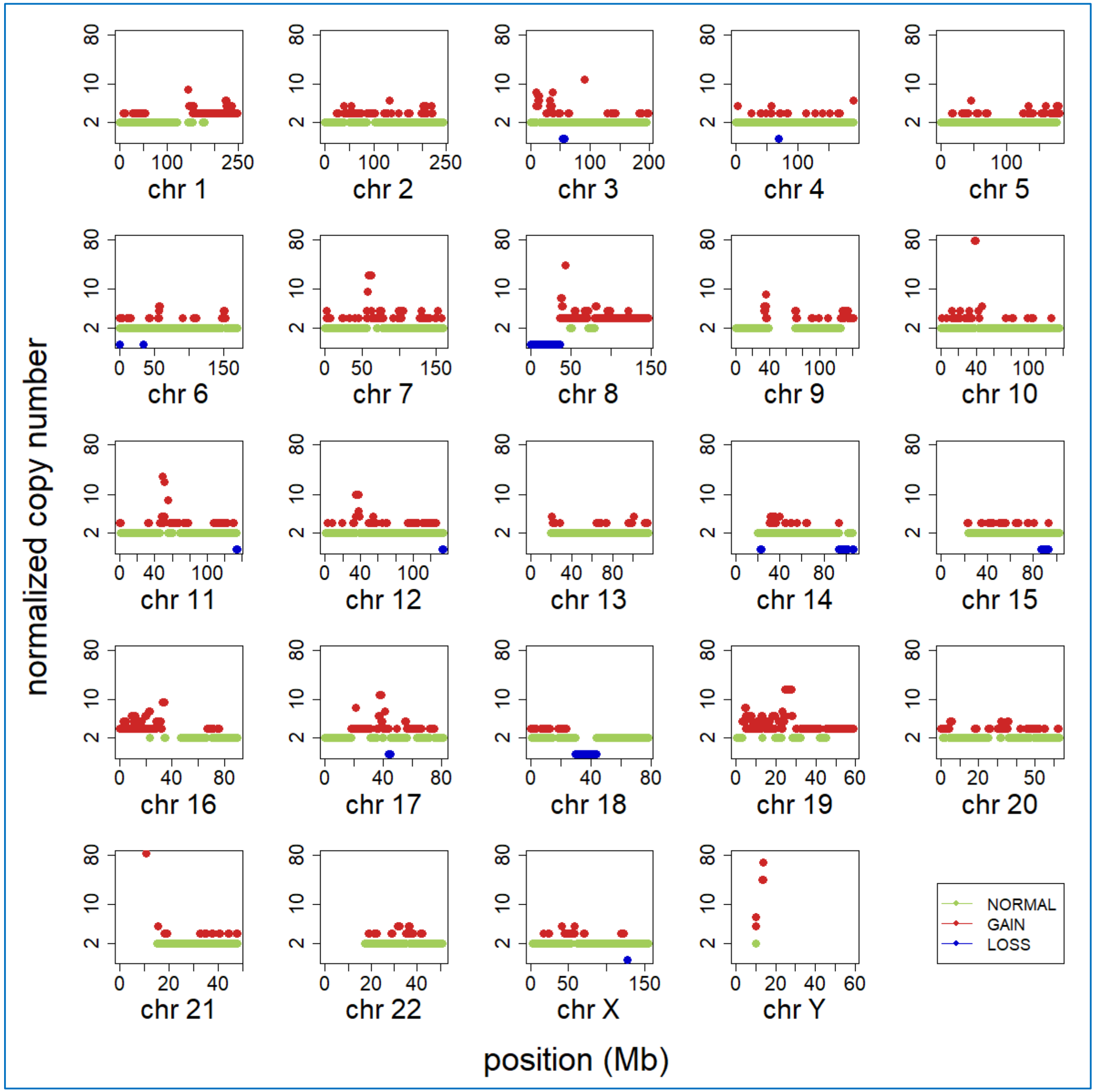

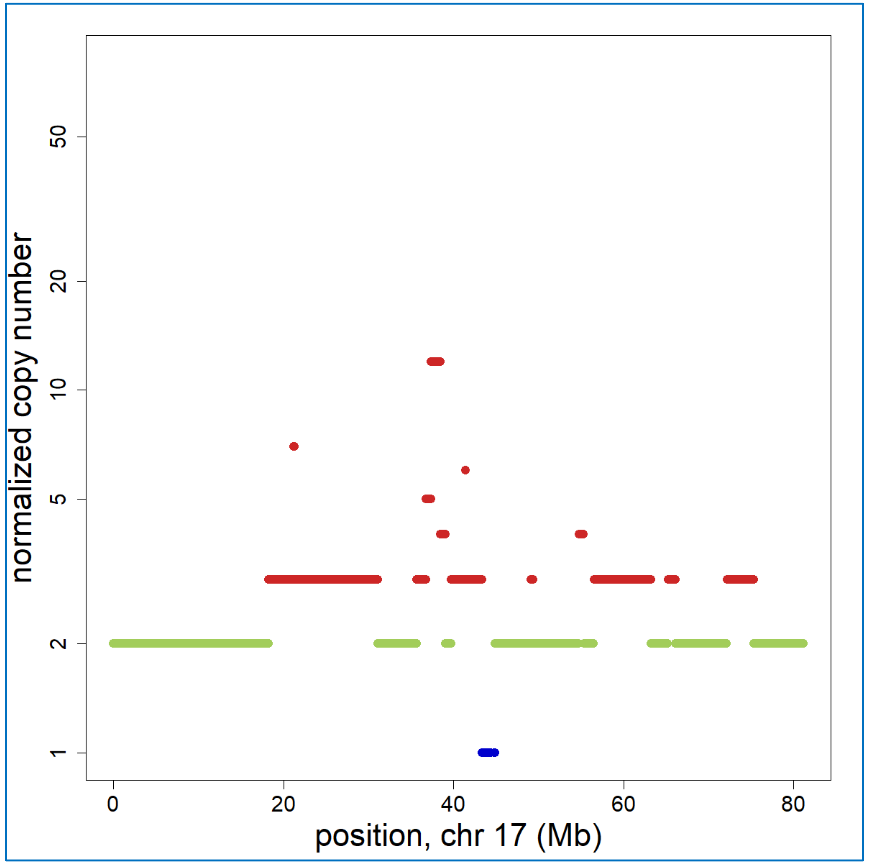

Control-FREEC

p-30

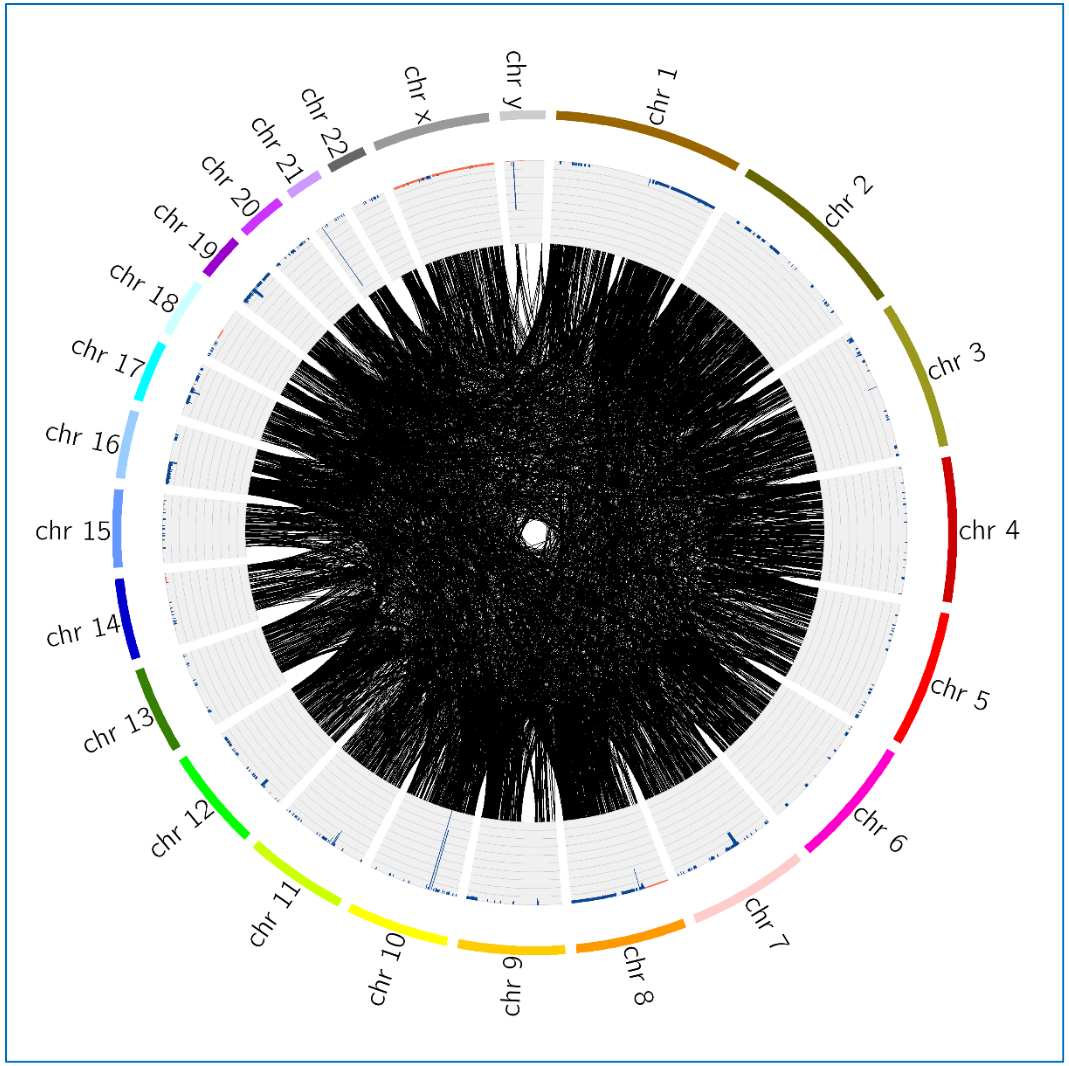

BreakDancer + Control-FREEC

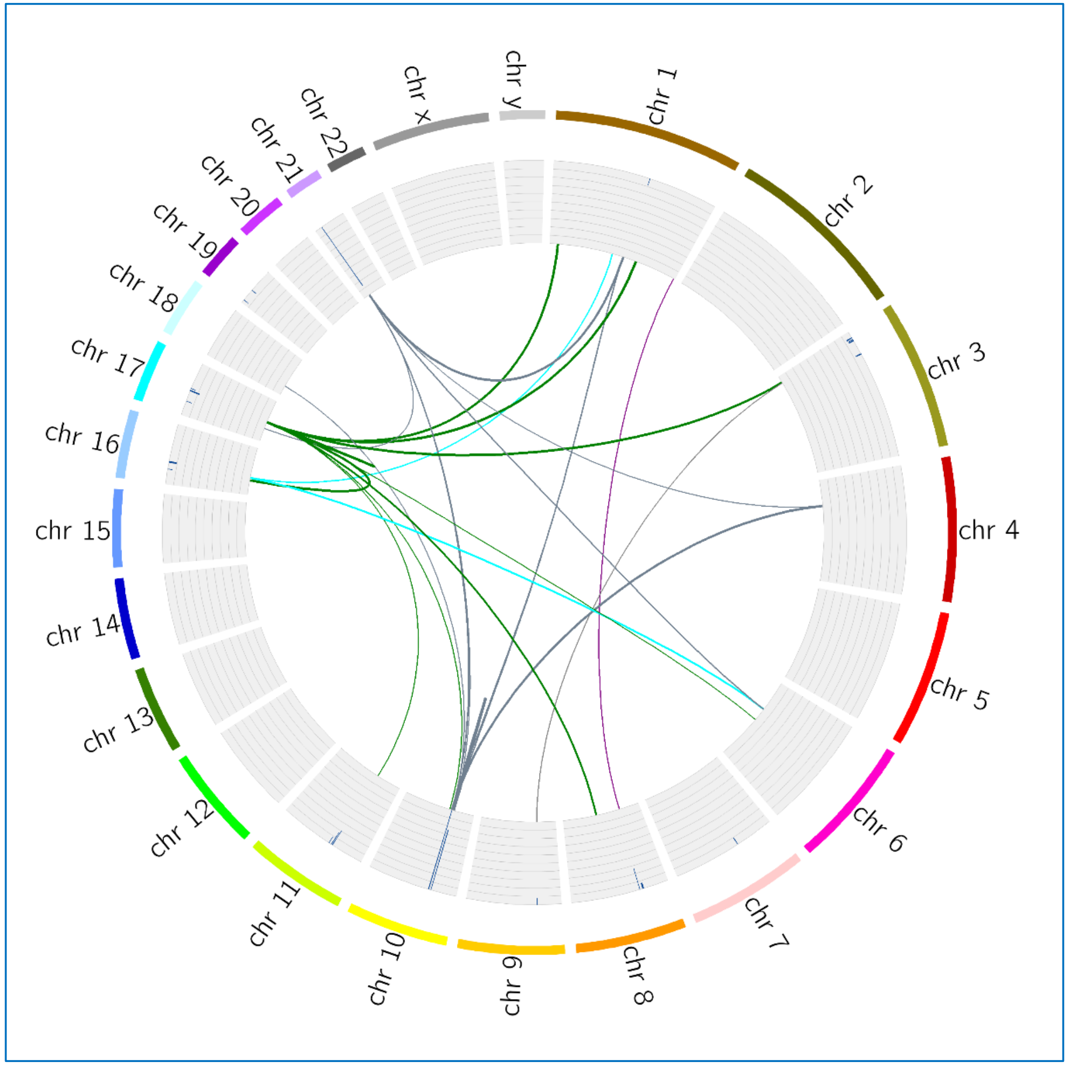

FAST – Whole Genome

p-30

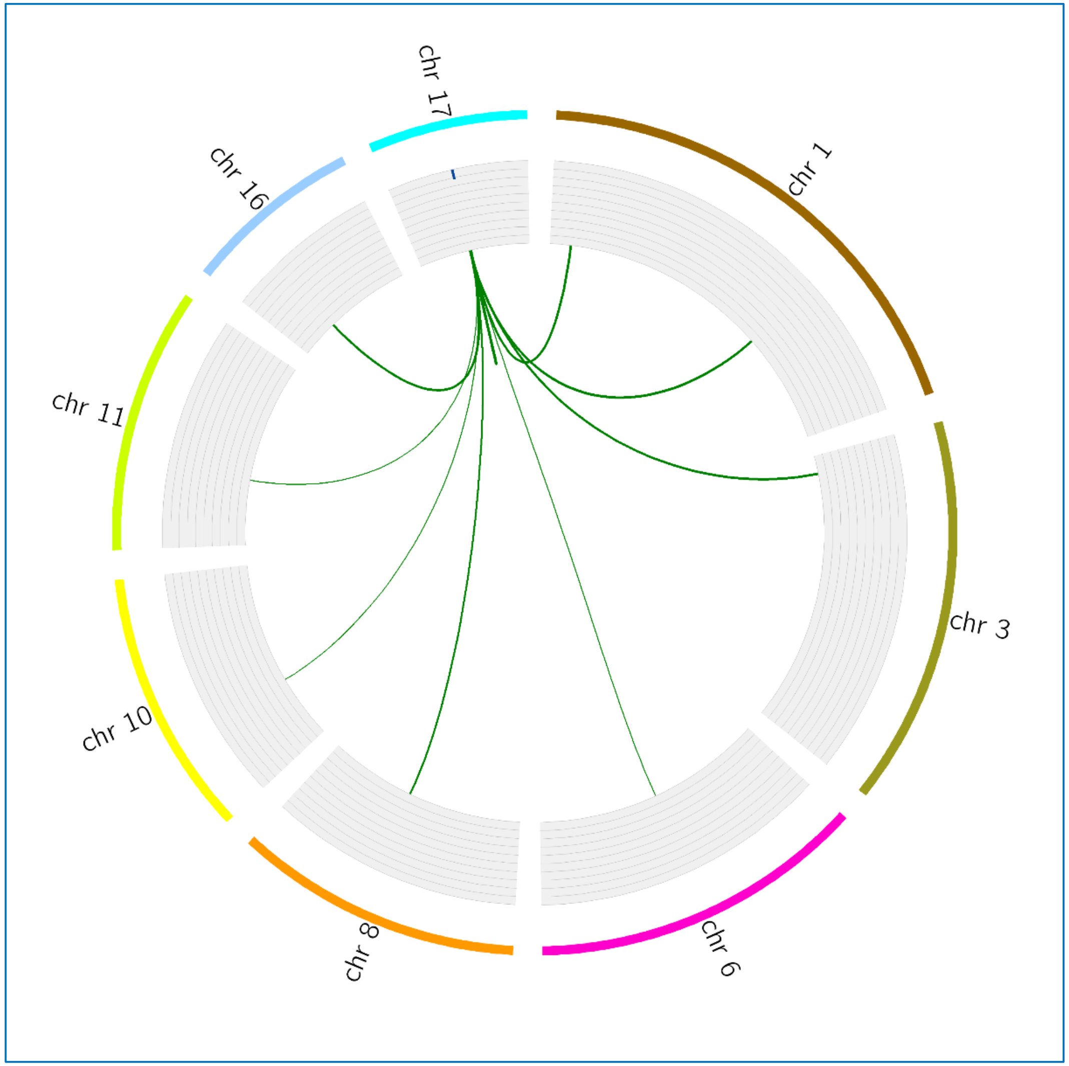

FAST – ERBB2 amplicon

p-33

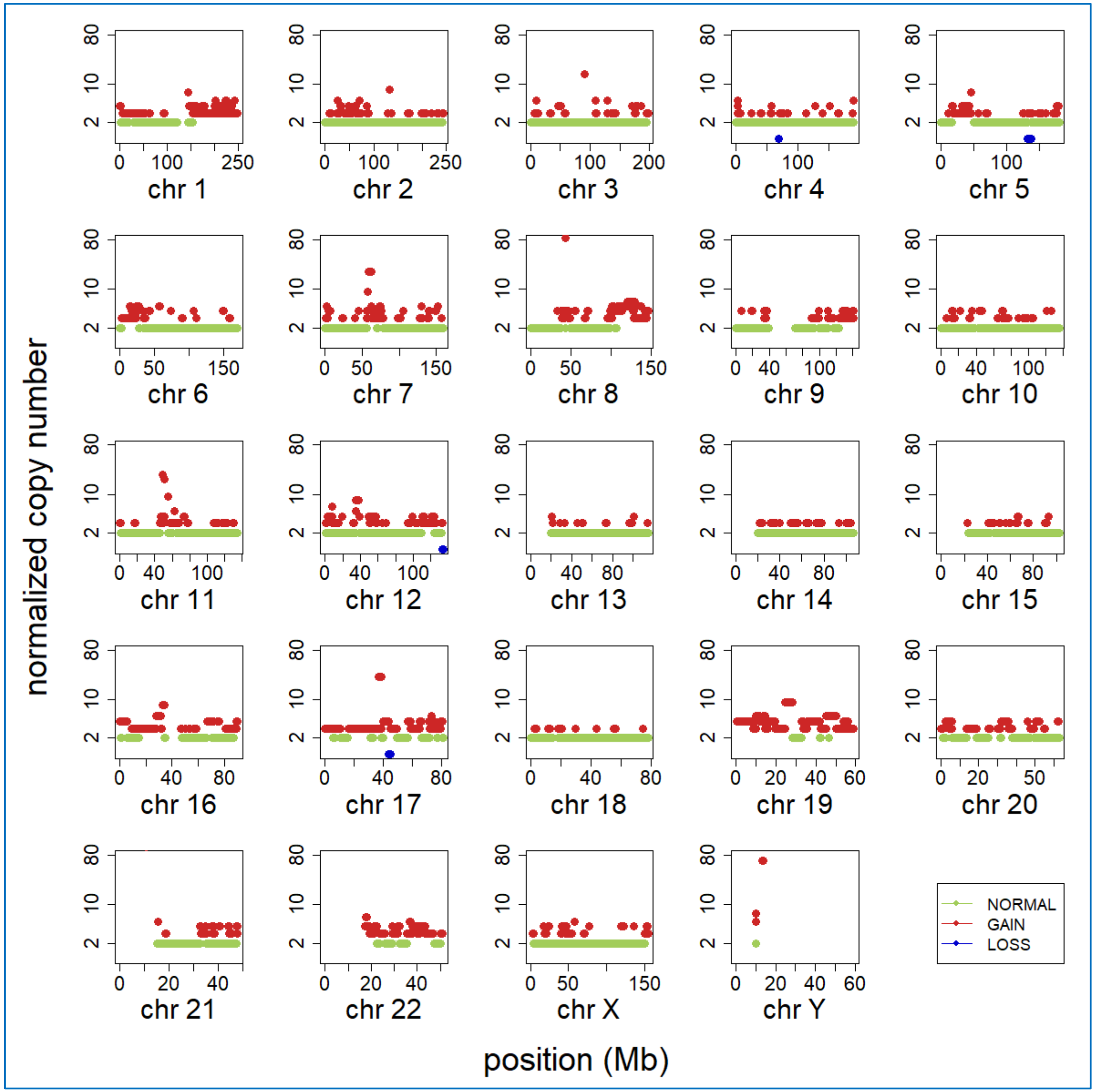

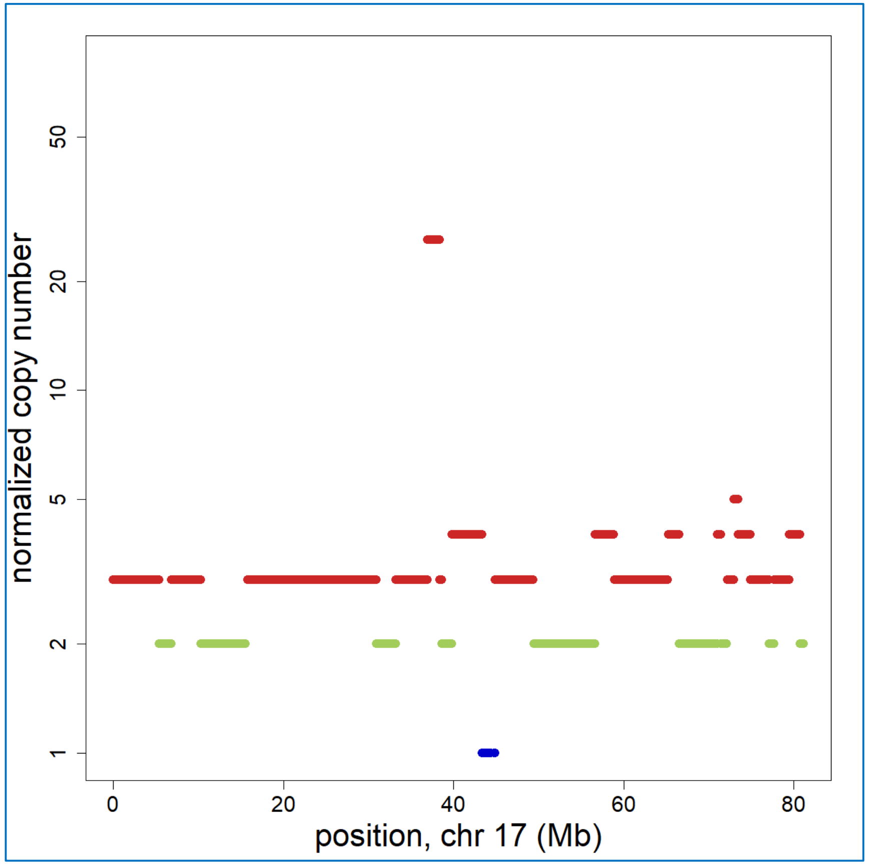

Control-FREEC

p-33

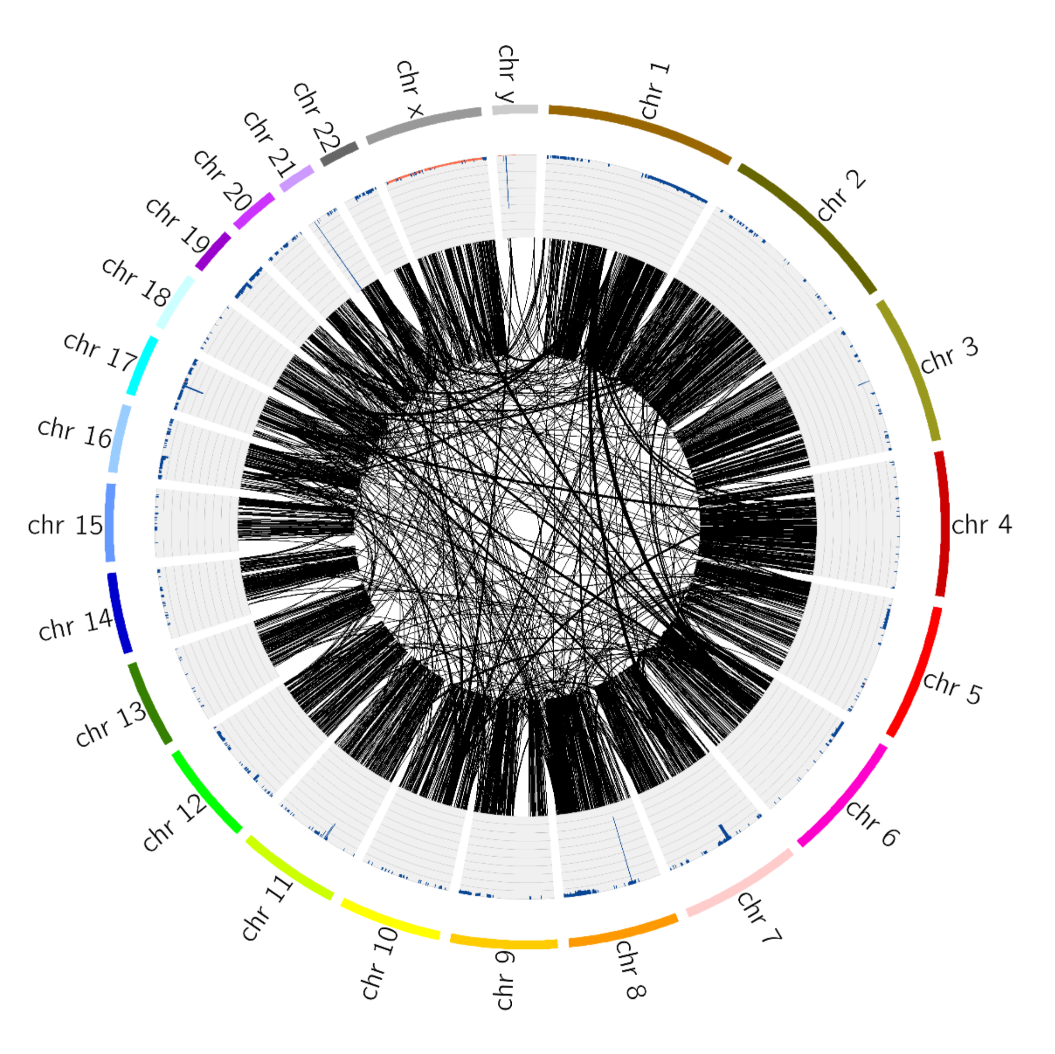

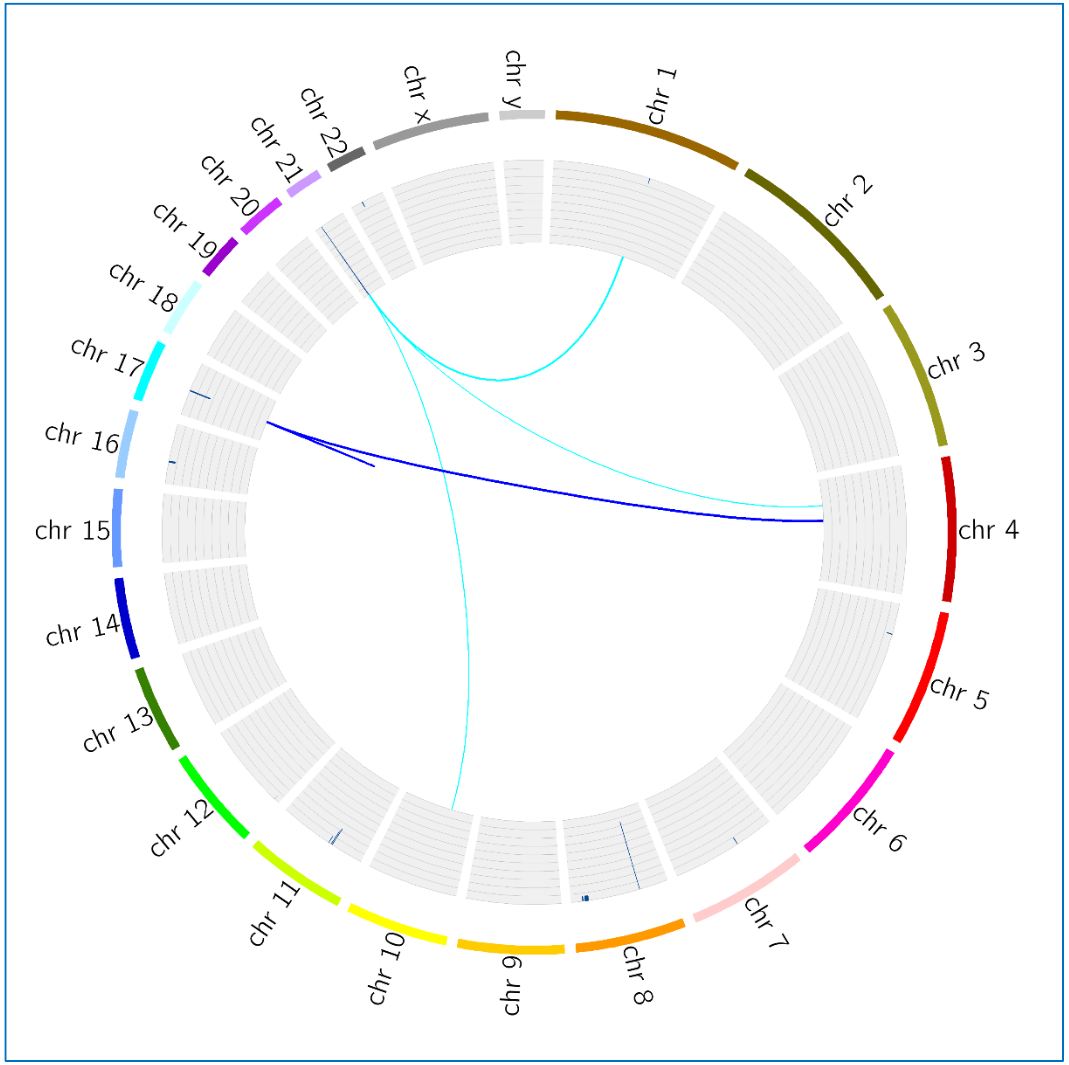

BreakDancer + Control-FREEC

FAST – Whole Genome

p-33

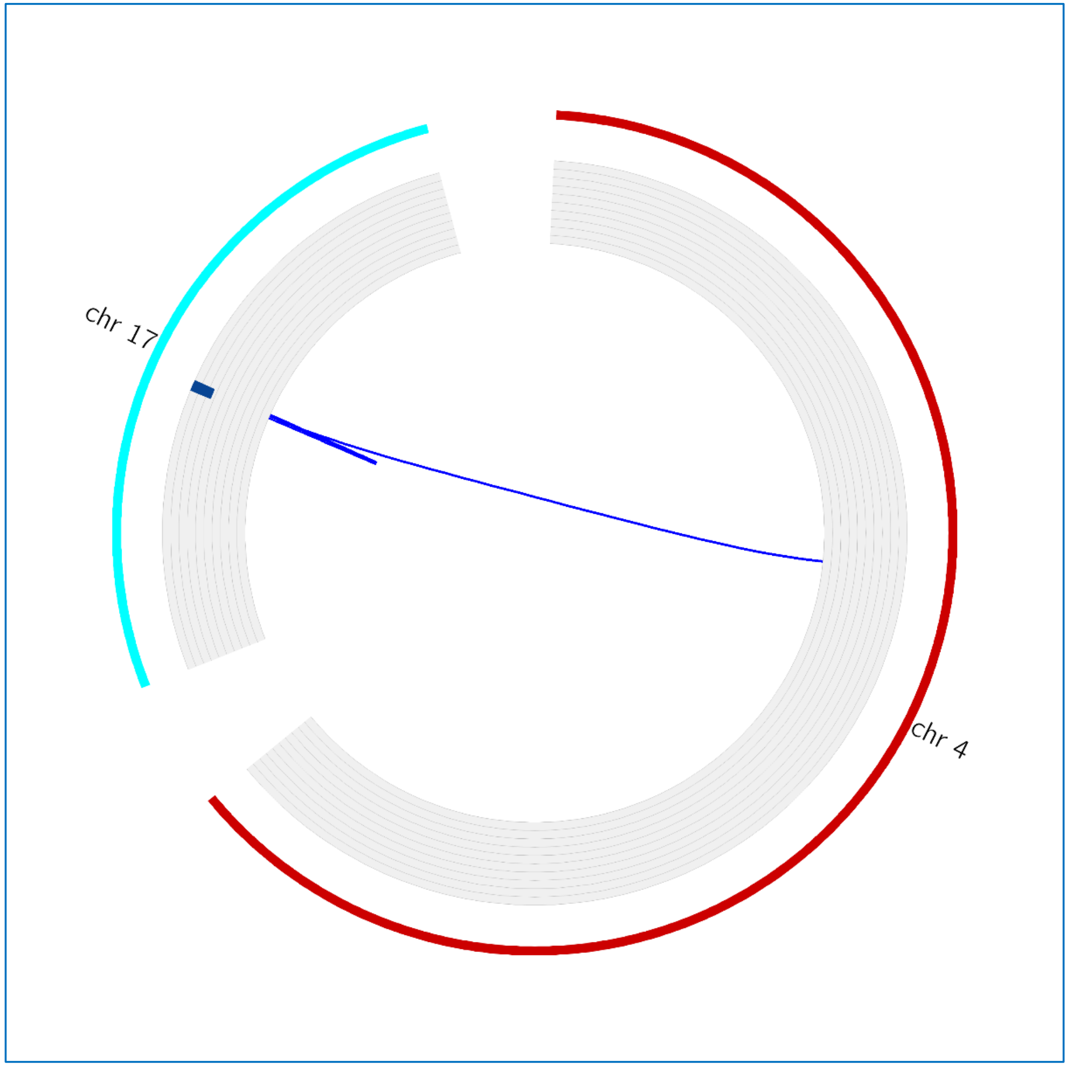

FAST – ERBB2 amplicon

p-102

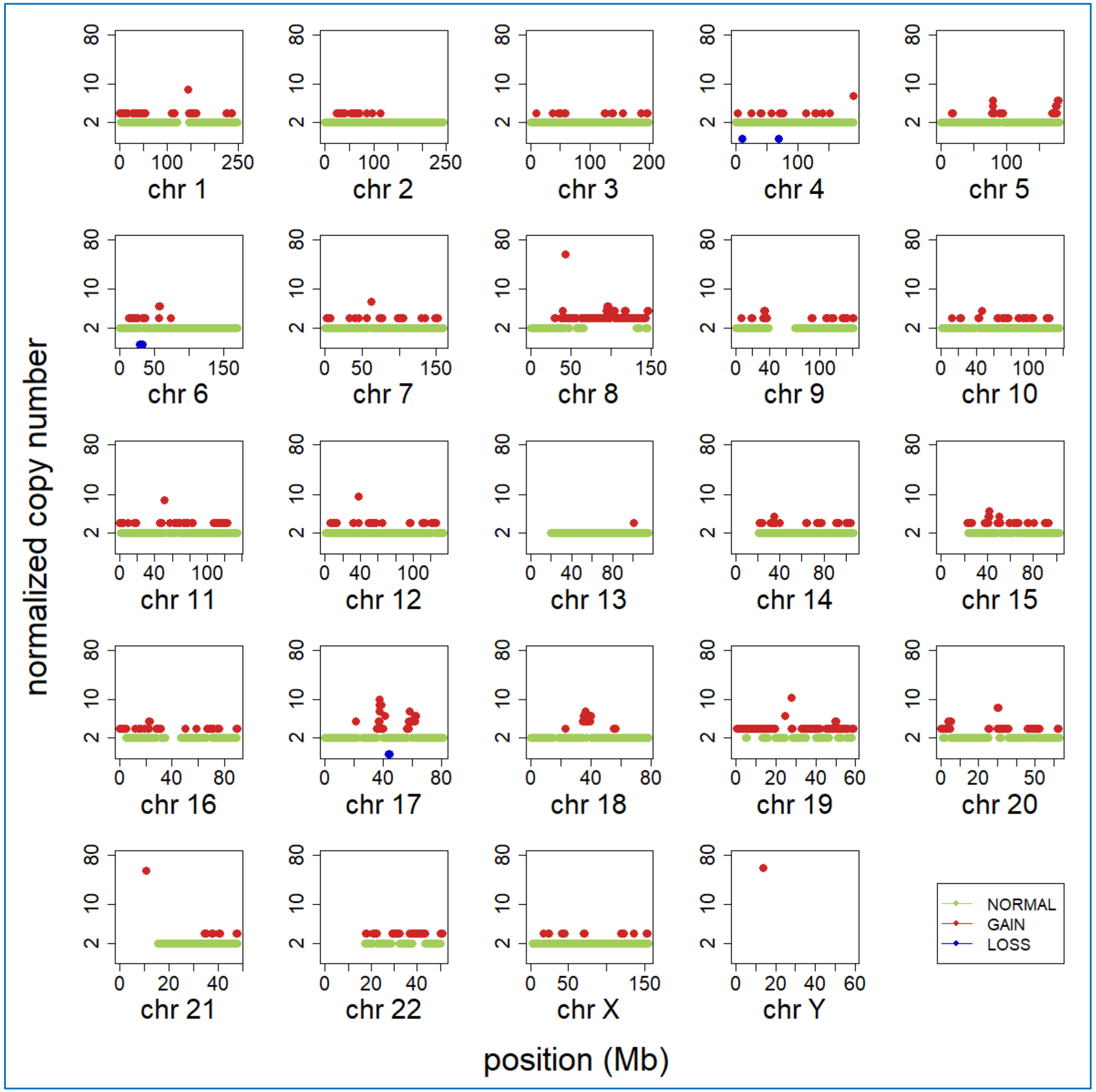

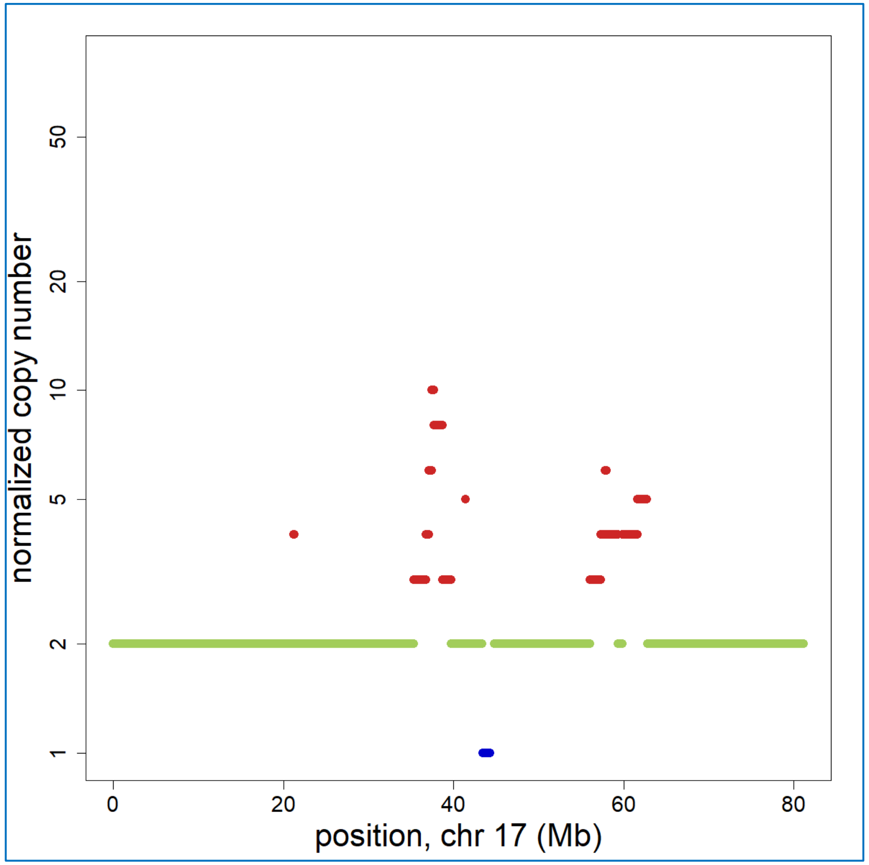

Control-FREEC

p-102

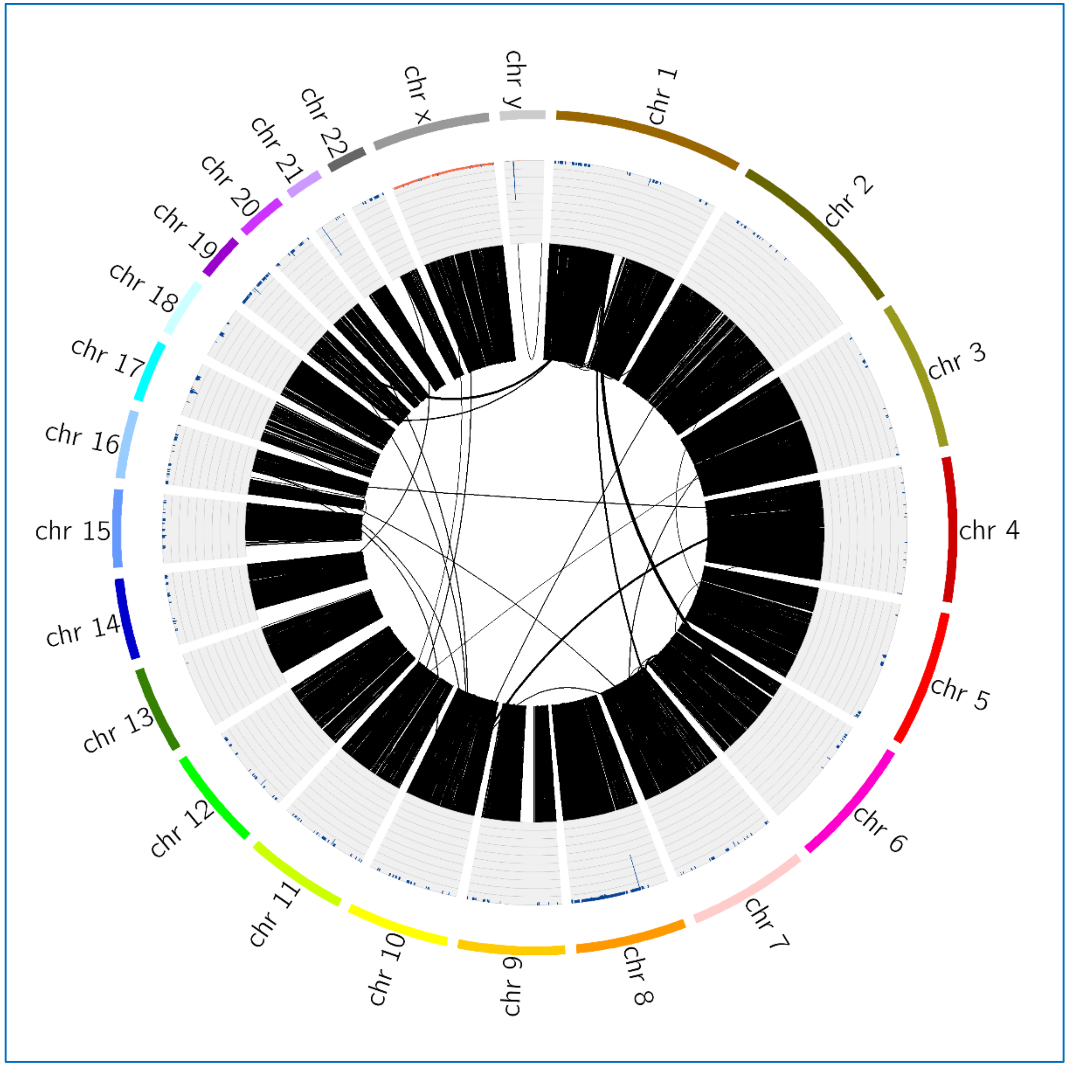

BreakDancer + Control-FREEC

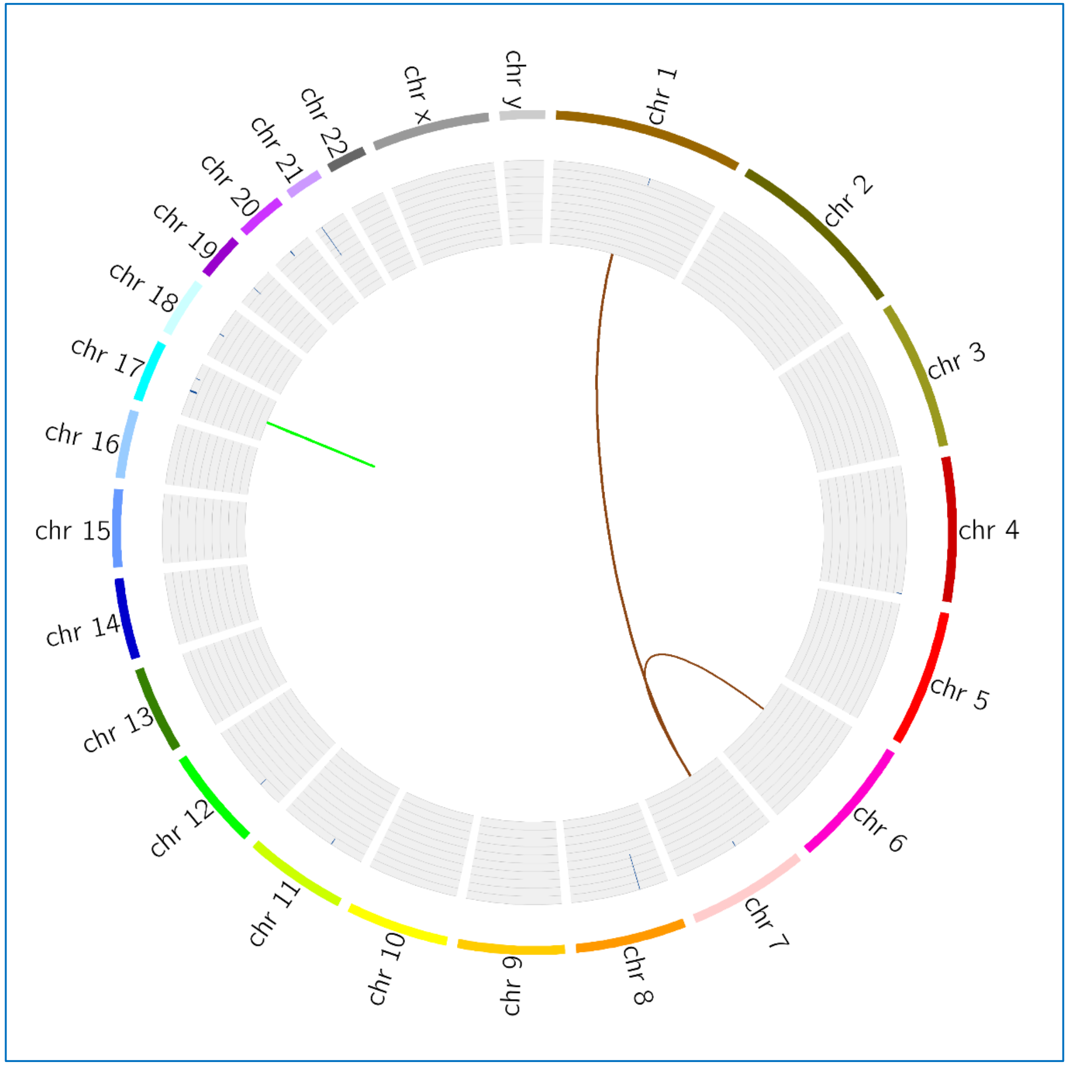

FAST – Whole Genome

p-102

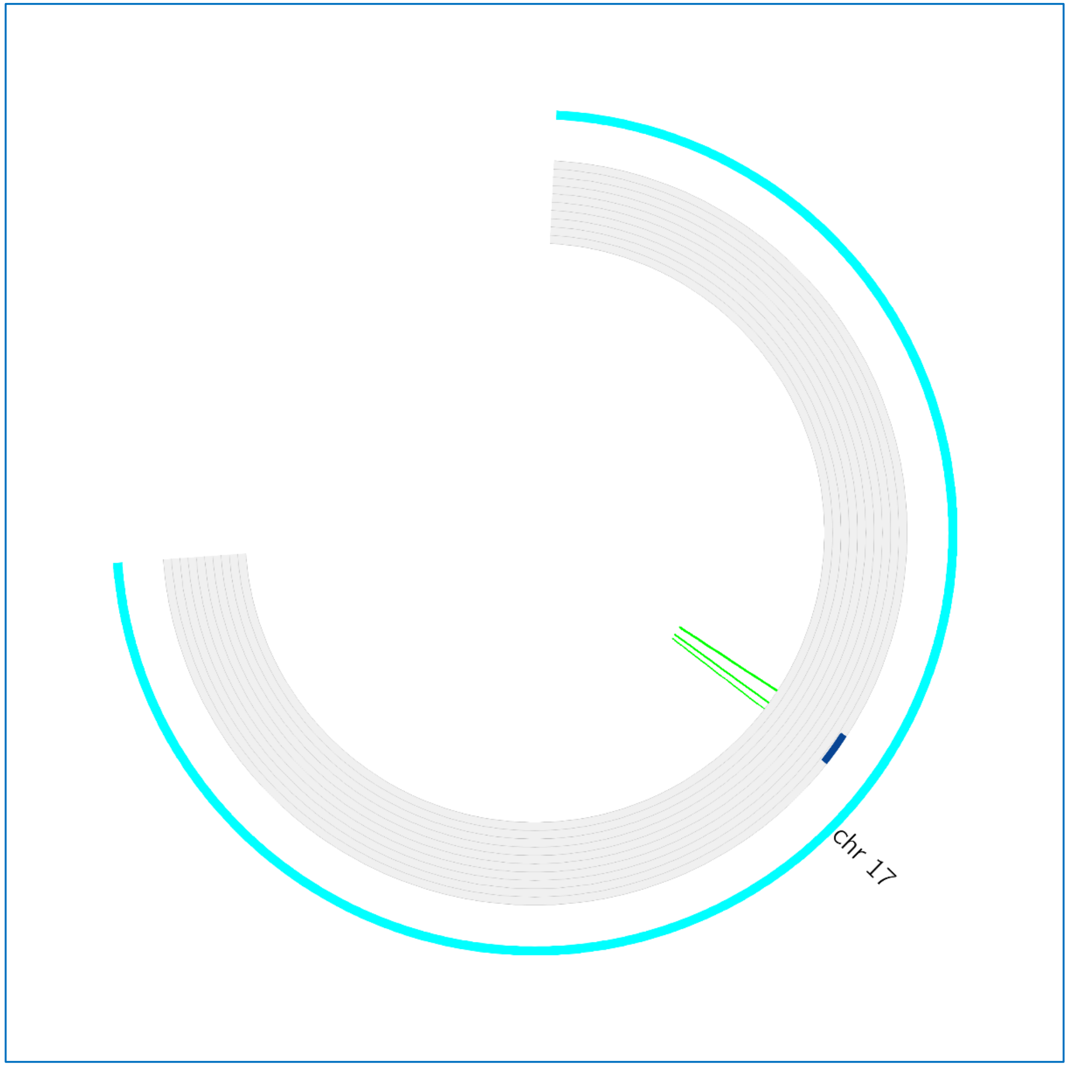

FAST – ERBB2 amplicon

p-103

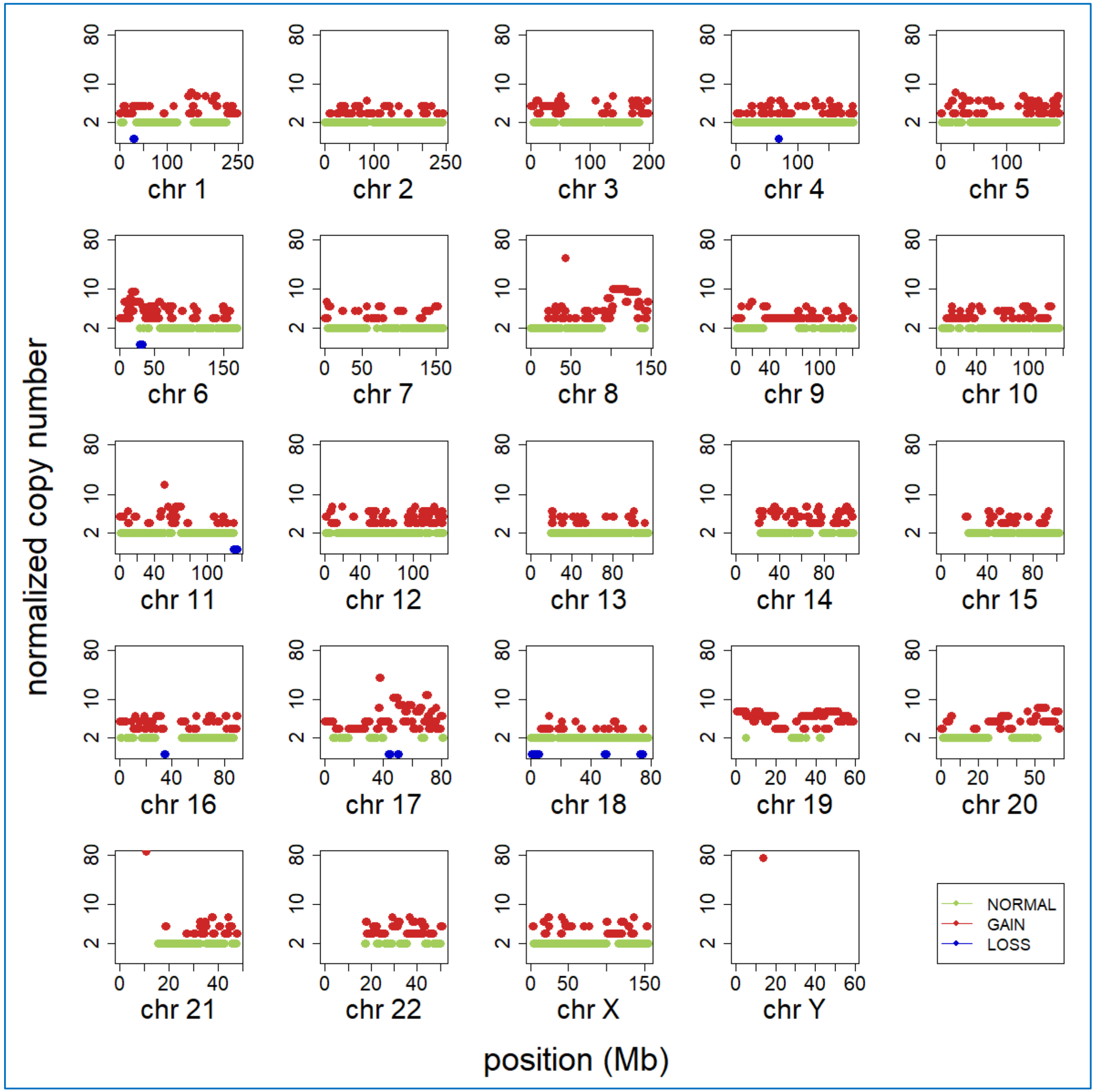

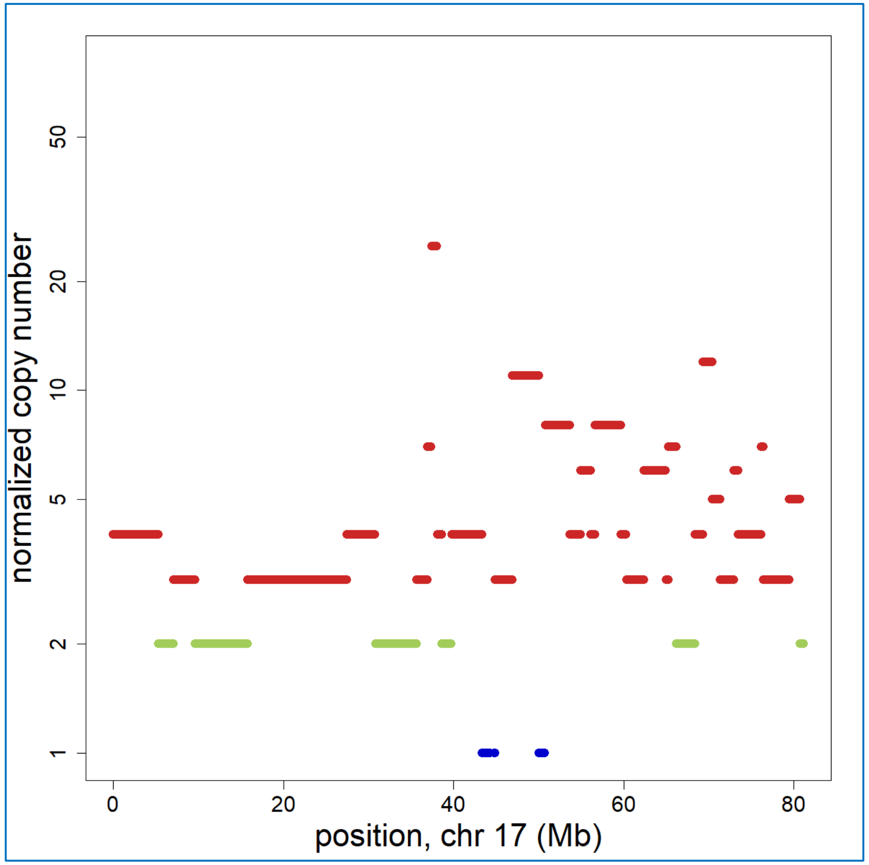

Control-FREEC

p-103

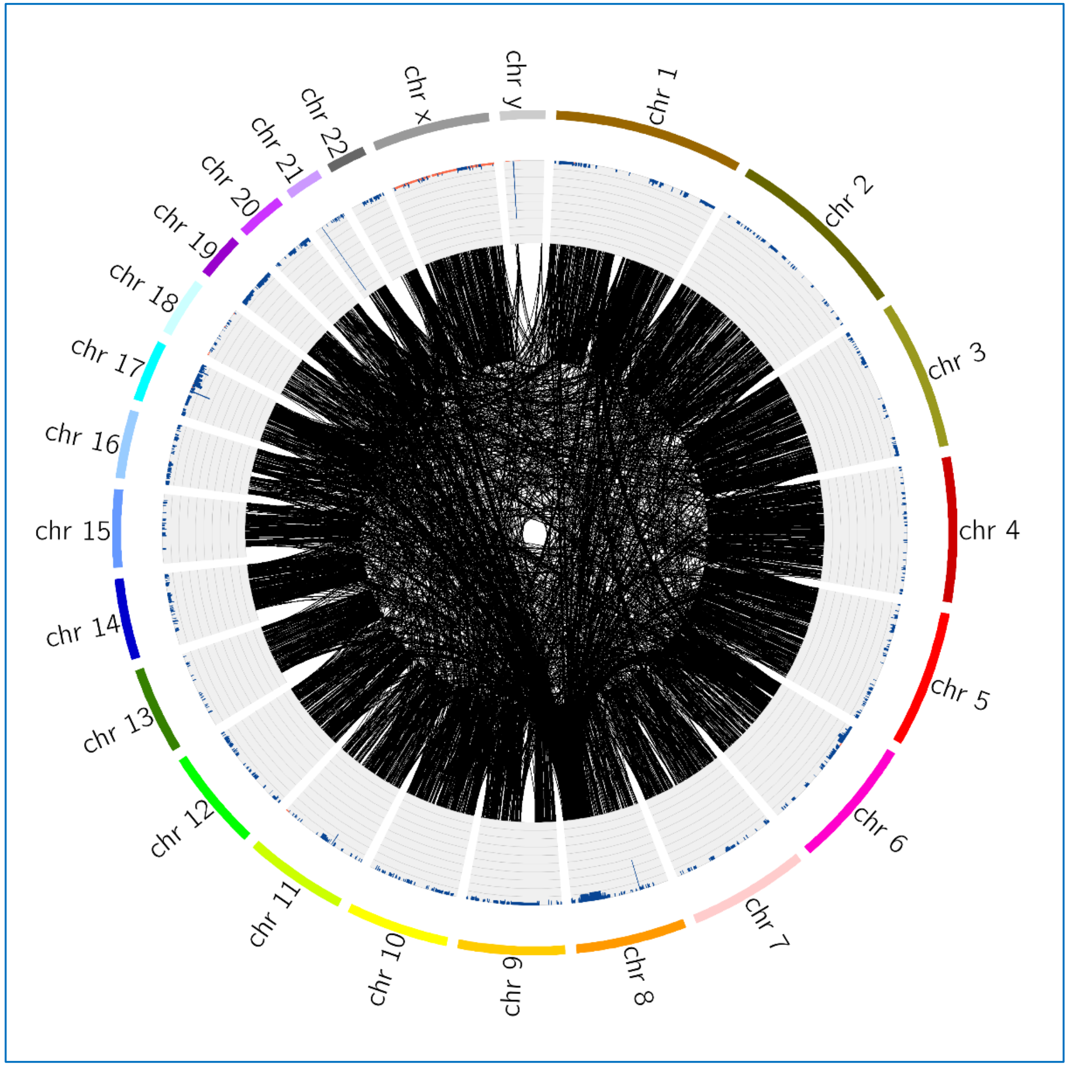

BreakDancer + Control-FREEC

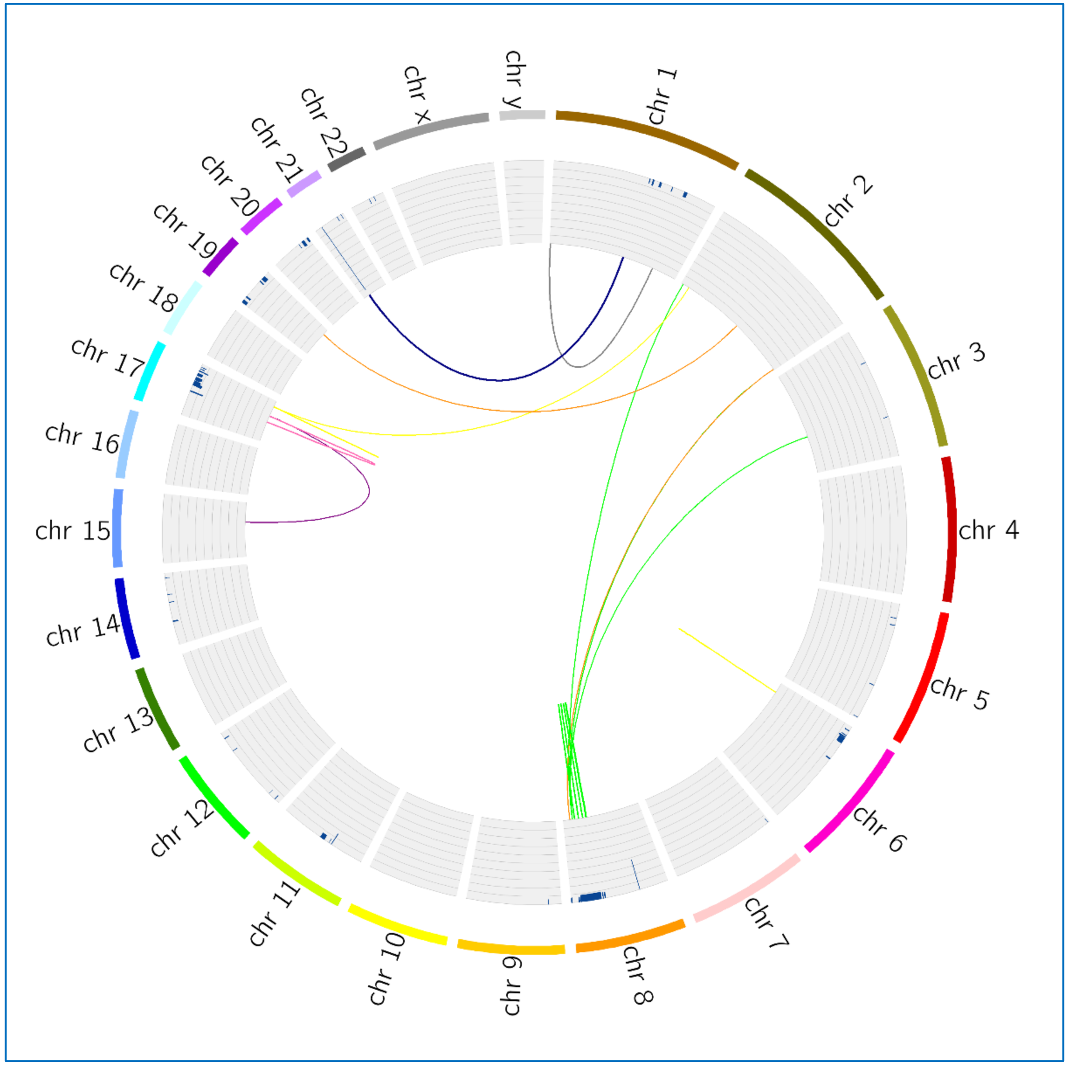

FAST – Whole Genome

p-103

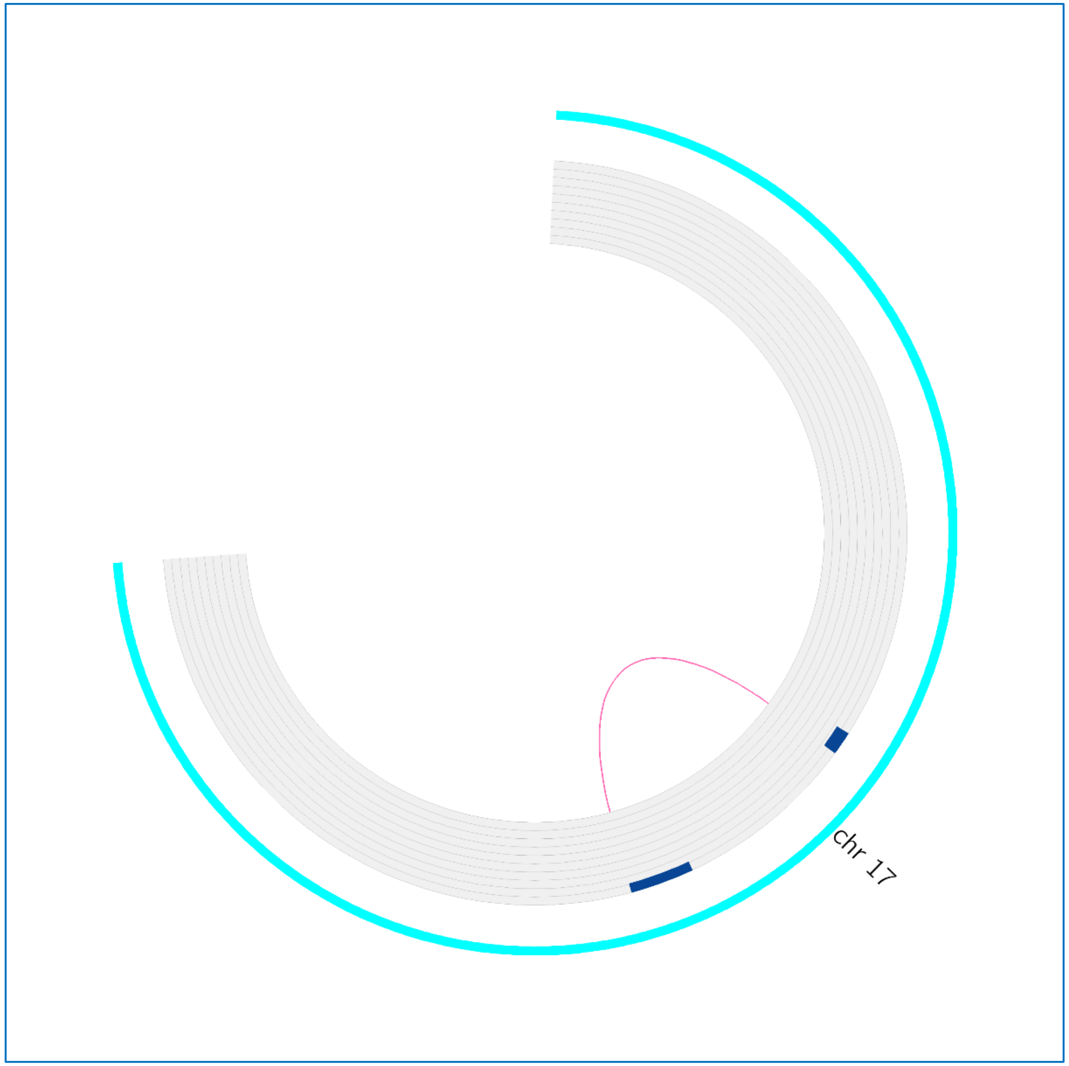

FAST – ERBB2 amplicon

p-104

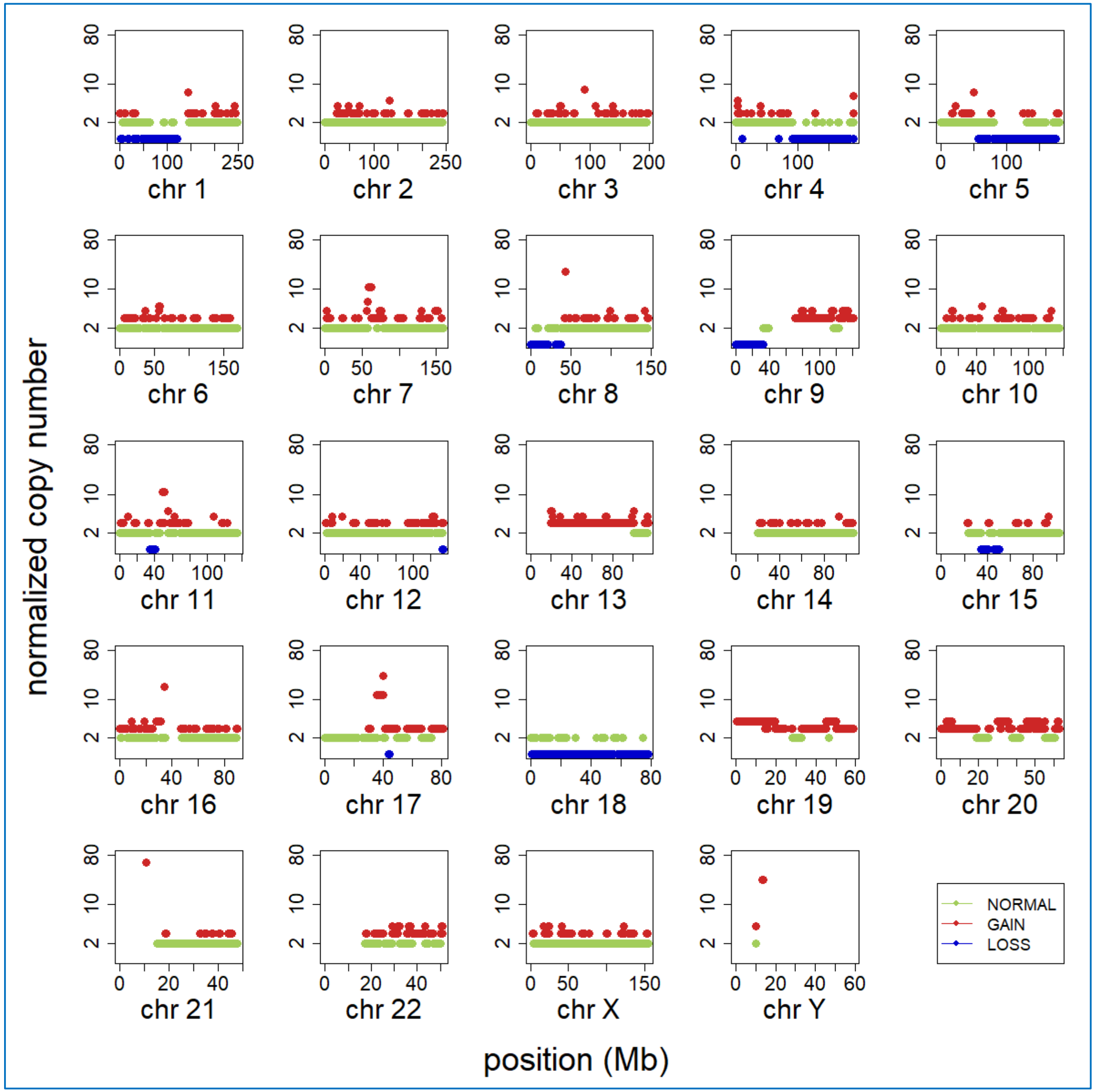

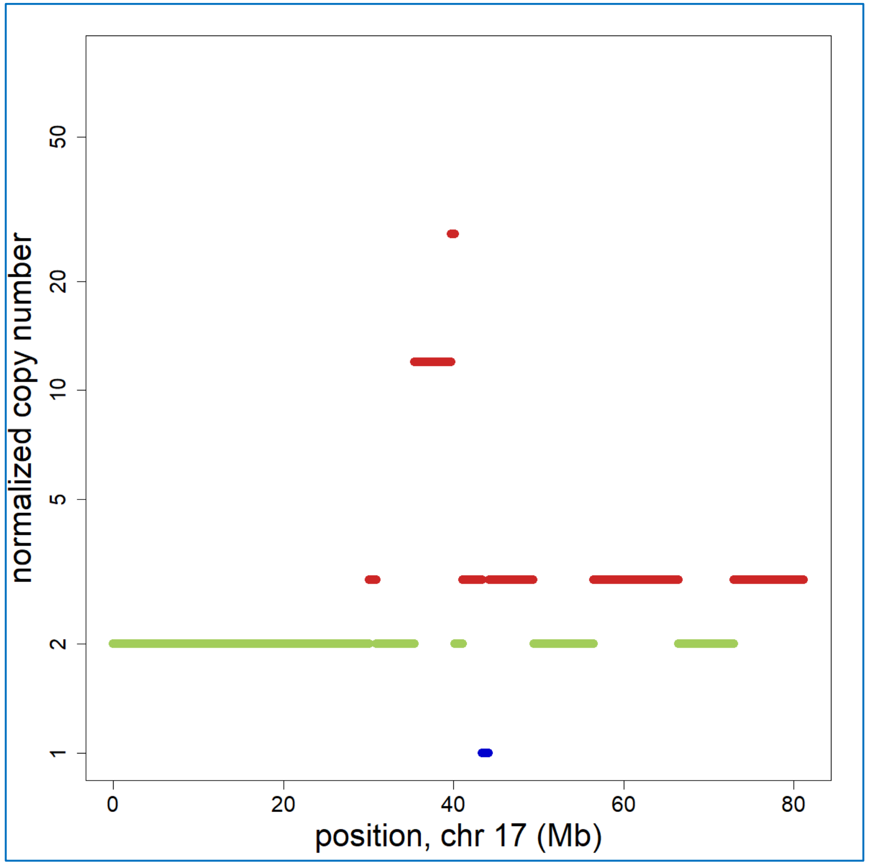

Control-FREEC

p-104

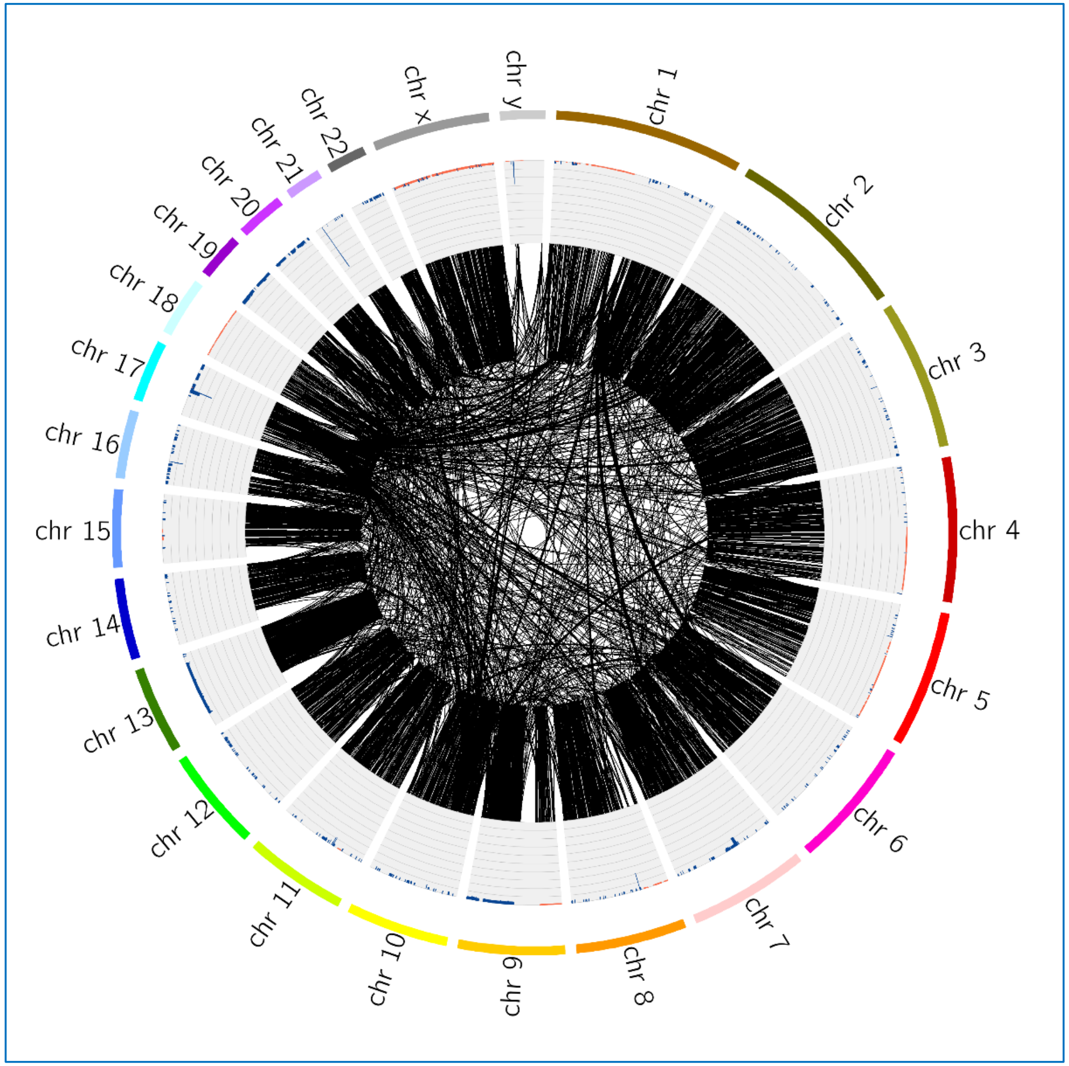

BreakDancer + Control-FREEC

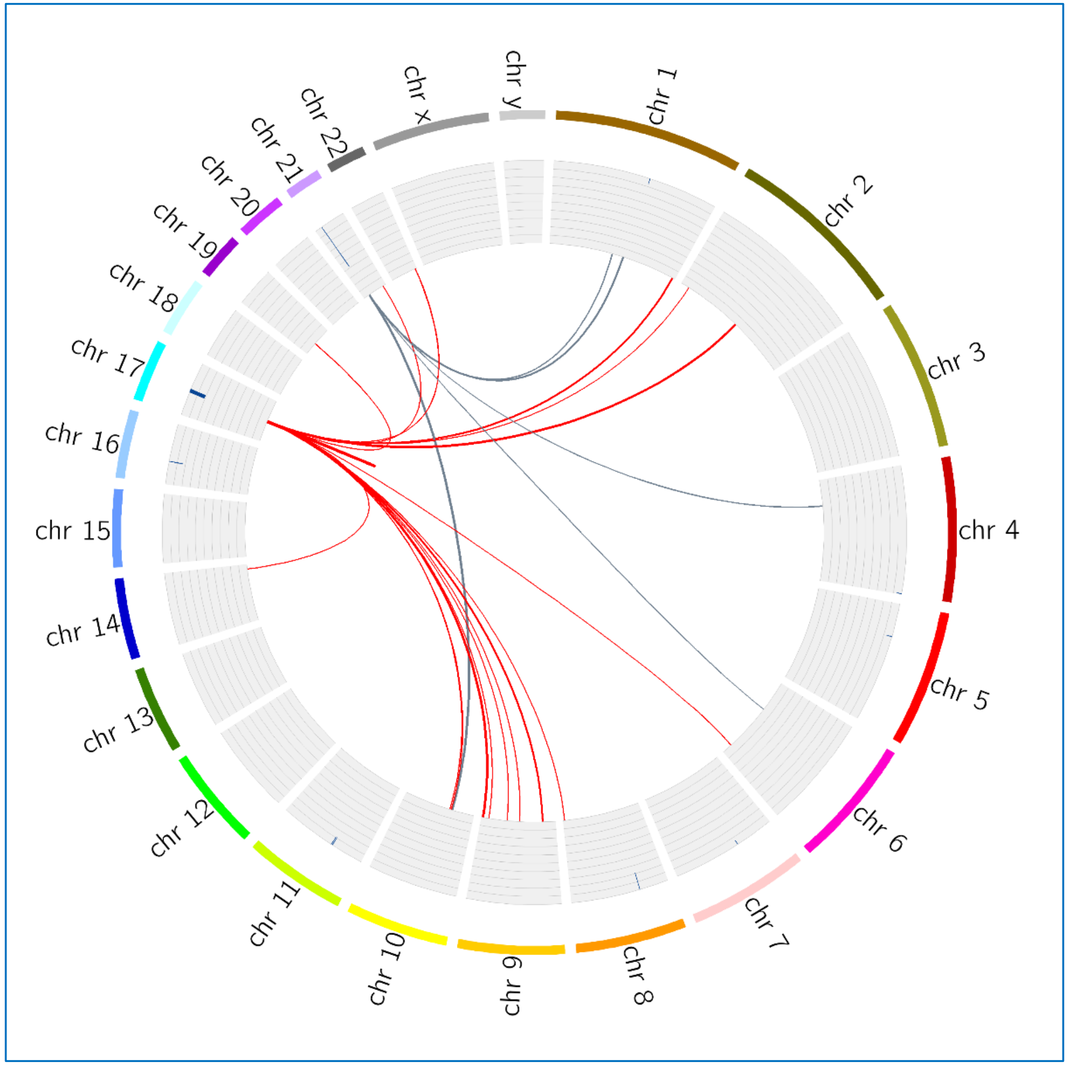

FAST – Whole Genome

p-104

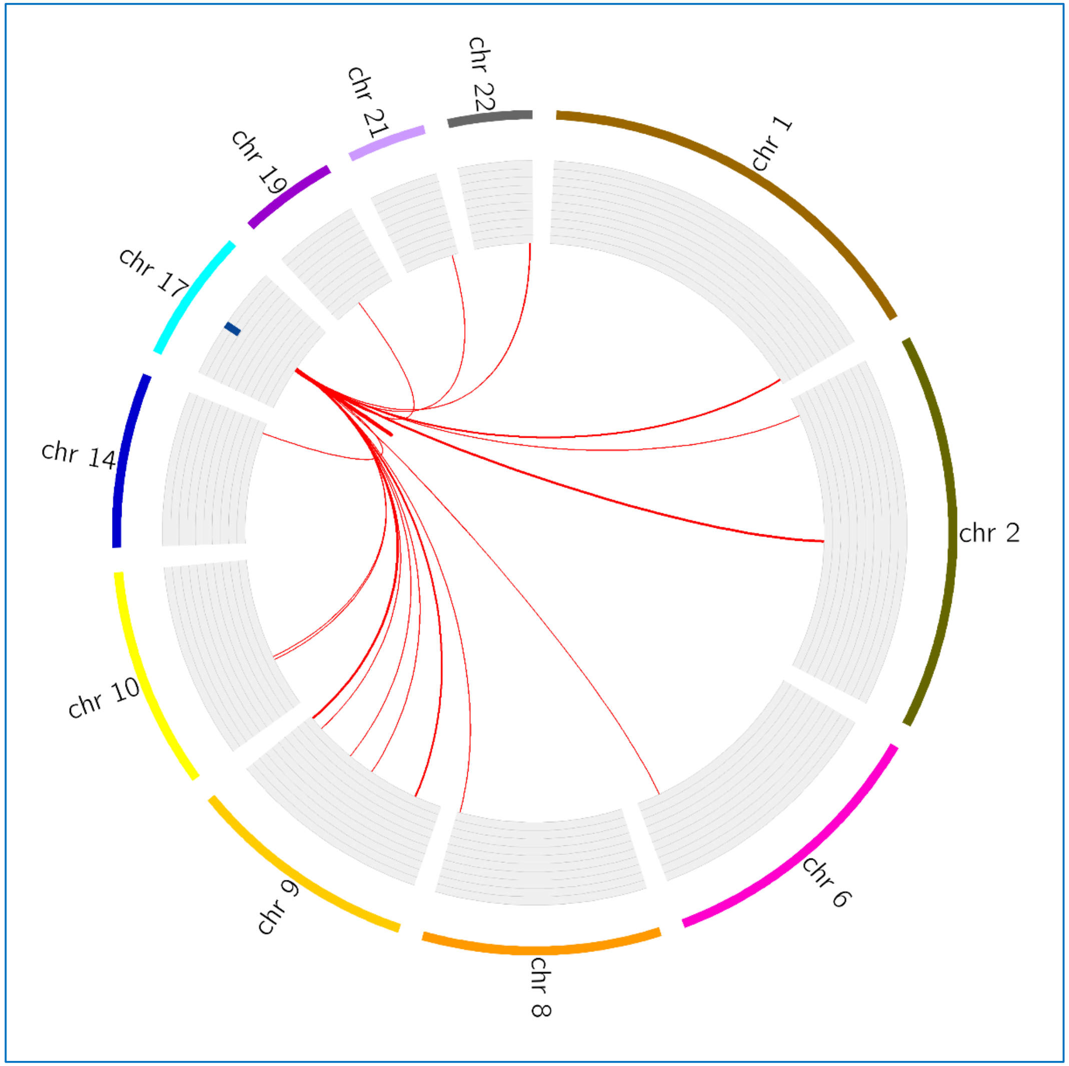

FAST – ERBB2 amplicon

p-106

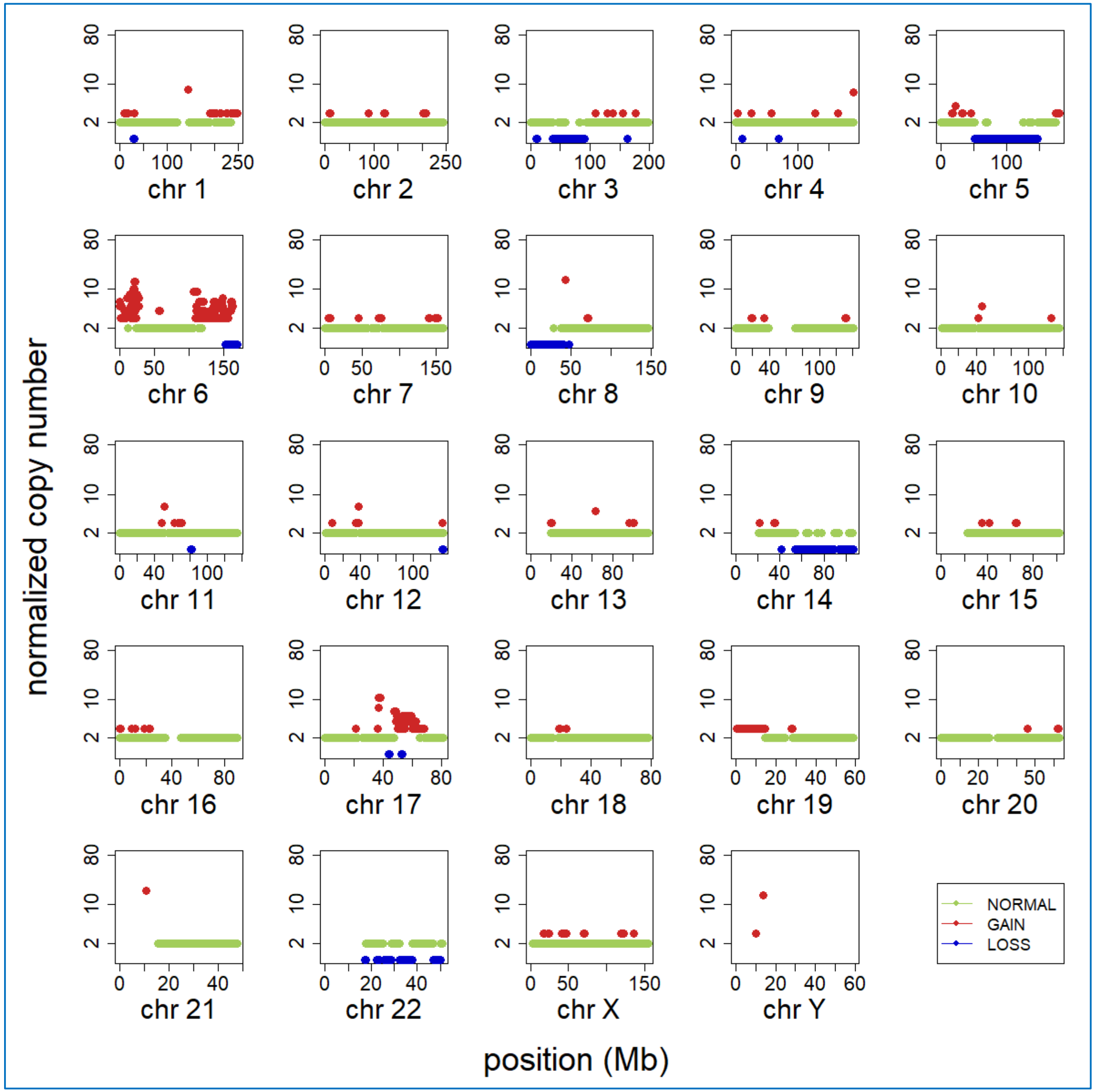

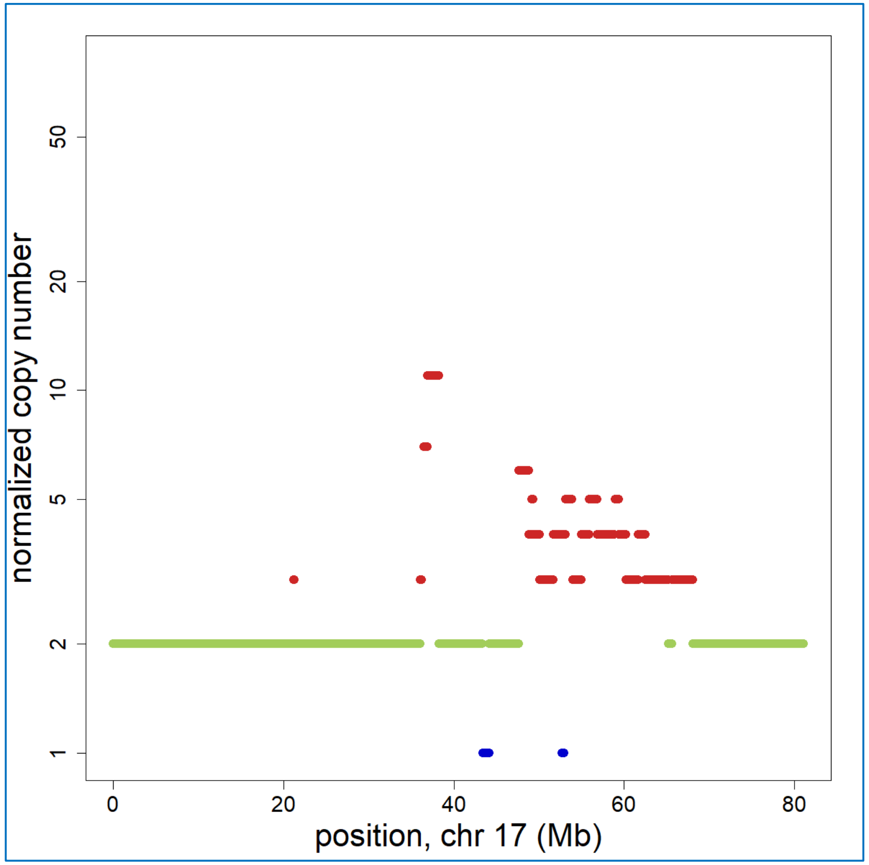

Control-FREEC

p-106

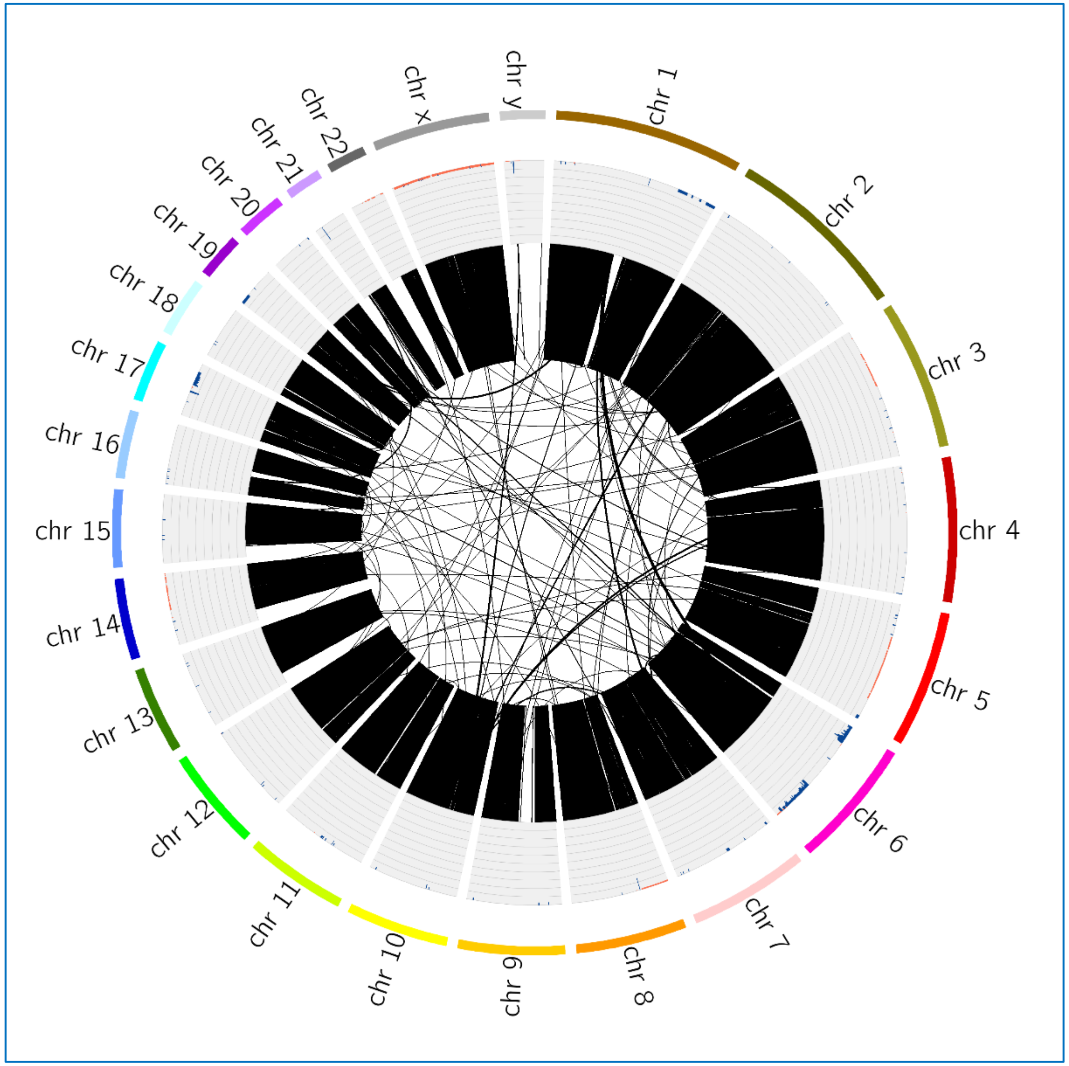

BreakDancer + Control-FREEC

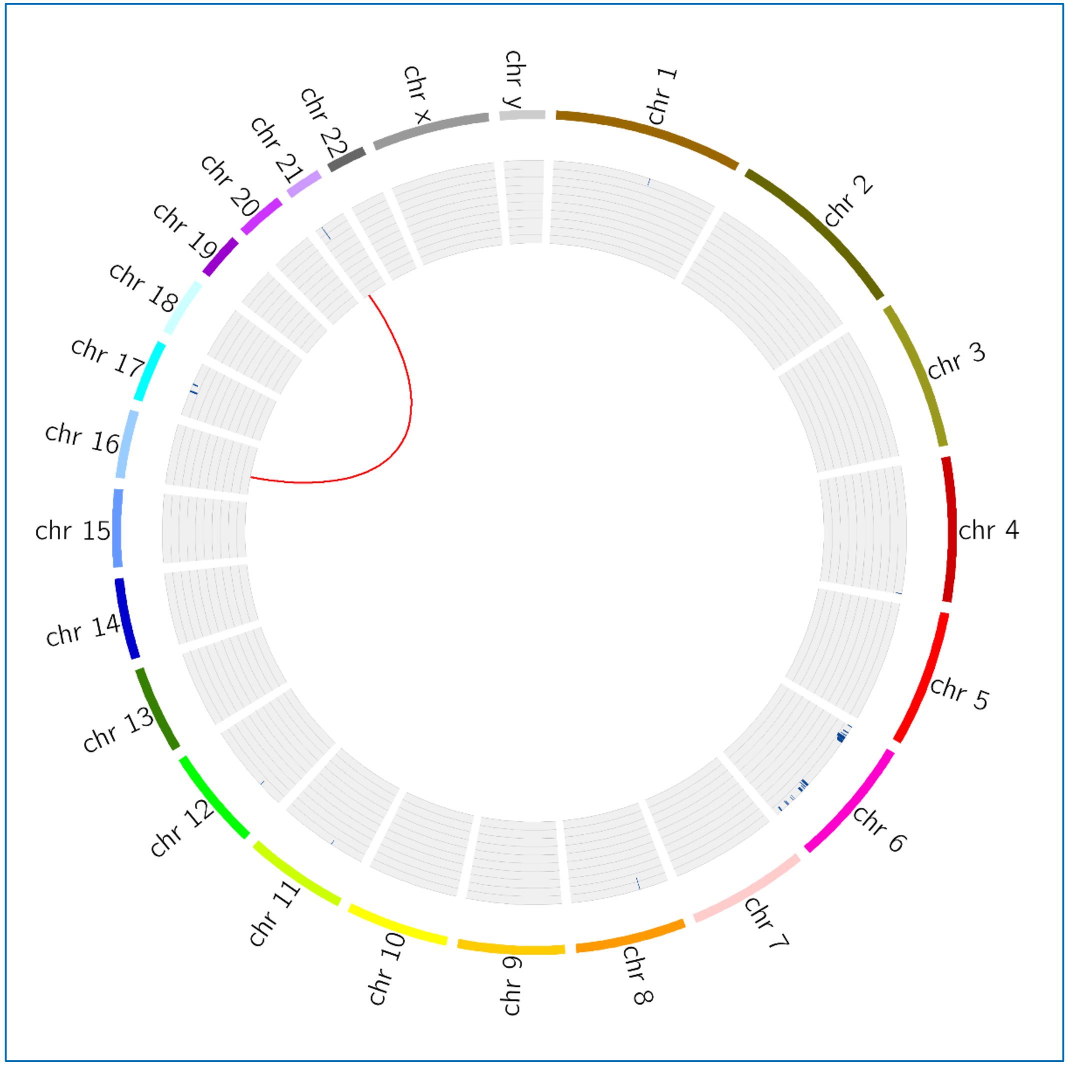

FAST – Whole Genome

p-106

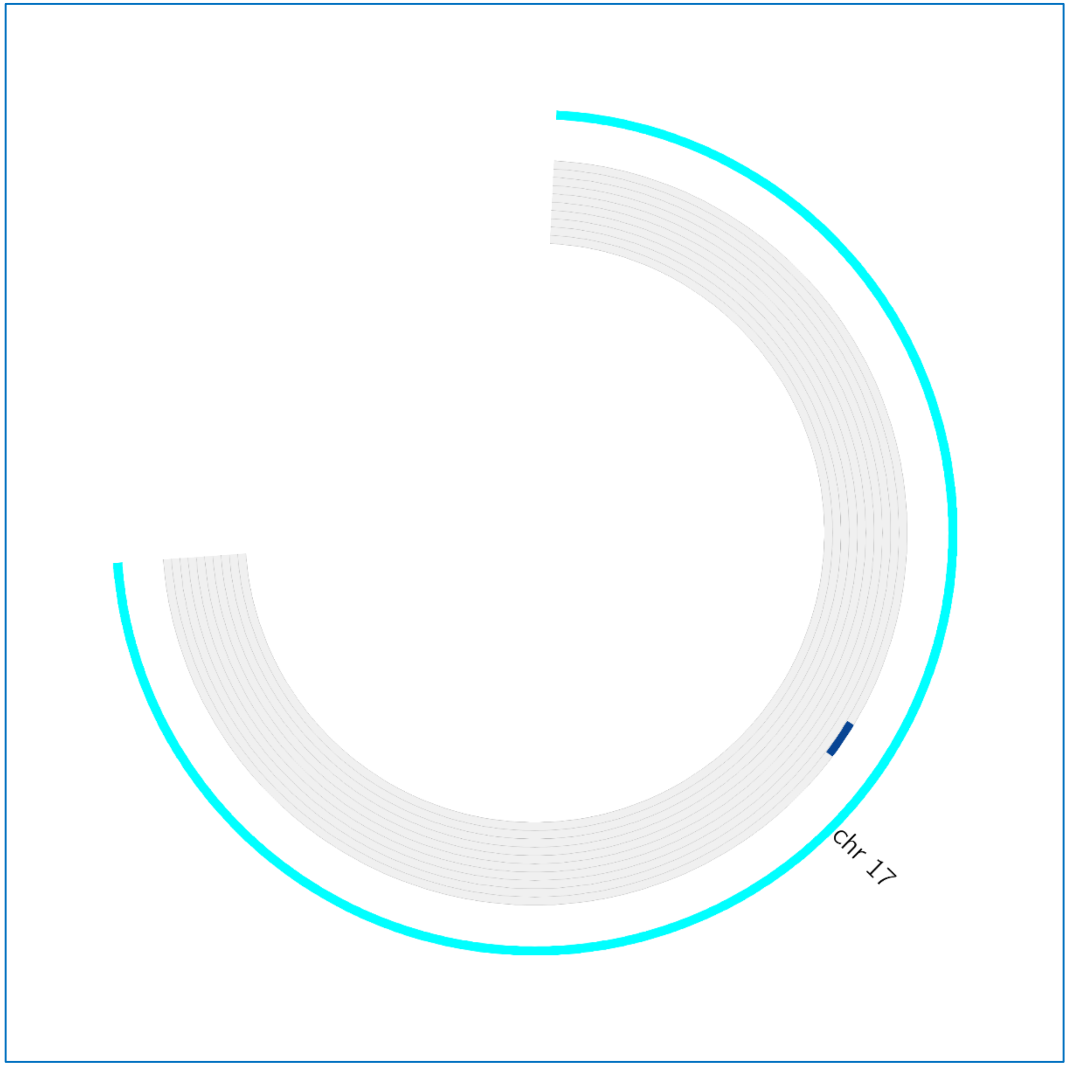

FAST – ERBB2 amplicon

p-108

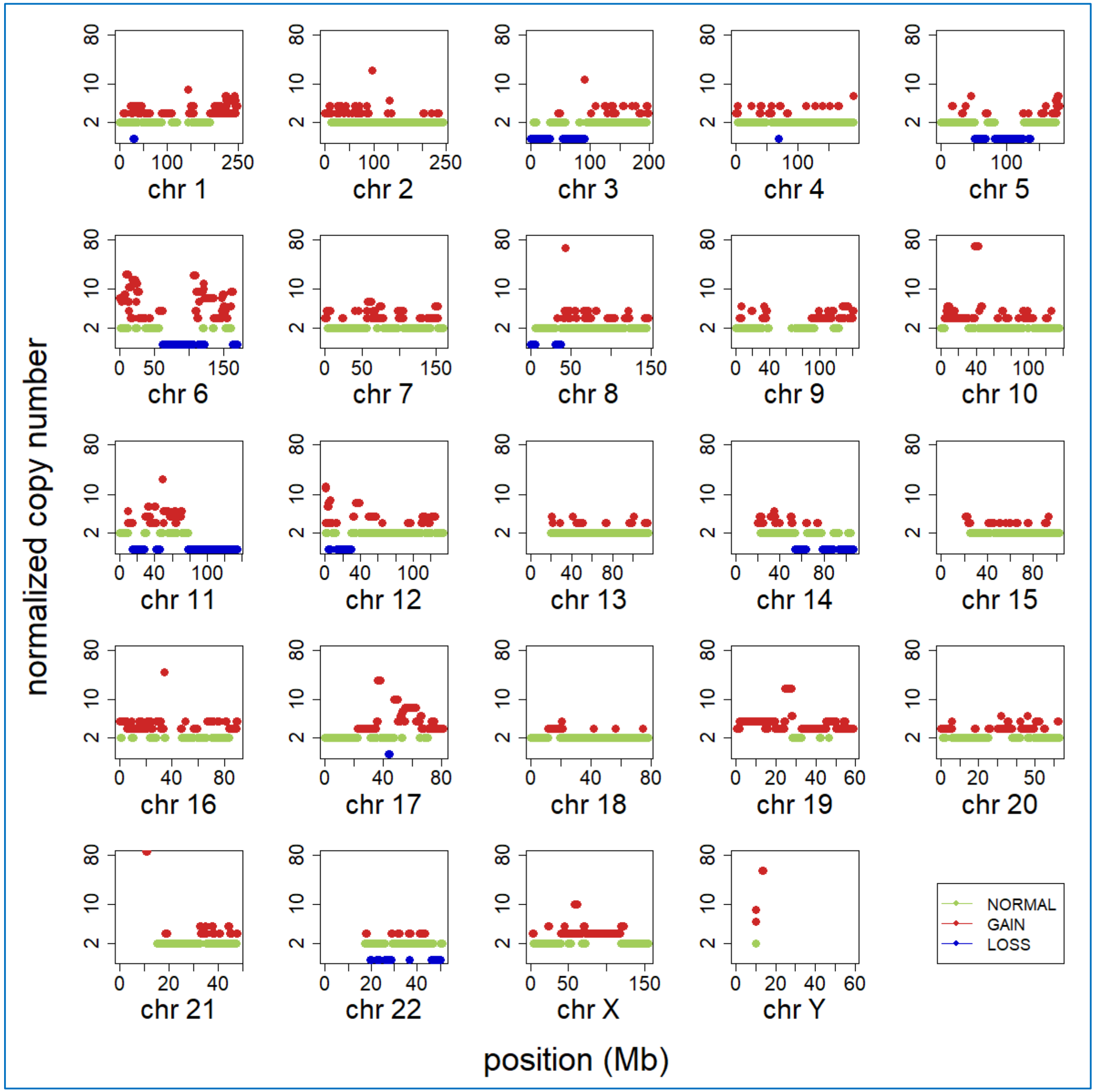

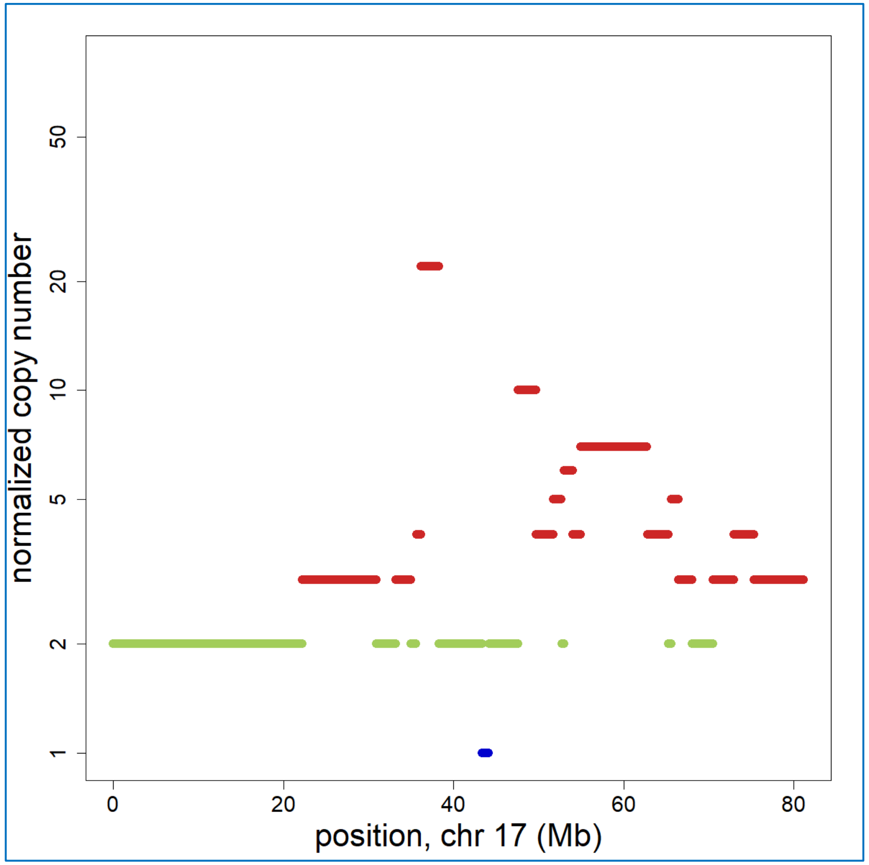

Control-FREEC

p-108

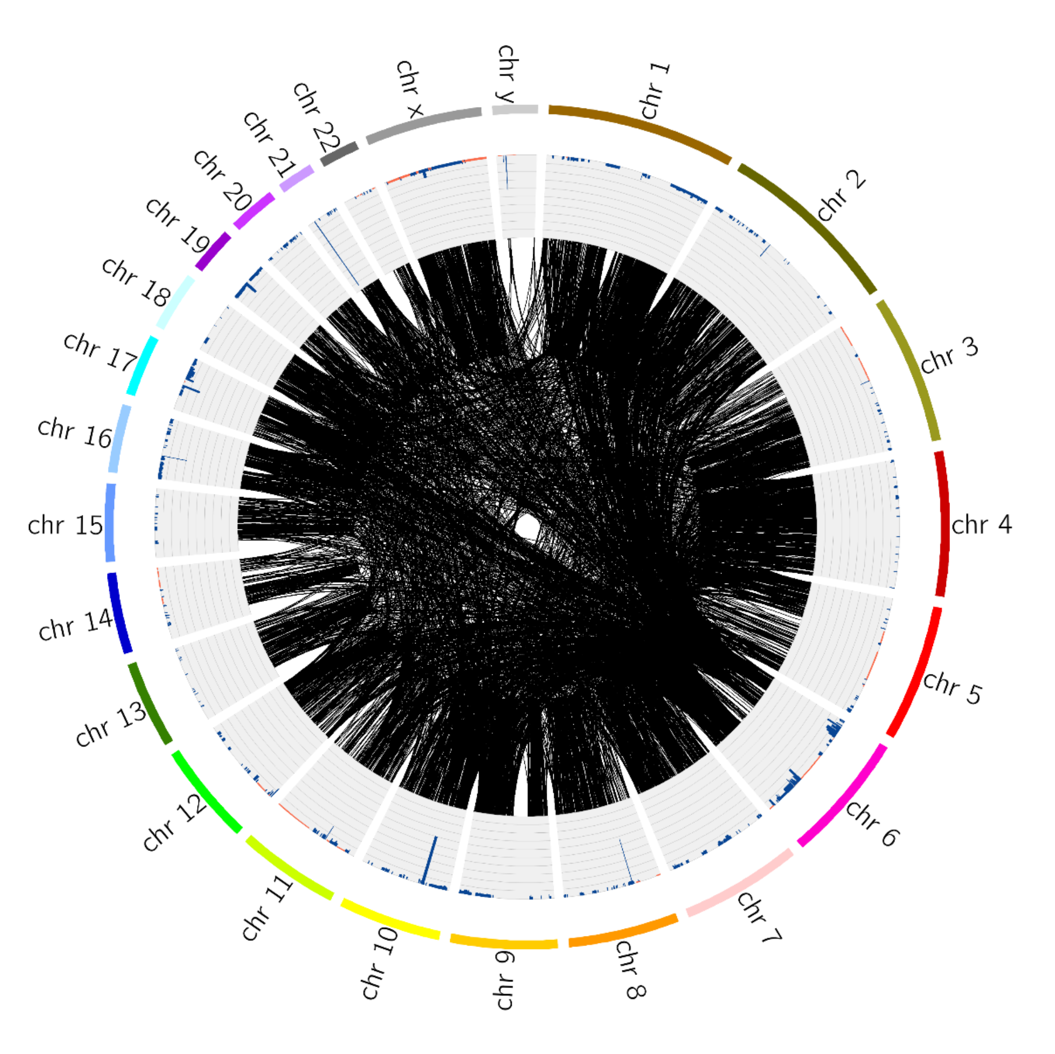

BreakDancer + Control-FREEC

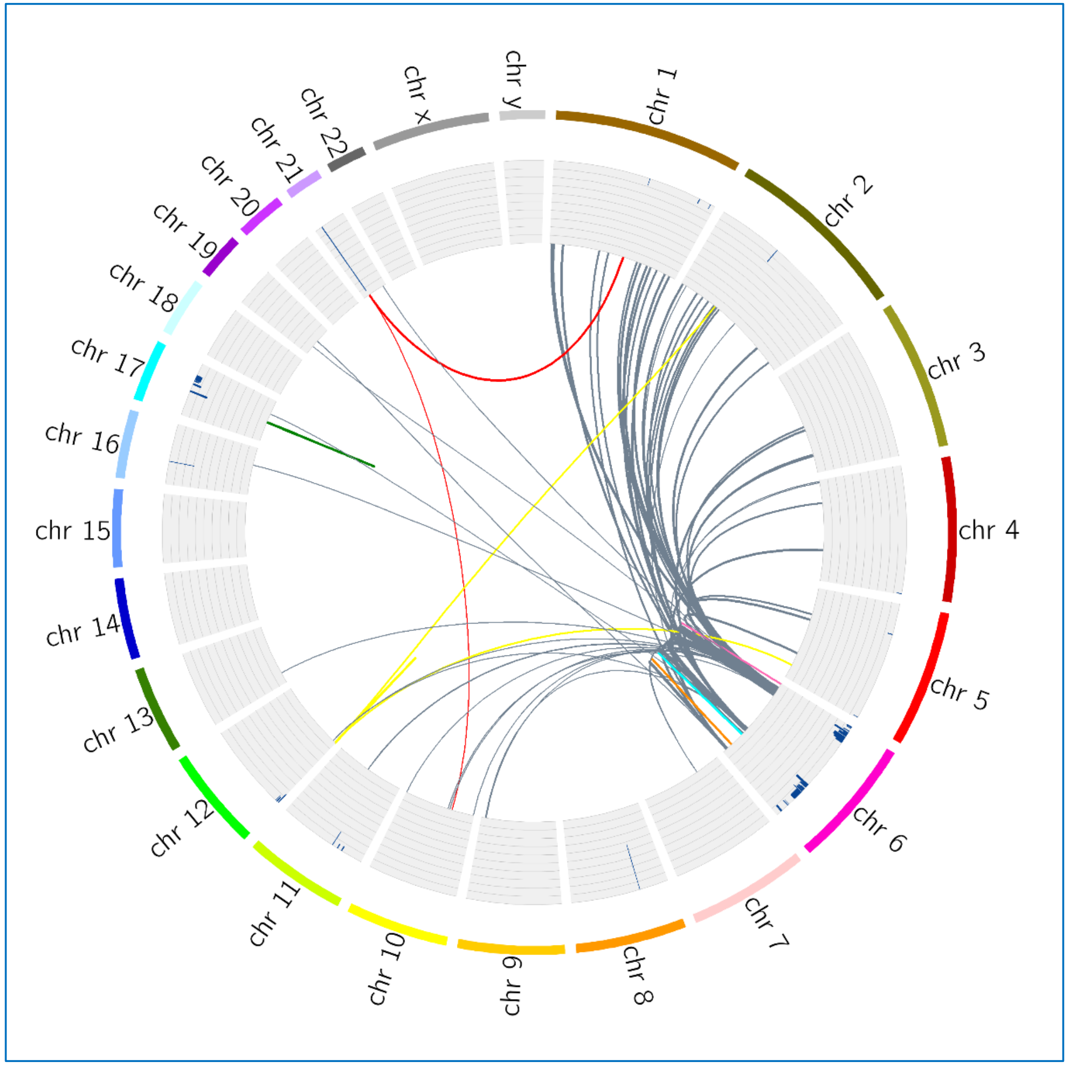

FAST – Whole Genome

p-108

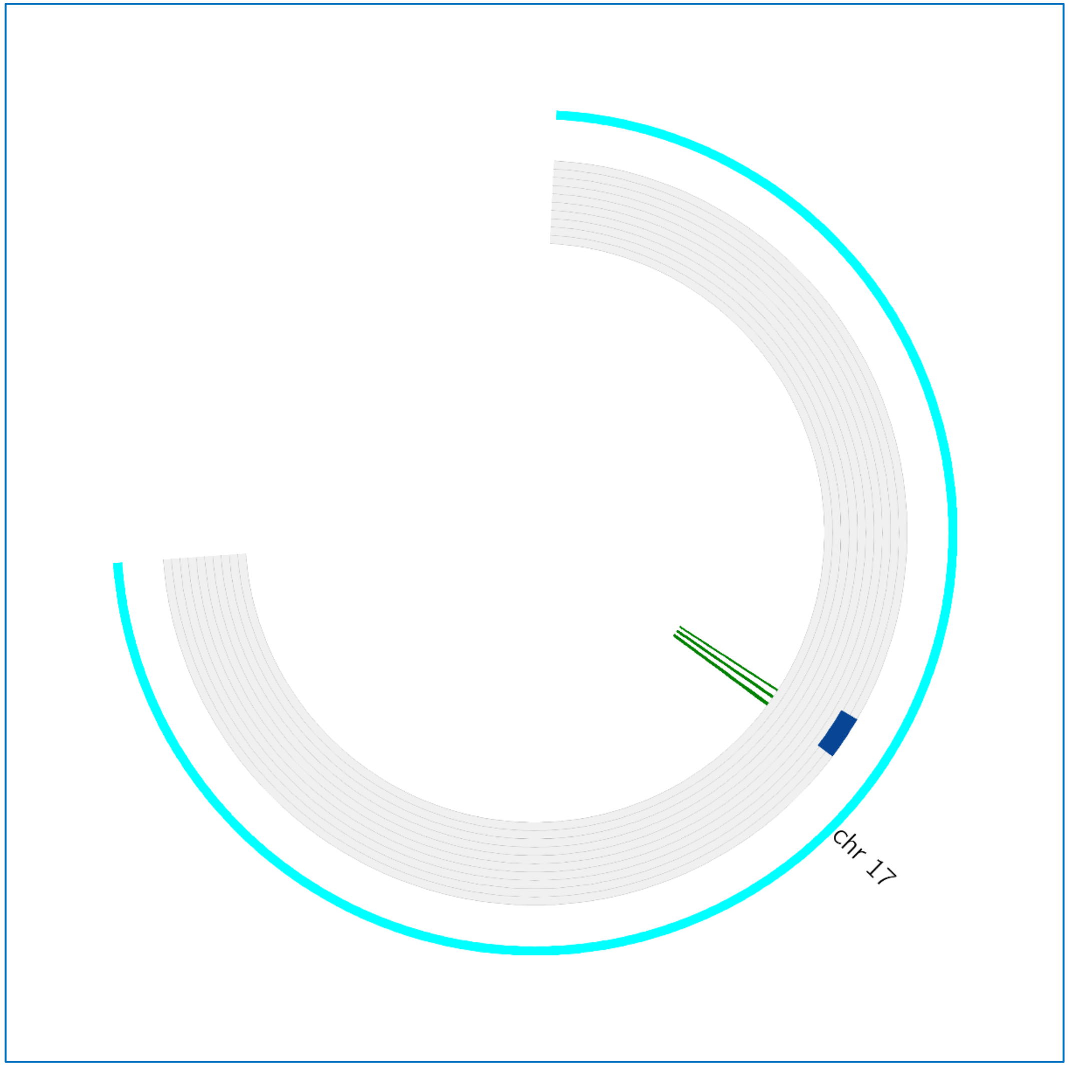

FAST – ERBB2 amplicon

p-112

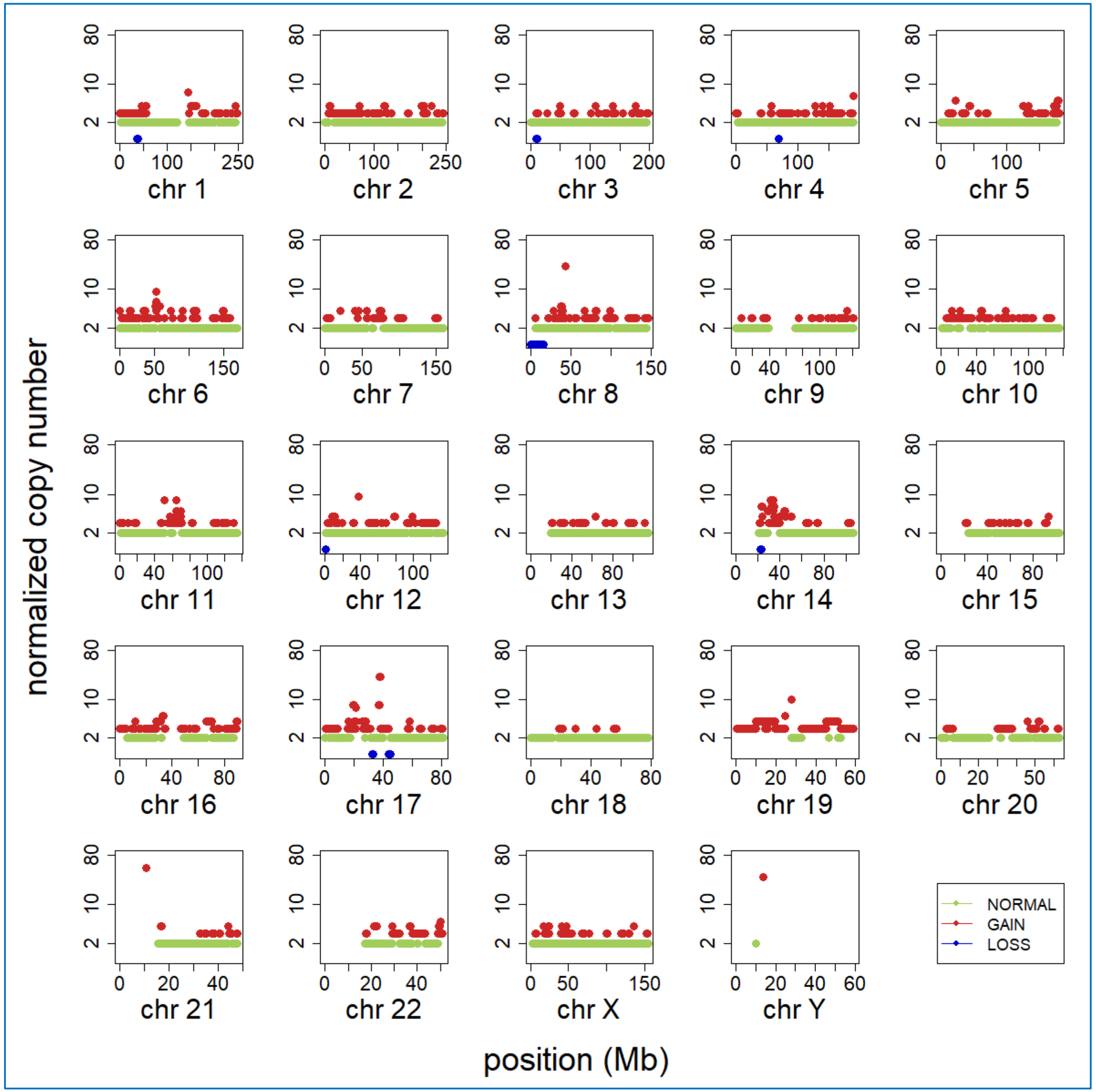

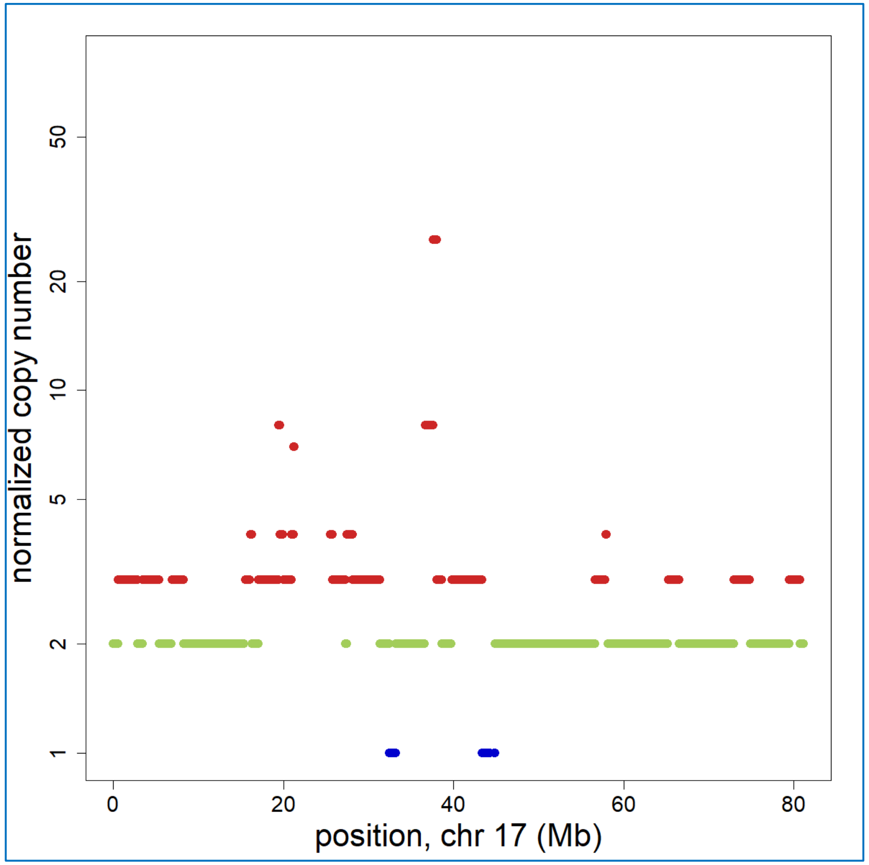

Control-FREEC

p-112

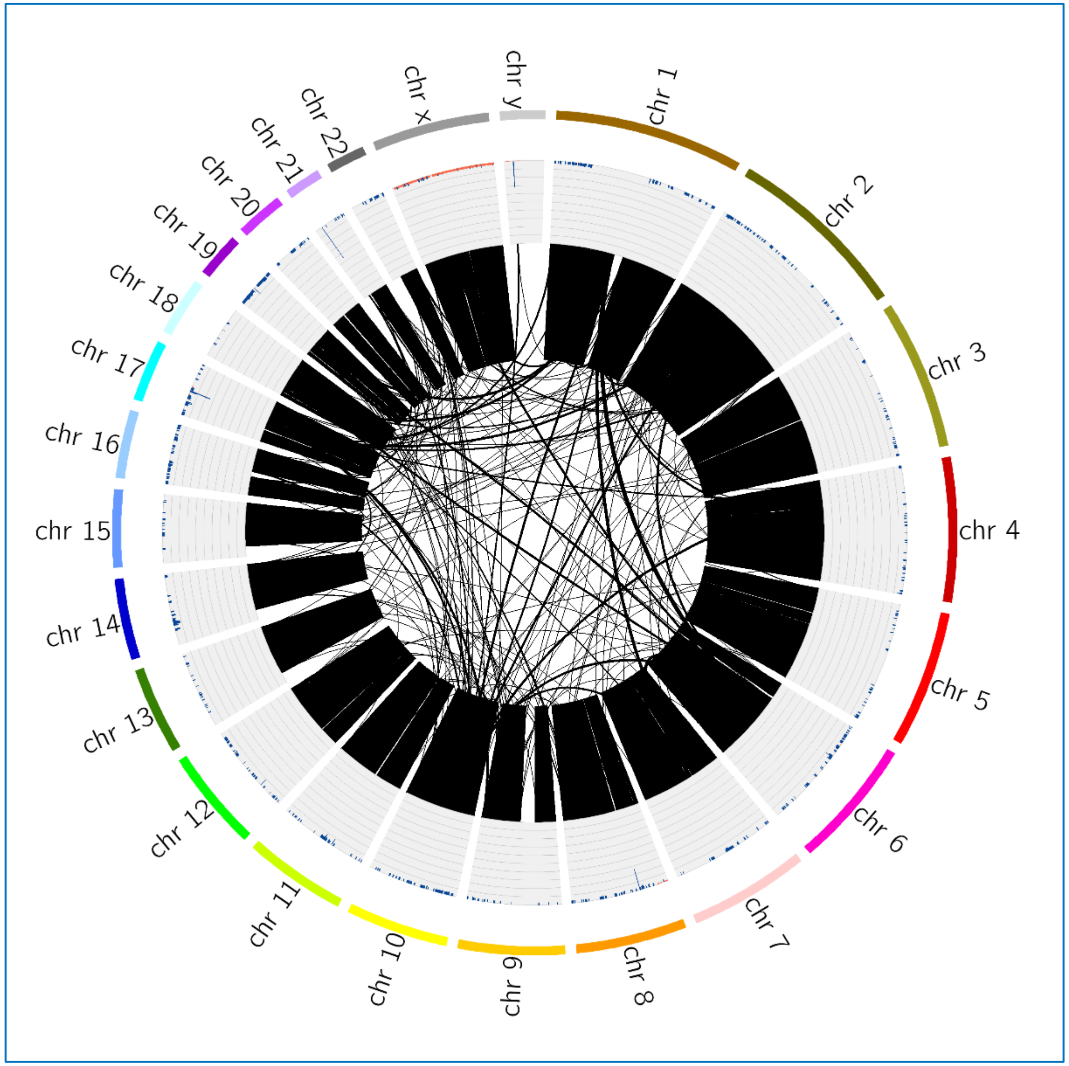

BreakDancer + Control-FREEC

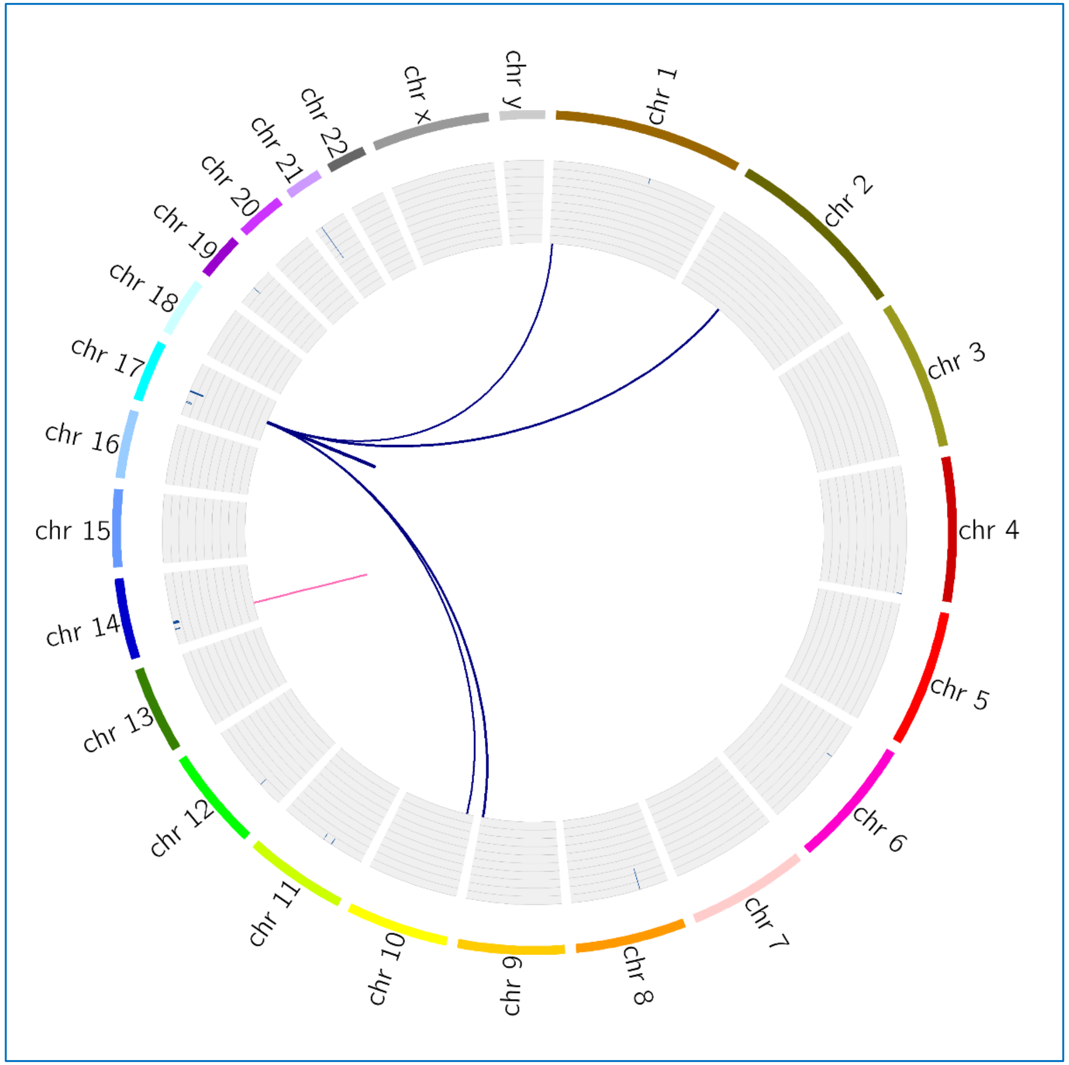

FAST – Whole Genome

p-112

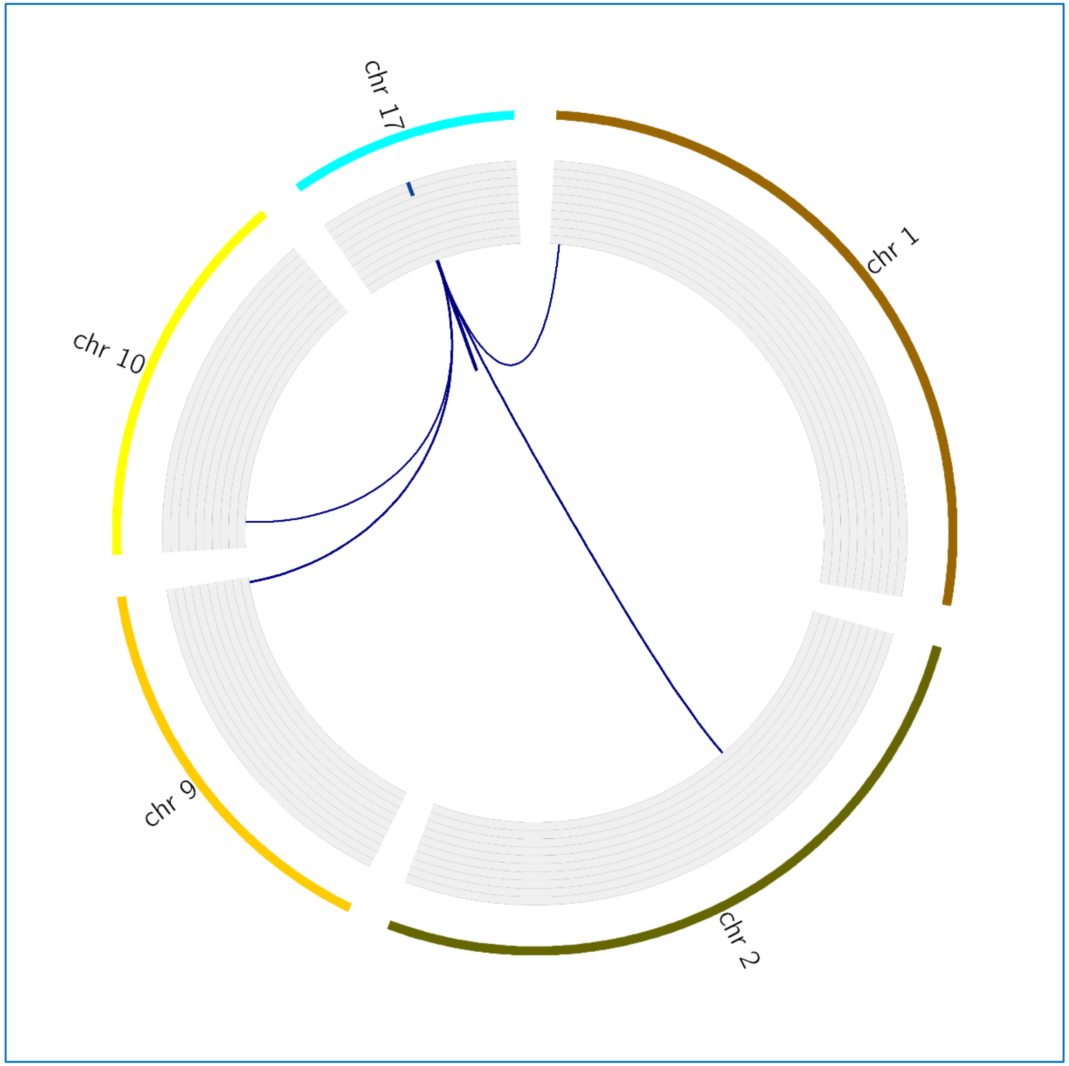

FAST – ERBB2 amplicon

p-114

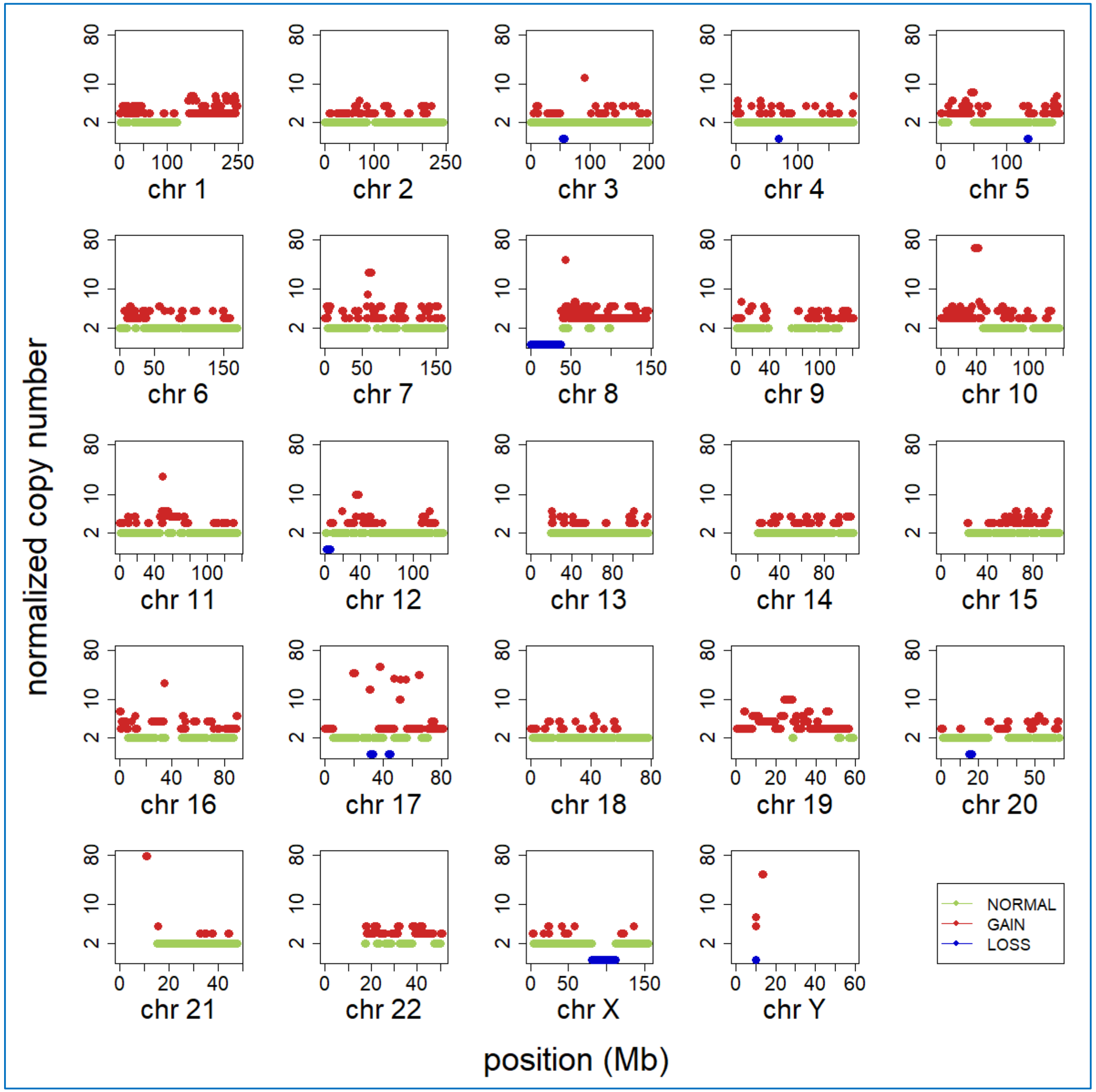

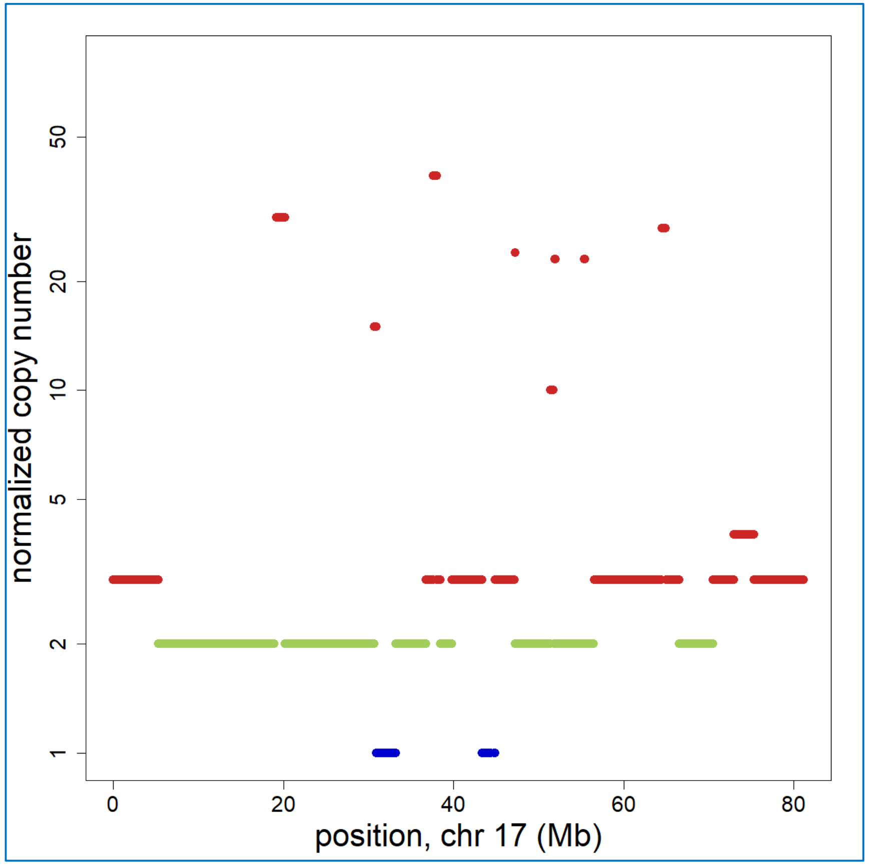

Control-FREEC

p-114

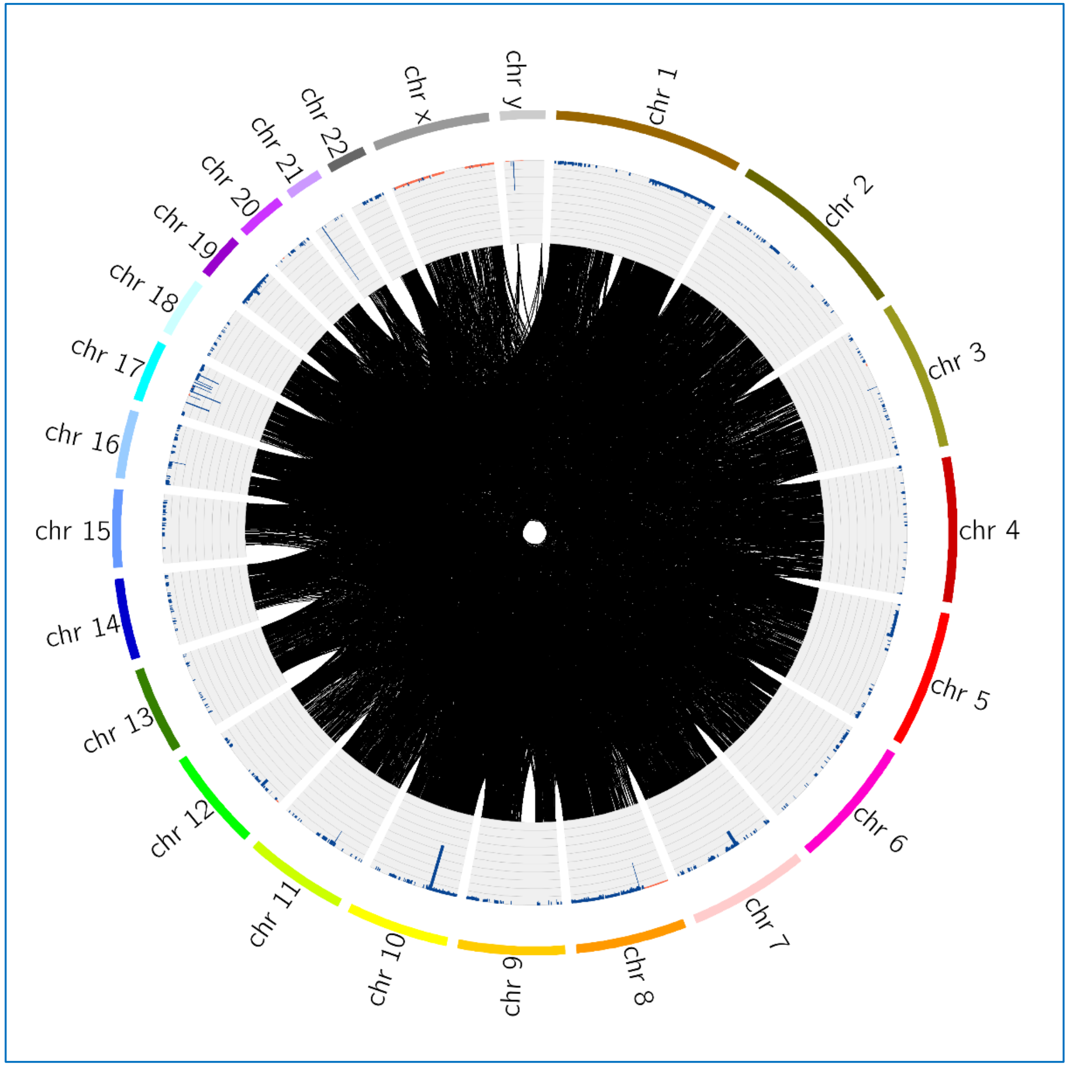

BreakDancer + Control-FREEC

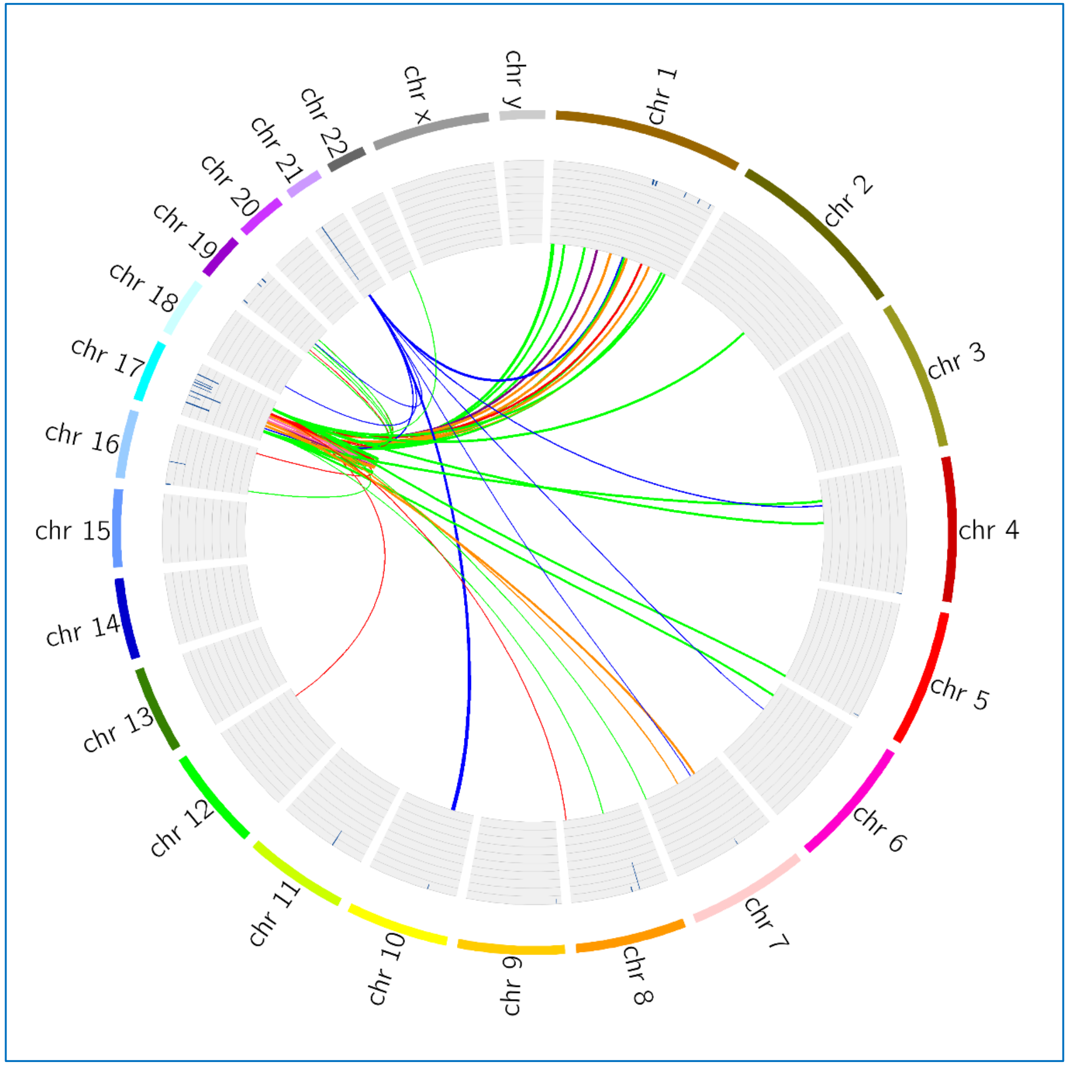

FAST – Whole Genome

p-114

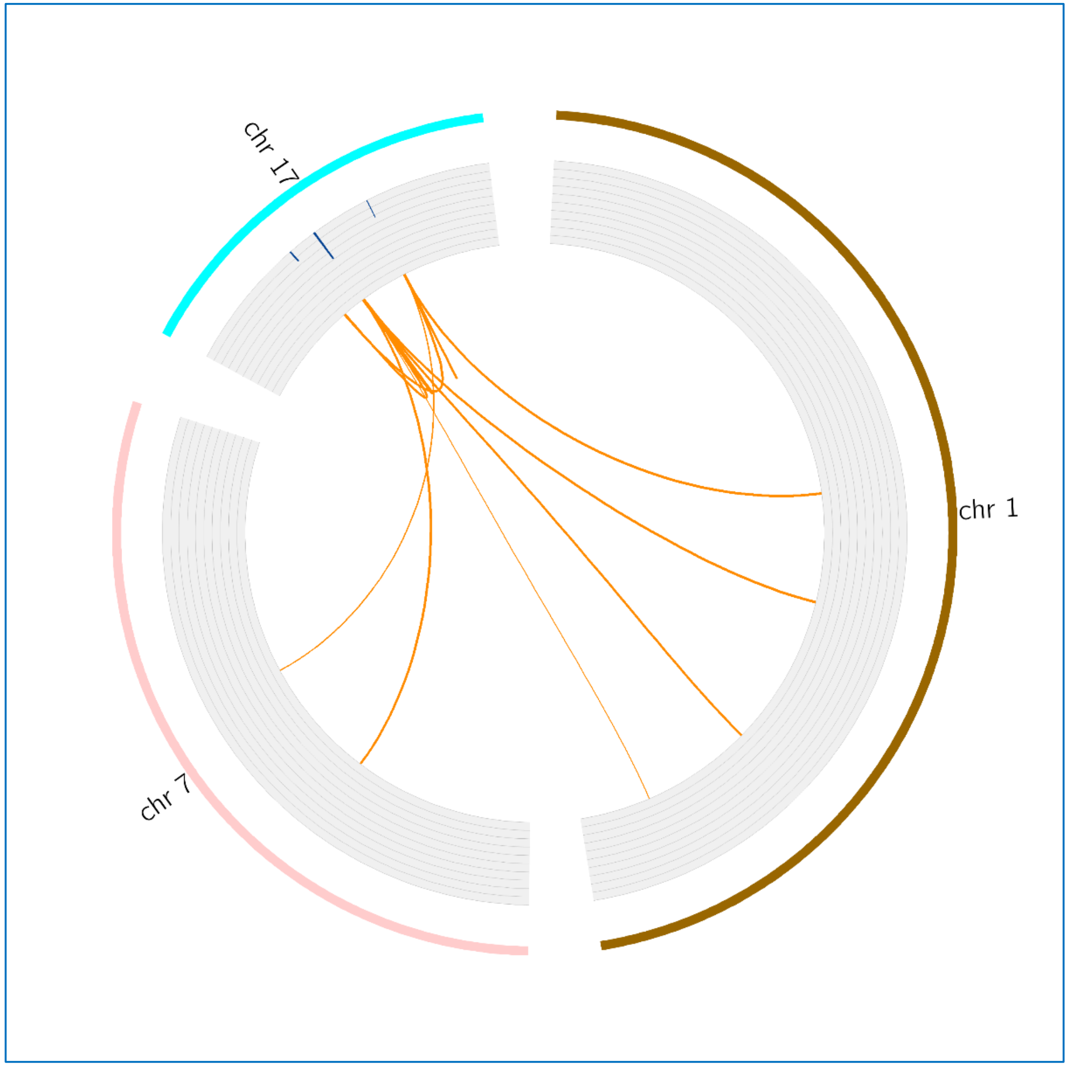

FAST – ERBB2 amplicon

p-115

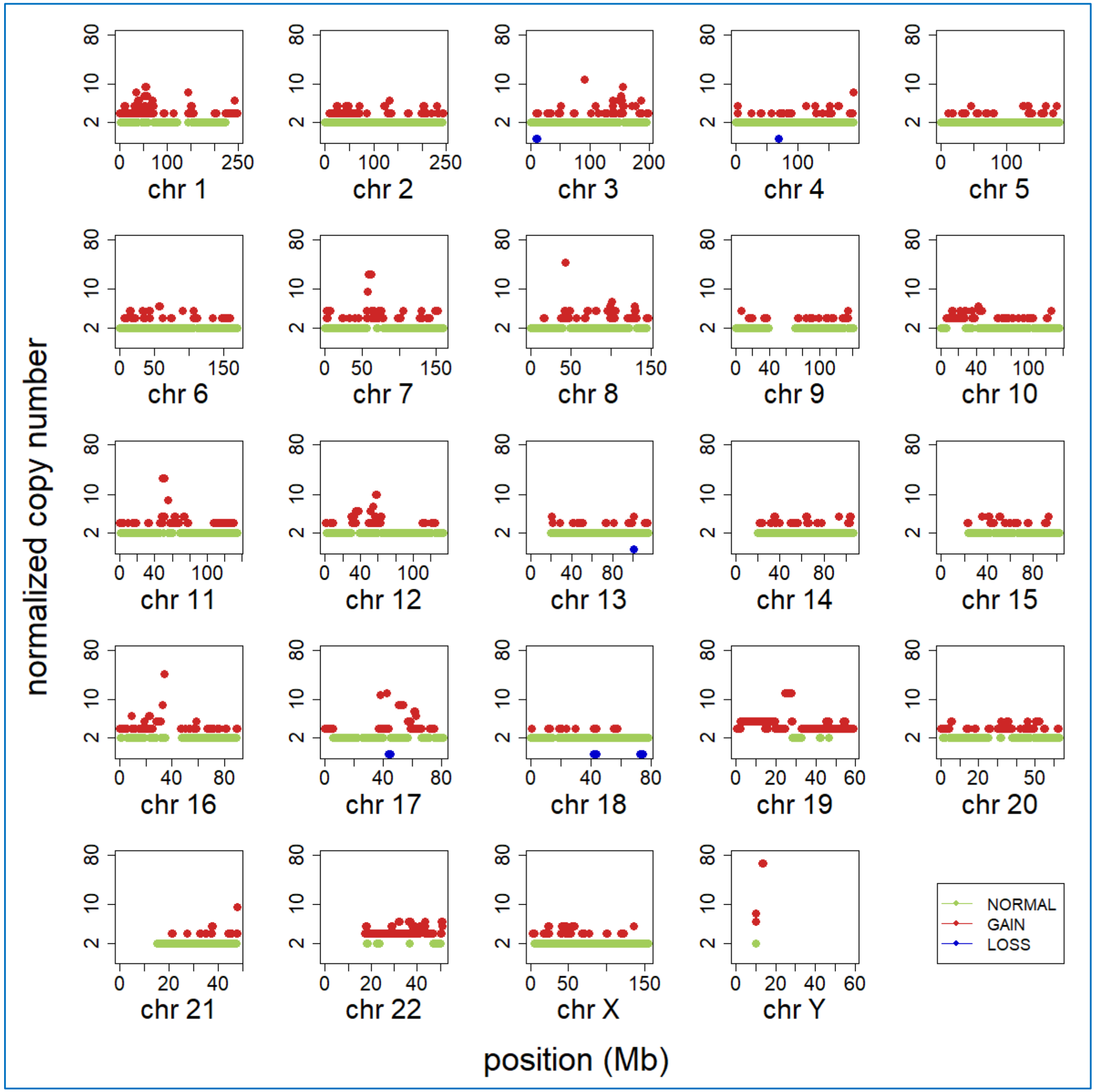

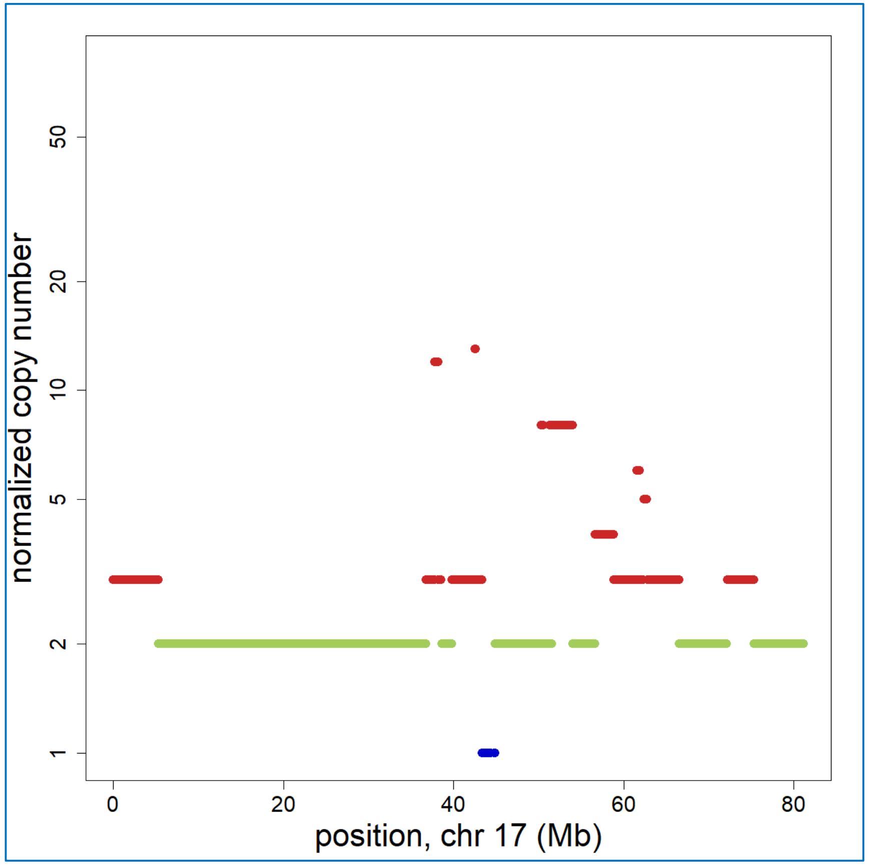

Control-FREEC

p-115

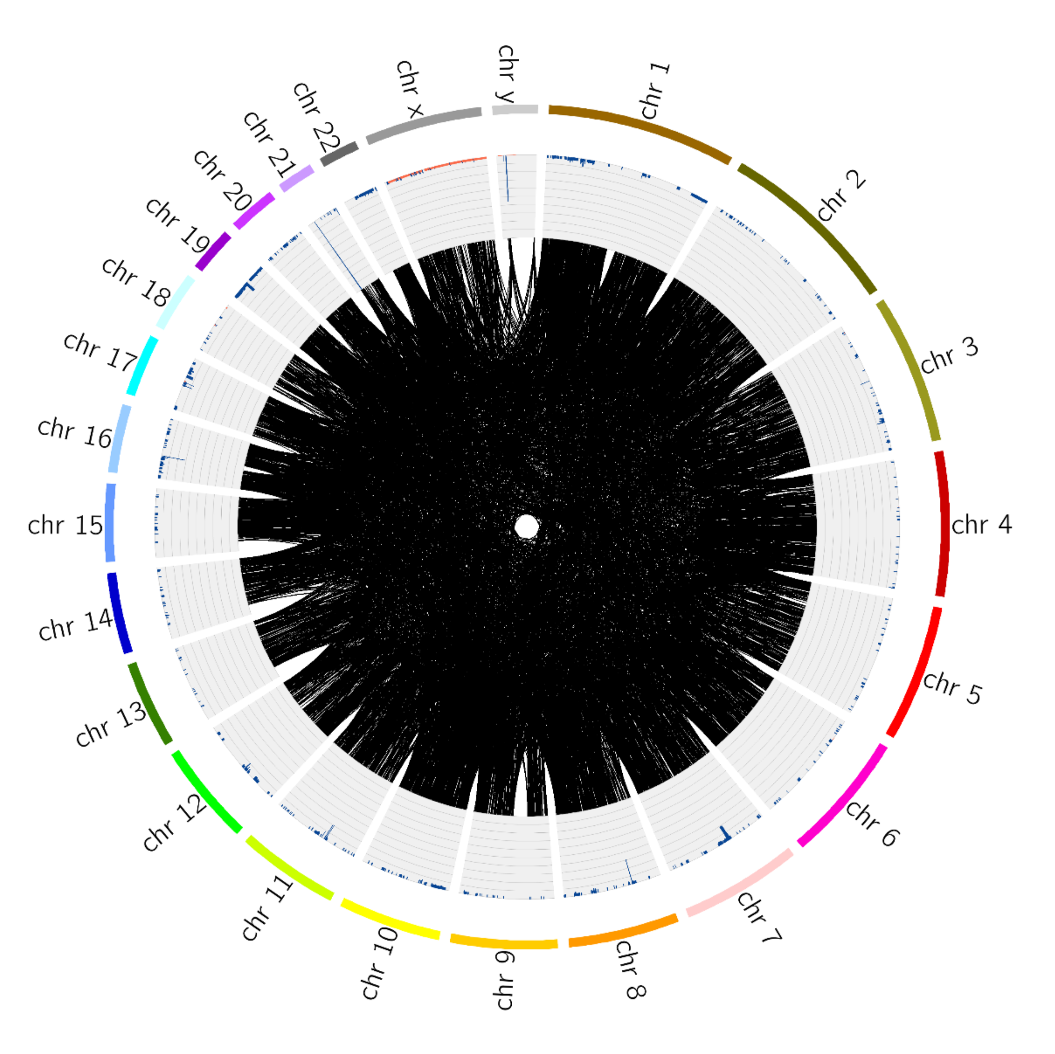

BreakDancer + Control-FREEC

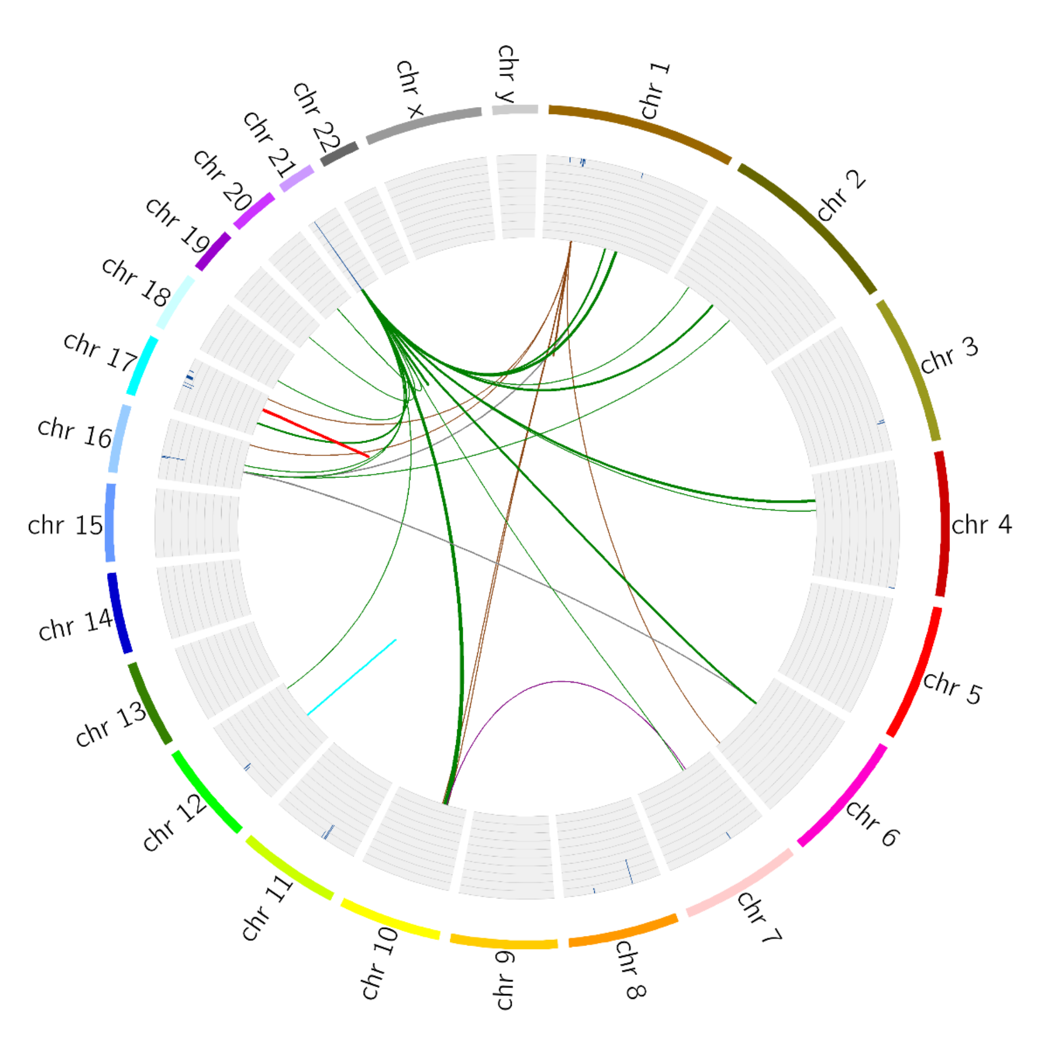

FAST – Whole Genome

p-115

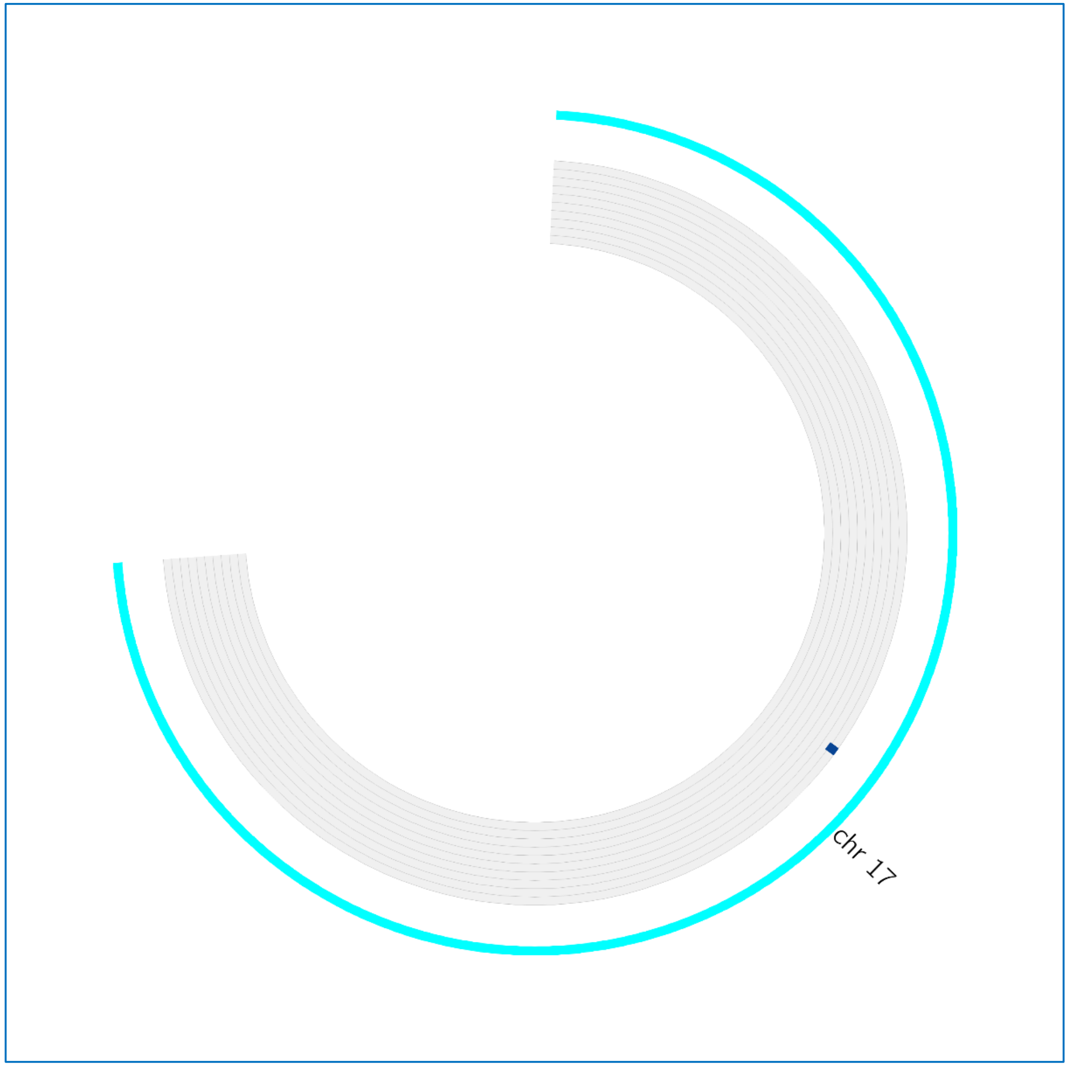

FAST – ERBB2 amplicon

p-119

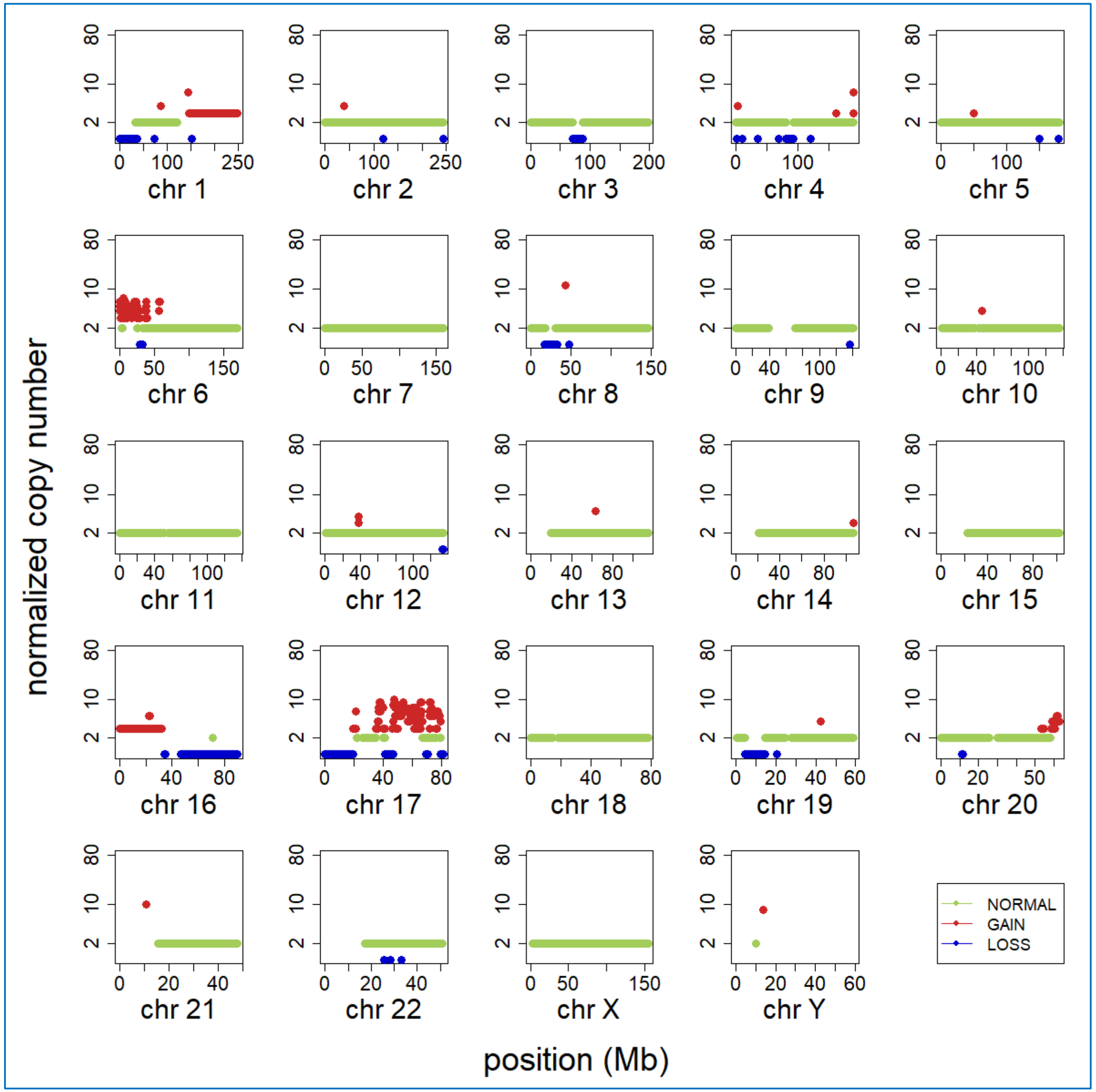

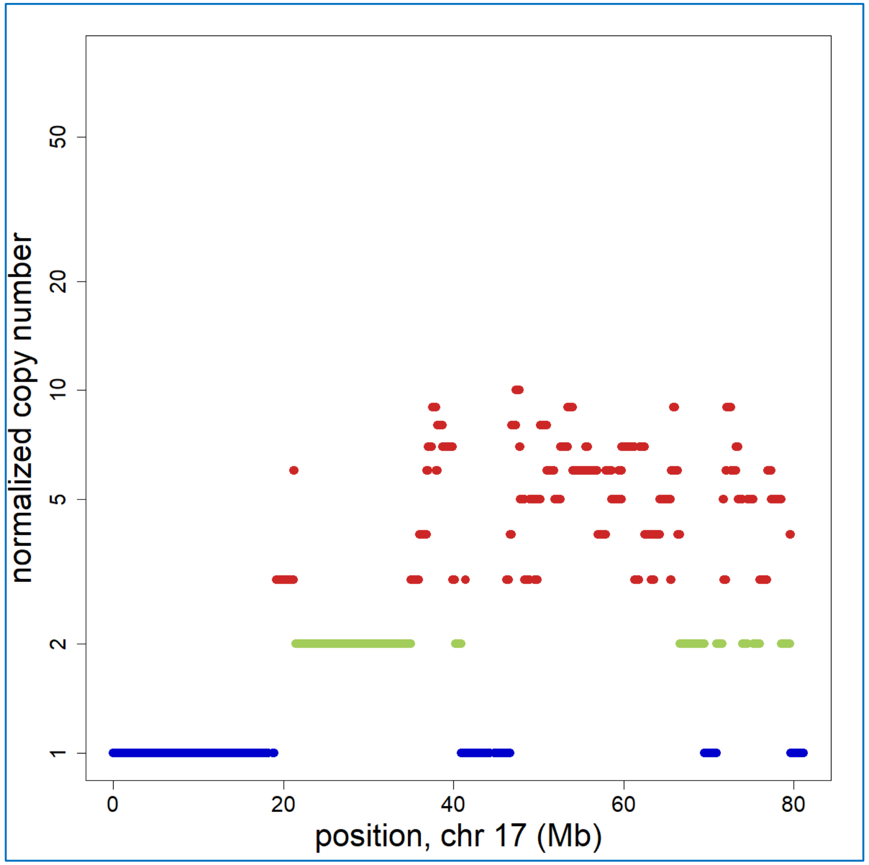

Control-FREEC

p-119

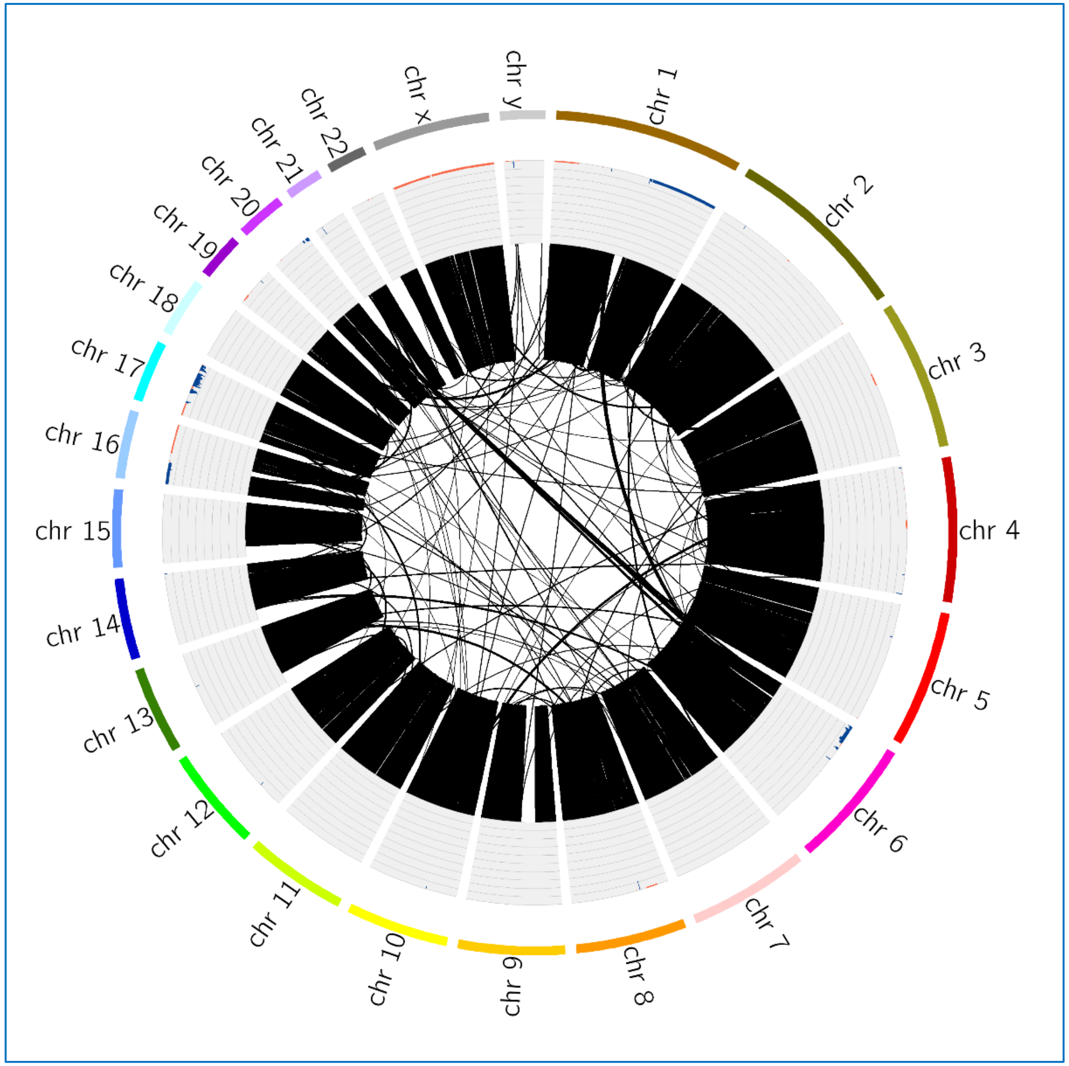

BreakDancer + Control-FREEC

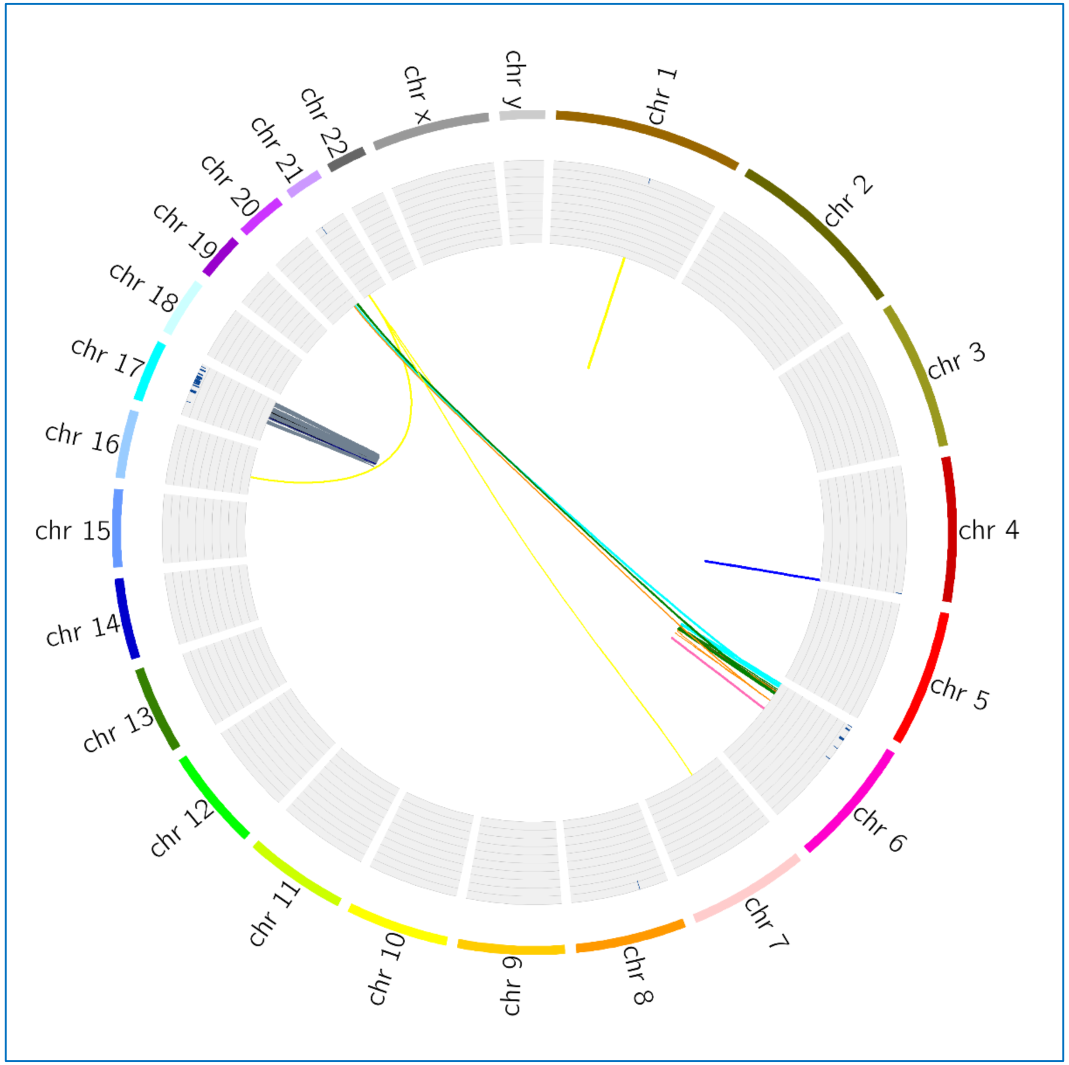

FAST – Whole Genome

p-119

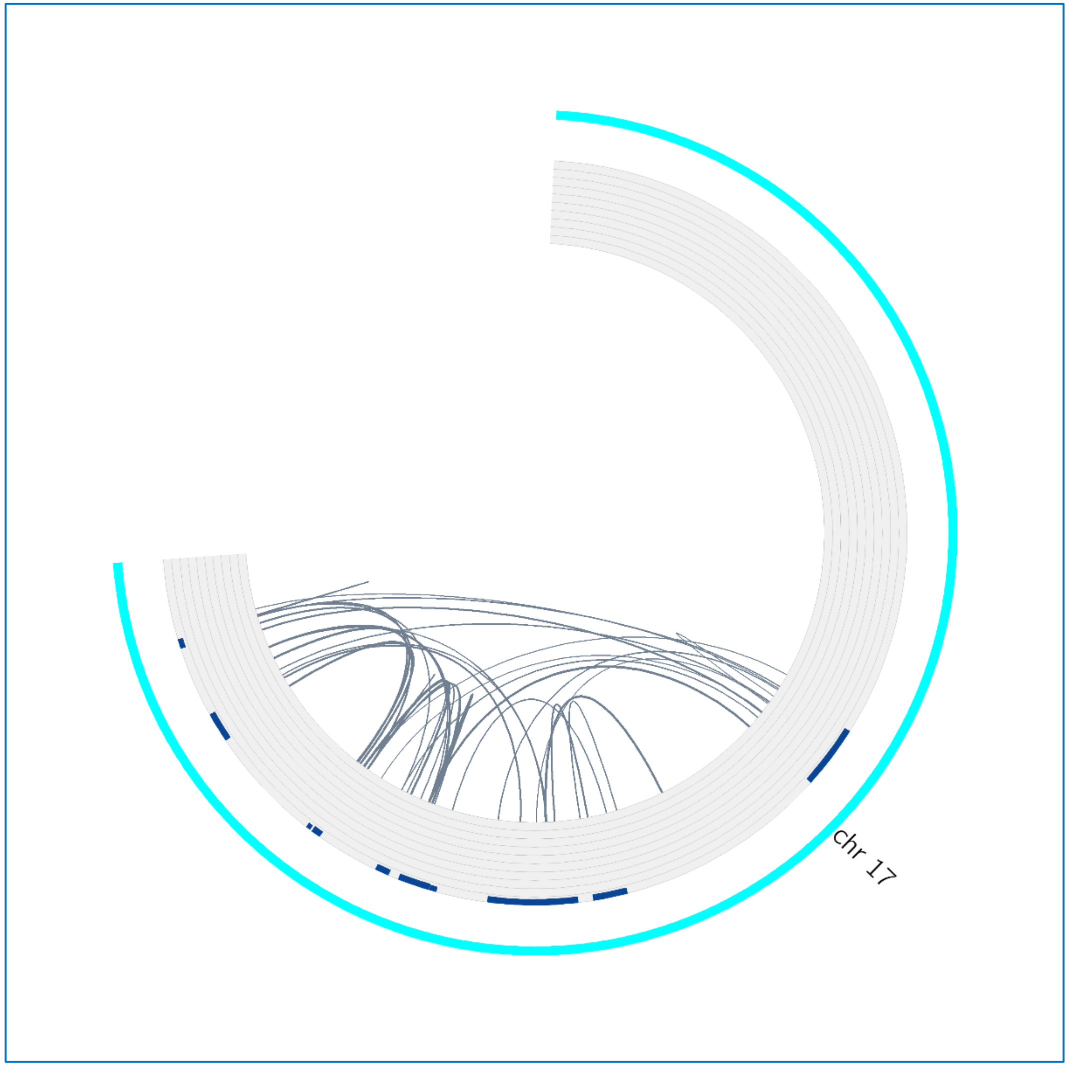

FAST – ERBB2 amplicon

166

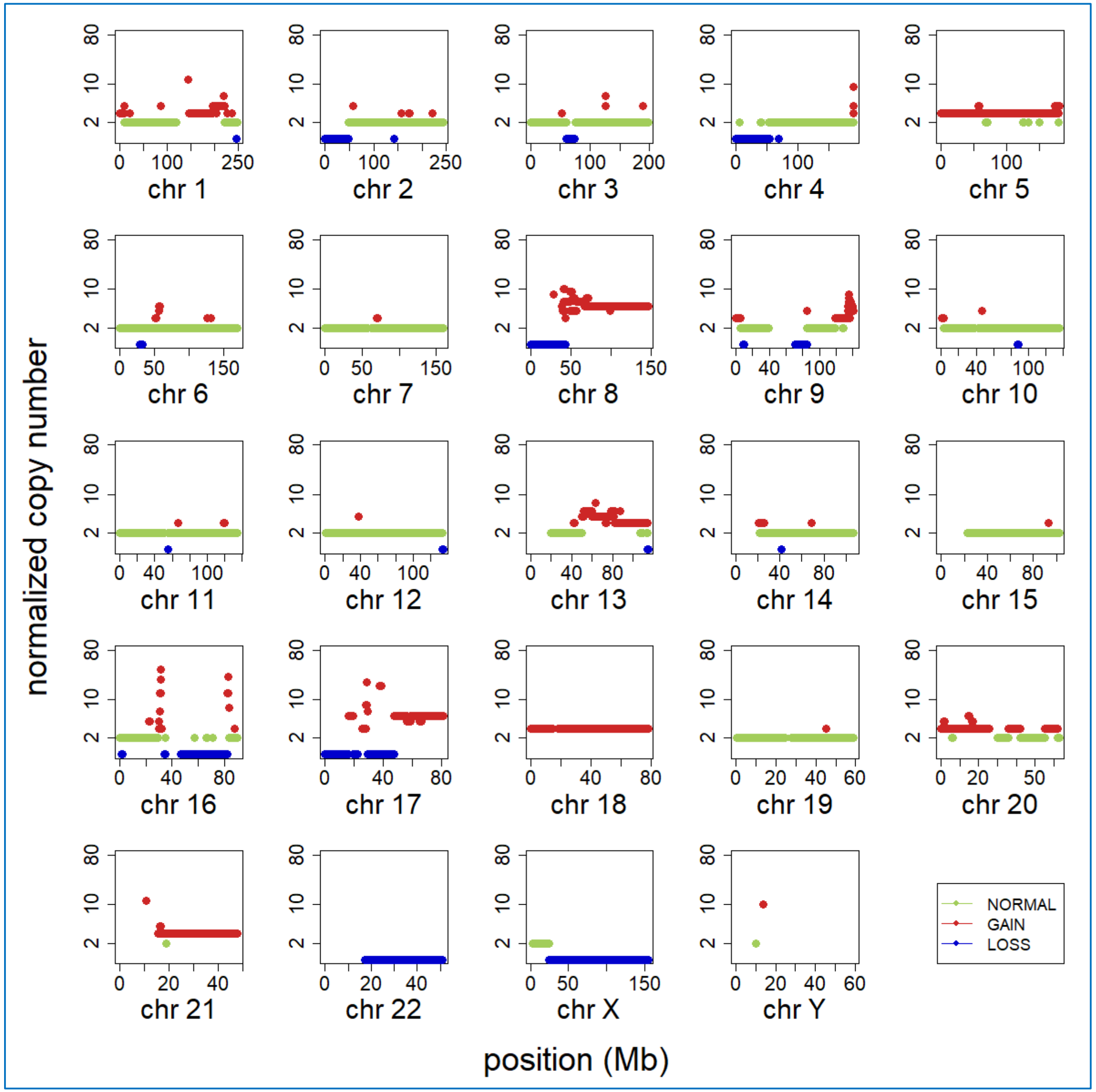

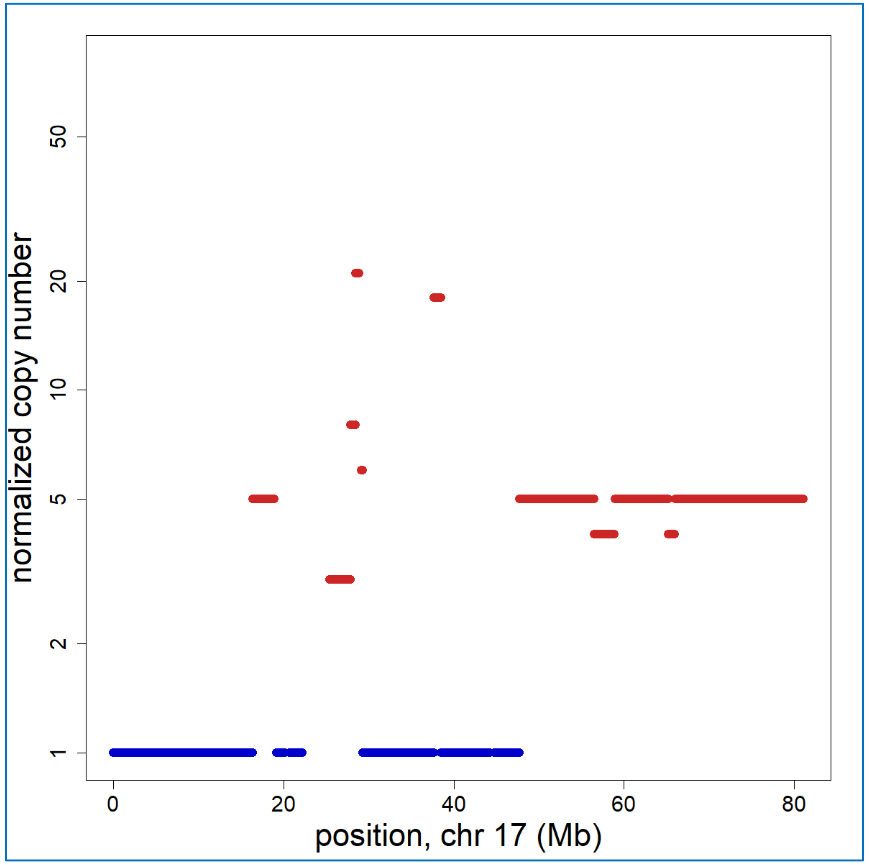

Control-FREEC

166

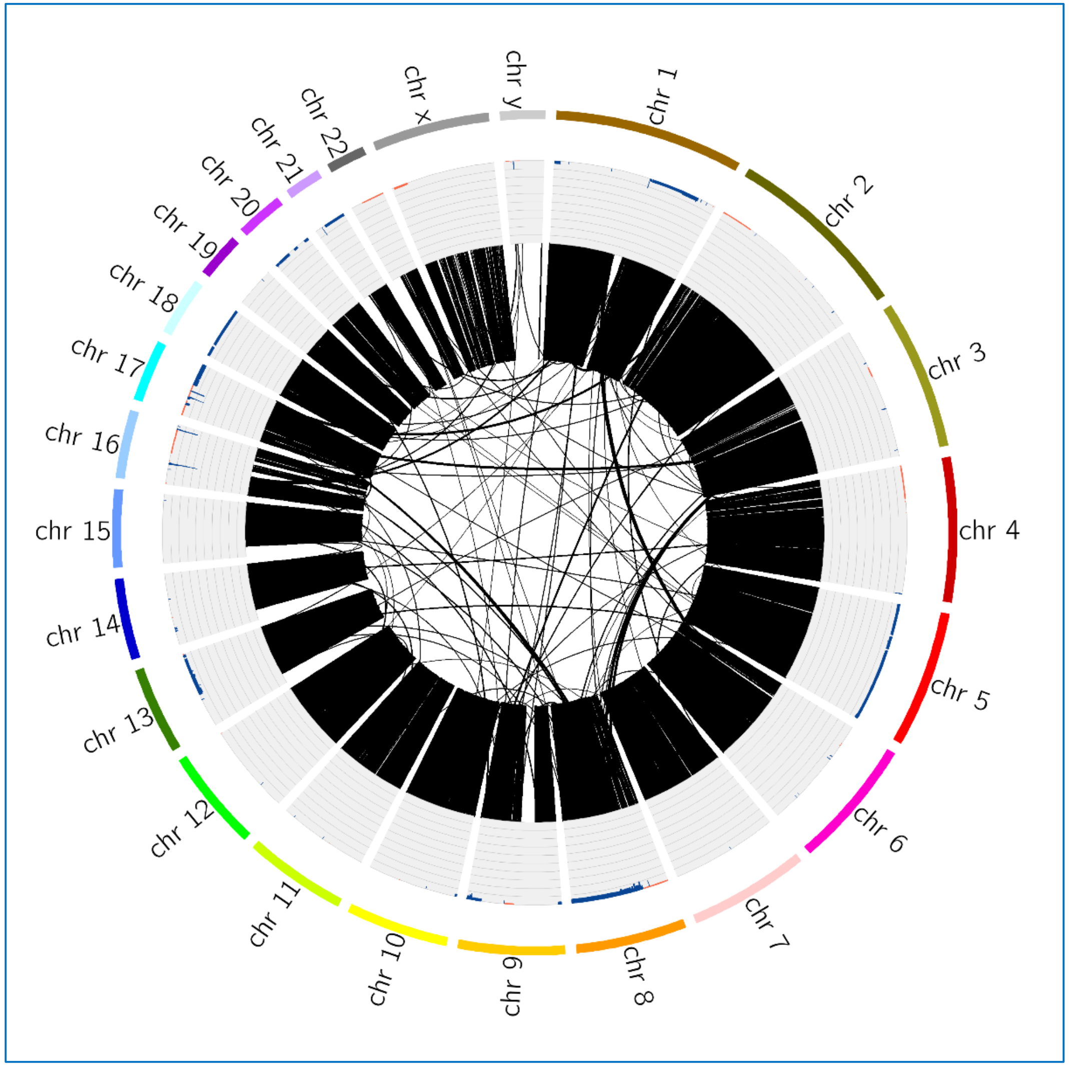

BreakDancer + Control-FREEC

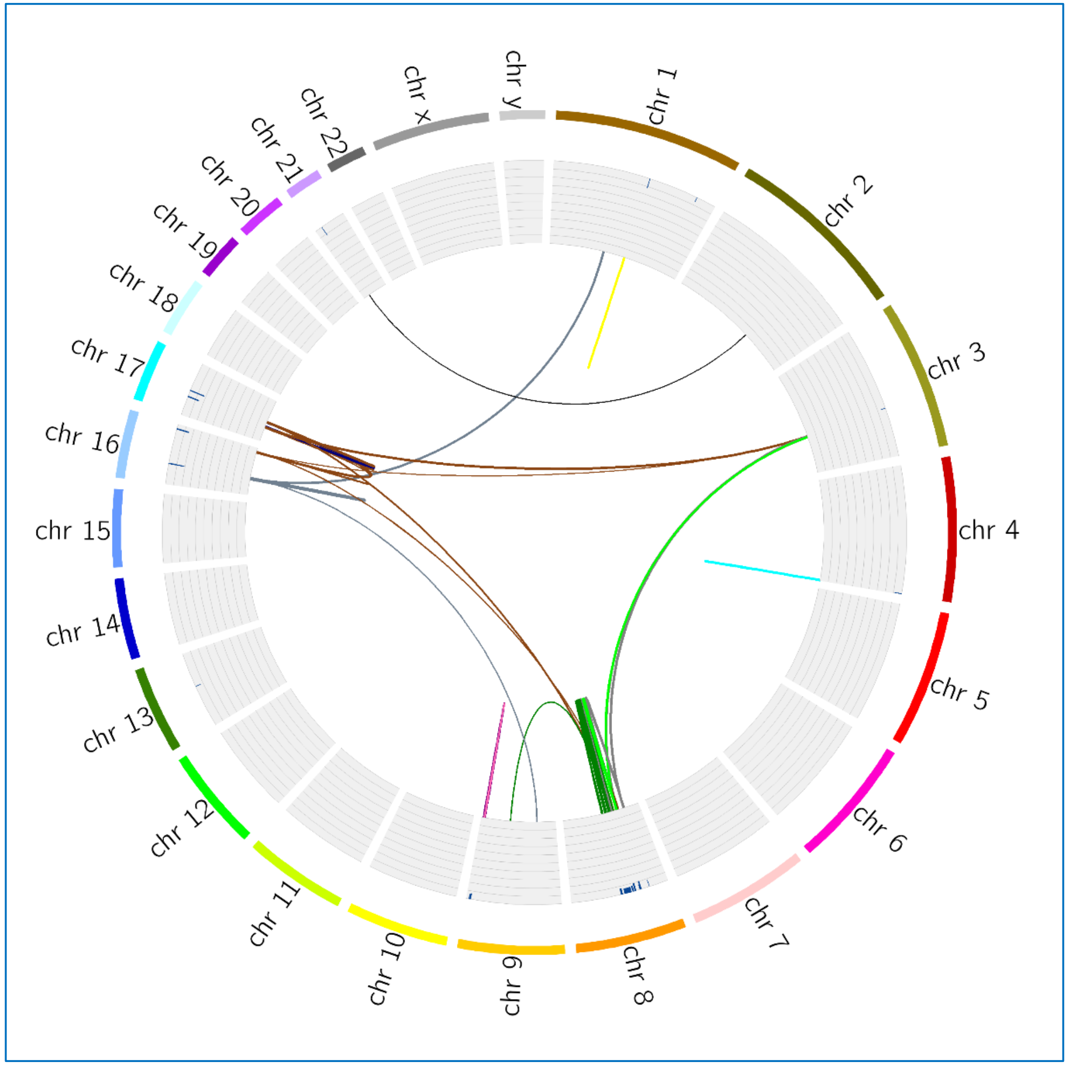

FAST – Whole Genome

166

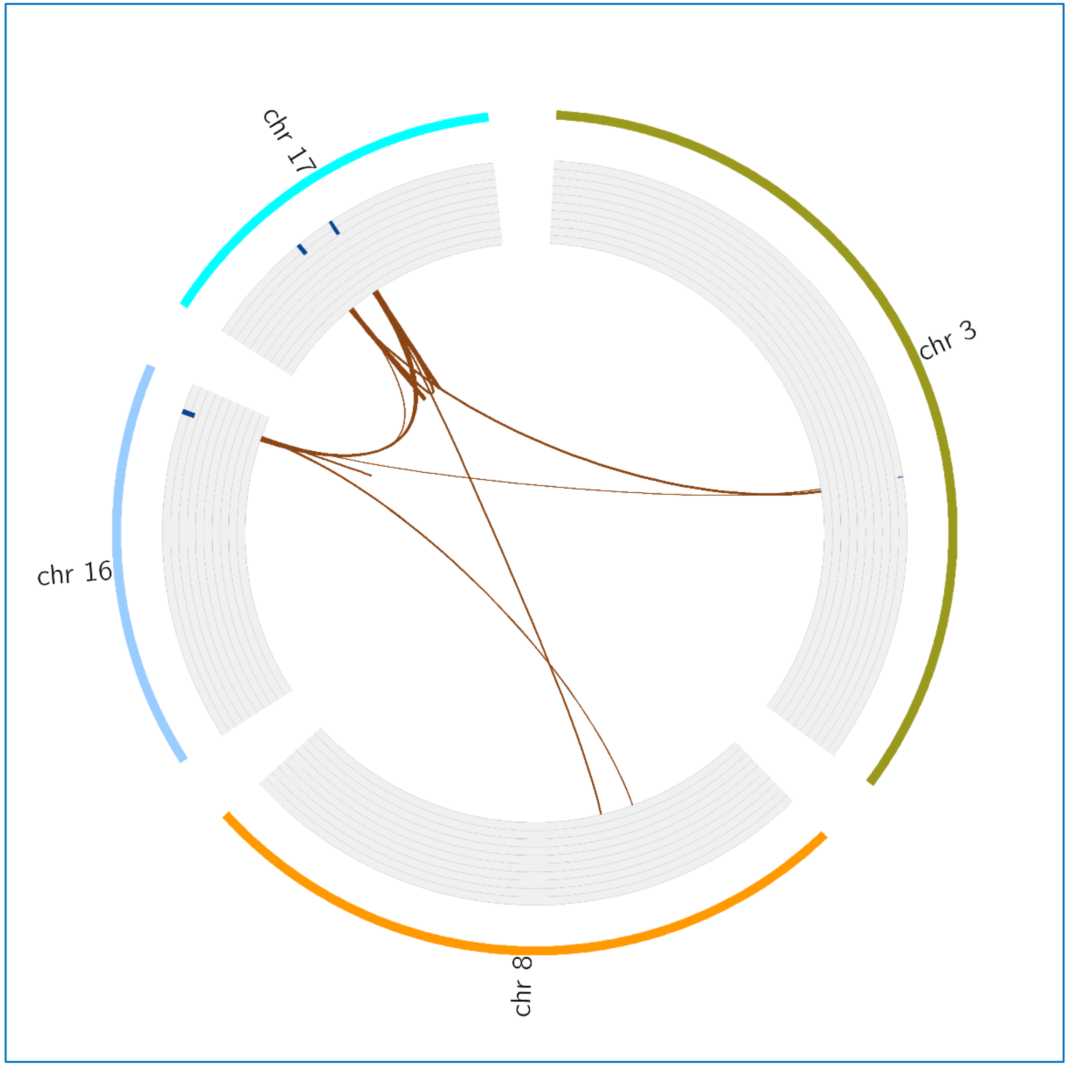

FAST – ERBB2 amplicon

p-203

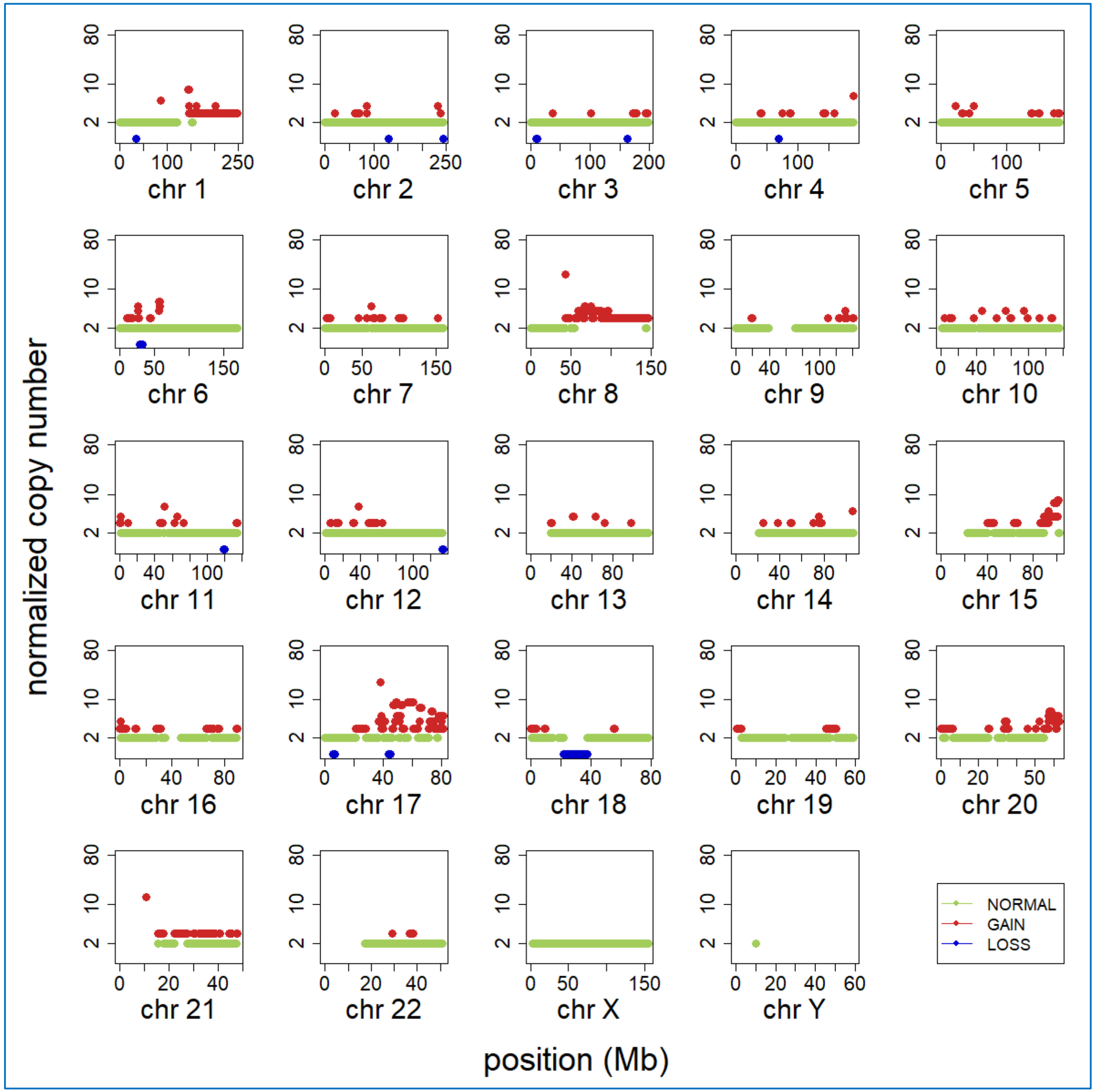

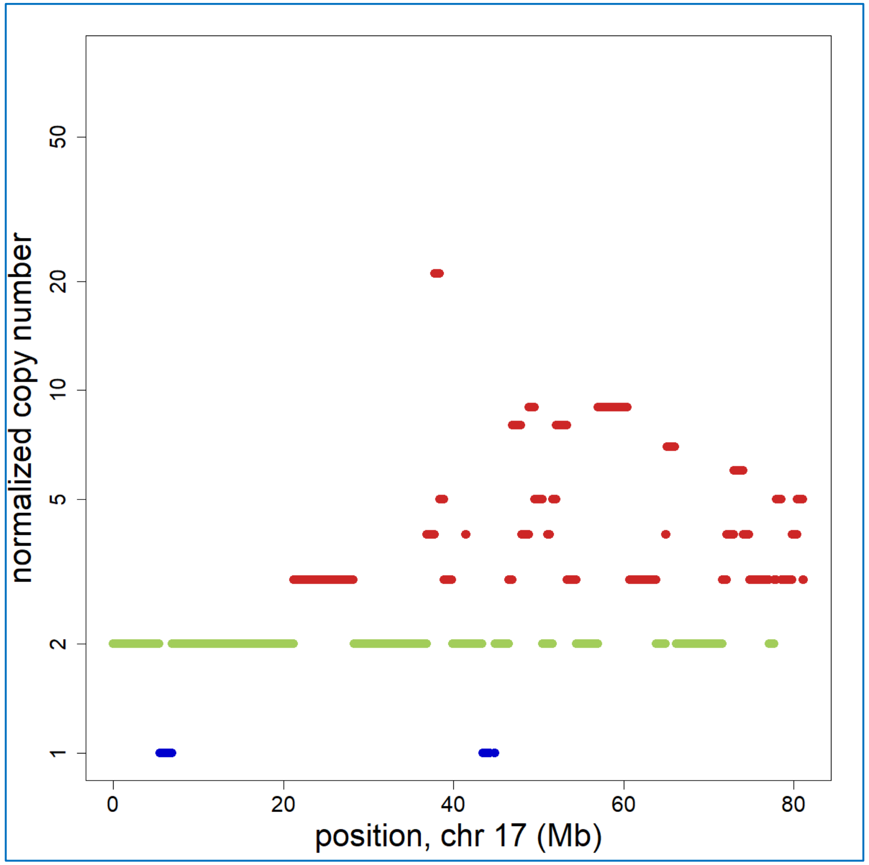

Control-FREEC

p-203

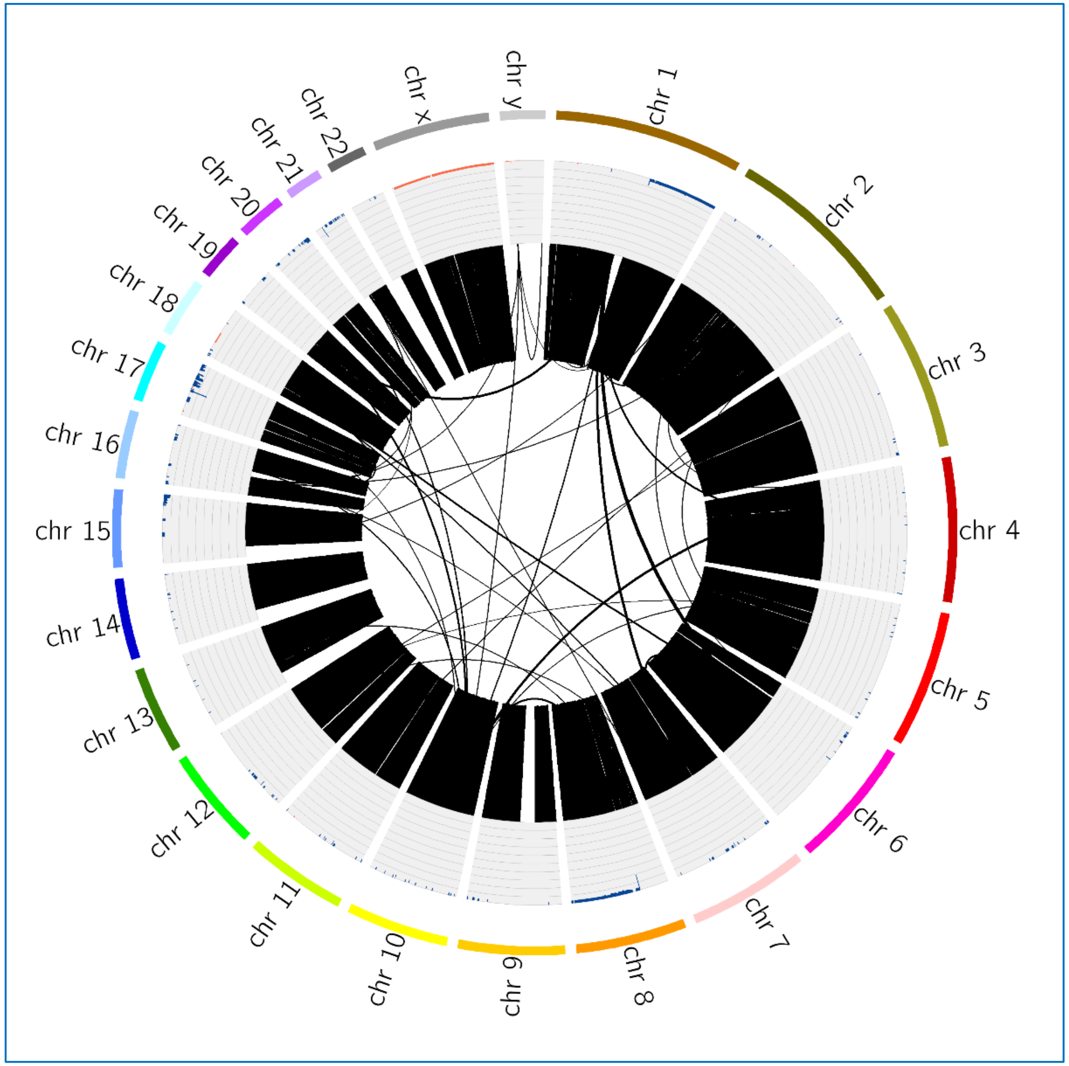

BreakDancer + Control-FREEC

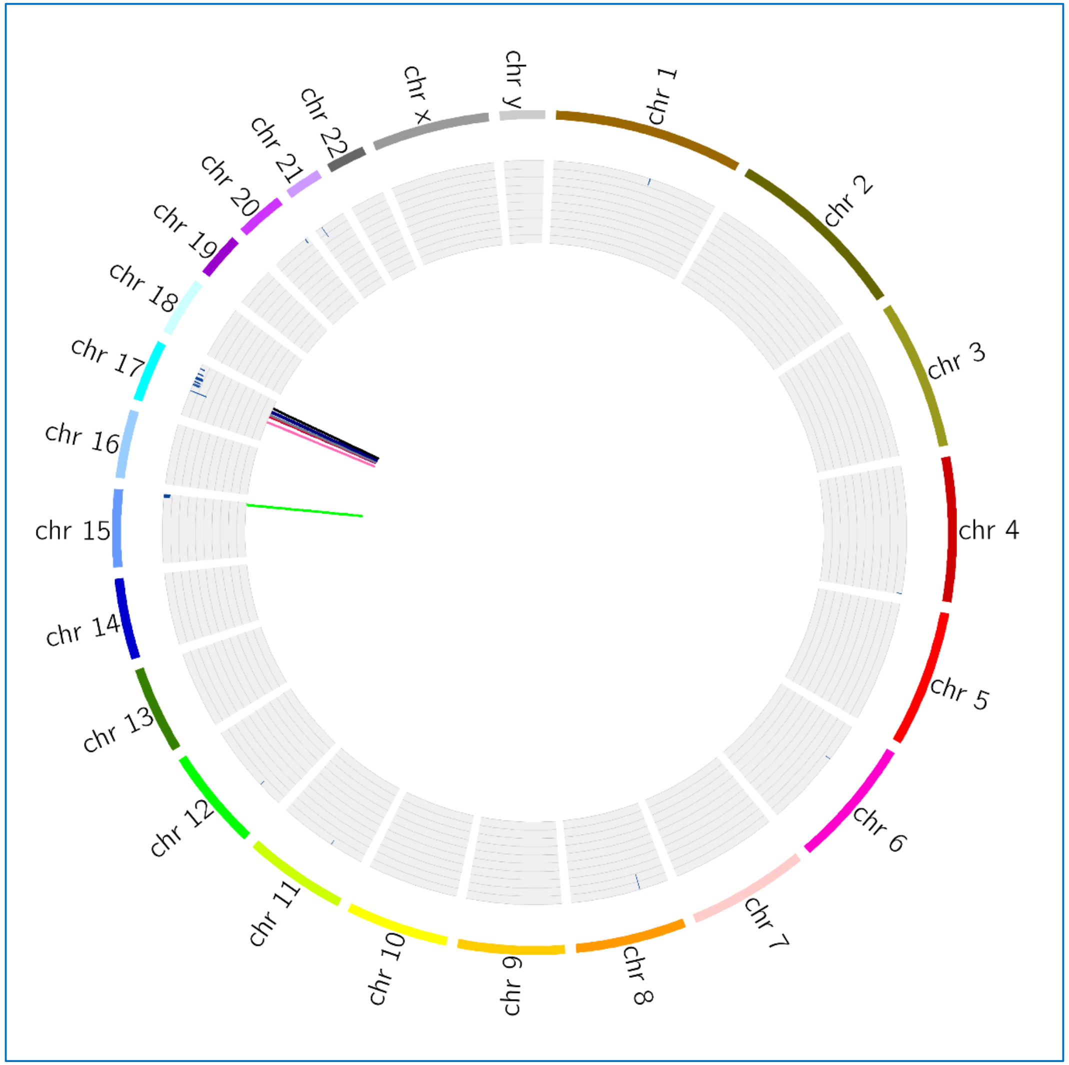

FAST – Whole Genome

p-203

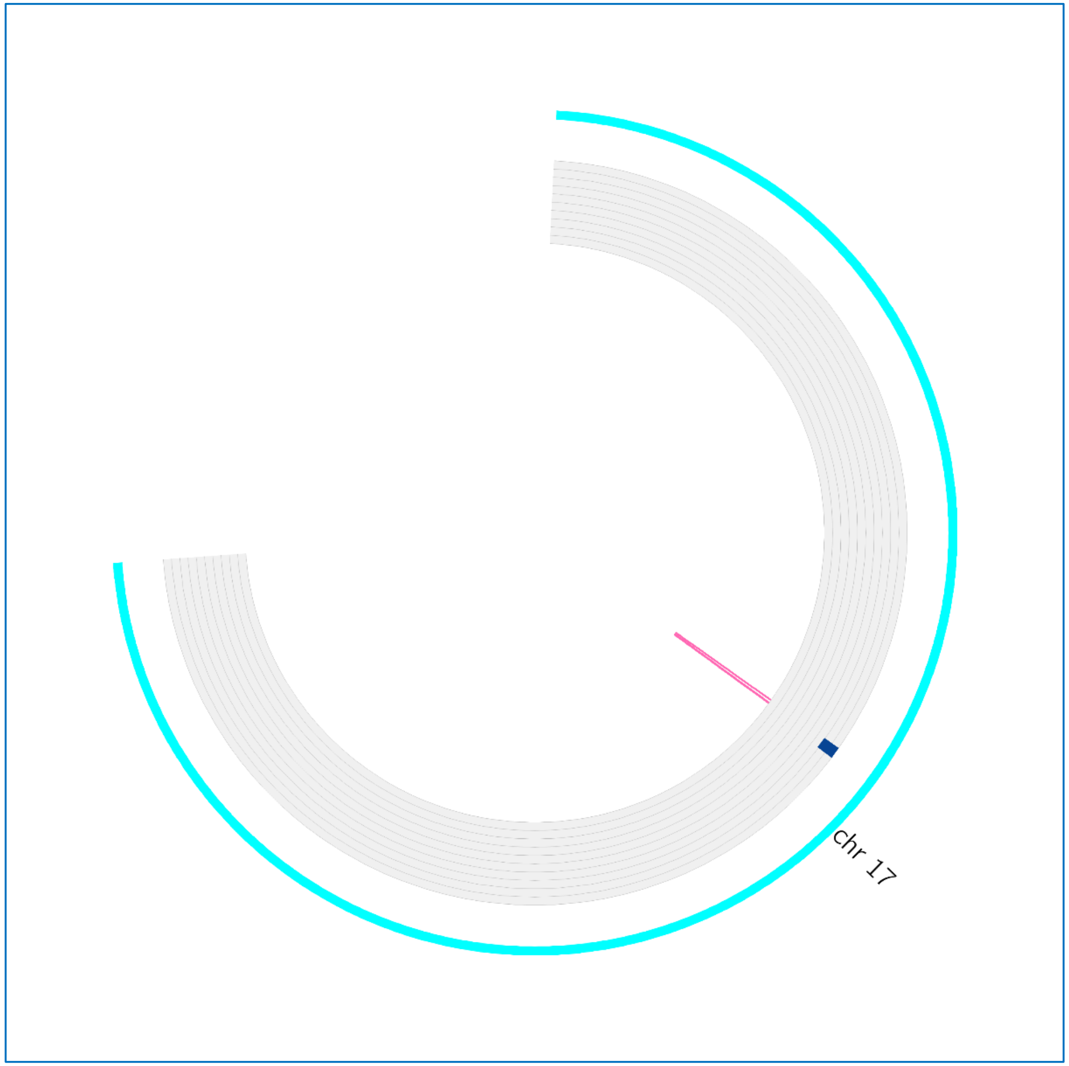

FAST – ERBB2 amplicon

p-204

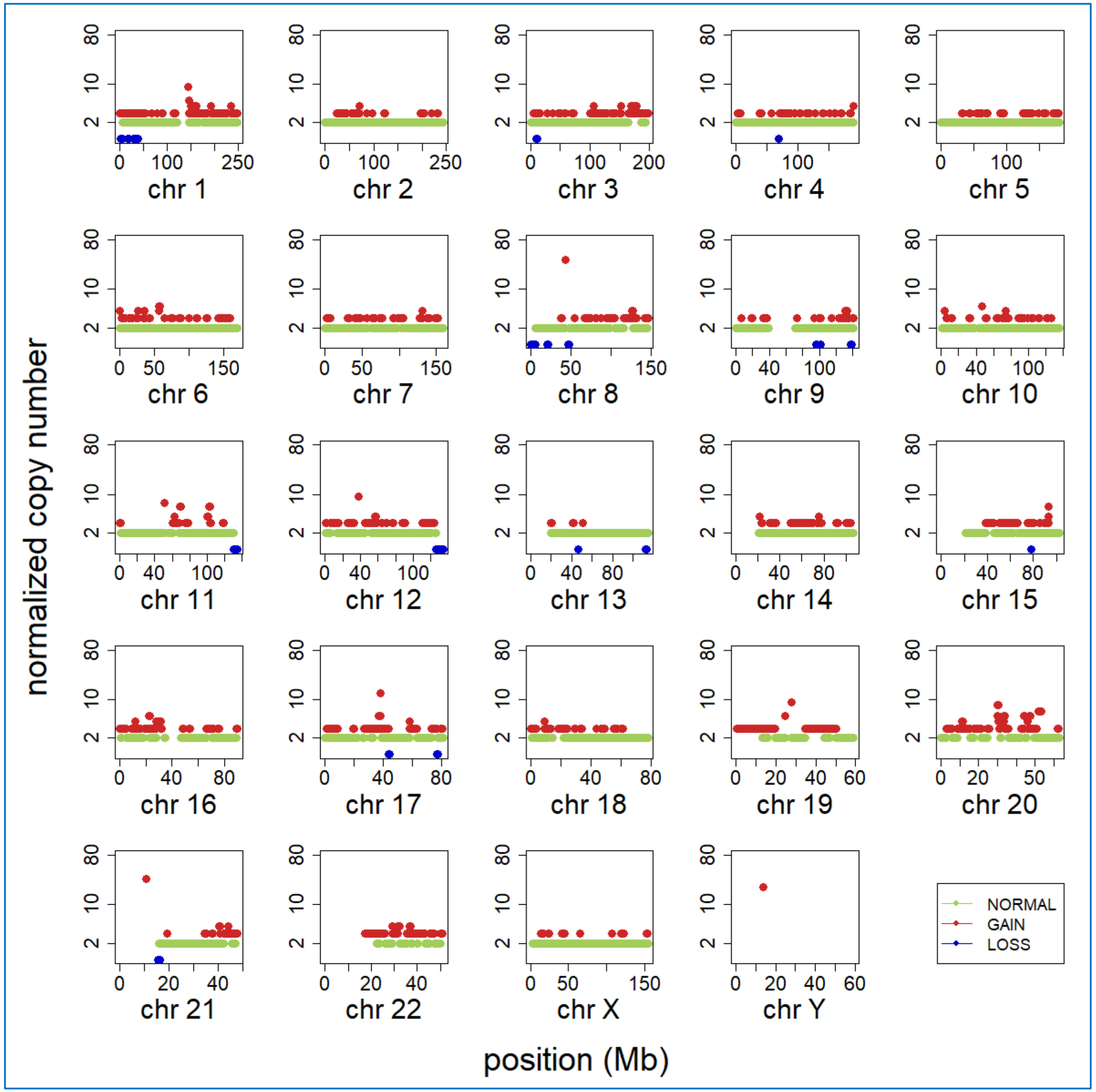

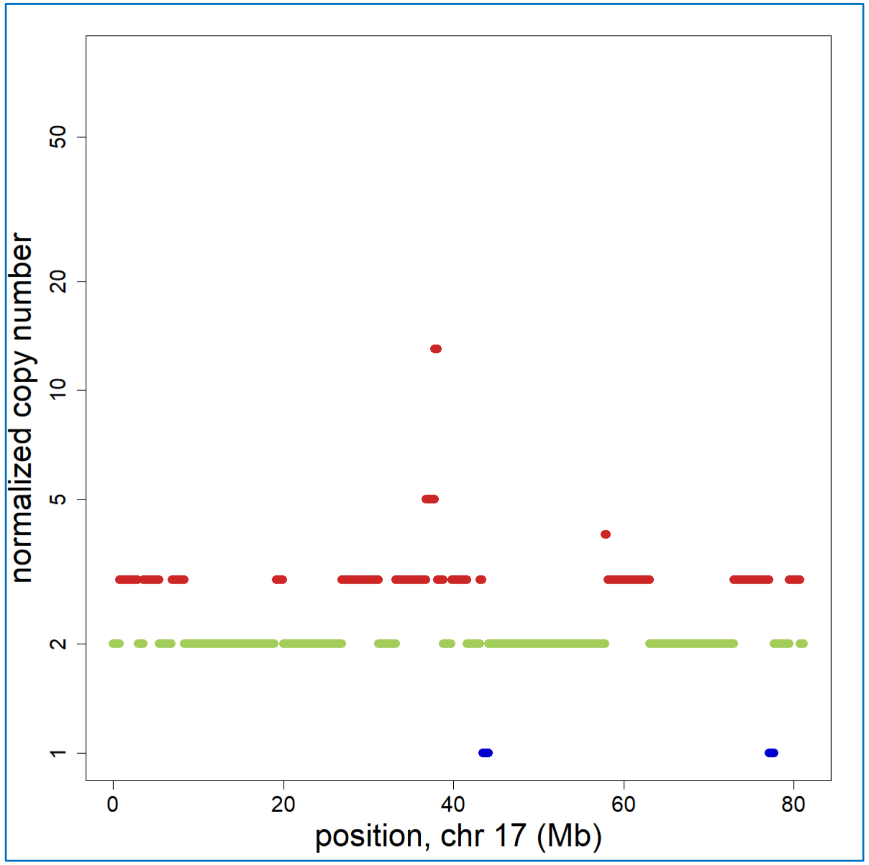

Control-FREEC

p-204

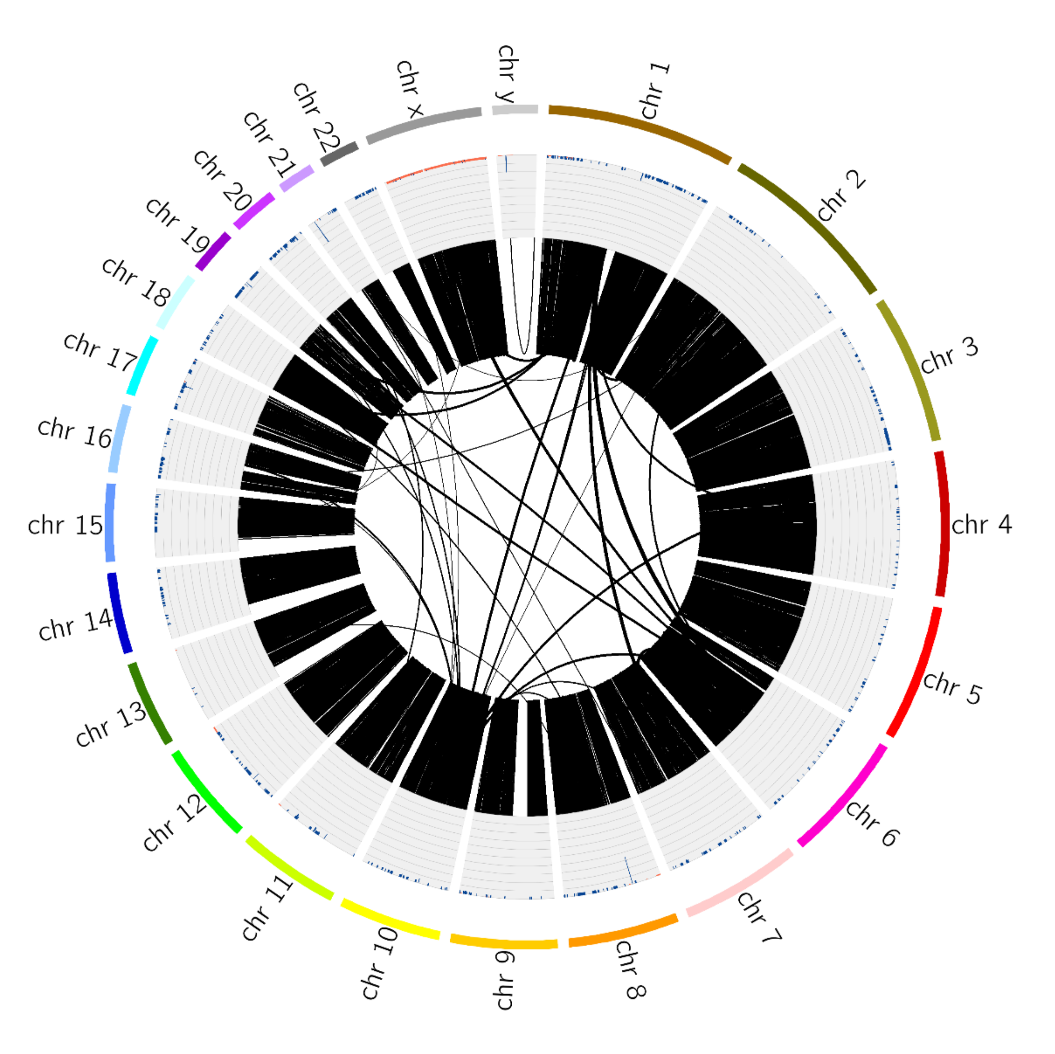

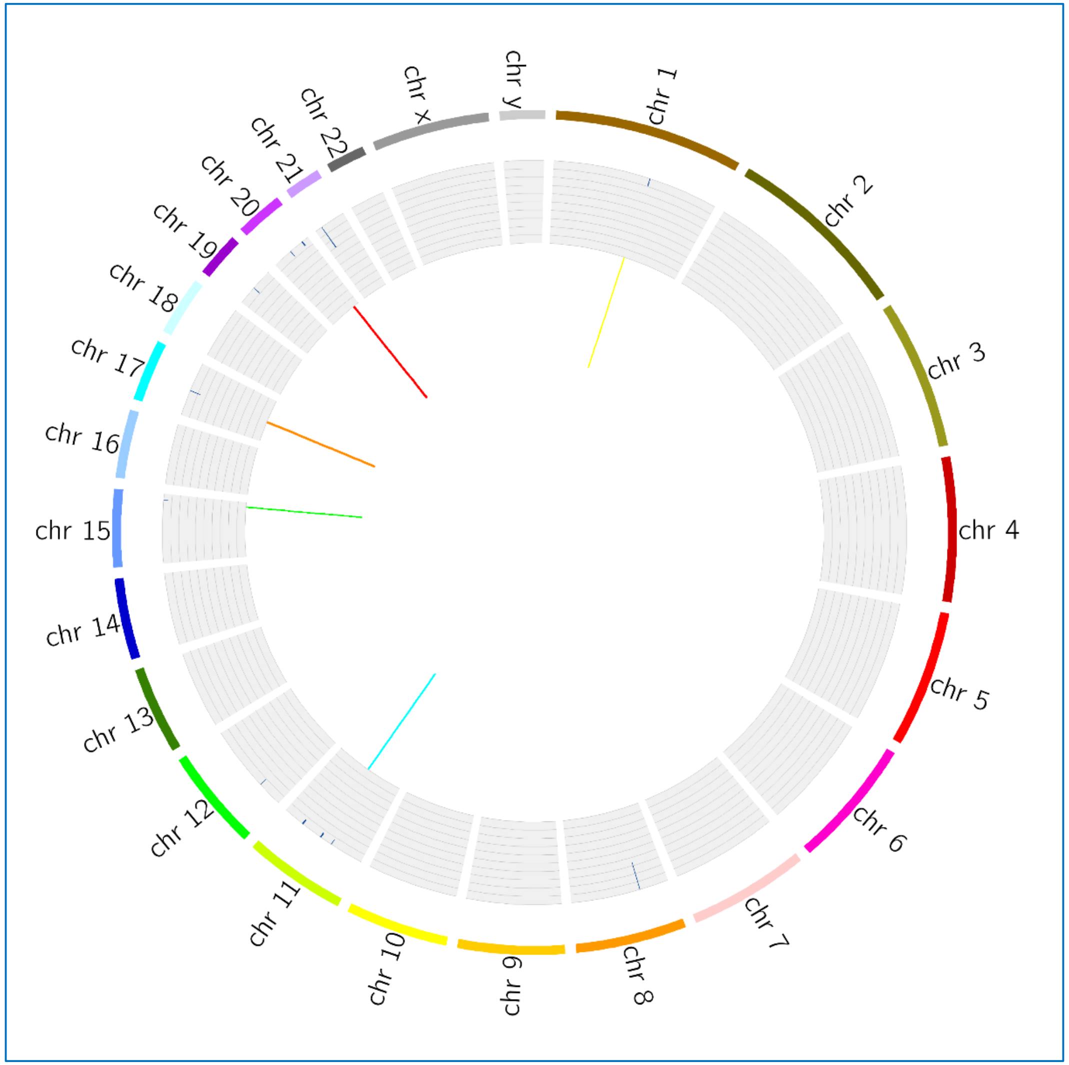

BreakDancer + Control-FREEC

FAST – Whole Genome

p-204

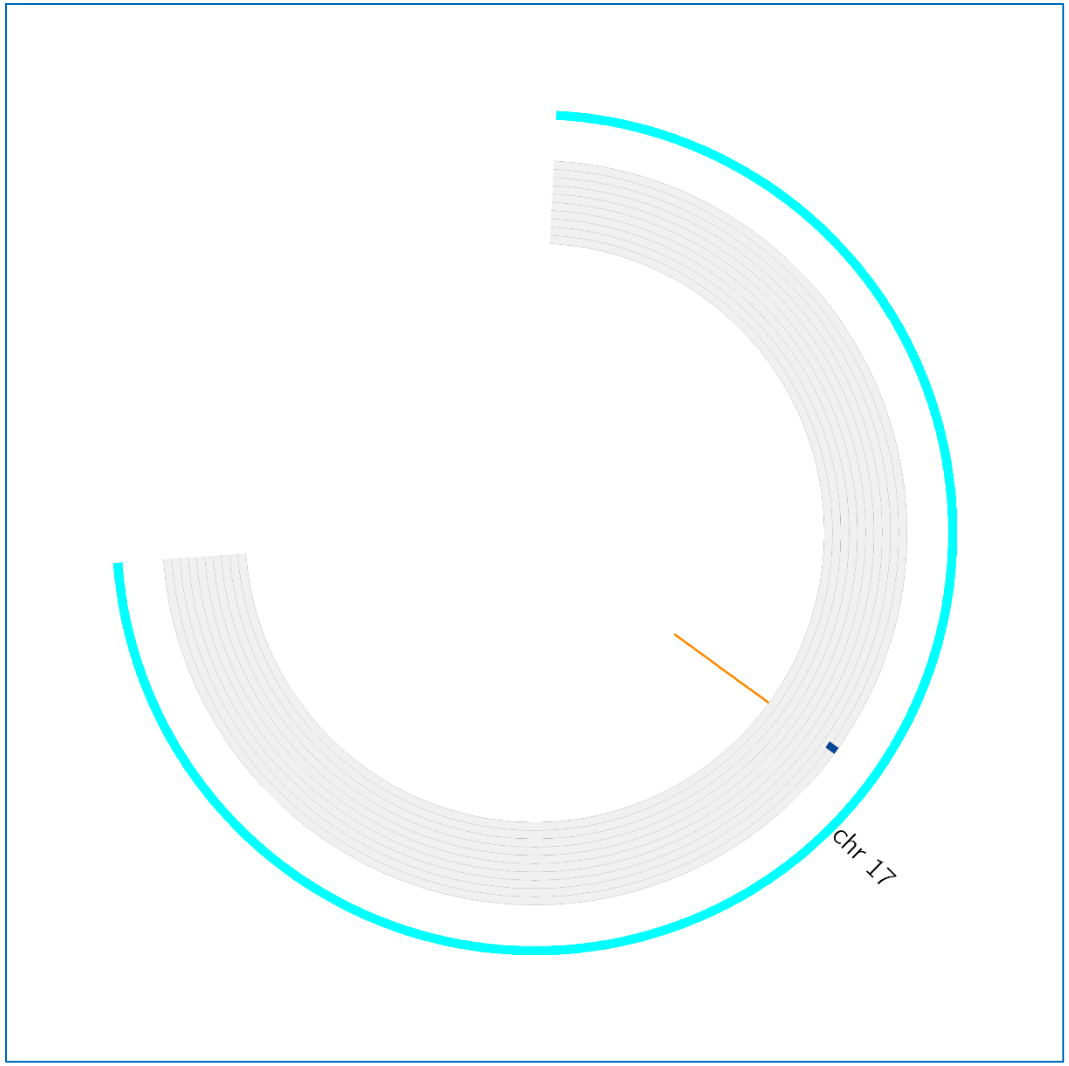

FAST – ERBB2 amplicon

p-205

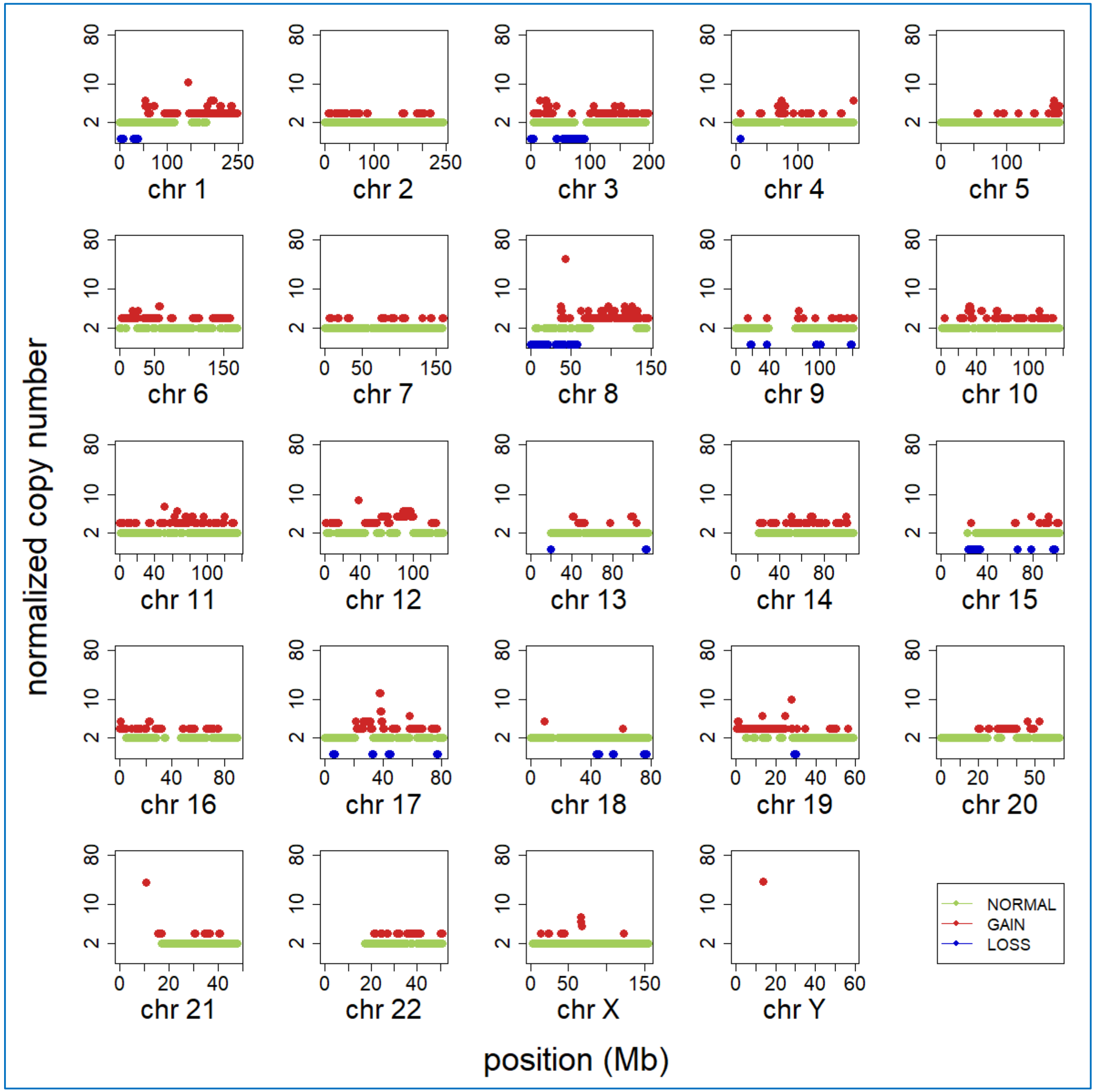

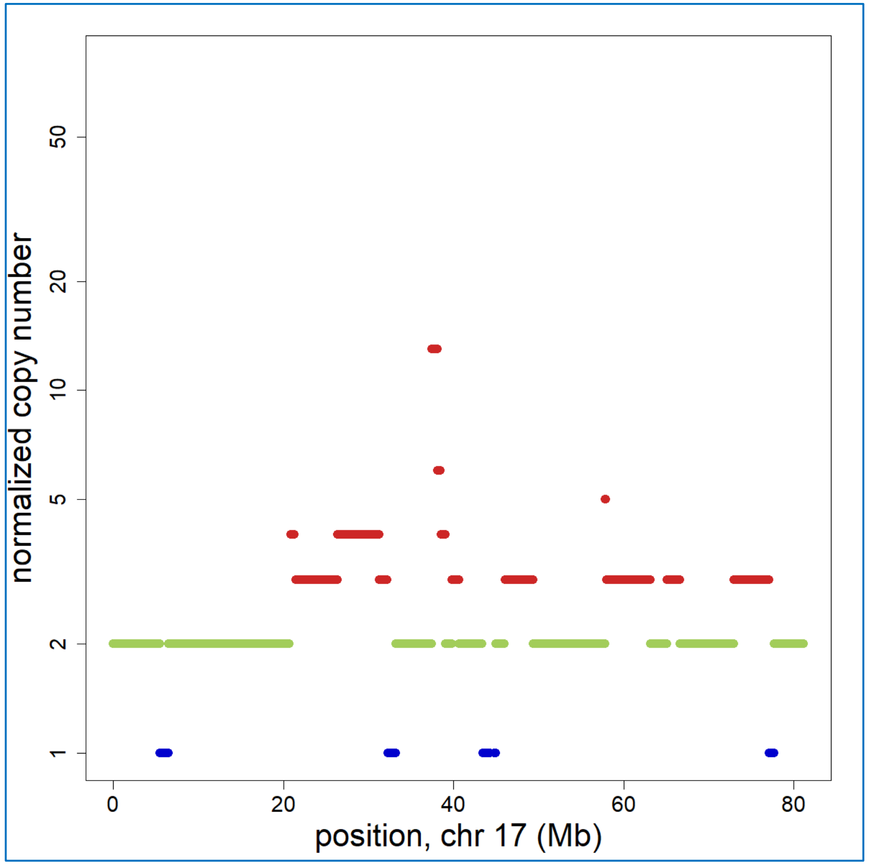

Control-FREEC

p-205

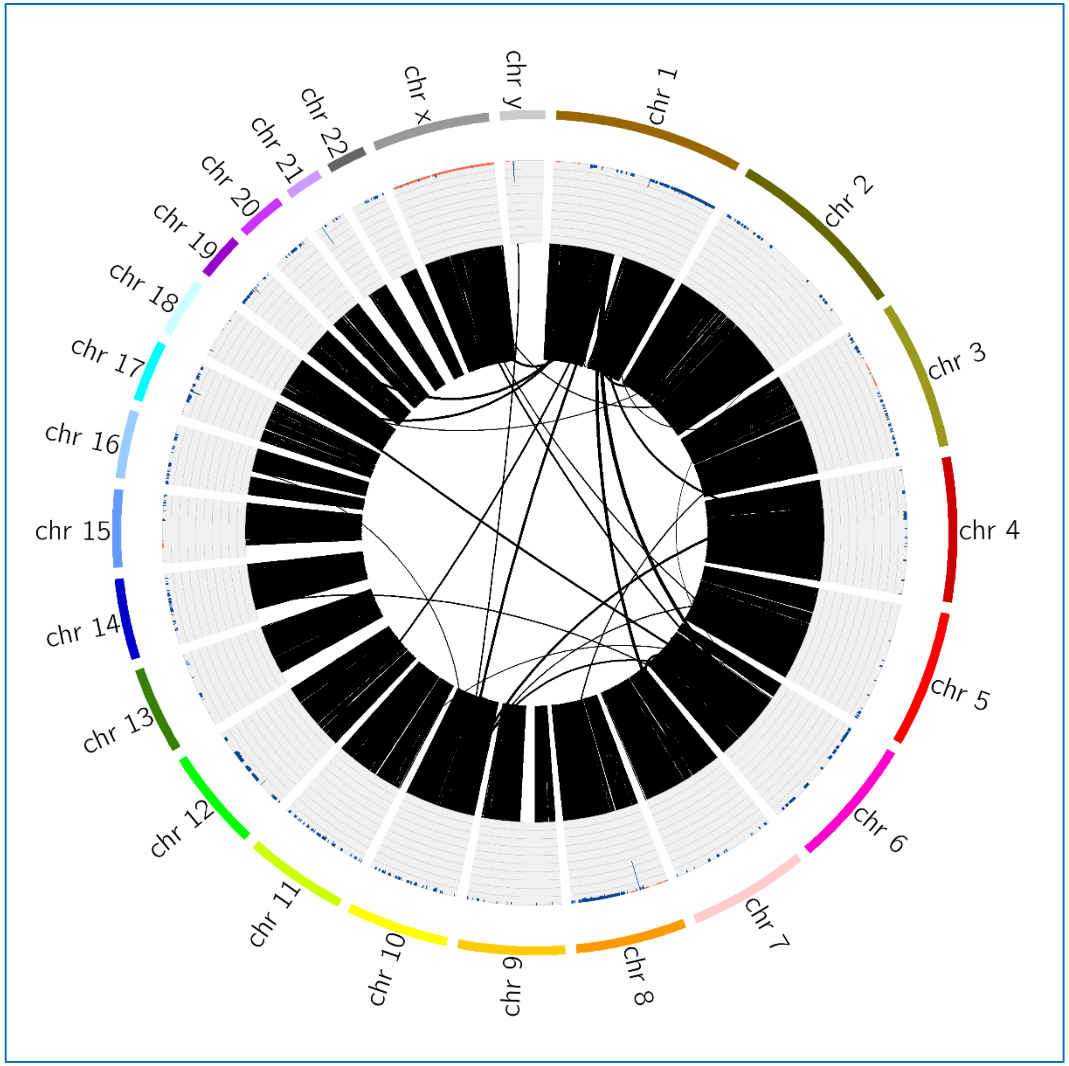

BreakDancer + Control-FREEC

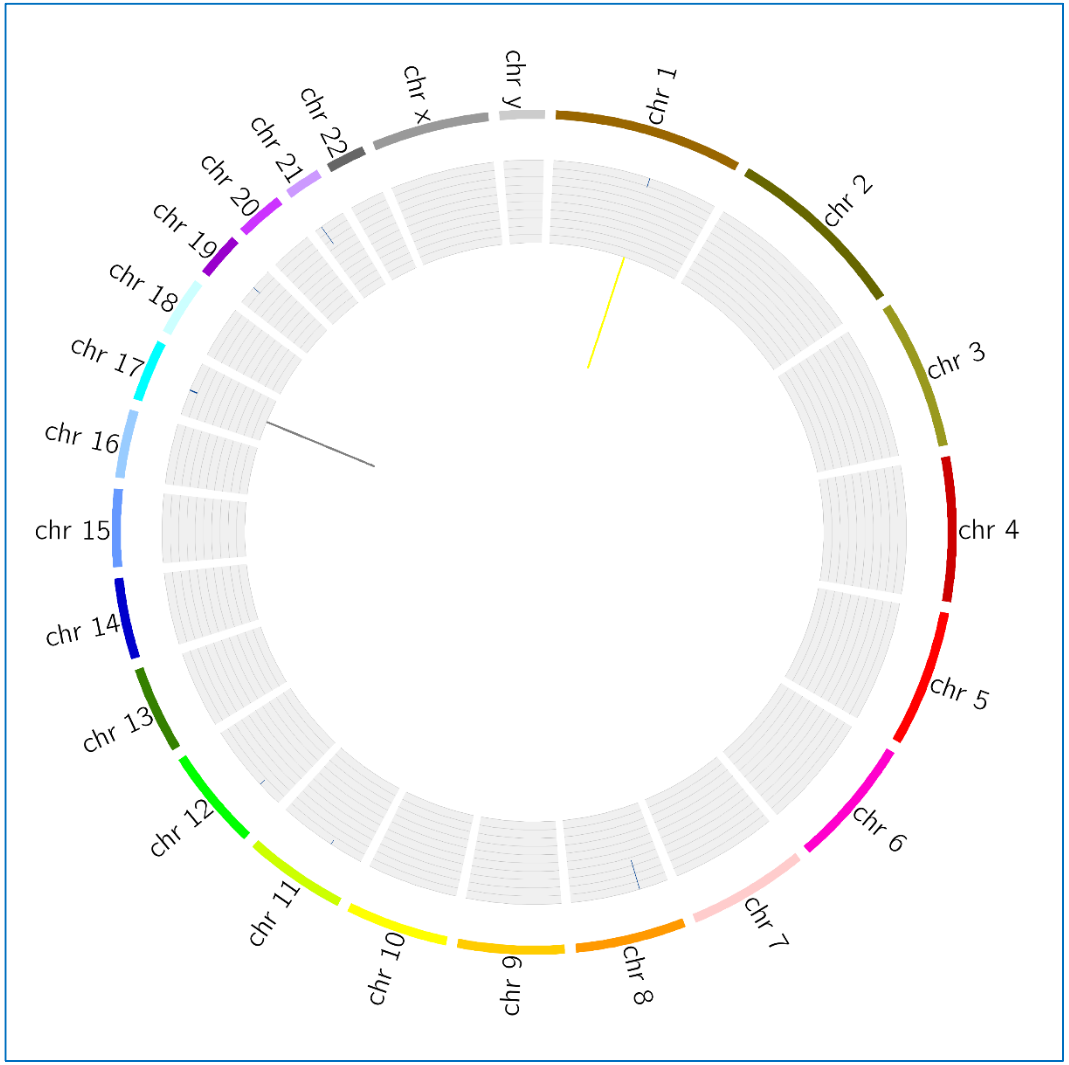

FAST – Whole Genome

p-205

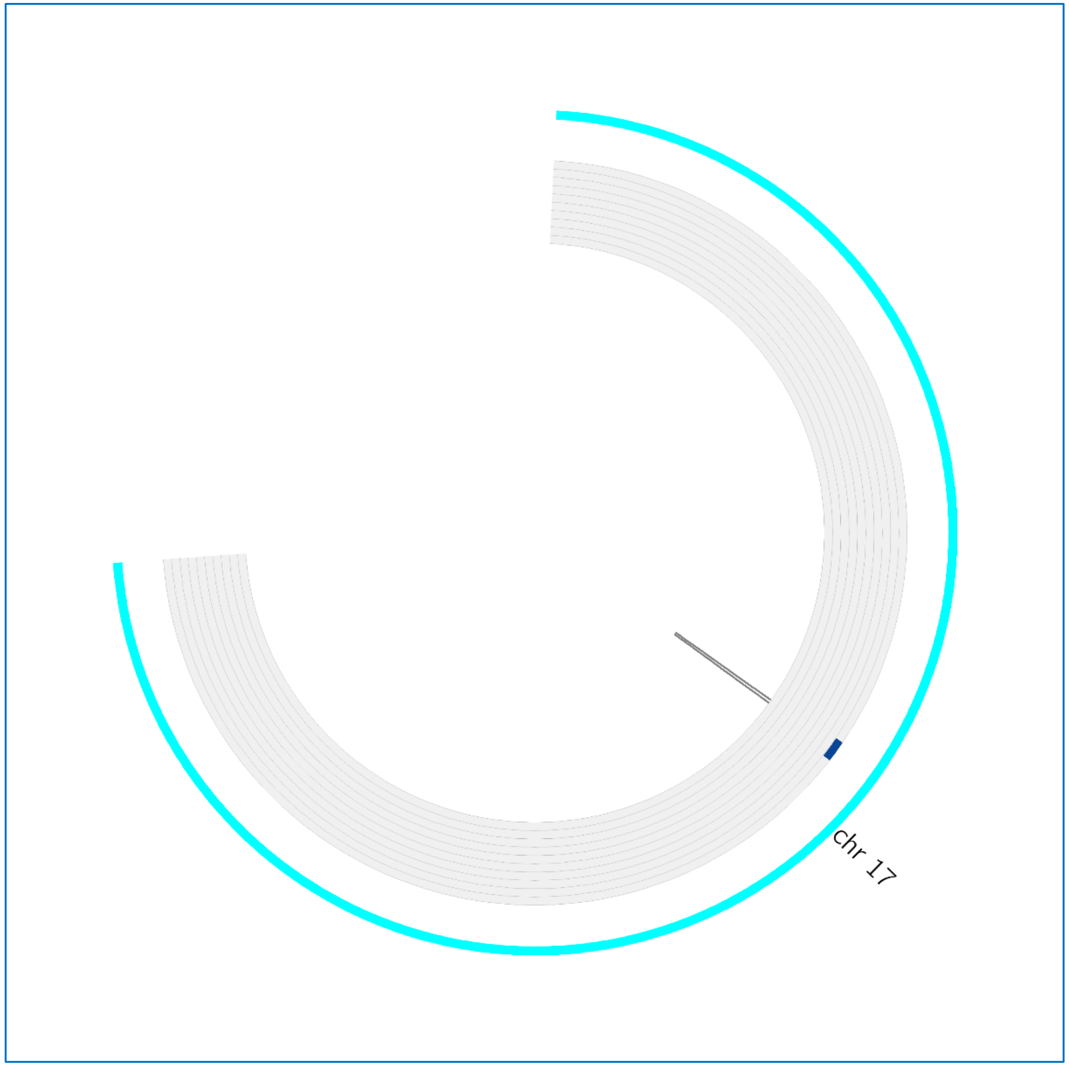

FAST – ERBB2 amplicon

p-209

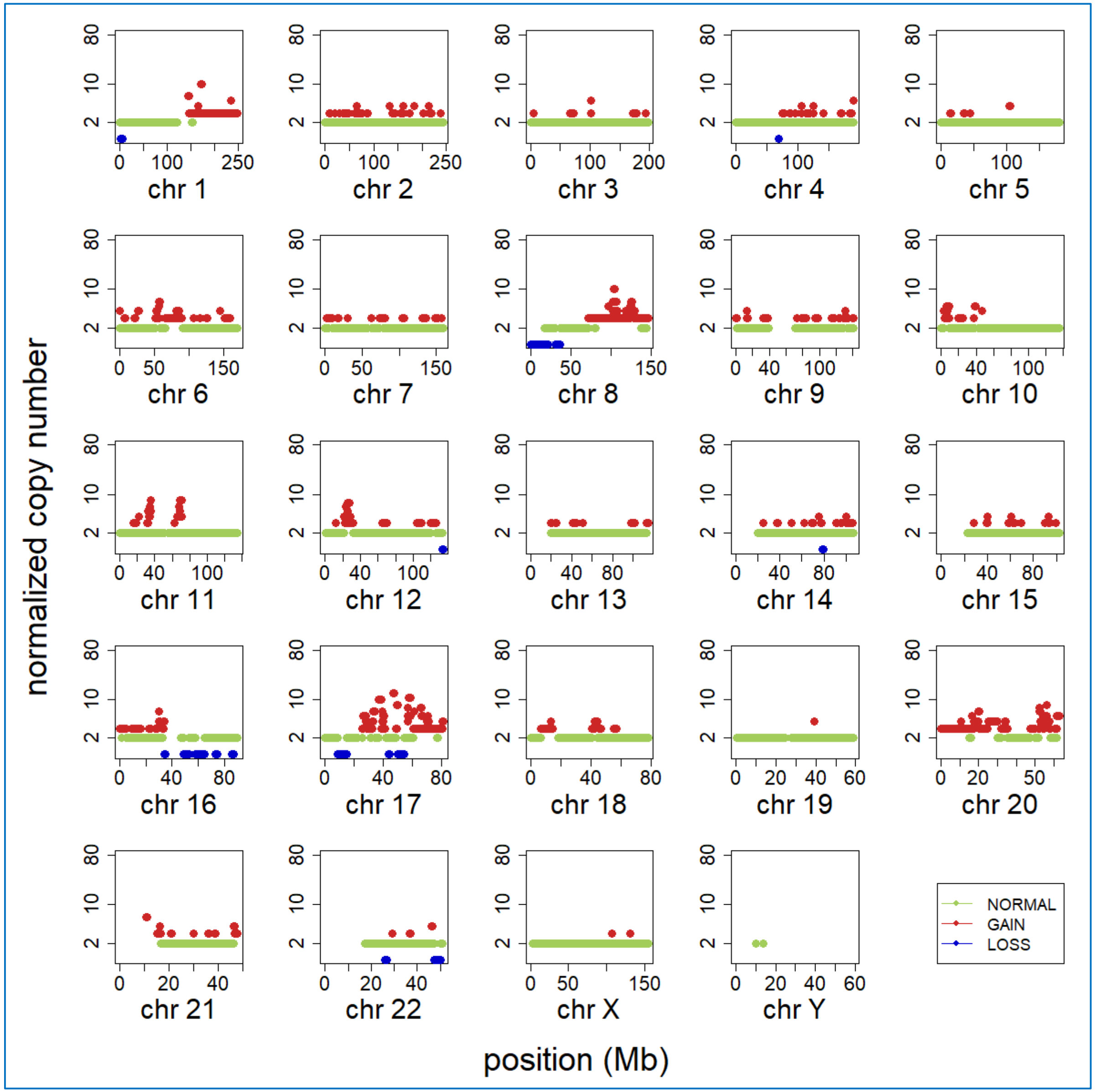

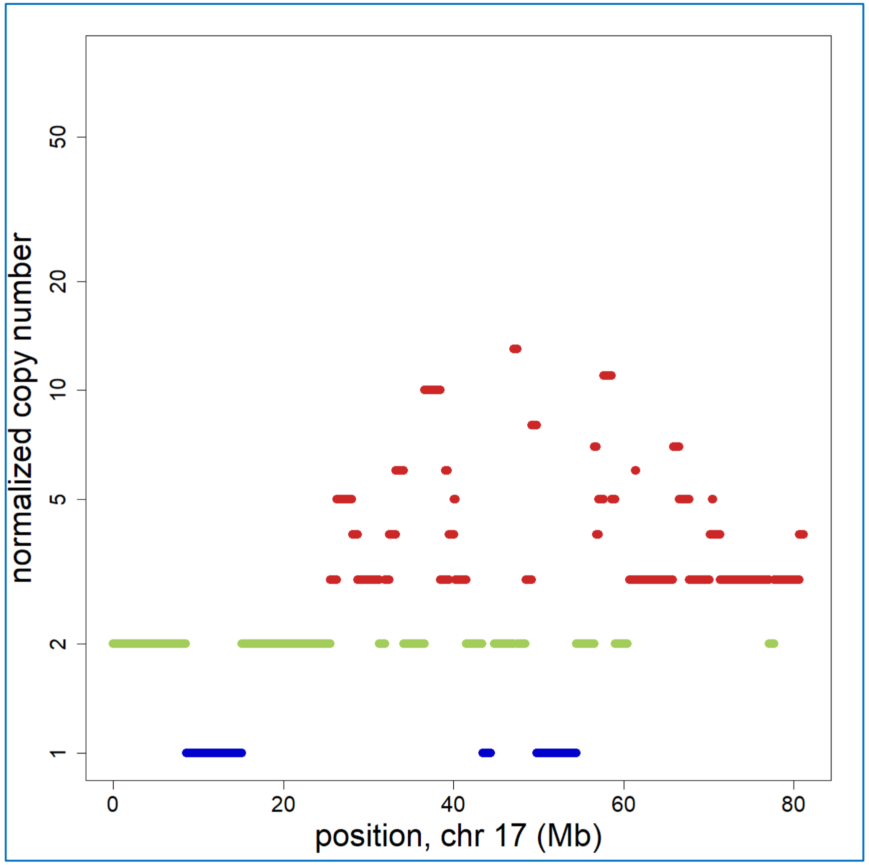

Control-FREEC

p-209

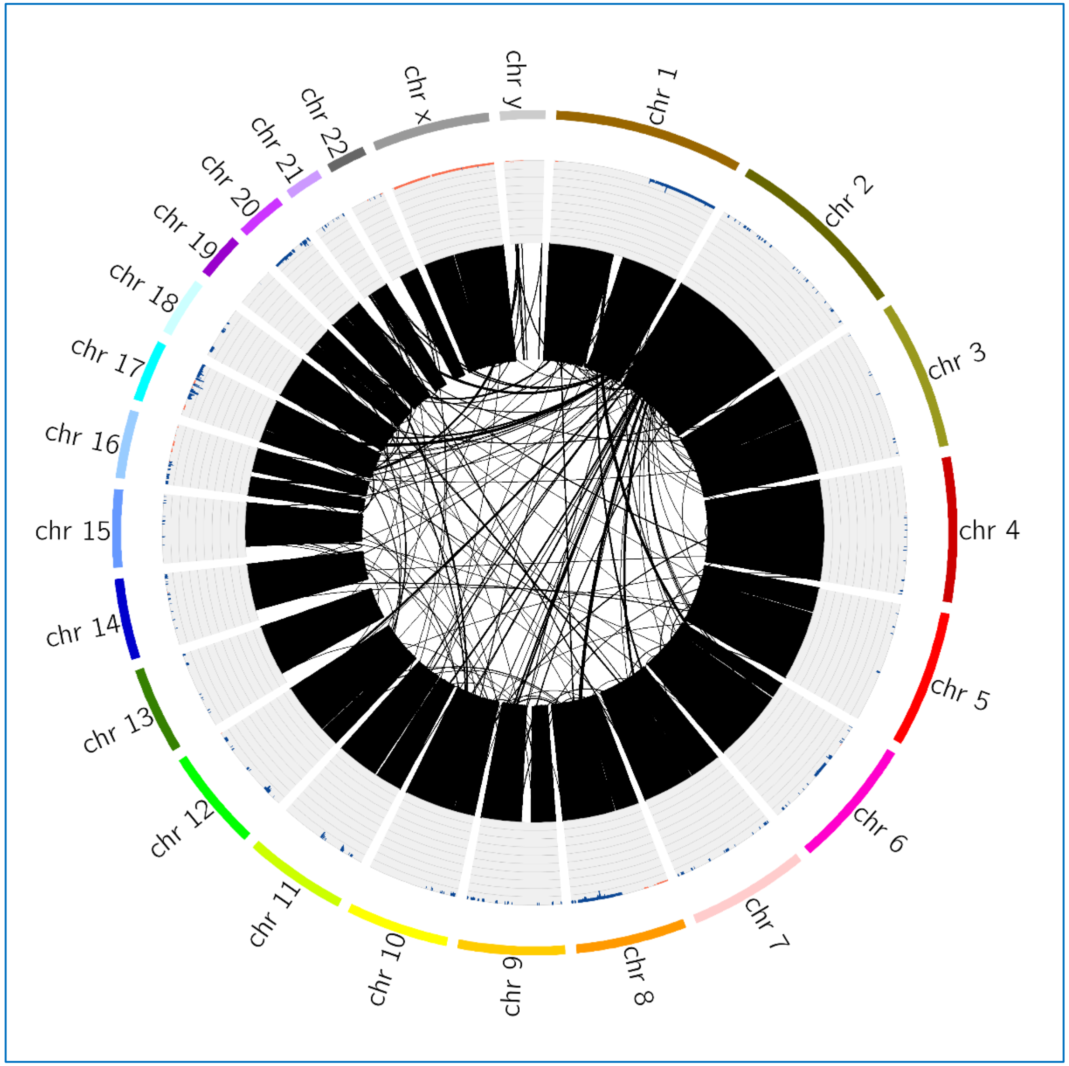

BreakDancer + Control-FREEC

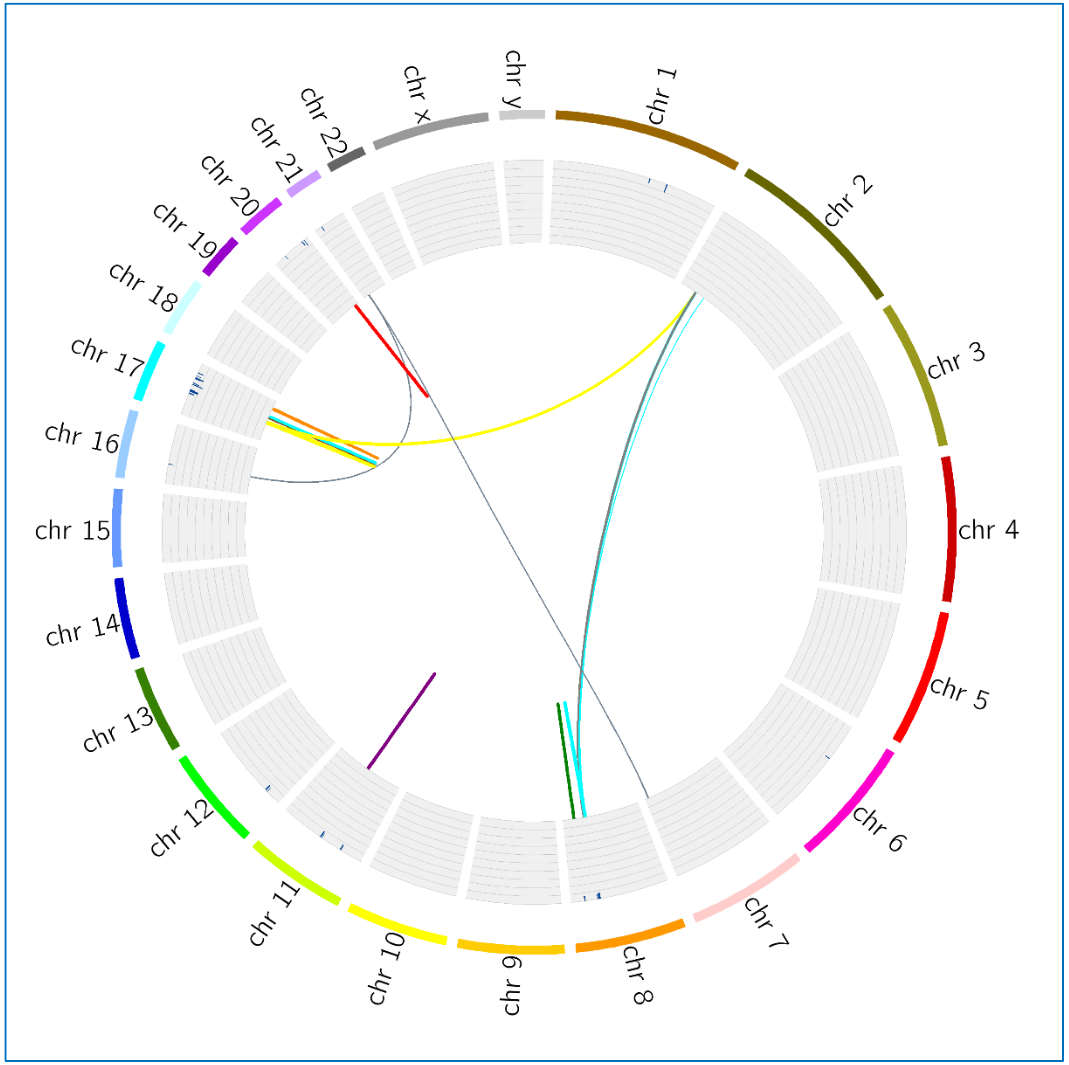

FAST – Whole Genome

p-209

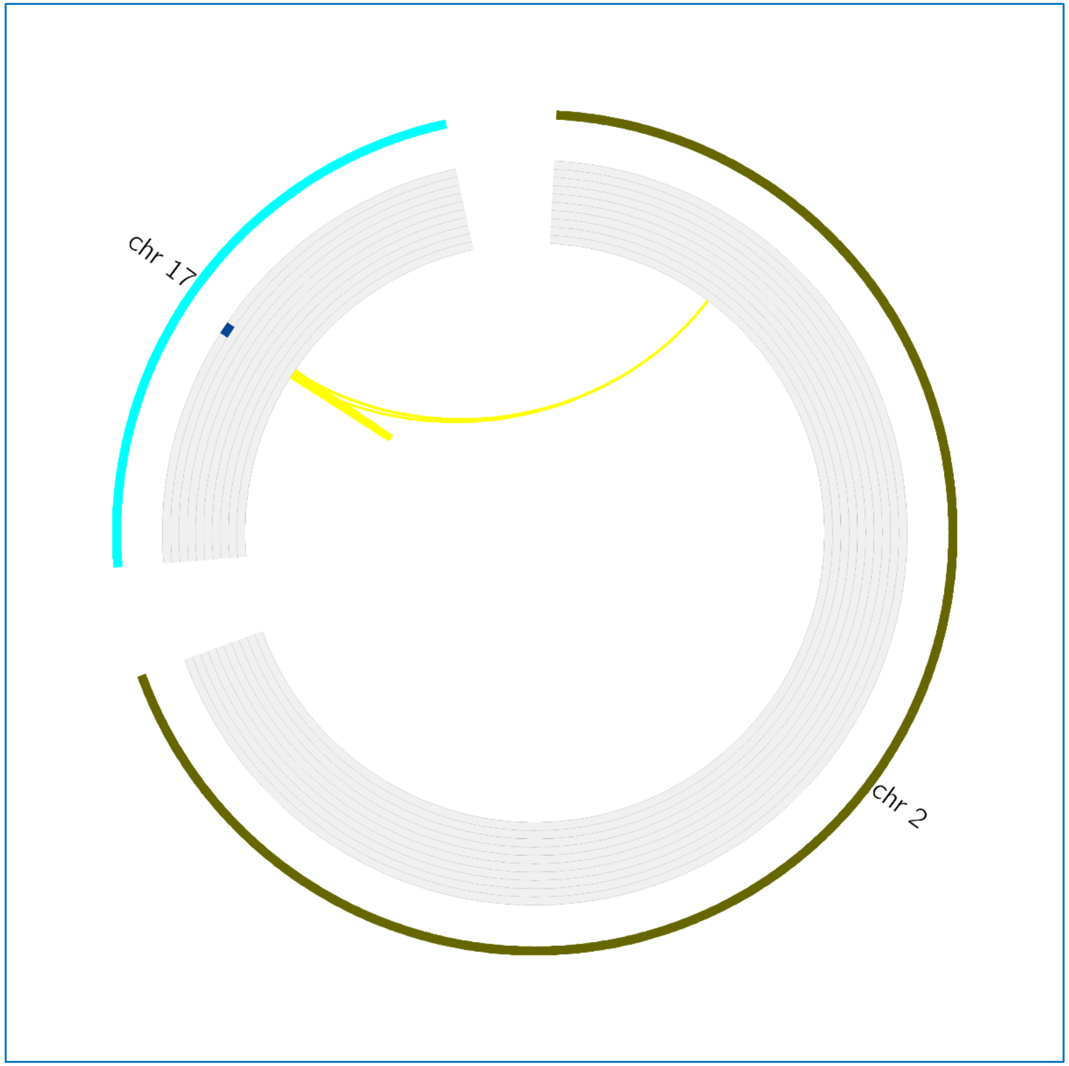

FAST – ERBB2 amplicon

p-214

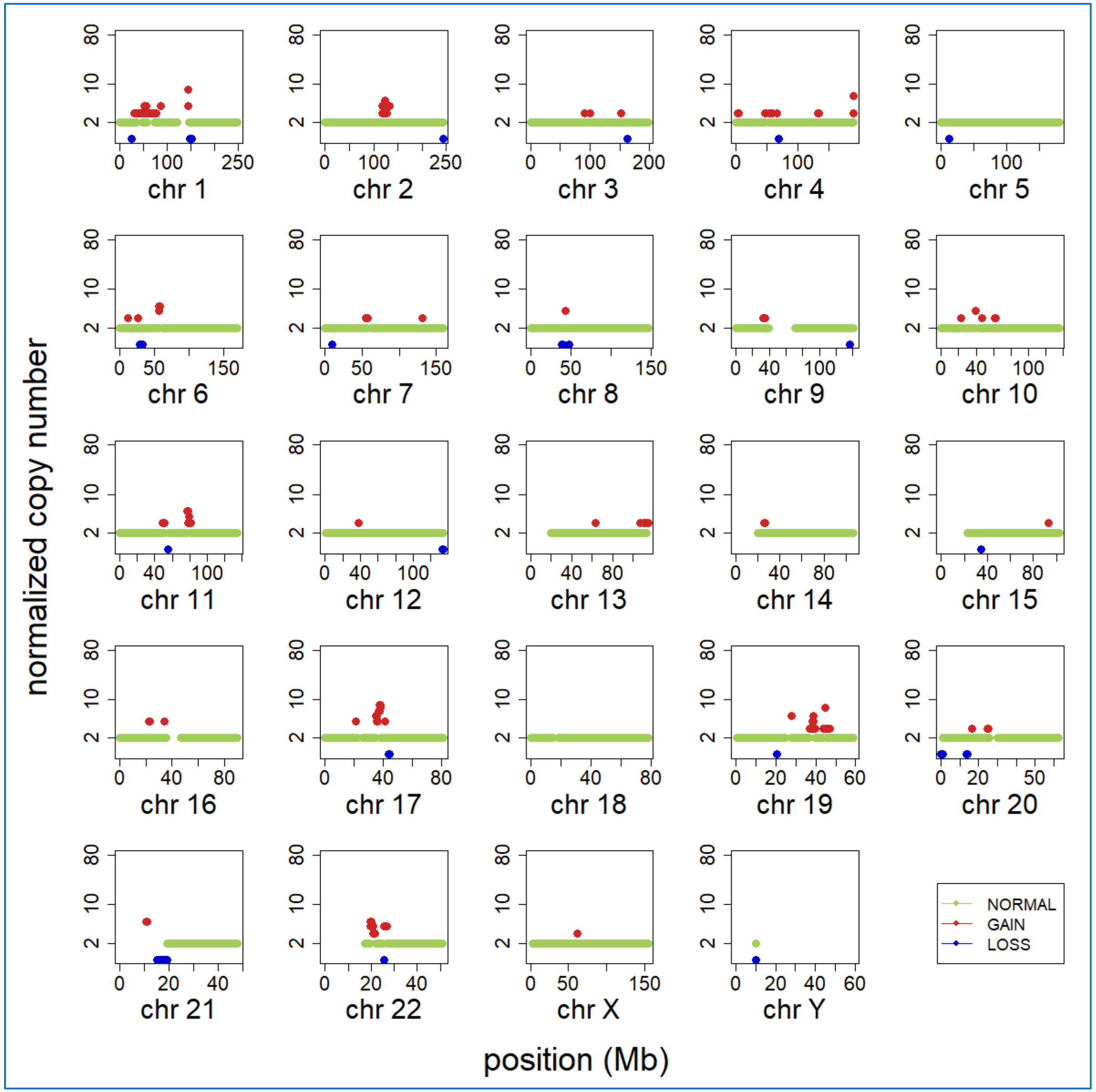

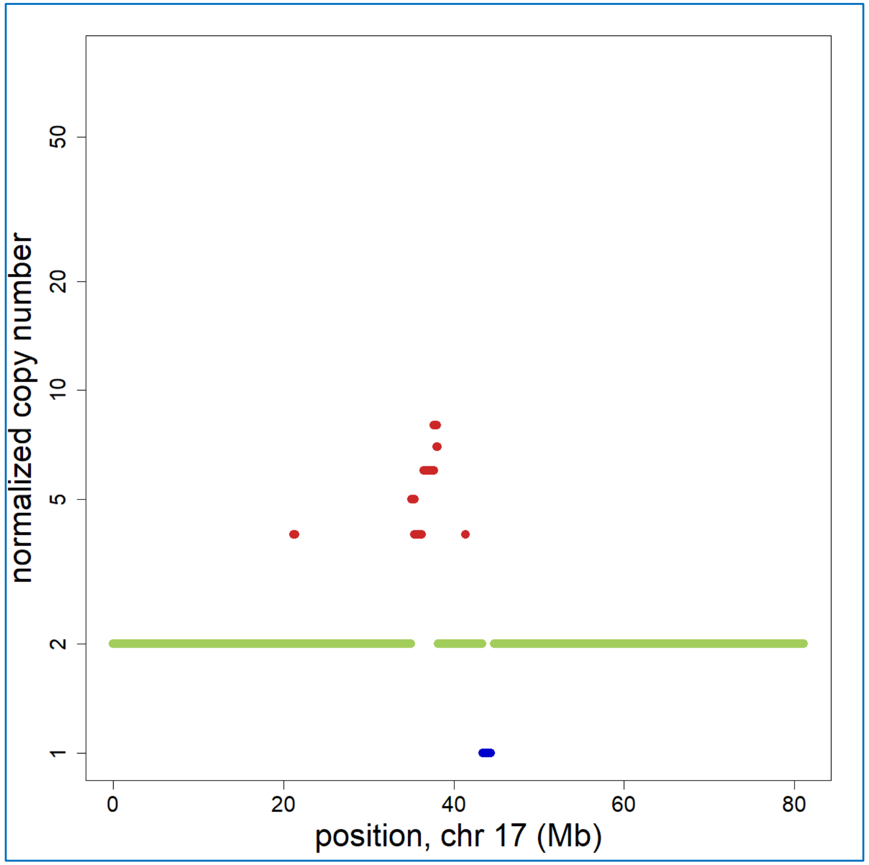

Control-FREEC

p-214

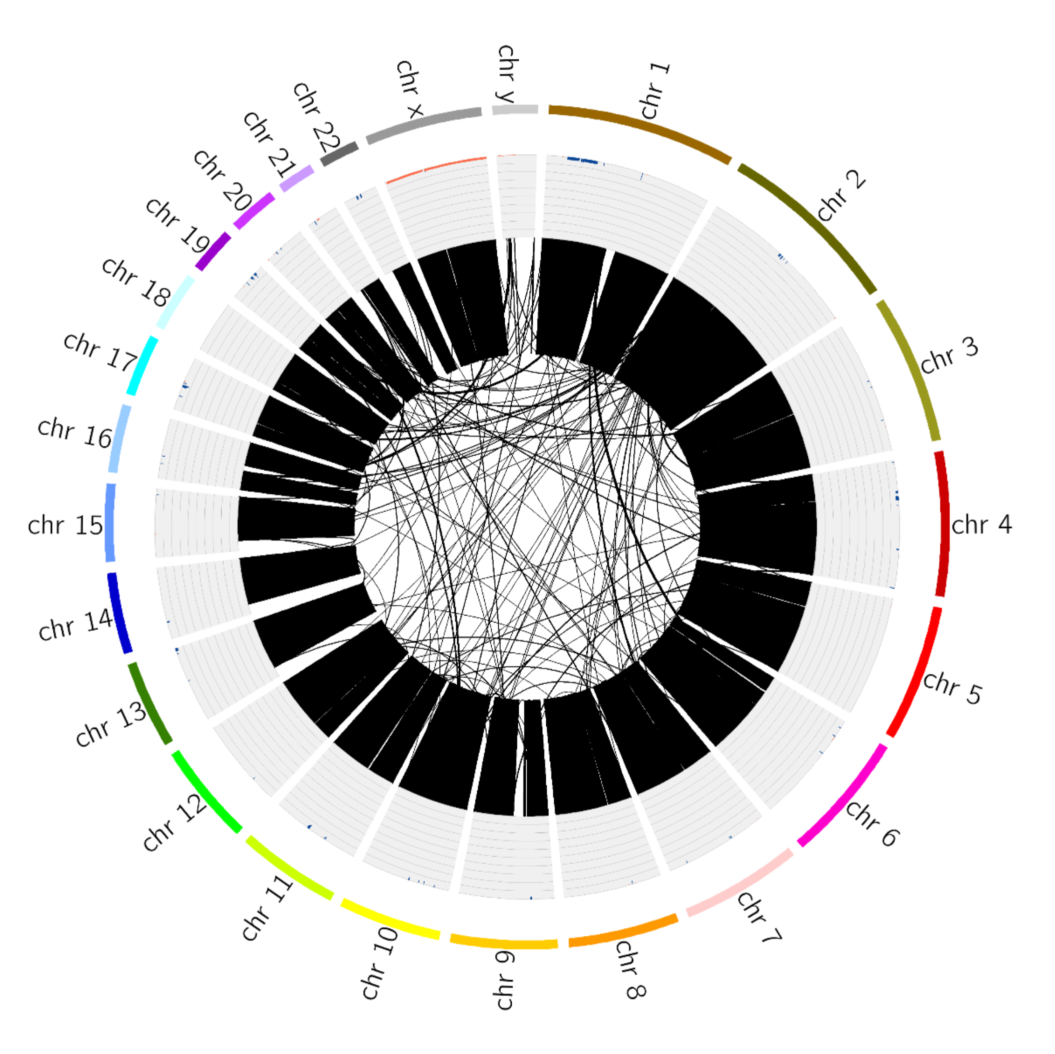

BreakDancer + Control-FREEC

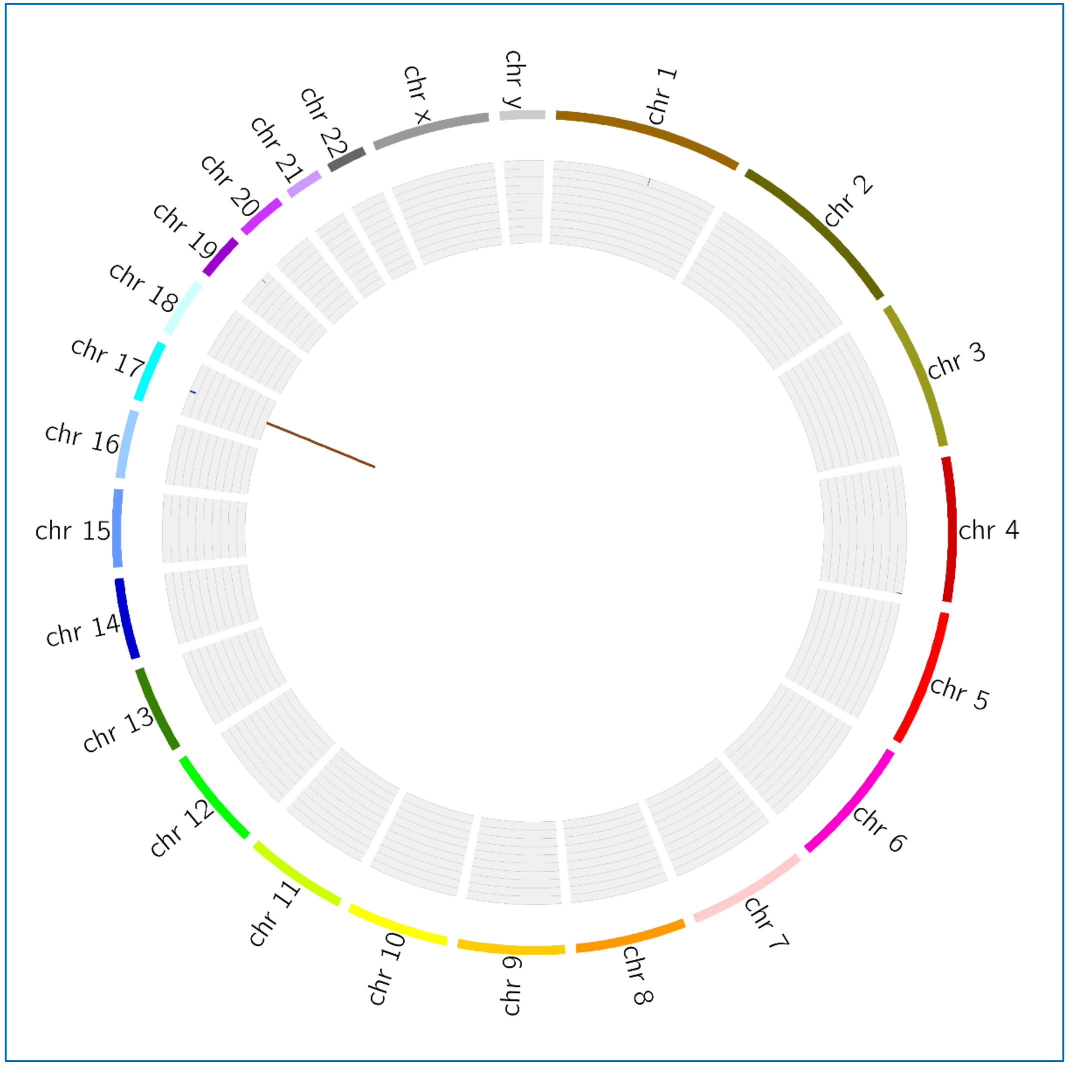

FAST – Whole Genome

p-214

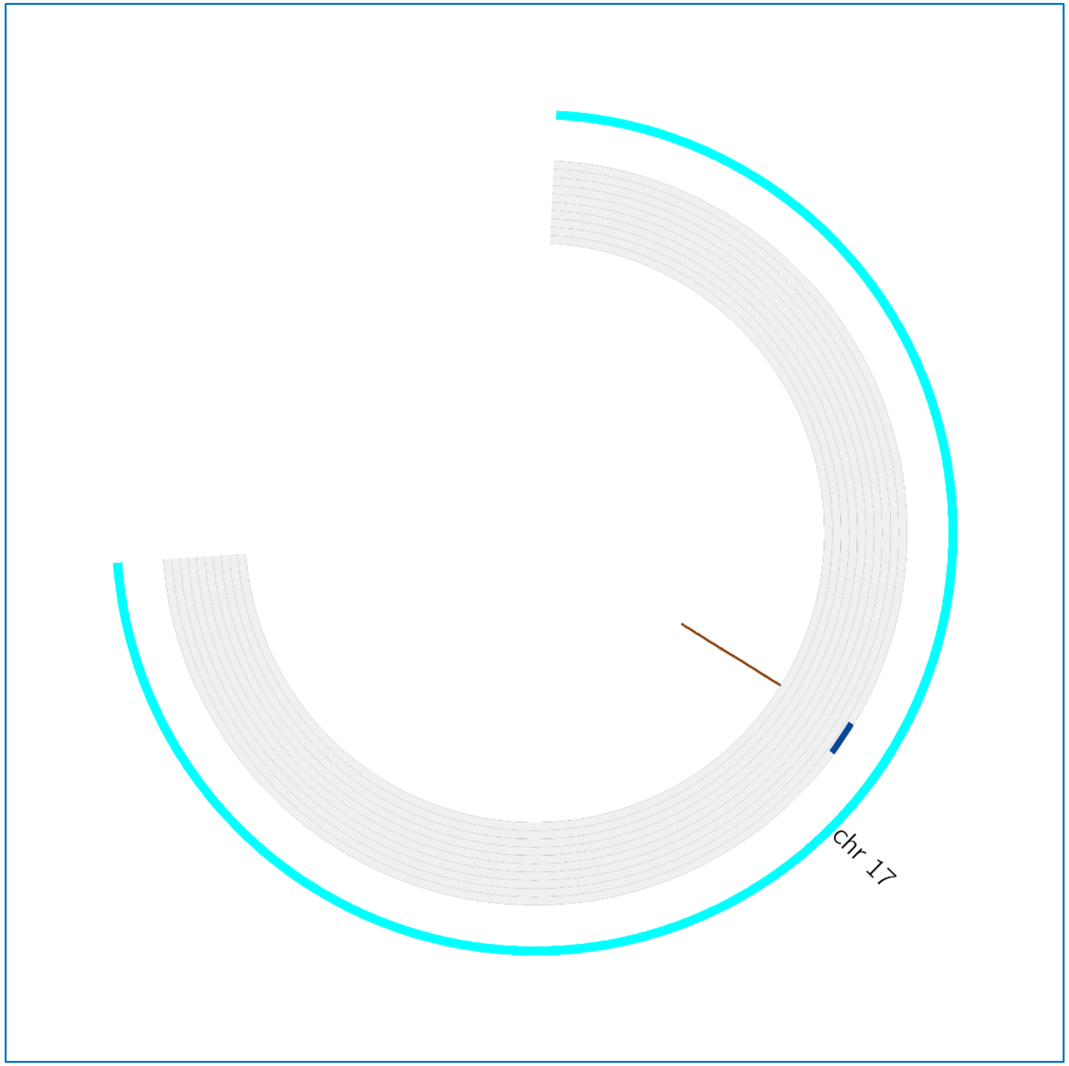

FAST – ERBB2 amplicon

p-215

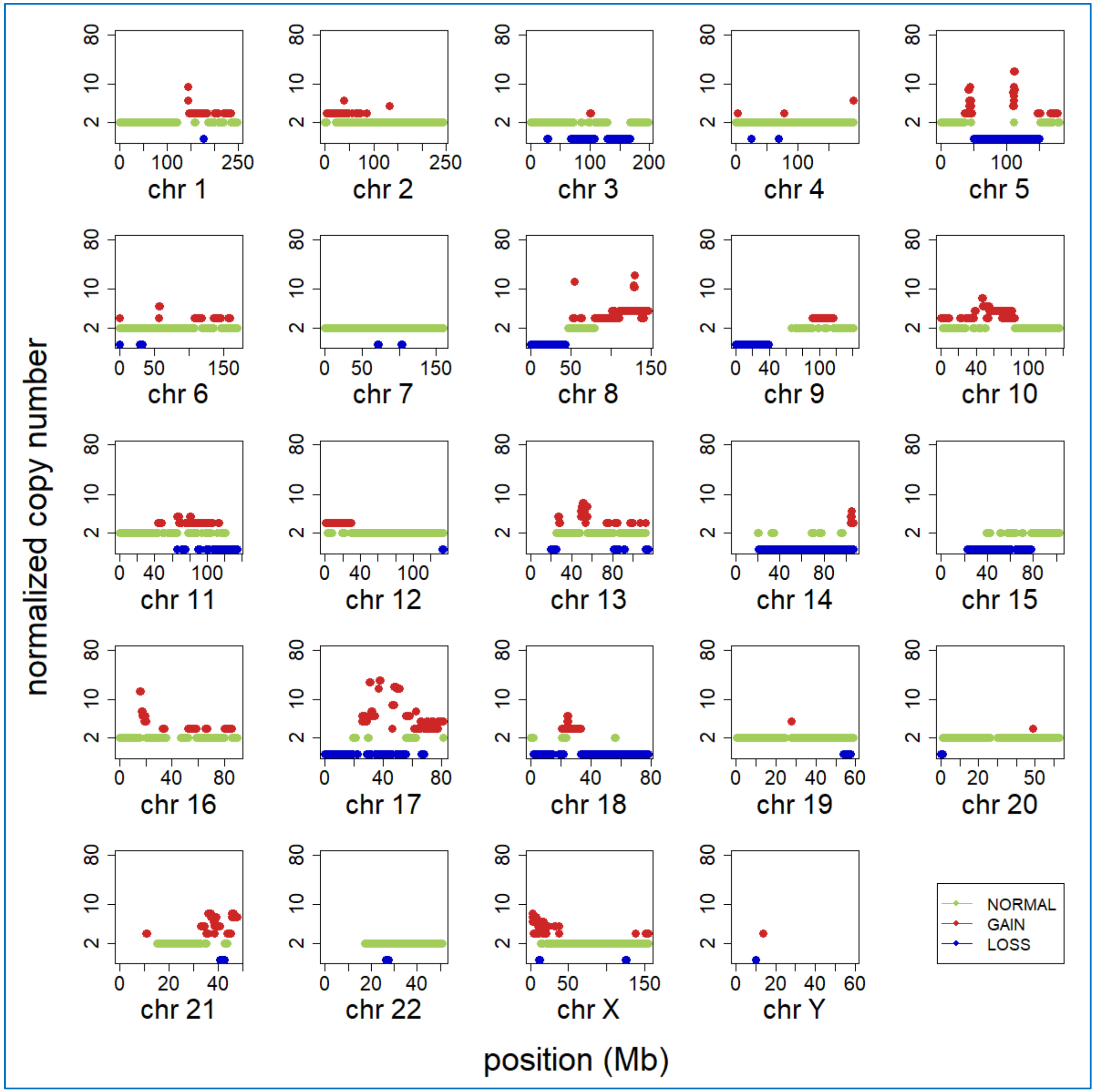

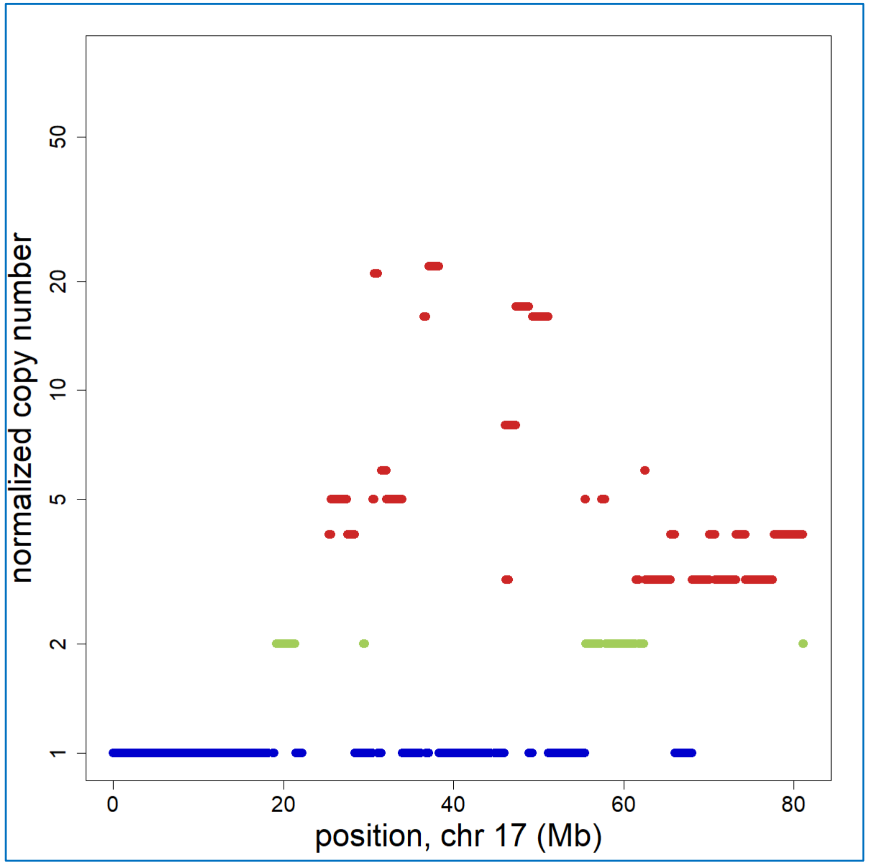

Control-FREEC

p-215

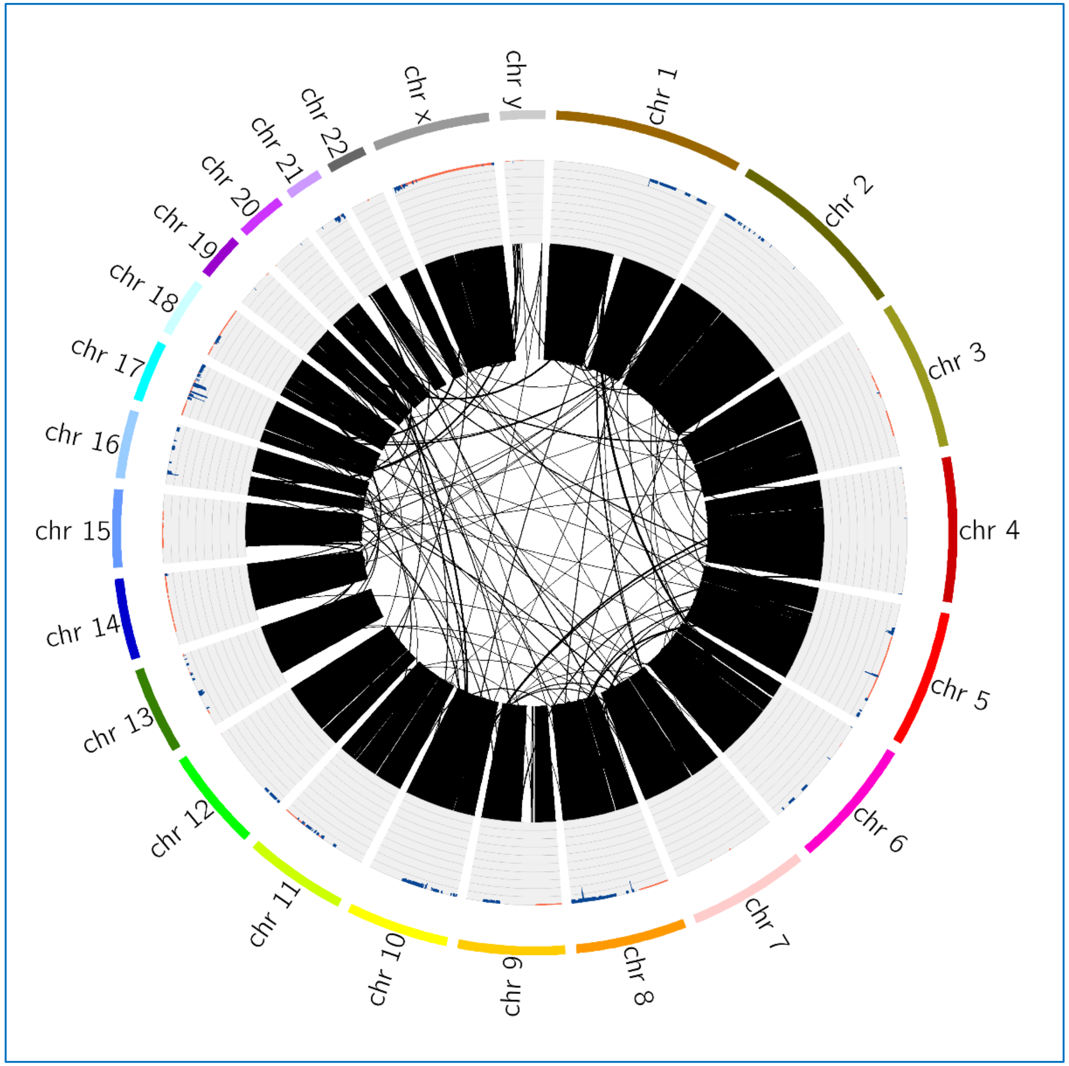

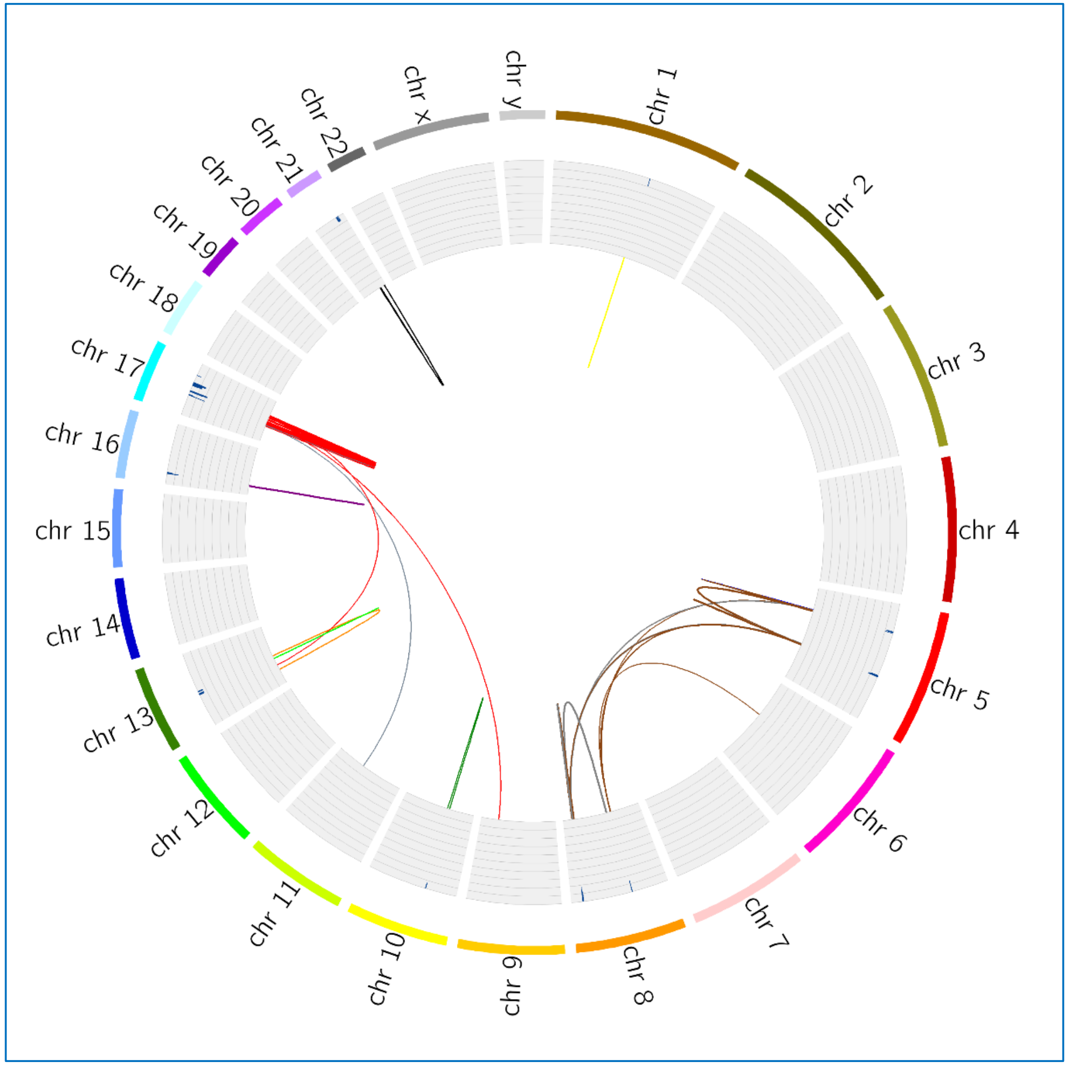

BreakDancer + Control-FREEC

FAST – Whole Genome

p-215

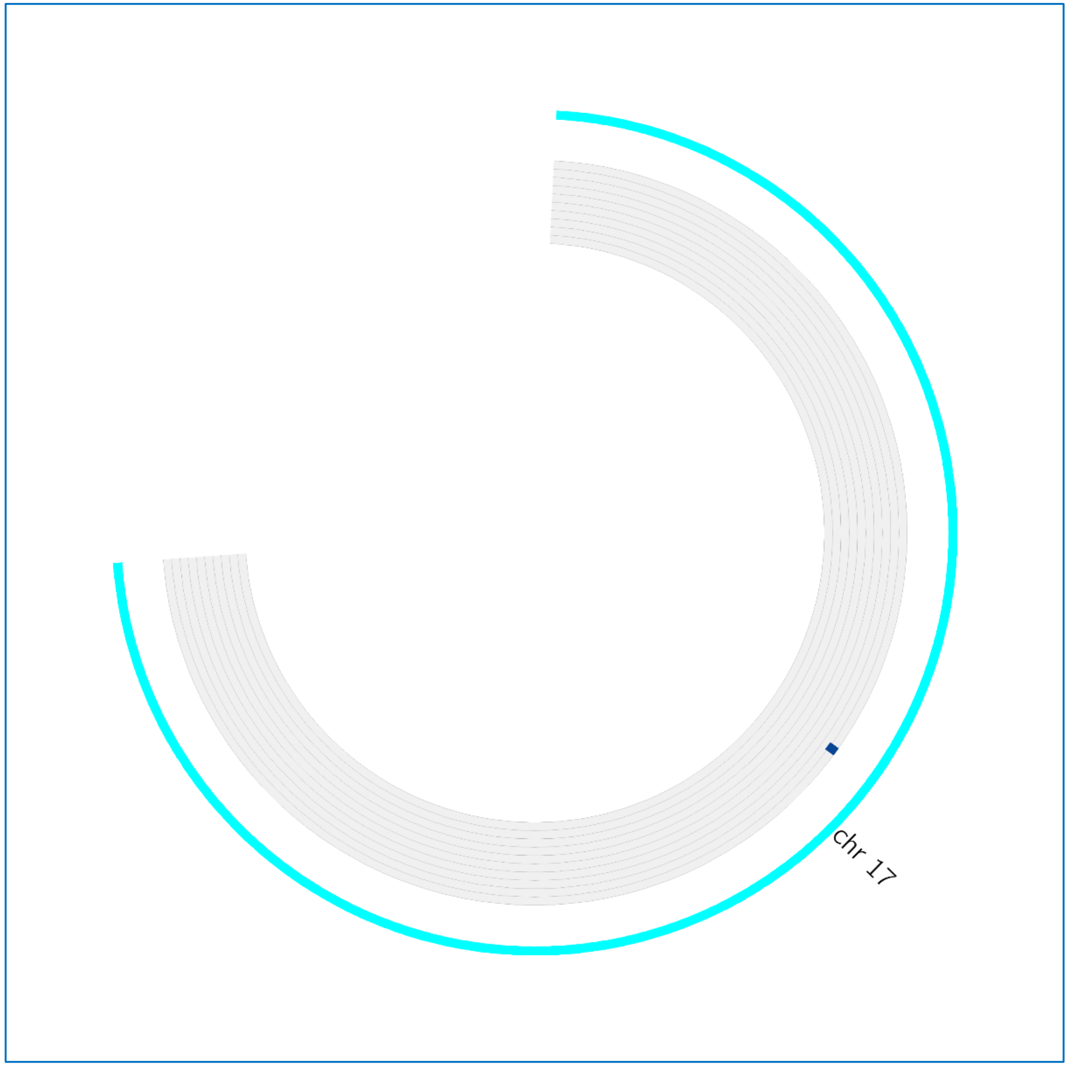

FAST – ERBB2 amplicon

p-216

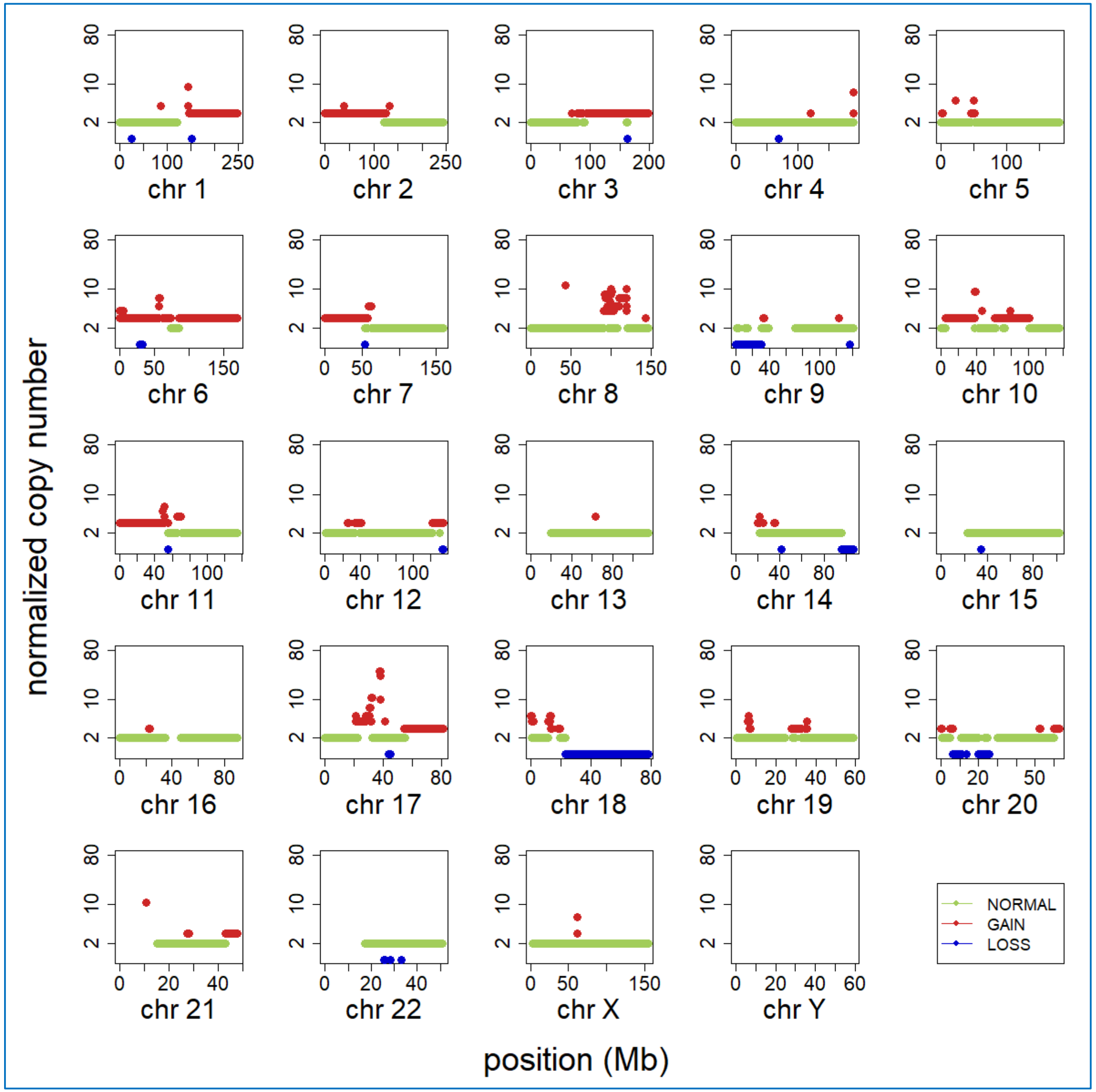

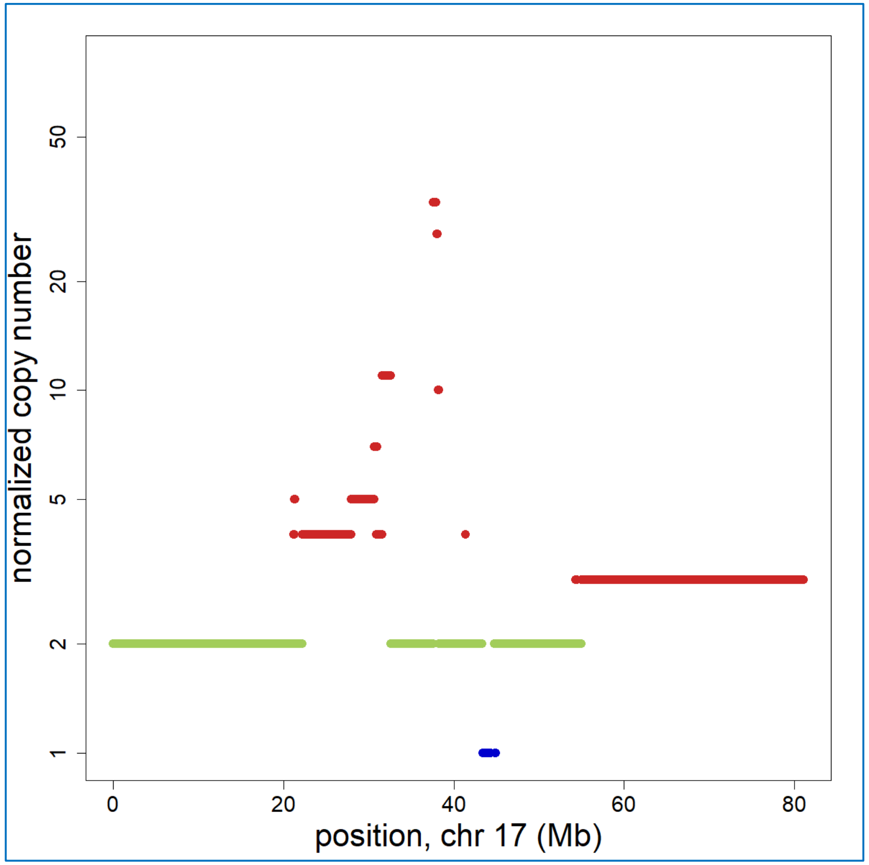

Control-FREEC

p-216

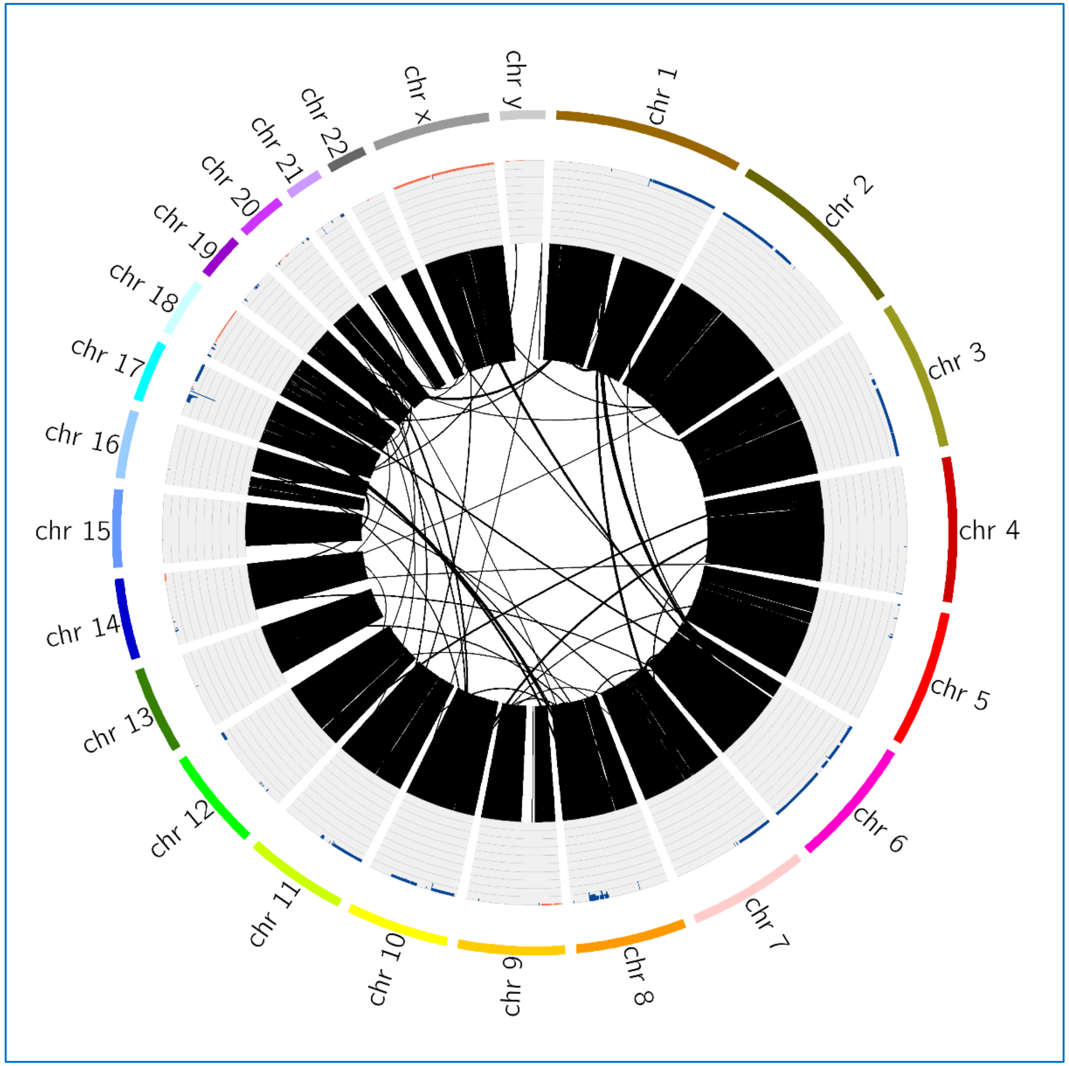

BreakDancer + Control-FREEC

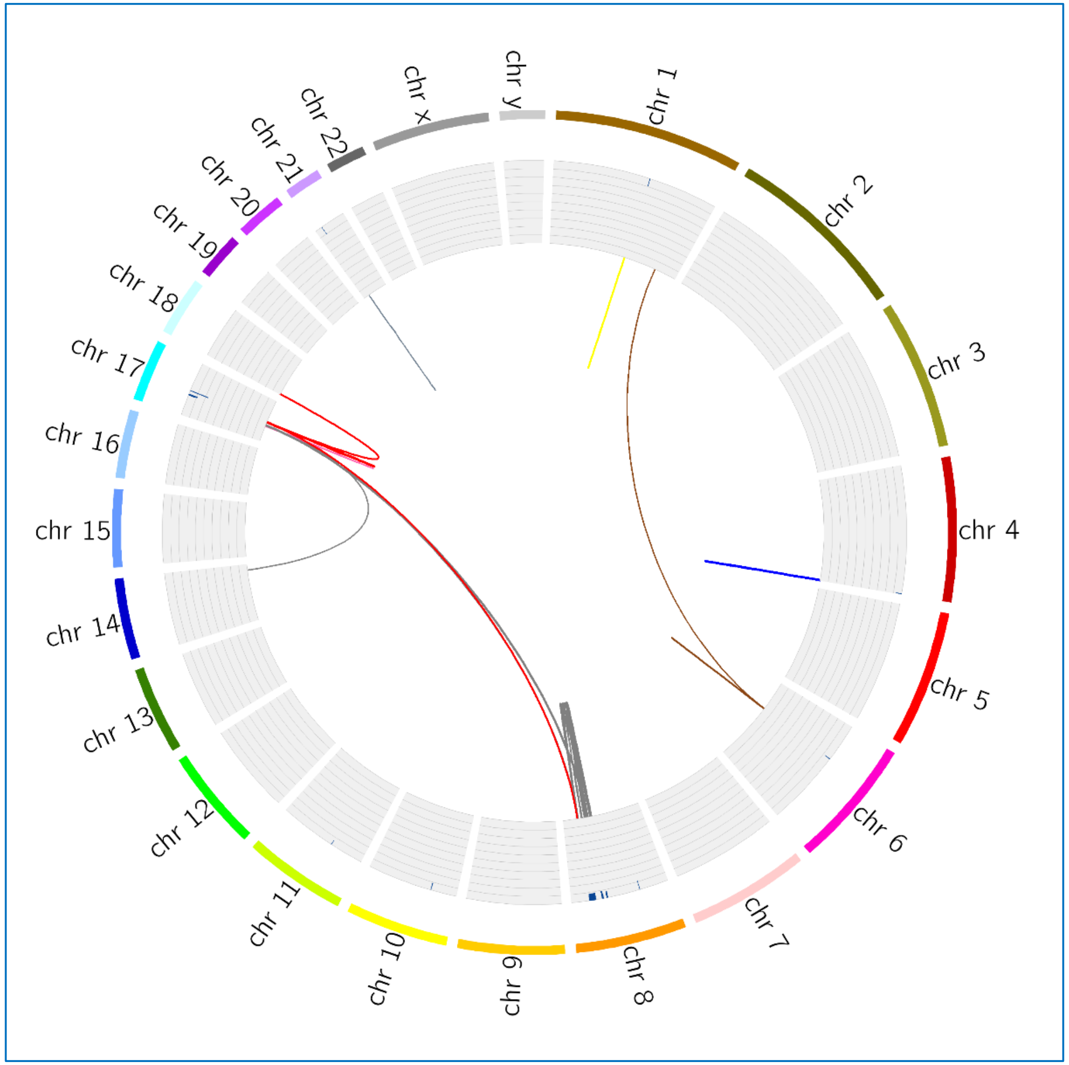

FAST – Whole Genome

p-216

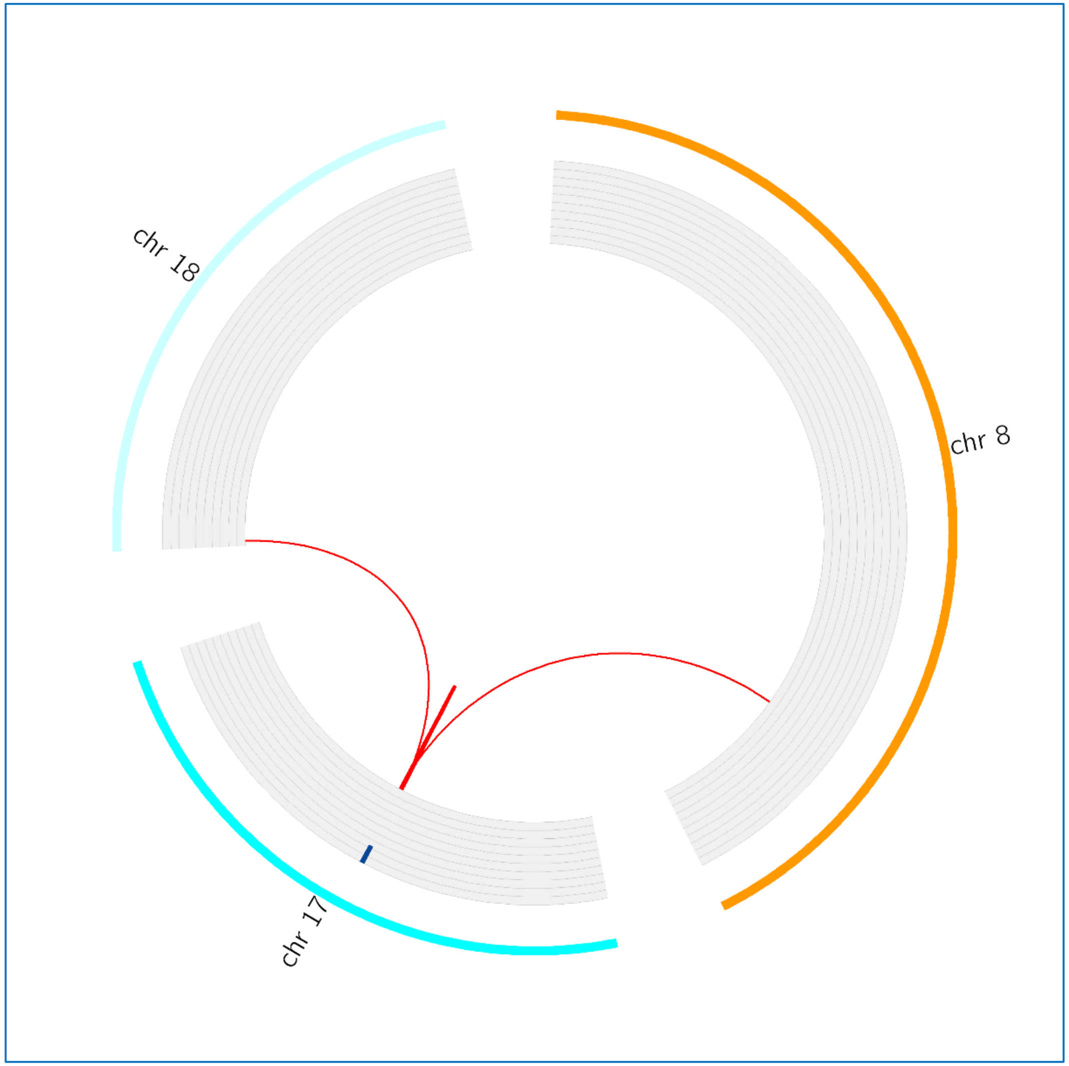

FAST – ERBB2 amplicon

p-217

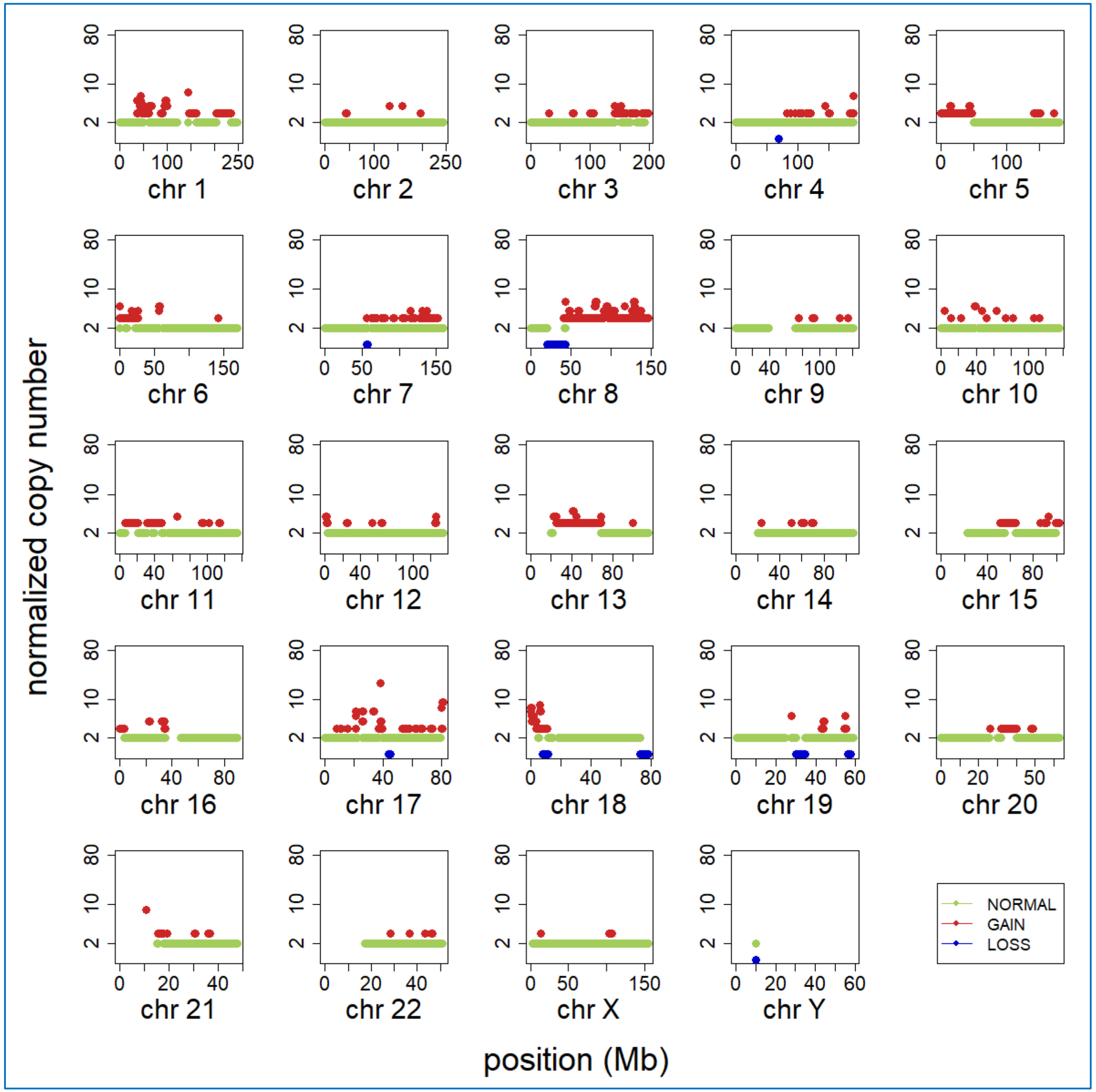

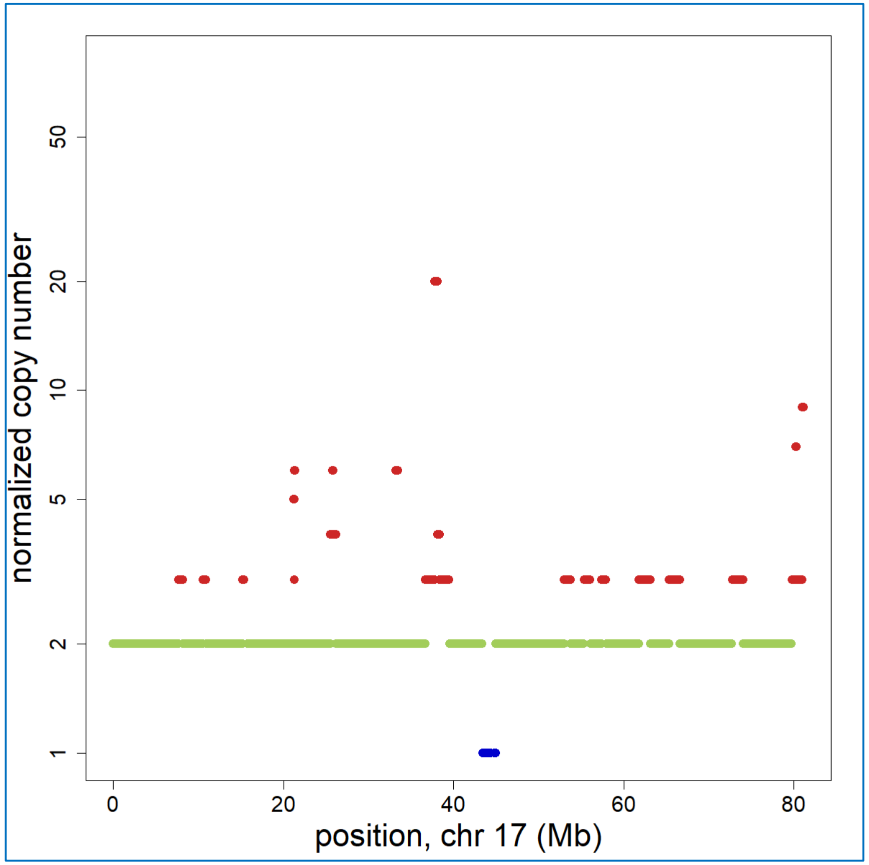

Control-FREEC

p-217

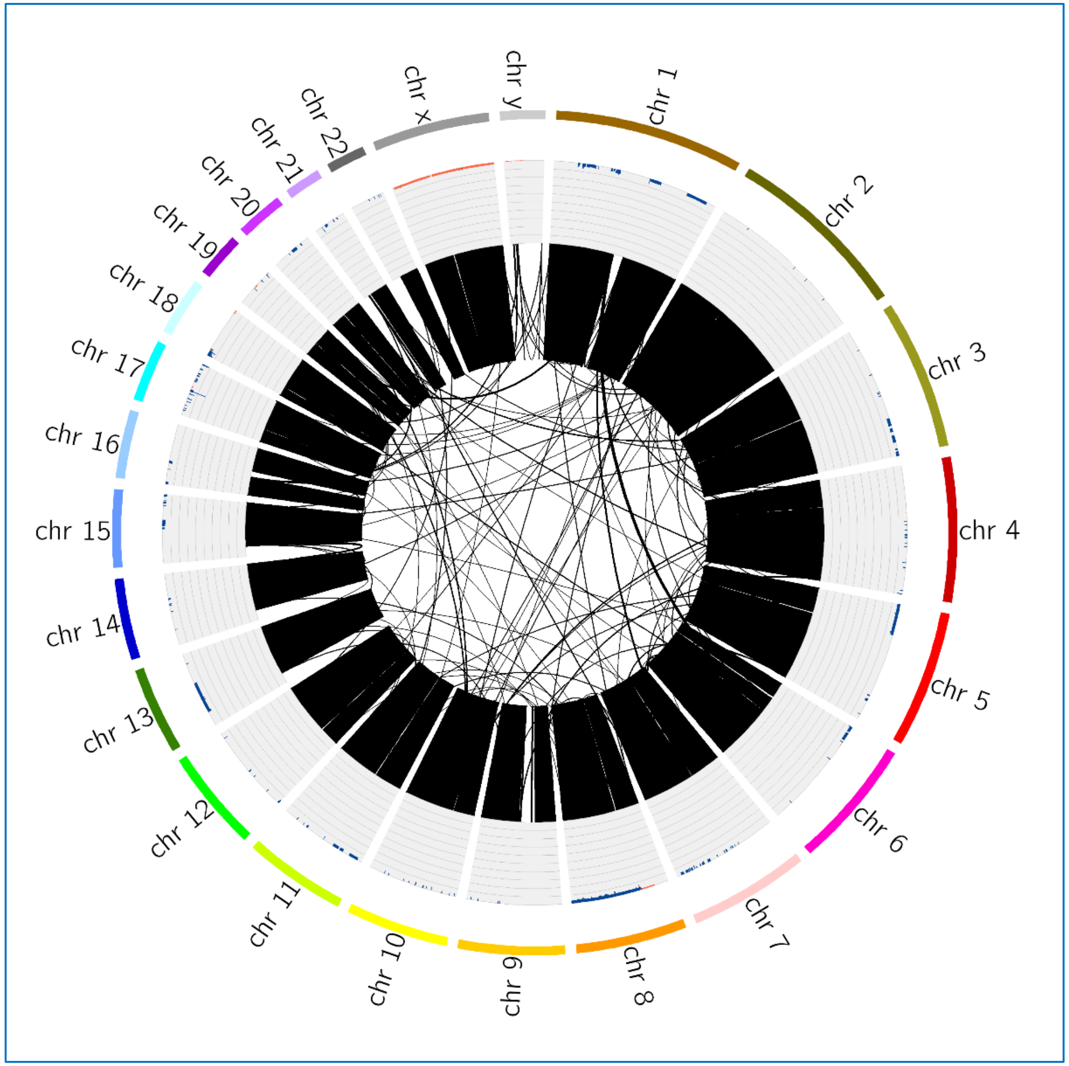

BreakDancer + Control-FREEC

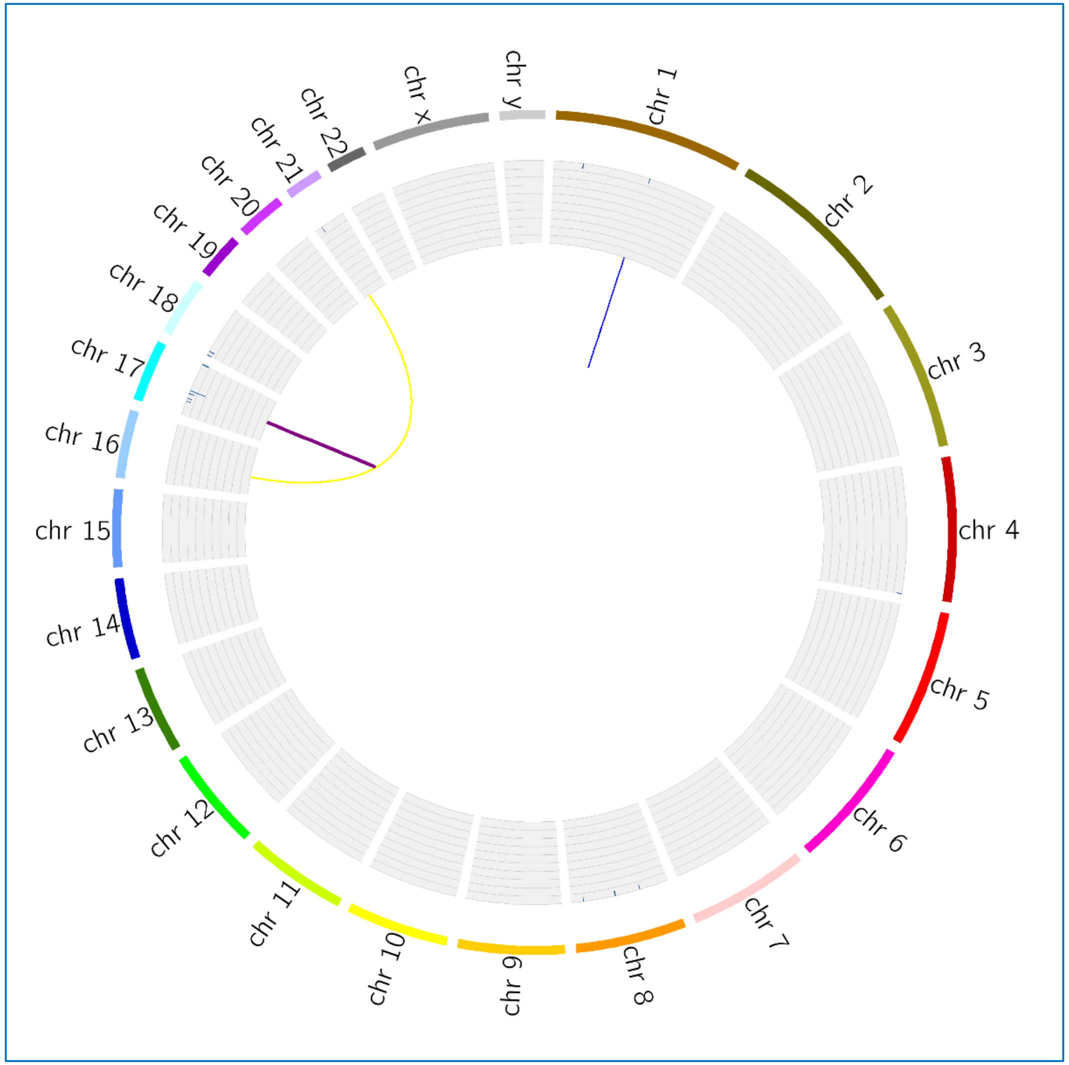

FAST – Whole Genome

p-217

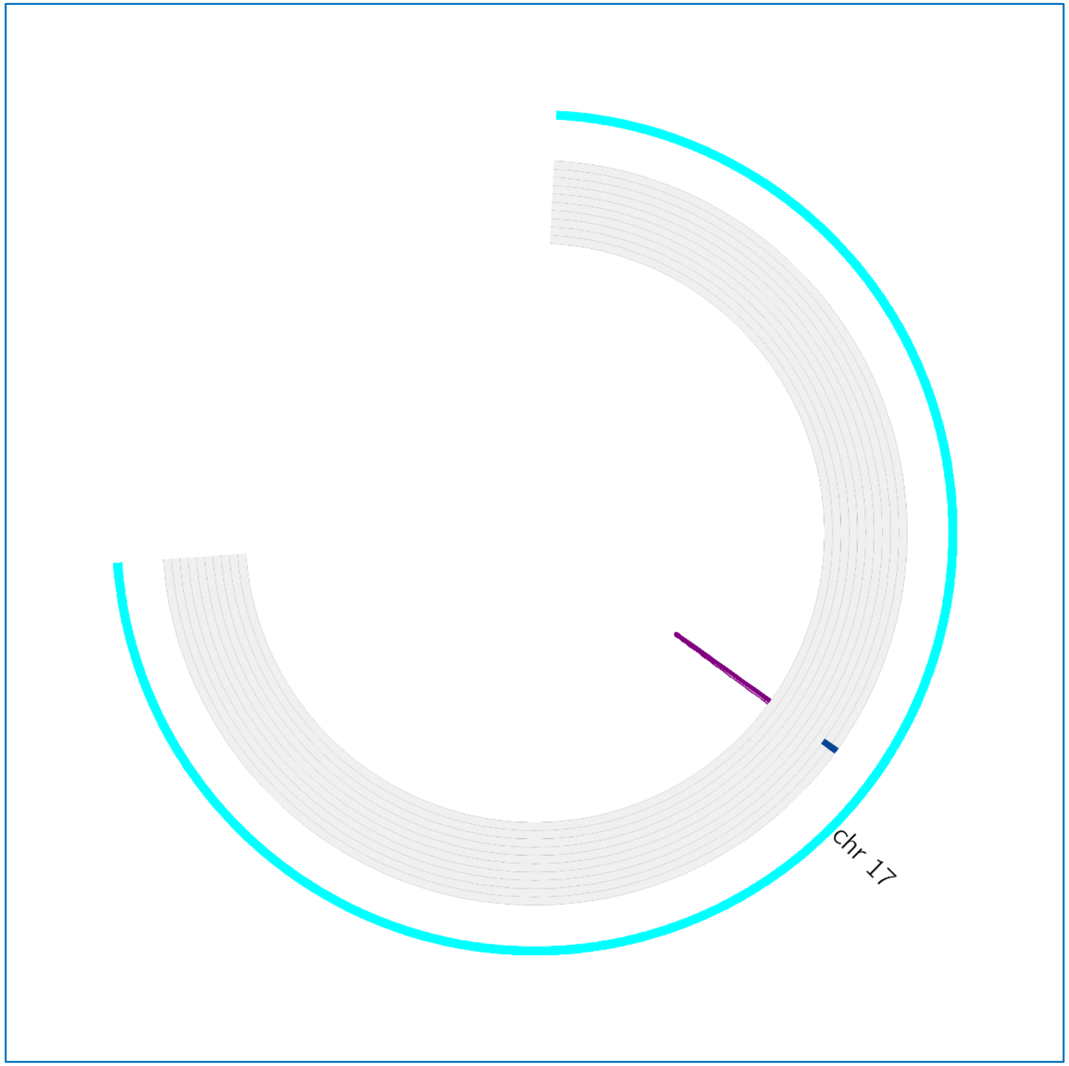

FAST – ERBB2 amplicon

p-218

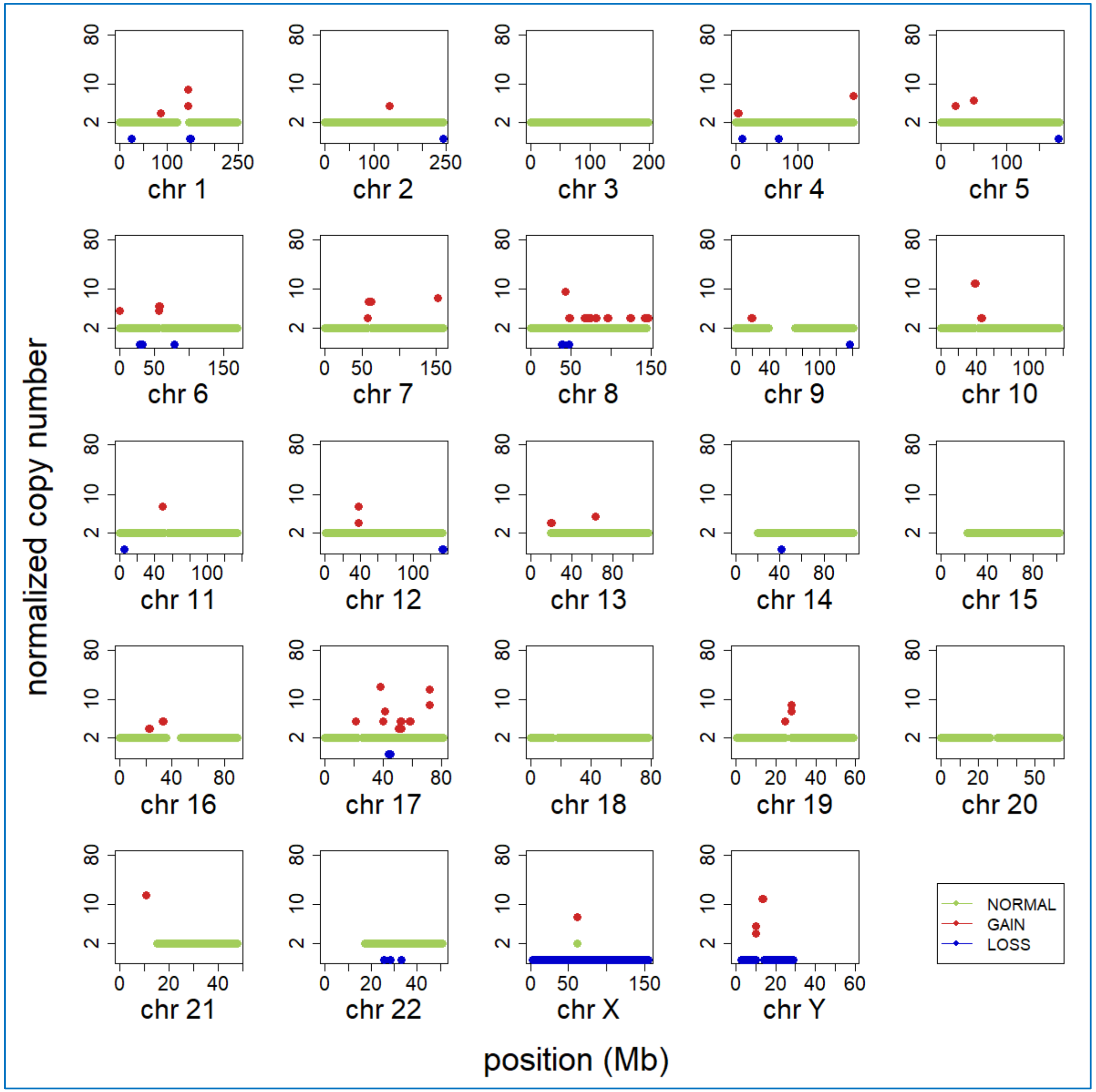

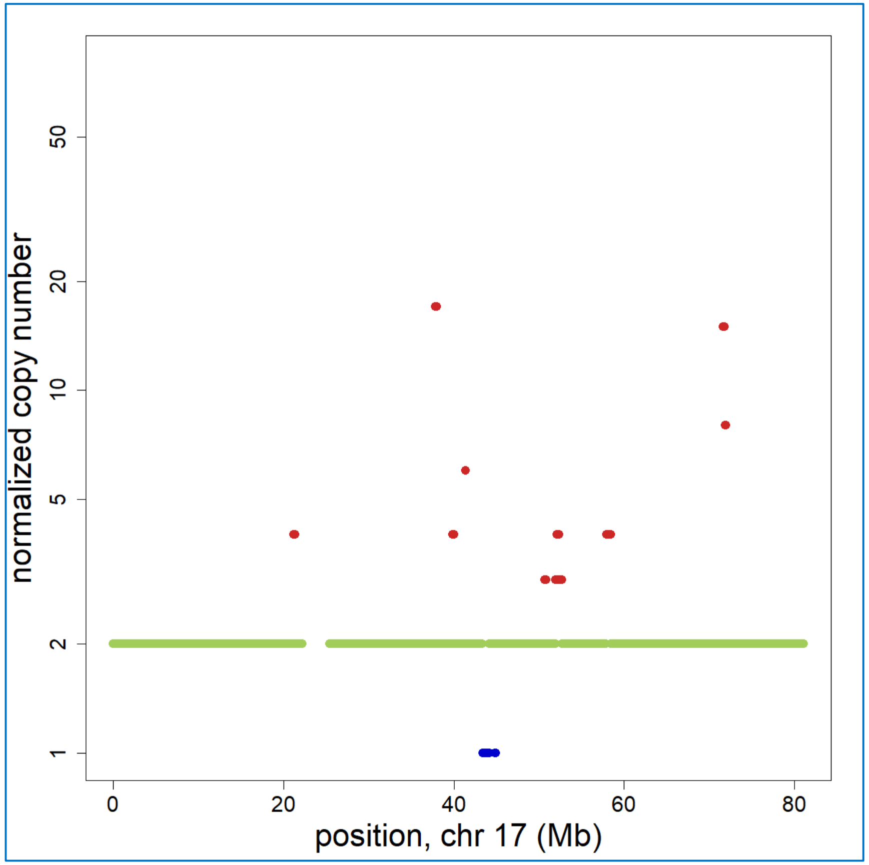

Control-FREEC

p-218

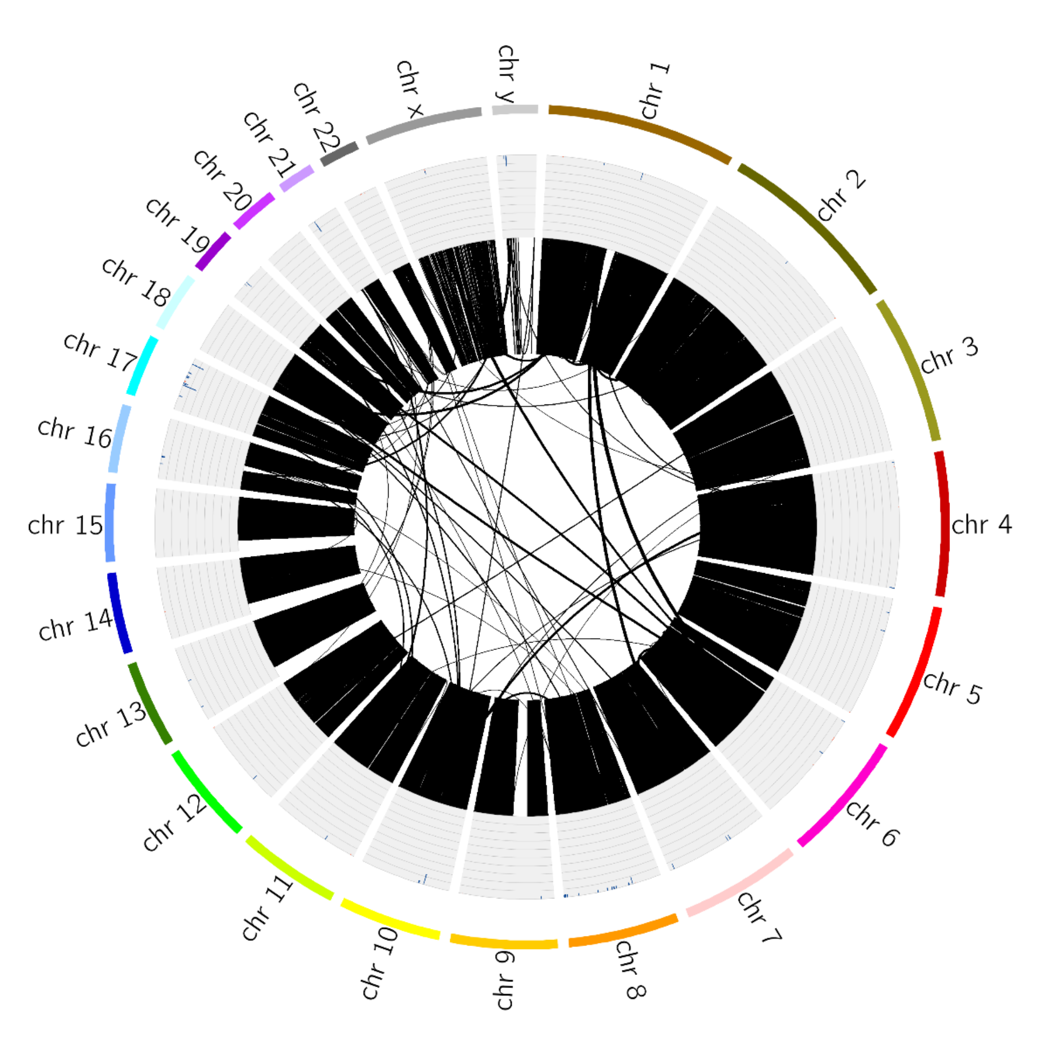

BreakDancer + Control-FREEC

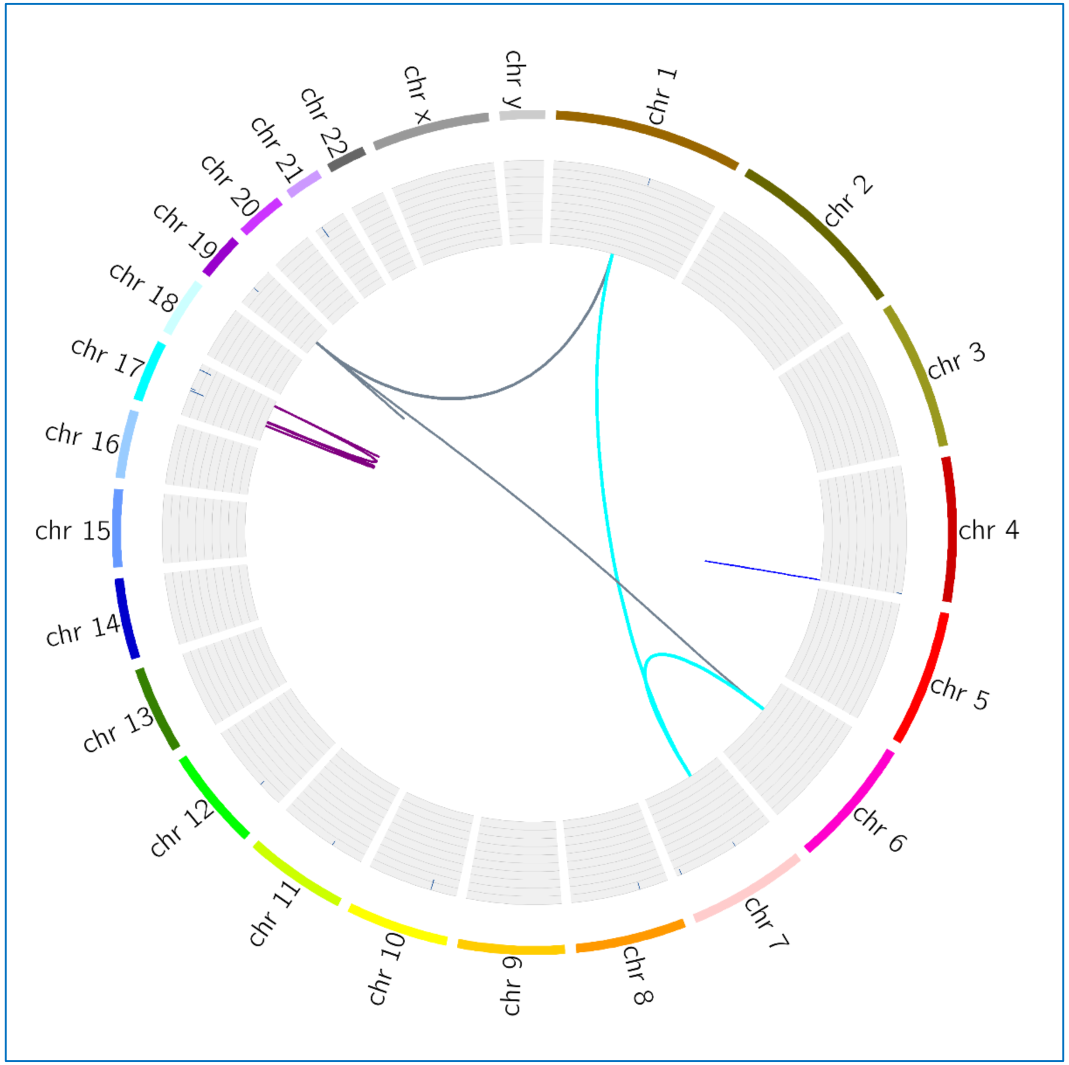

FAST – Whole Genome

p-218

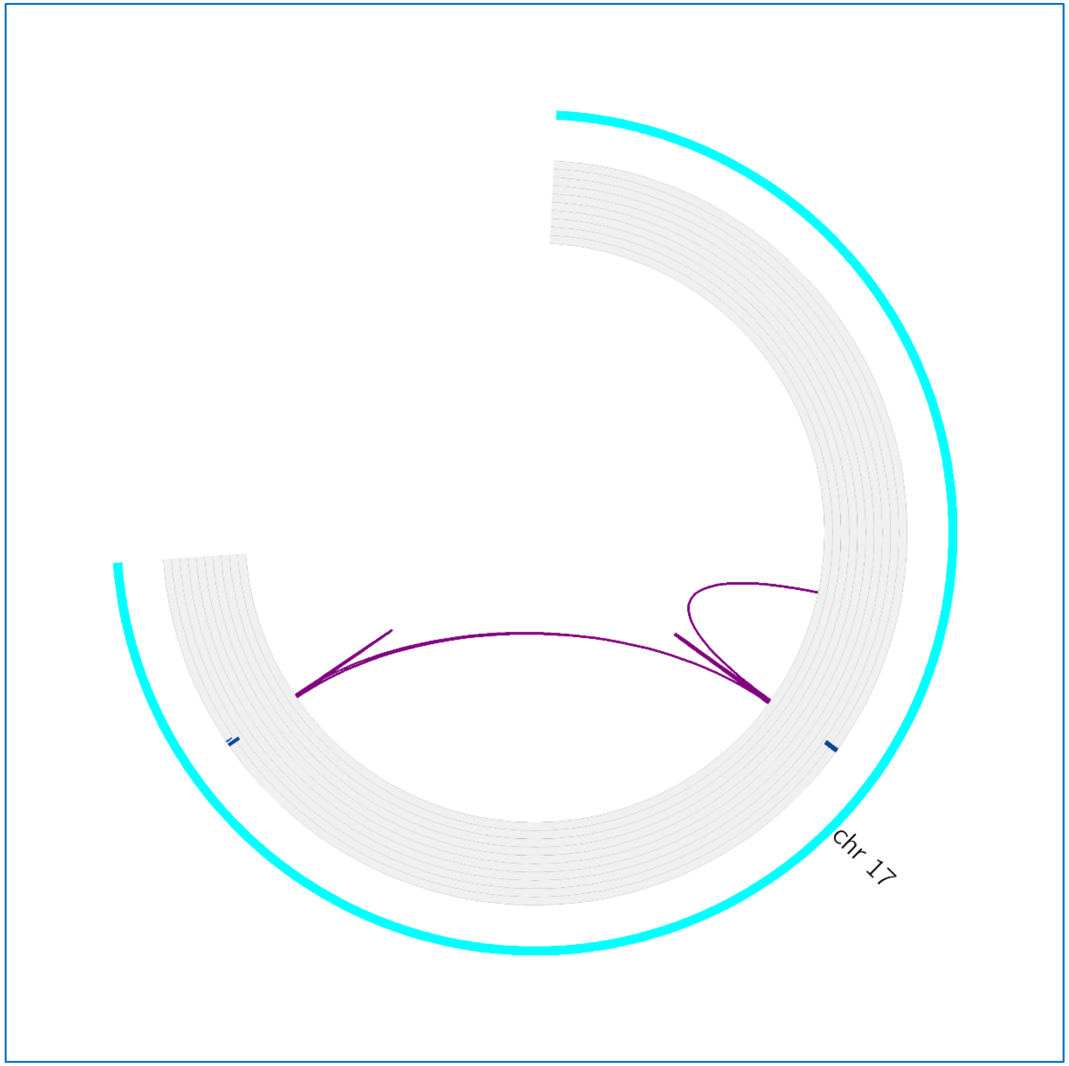

FAST – ERBB2 amplicon

p-6890

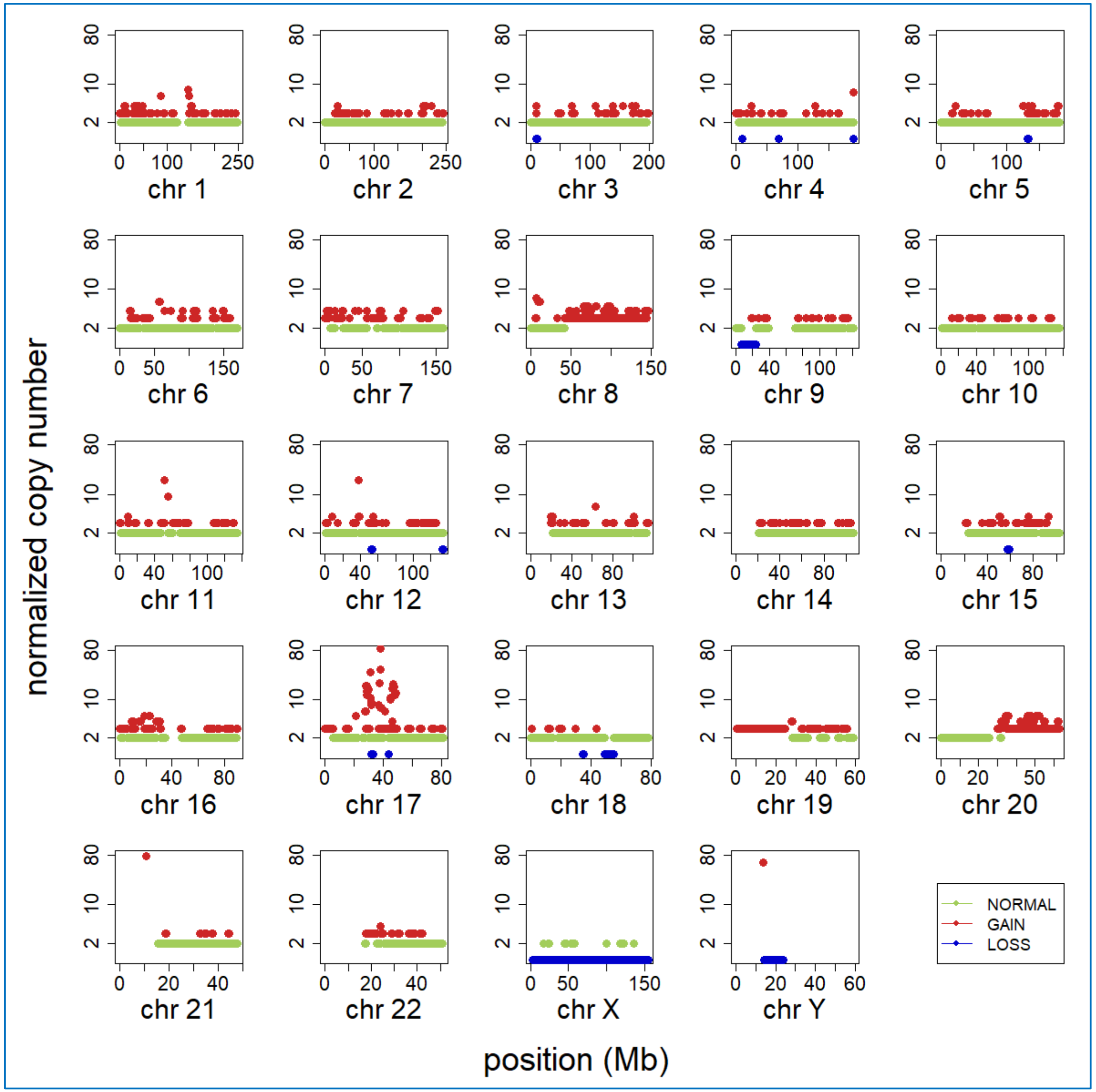

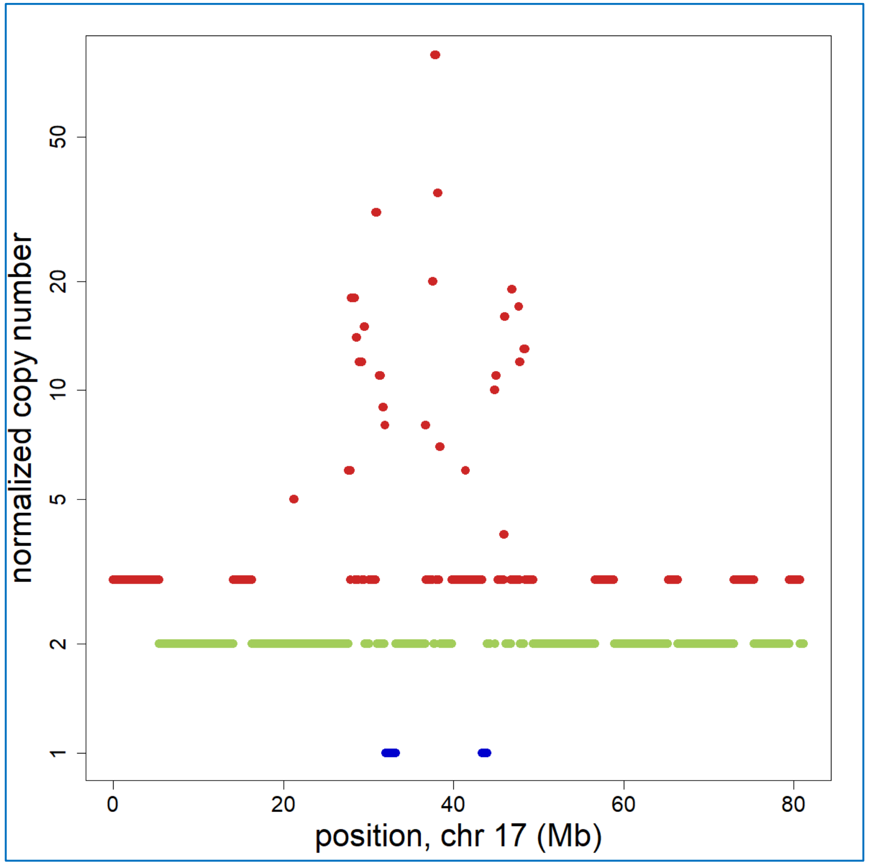

Control-FREEC

p-6890

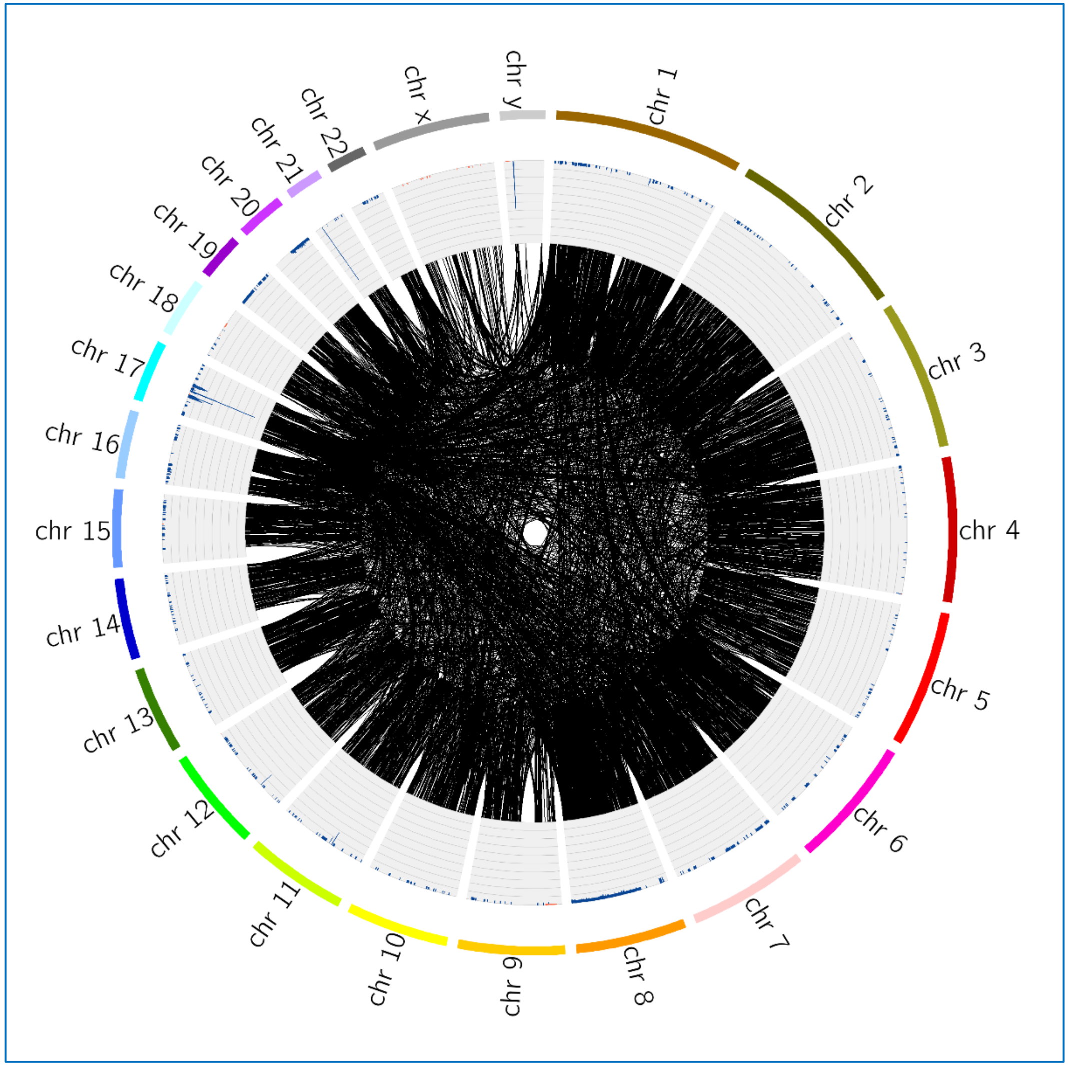

BreakDancer + Control-FREEC

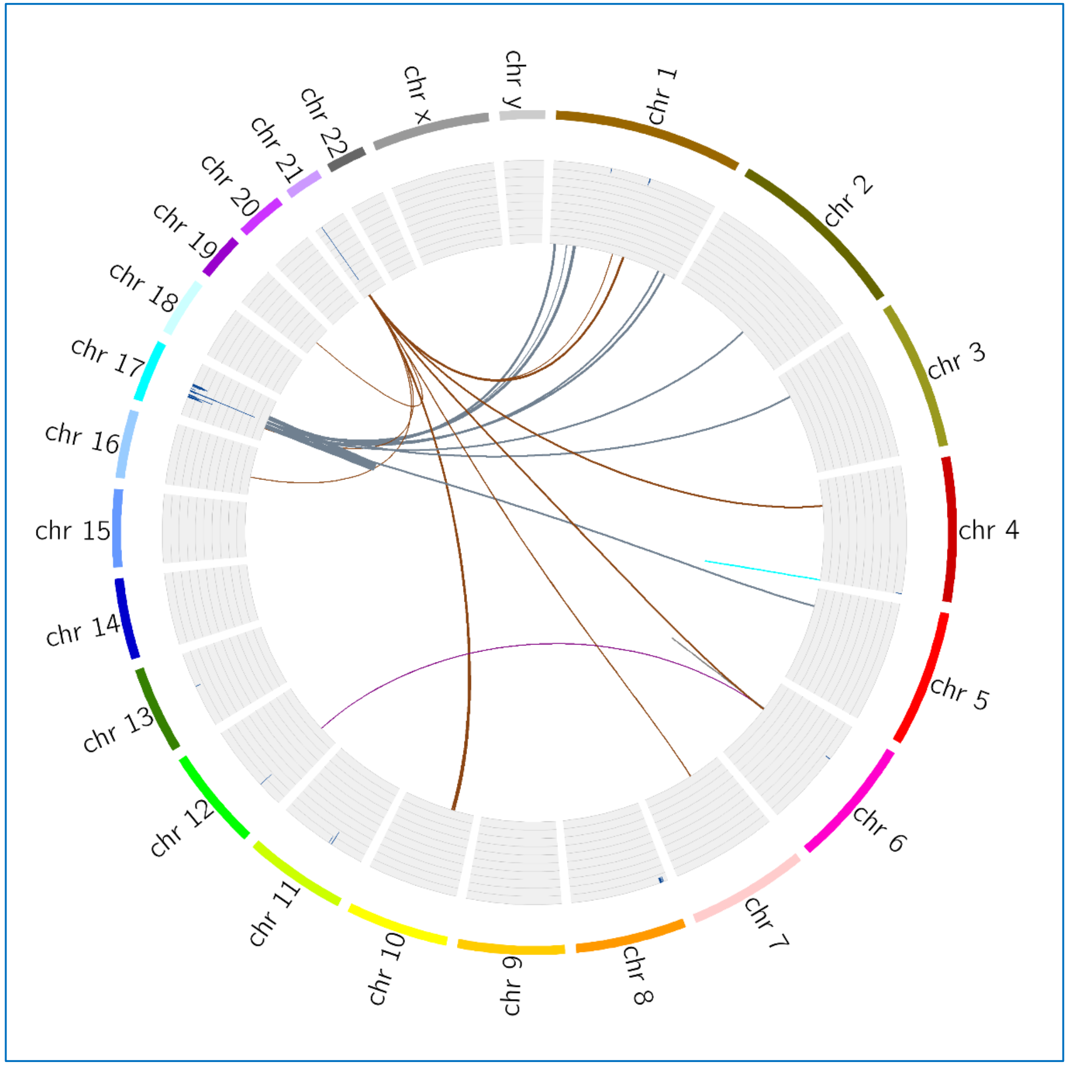

FAST – Whole Genome

p-6890

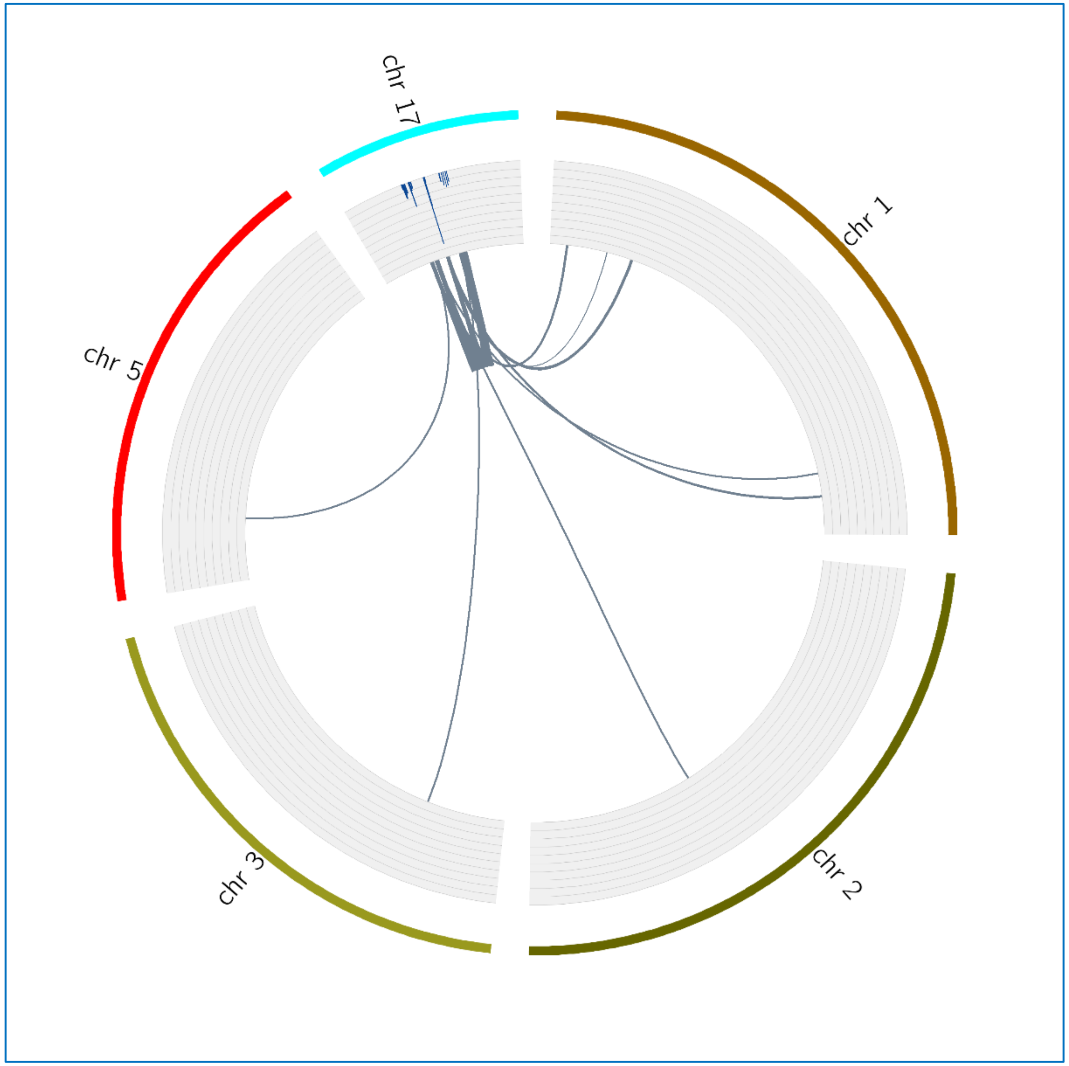

FAST – ERBB2 amplicon

p-8191

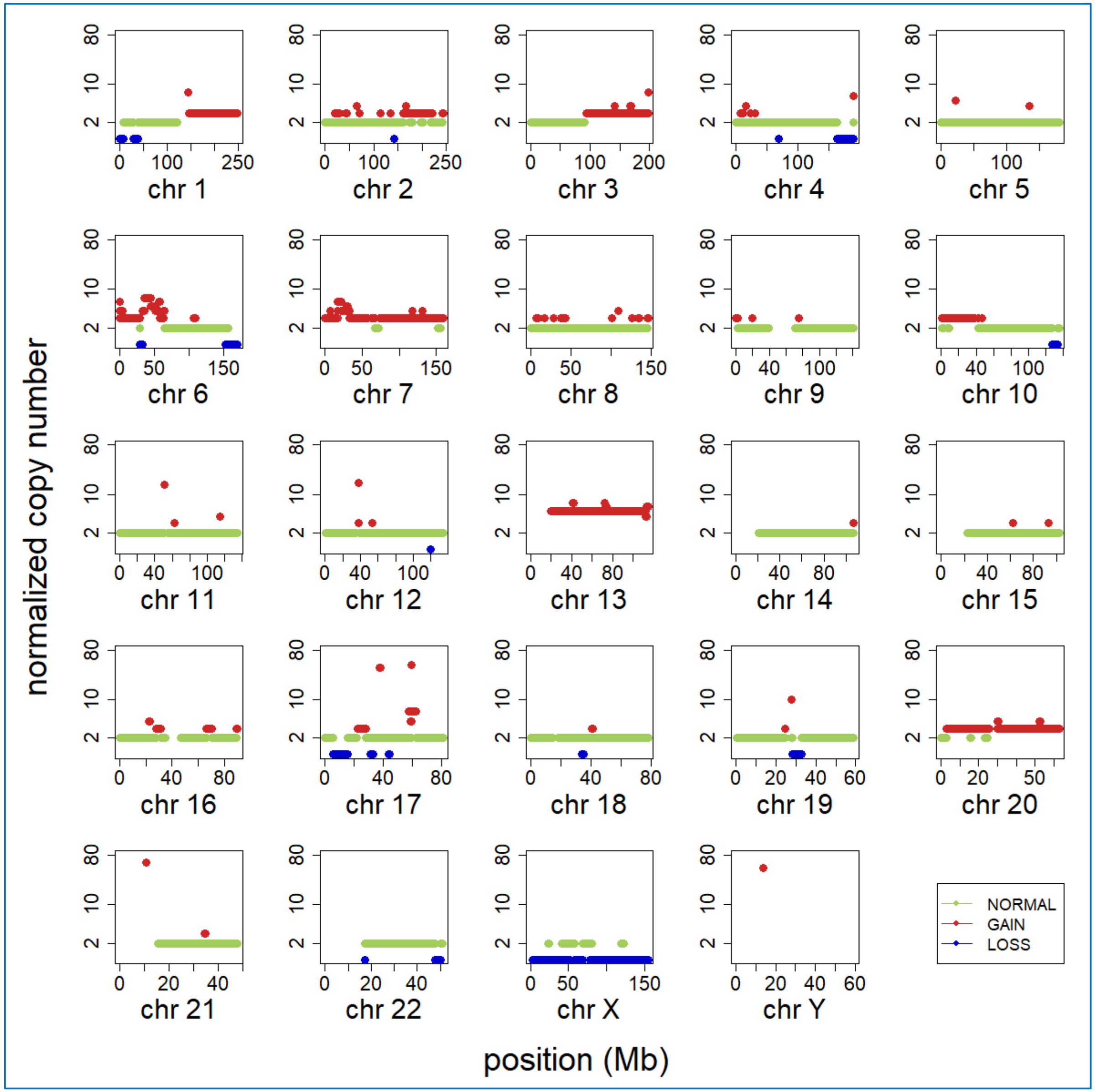

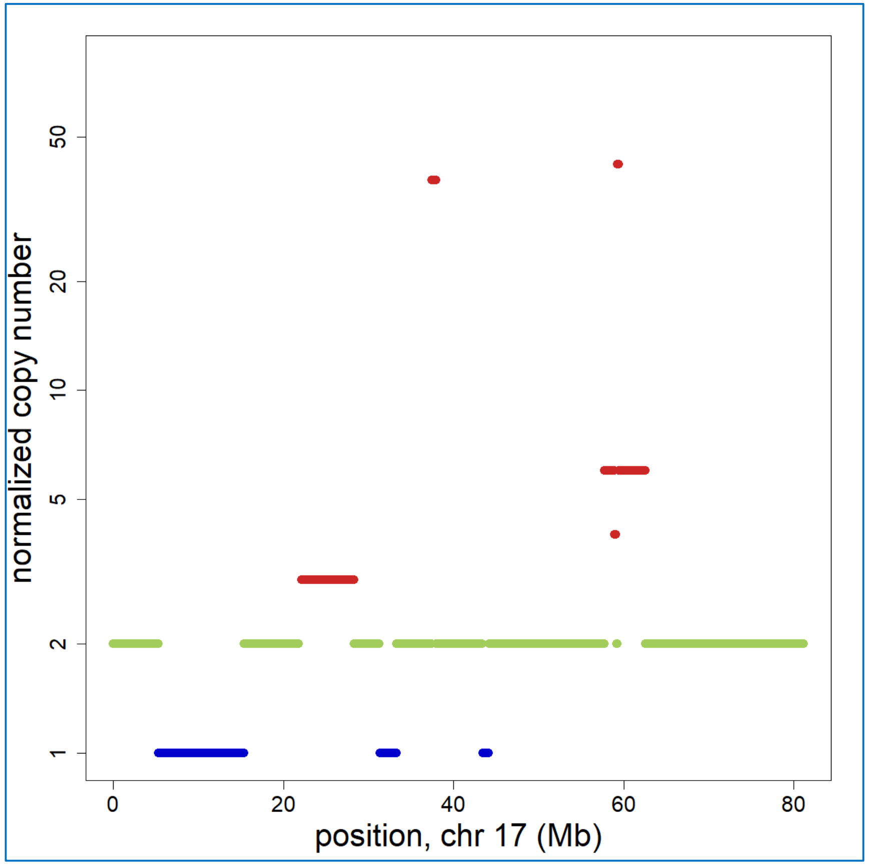

Control-FREEC

p-8191

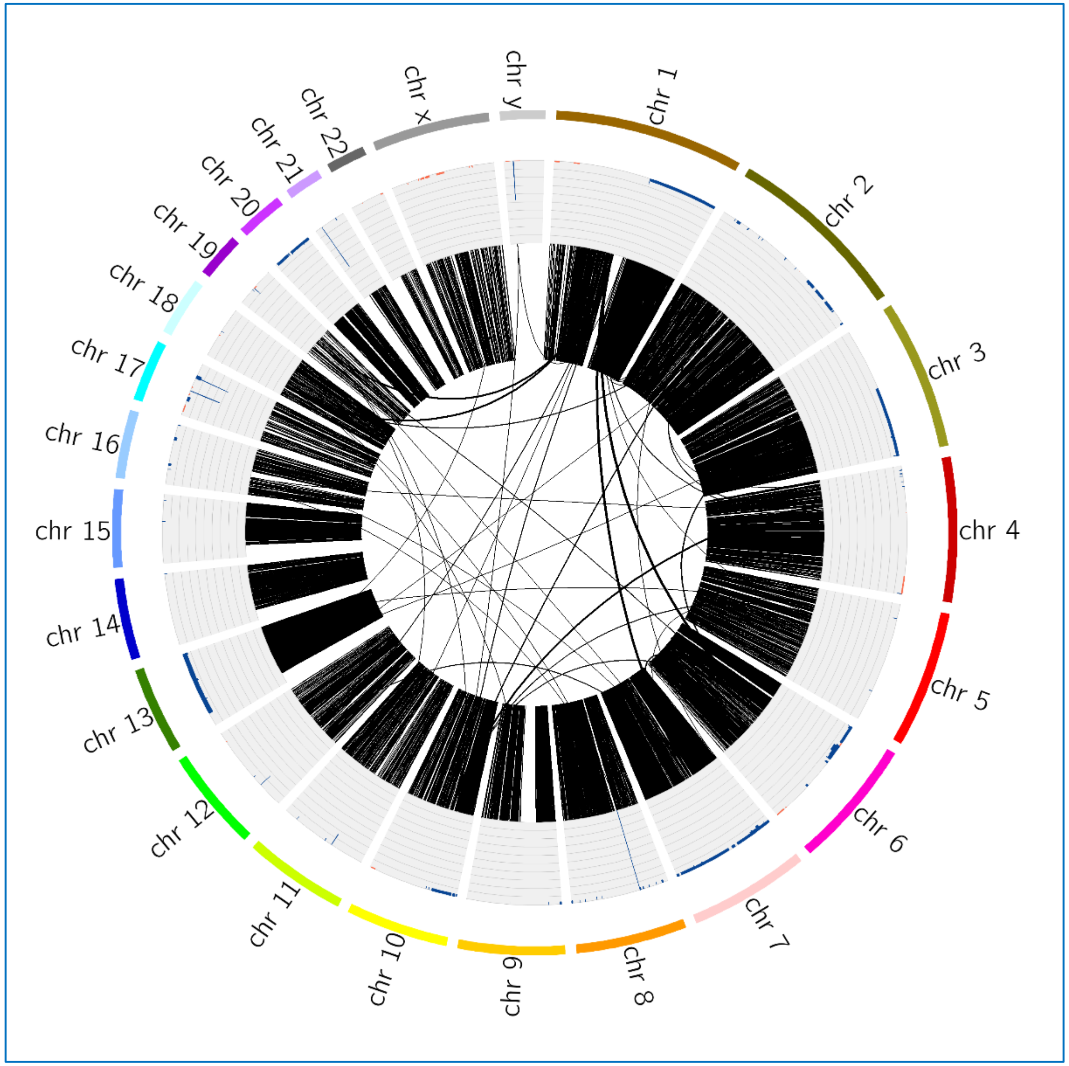

BreakDancer + Control-FREEC

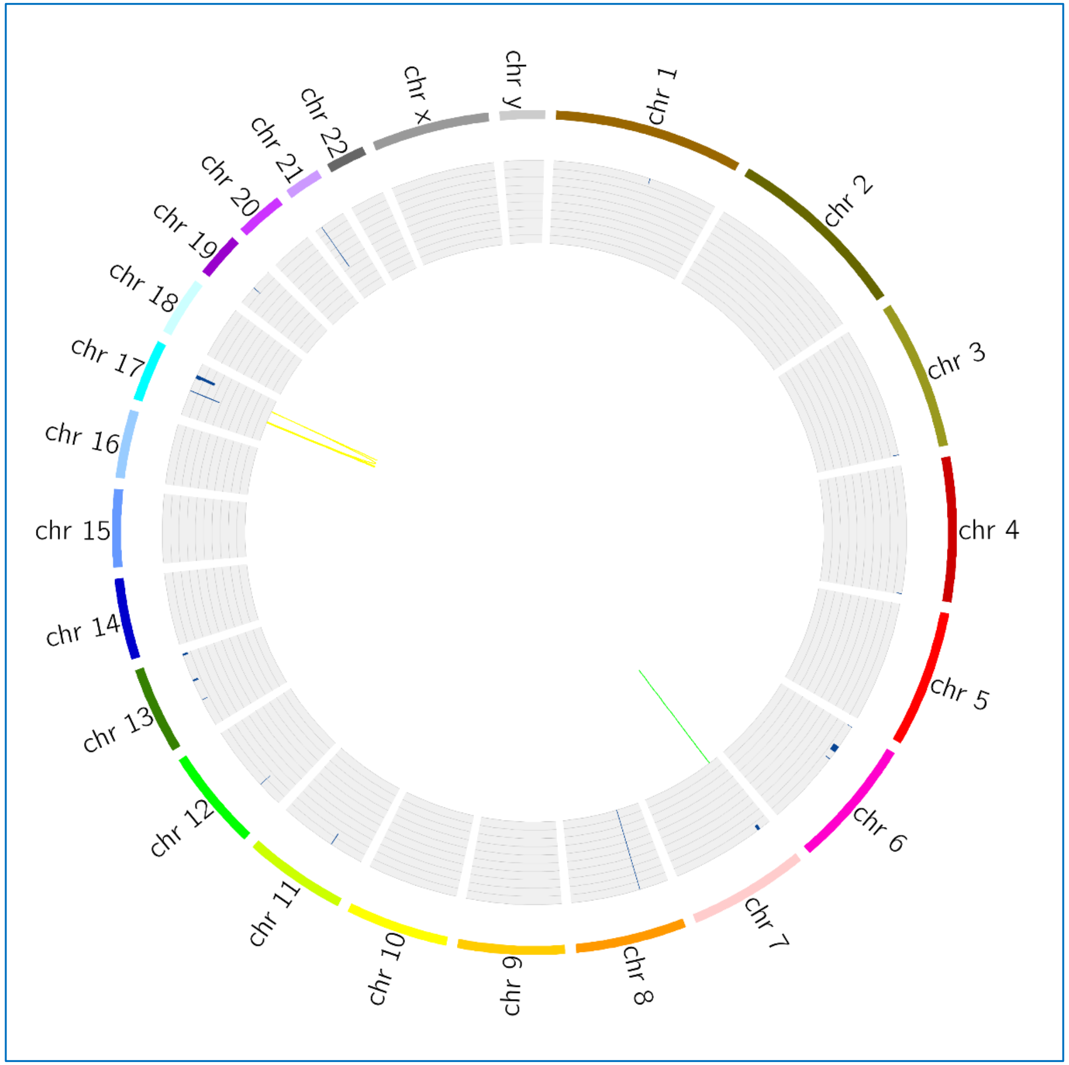

FAST – Whole Genome

p-8191

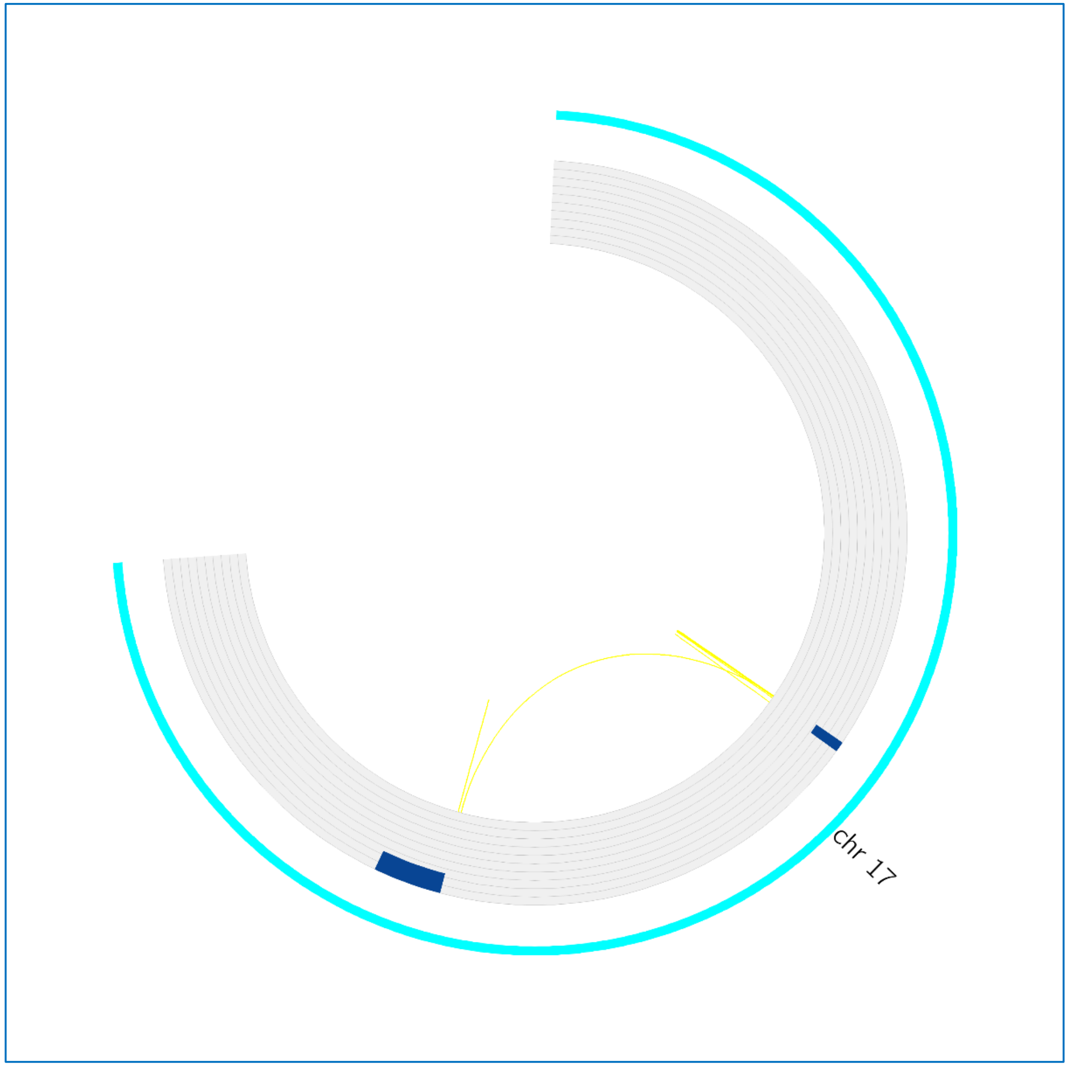

FAST – ERBB2 amplicon

20983

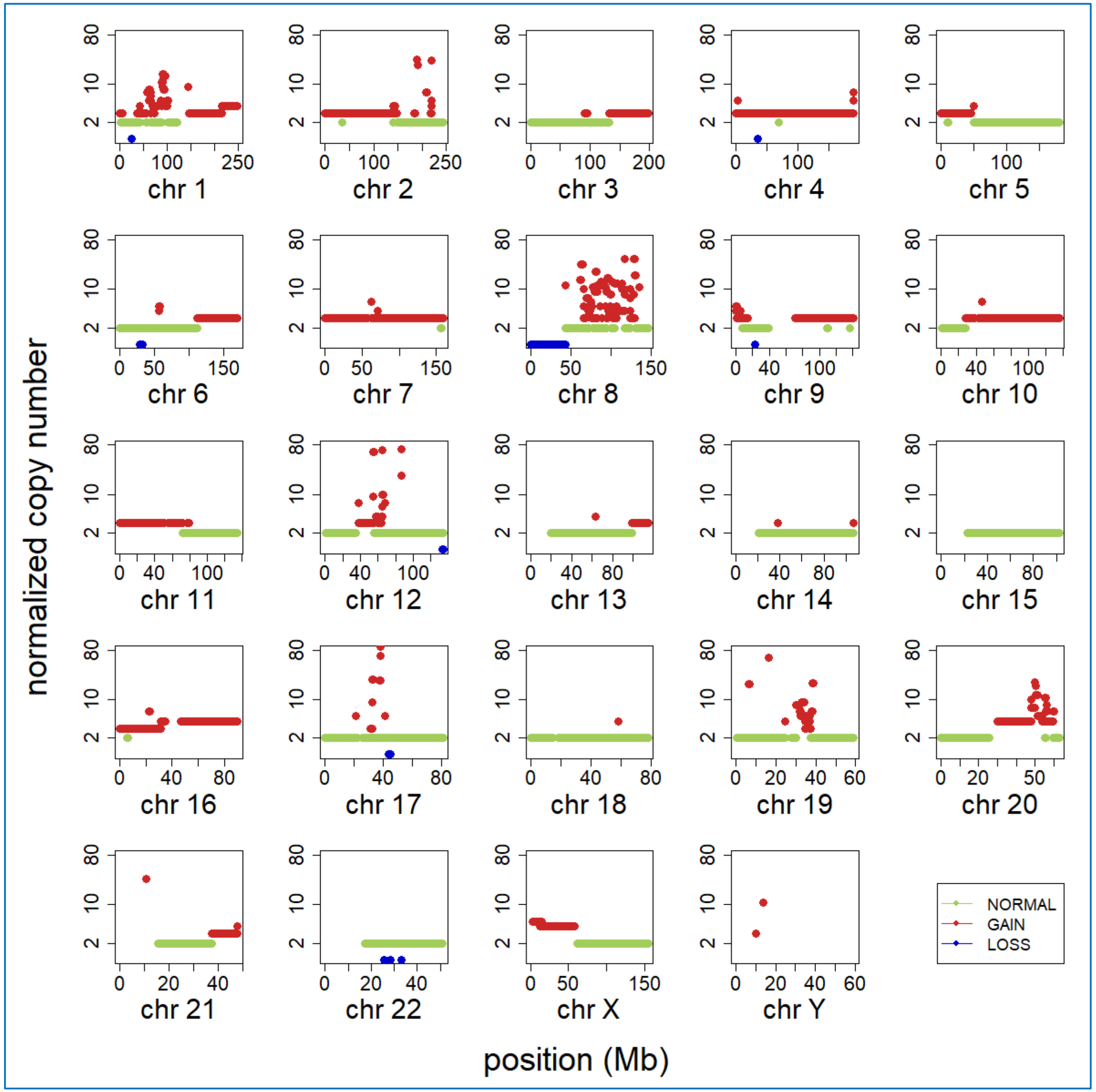

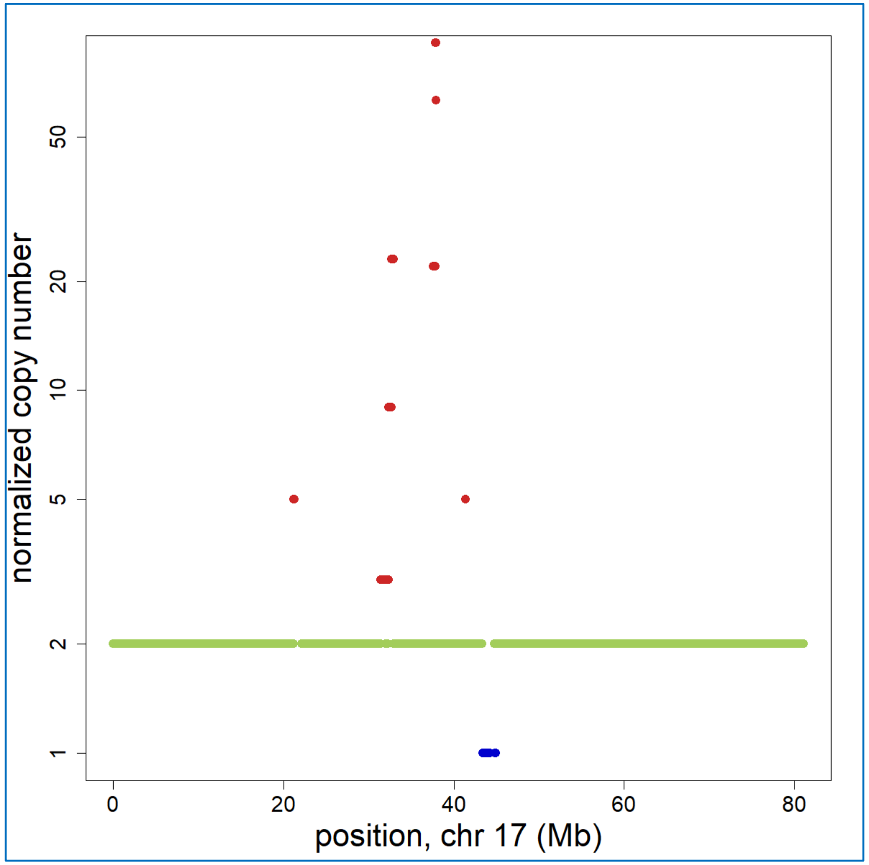

Control-FREEC

20983

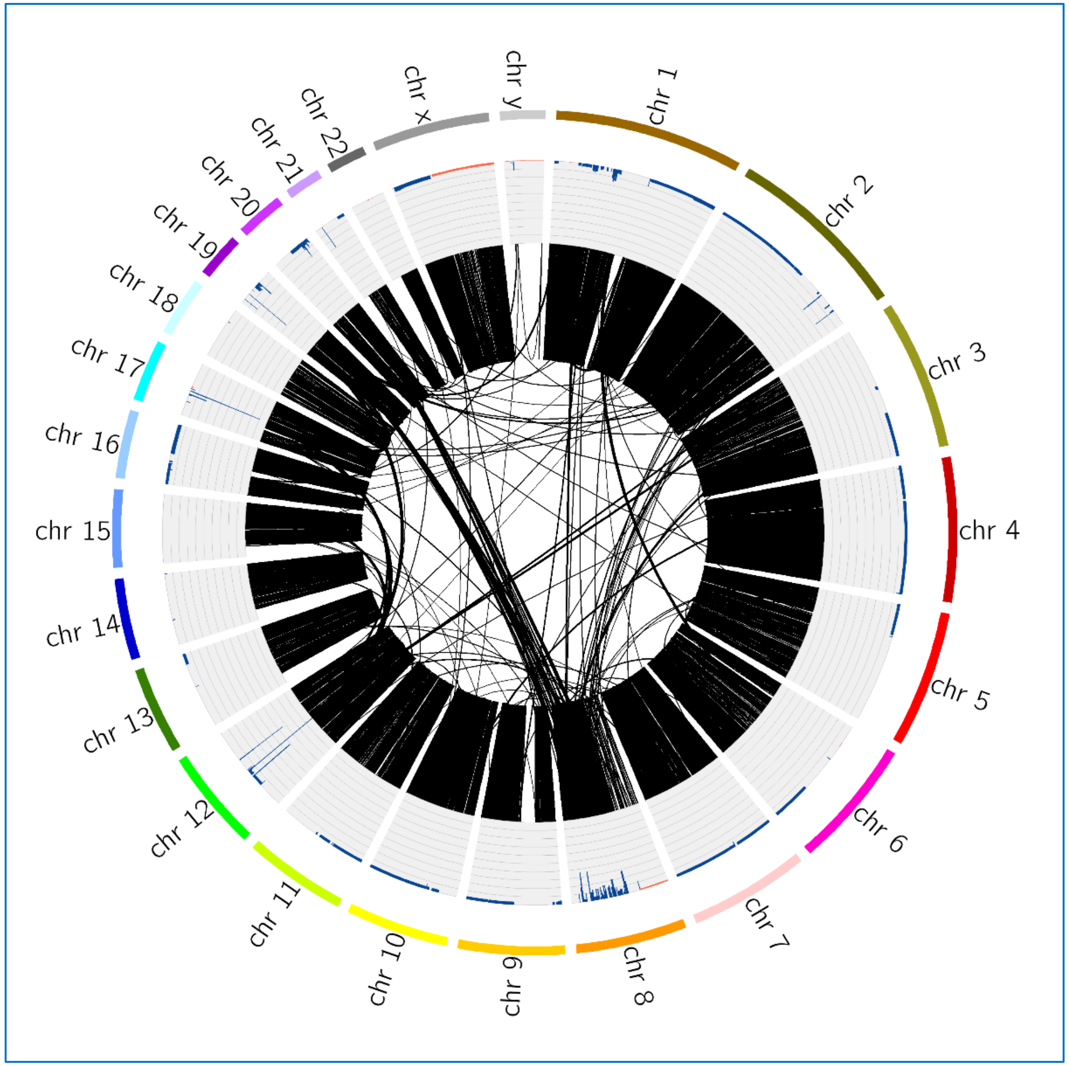

BreakDancer + Control-FREEC

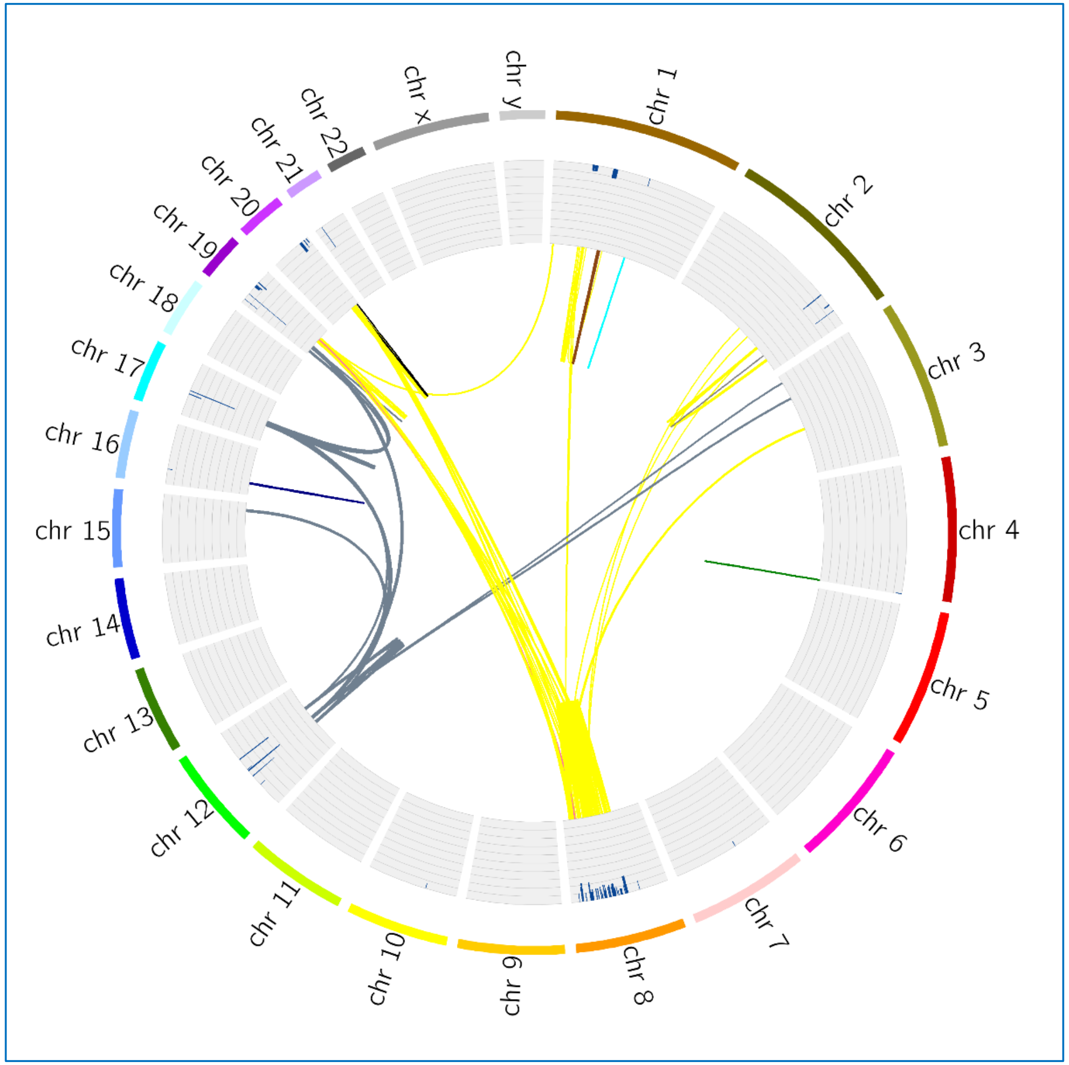

FAST – Whole Genome

20983

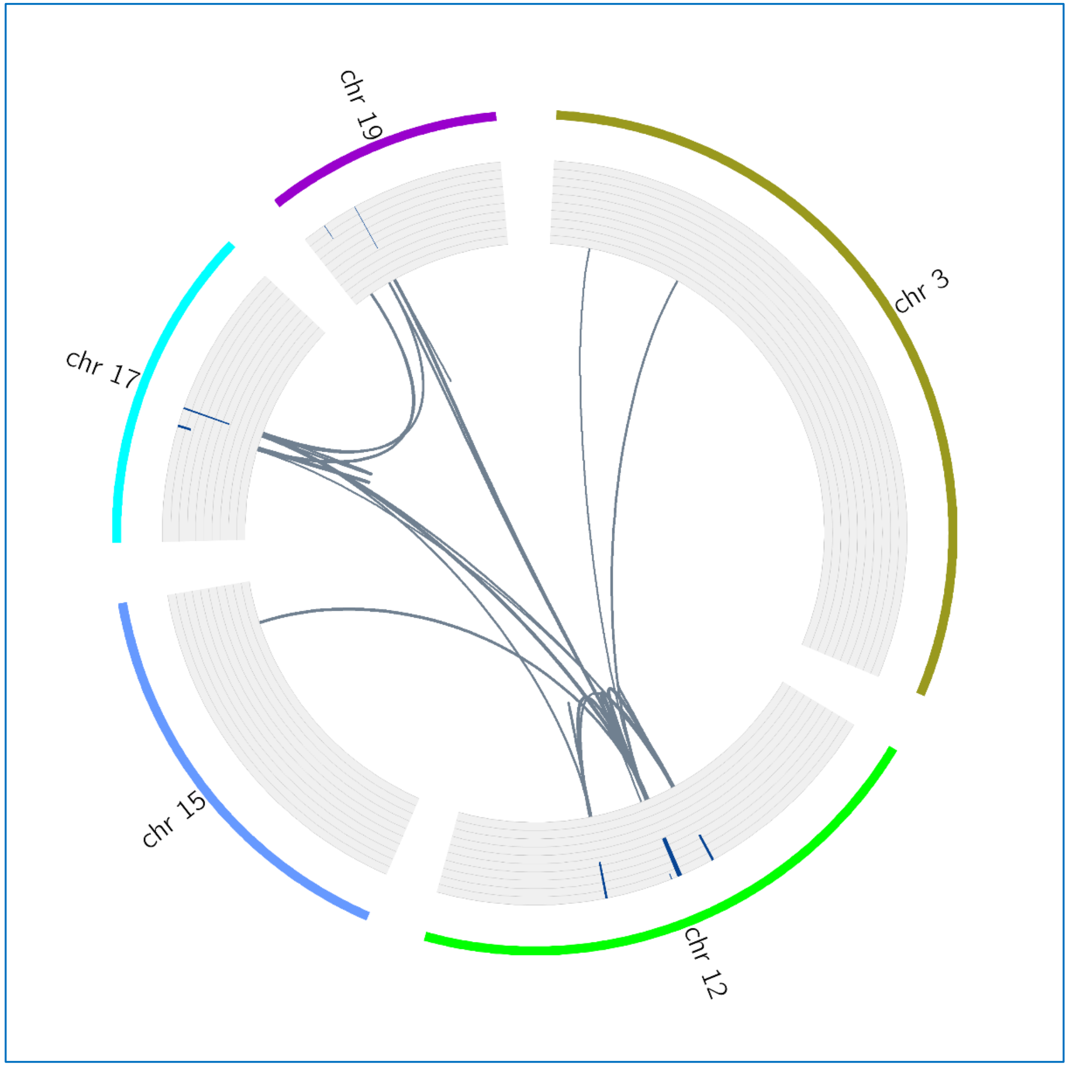

FAST – ERBB2 amplicon

80990

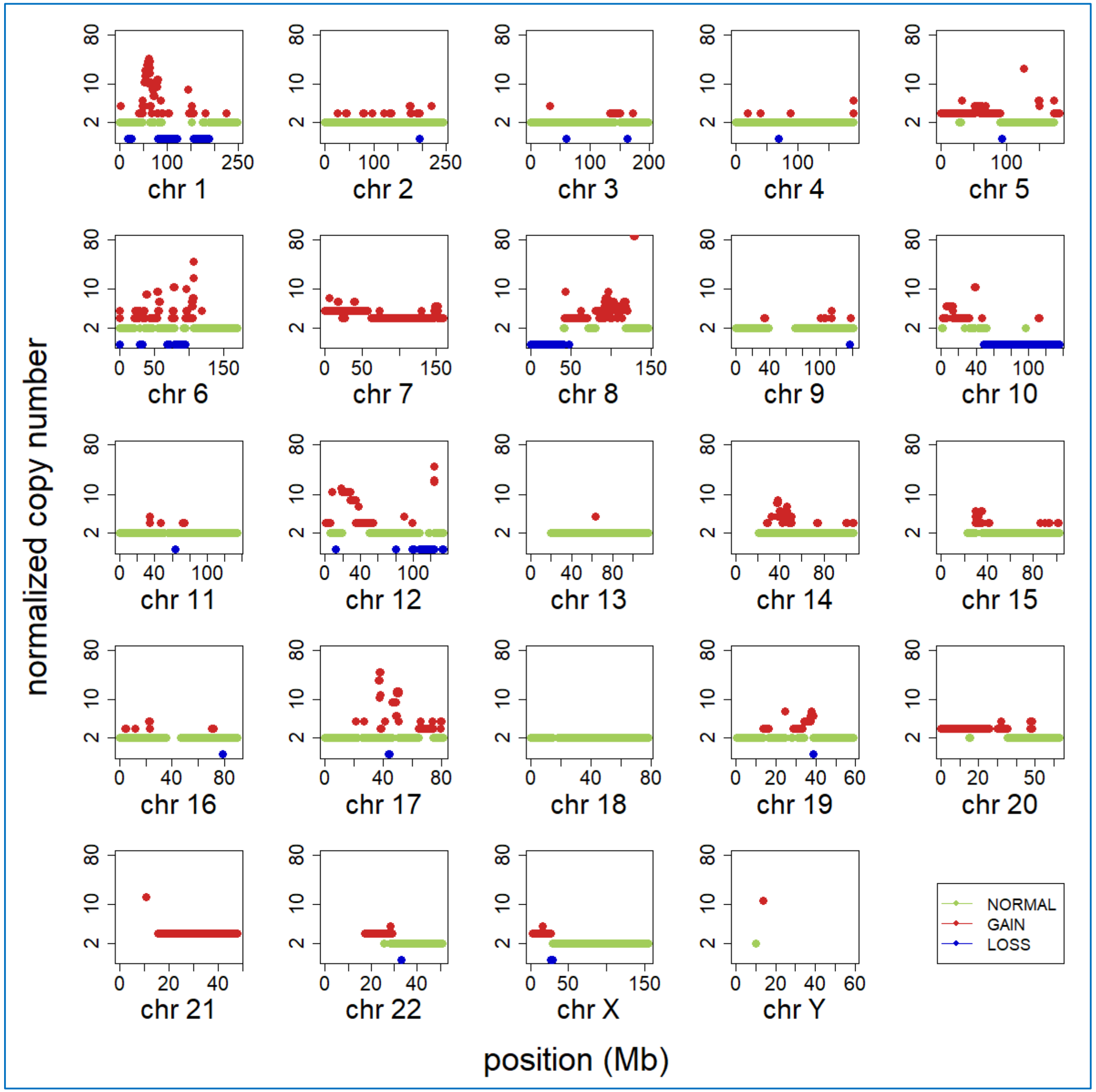

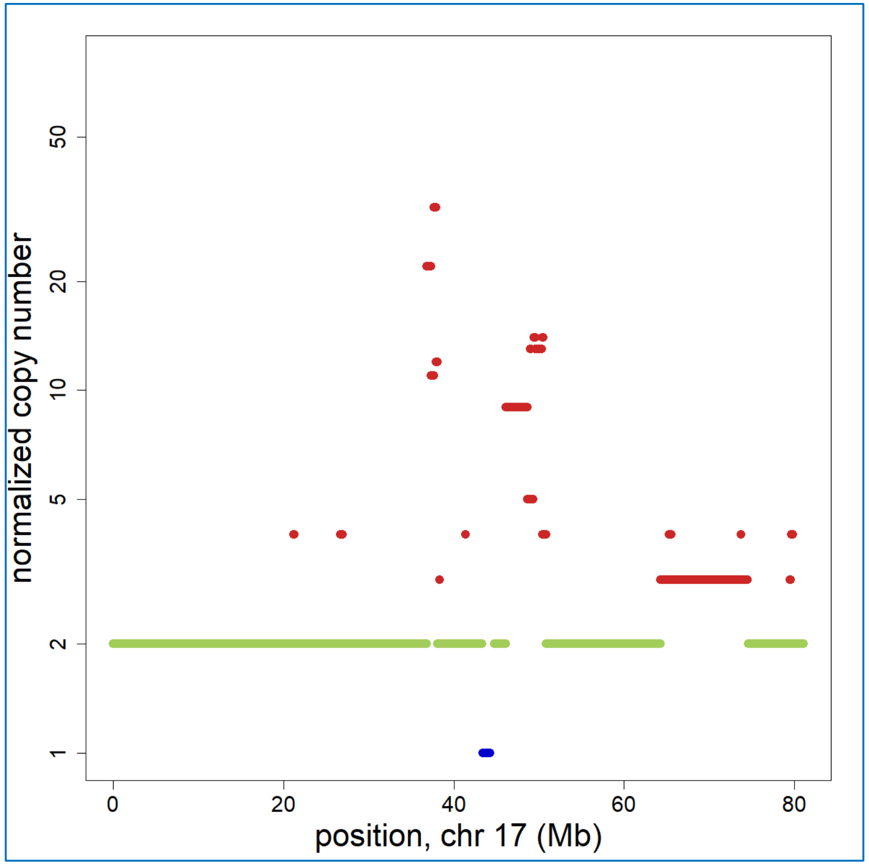

Control-FREEC

80990

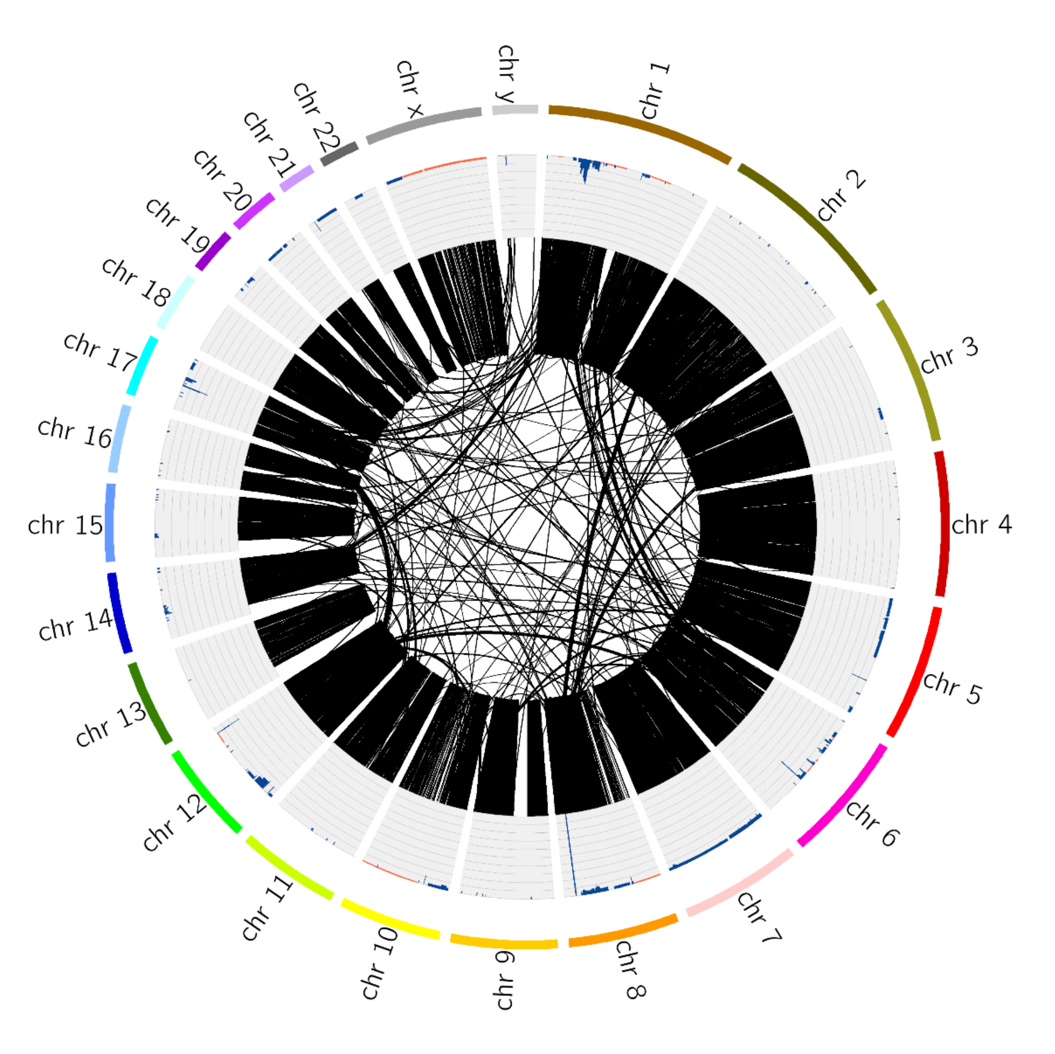

BreakDancer + Control-FREEC

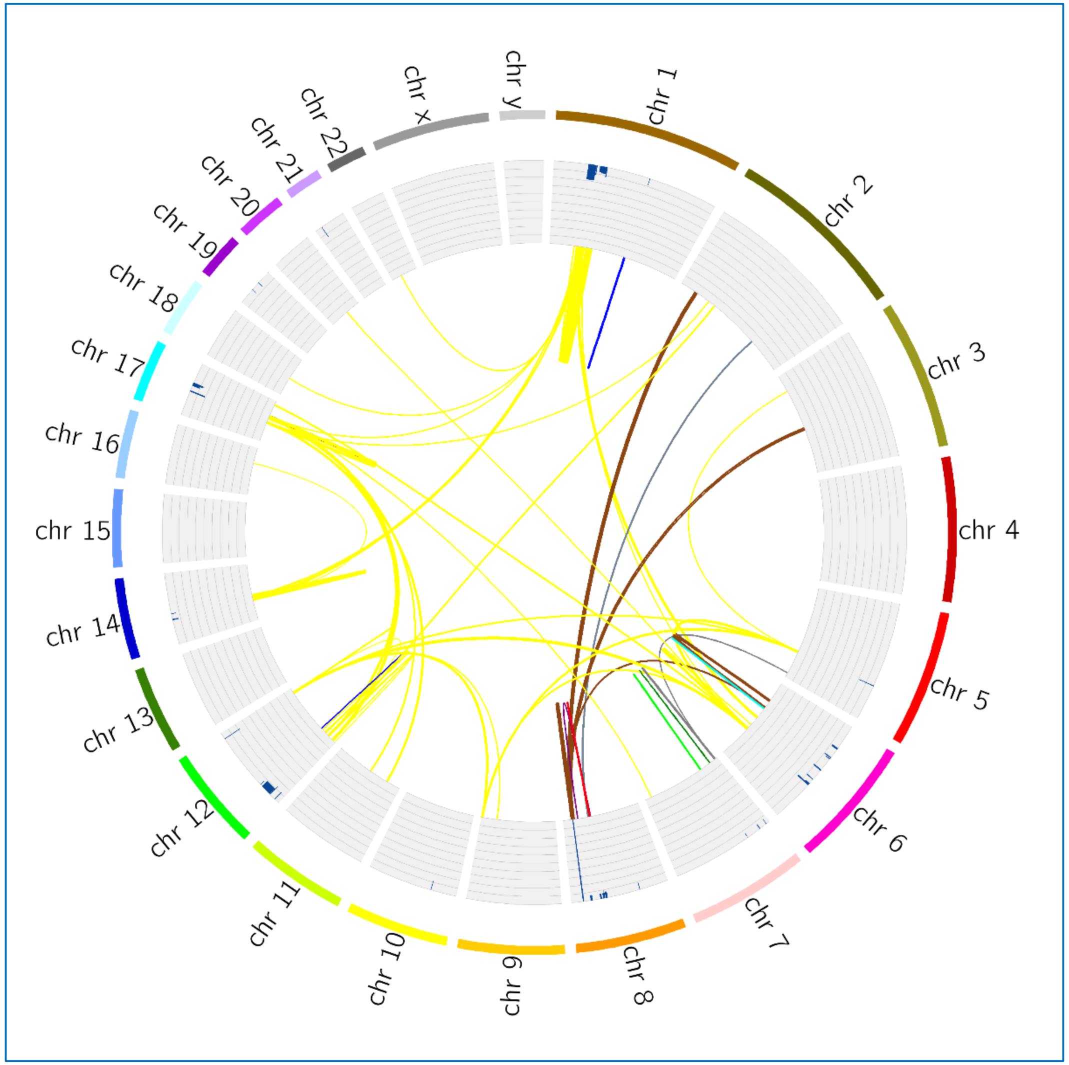

FAST – Whole Genome

80990

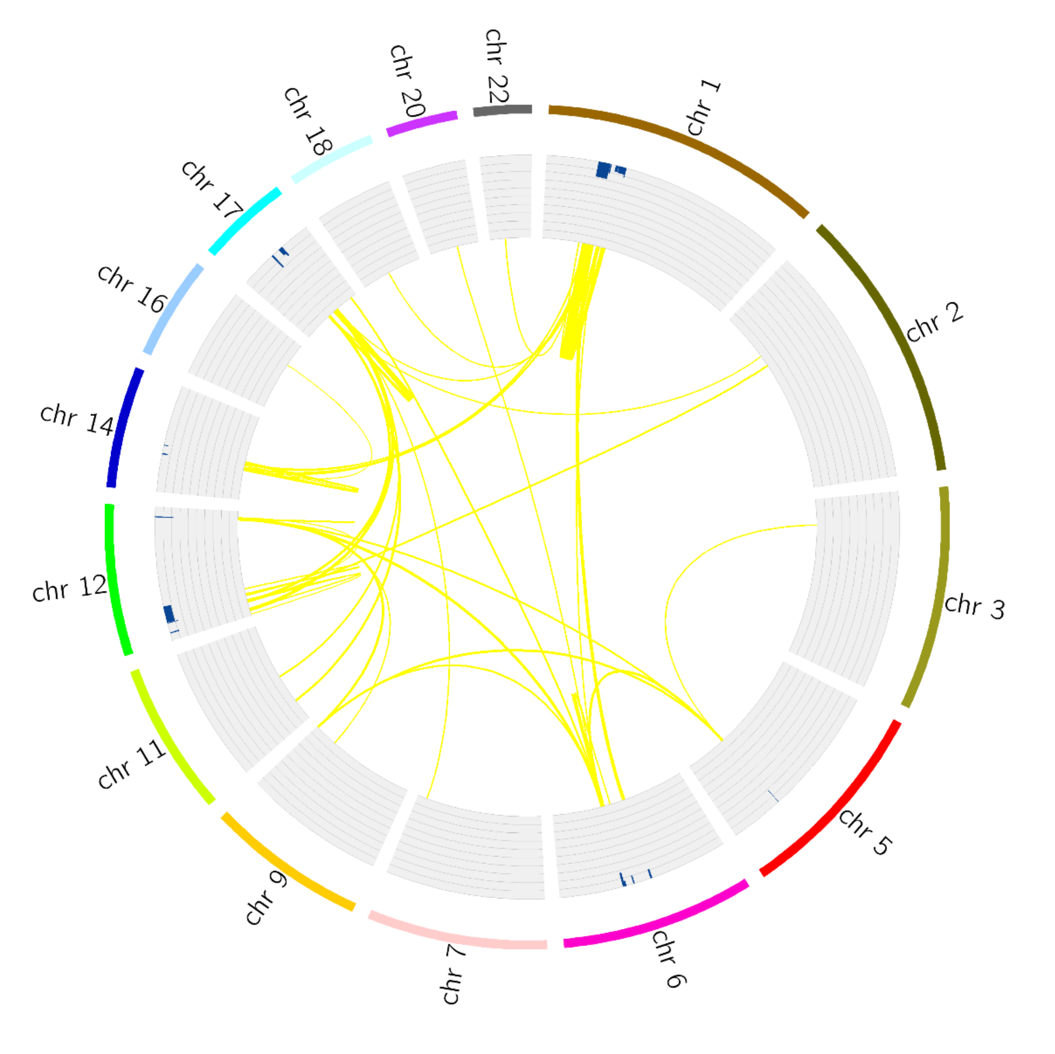

FAST – ERBB2 amplicon

BT474

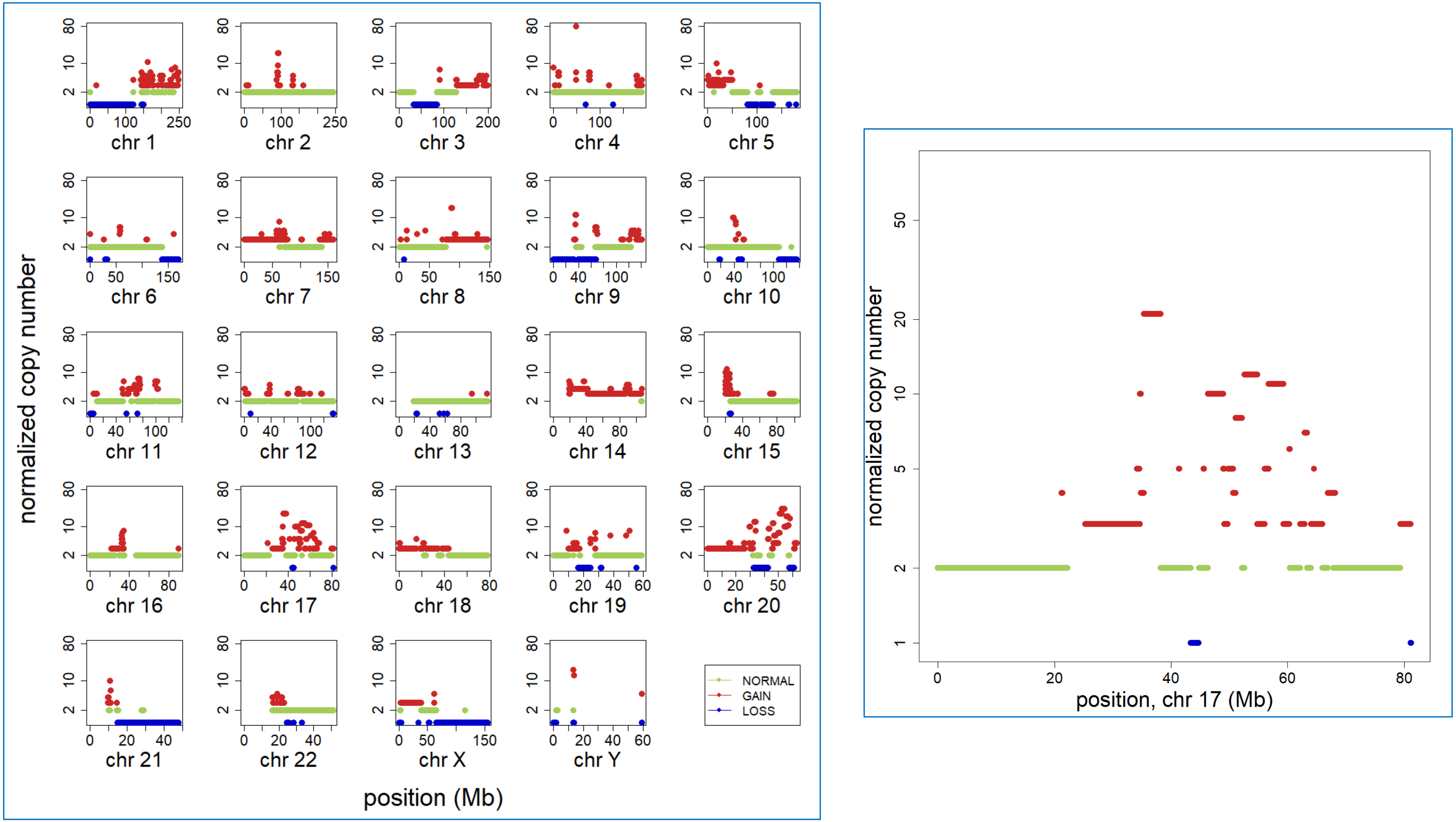

Control-FREEC

BT474

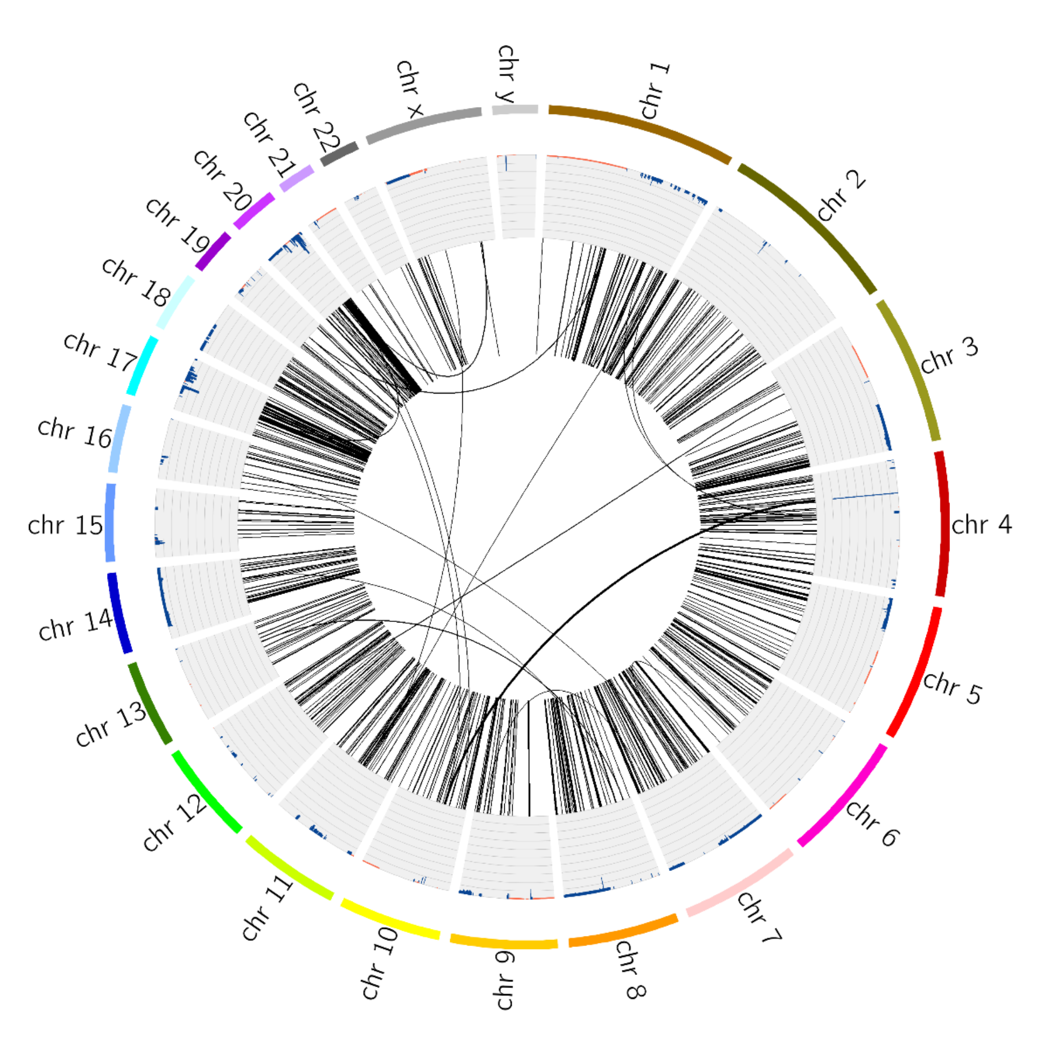

BreakDancer + Control-FREEC

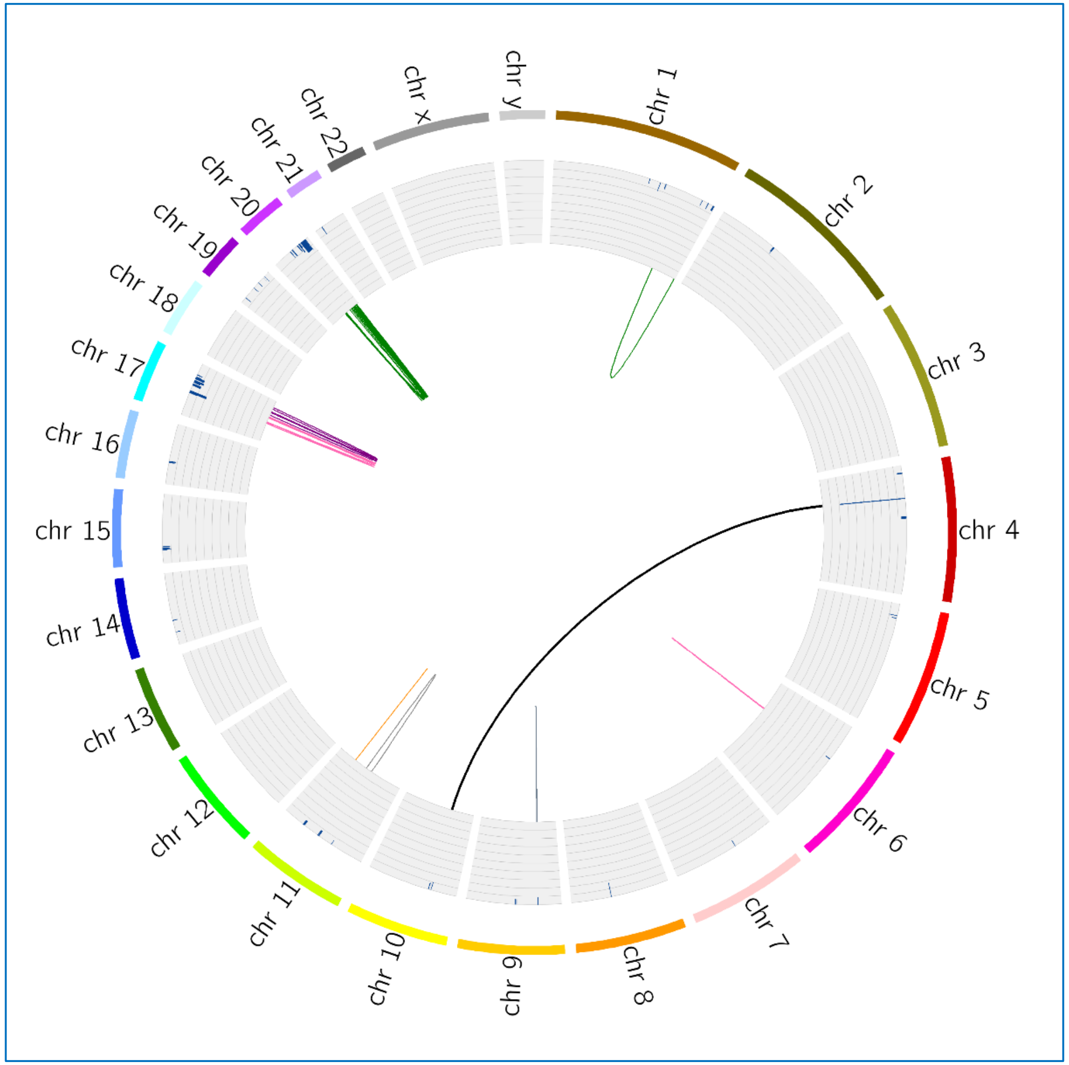

FAST – Whole Genome

BT474

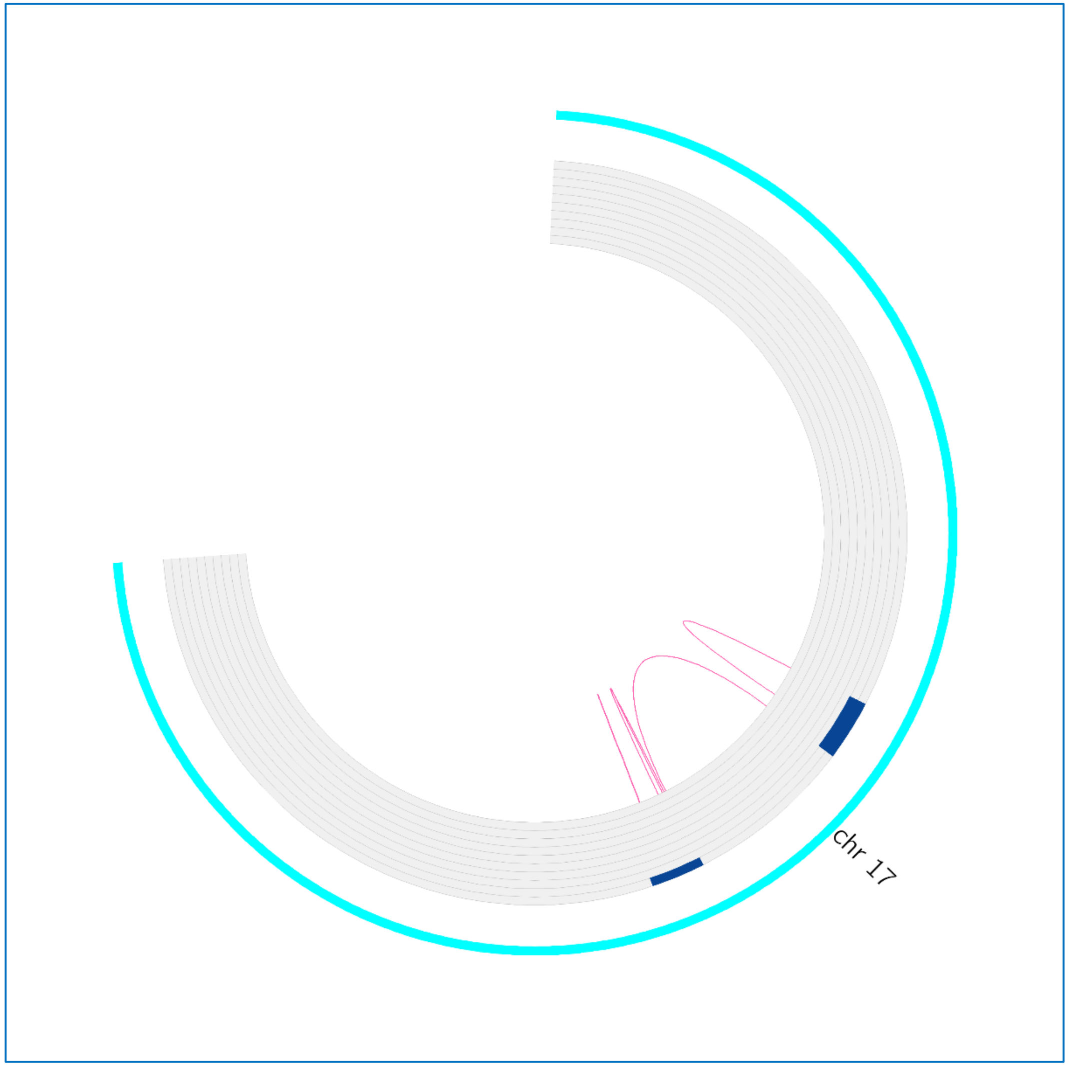

FAST – ERBB2 amplicon

HCC1954

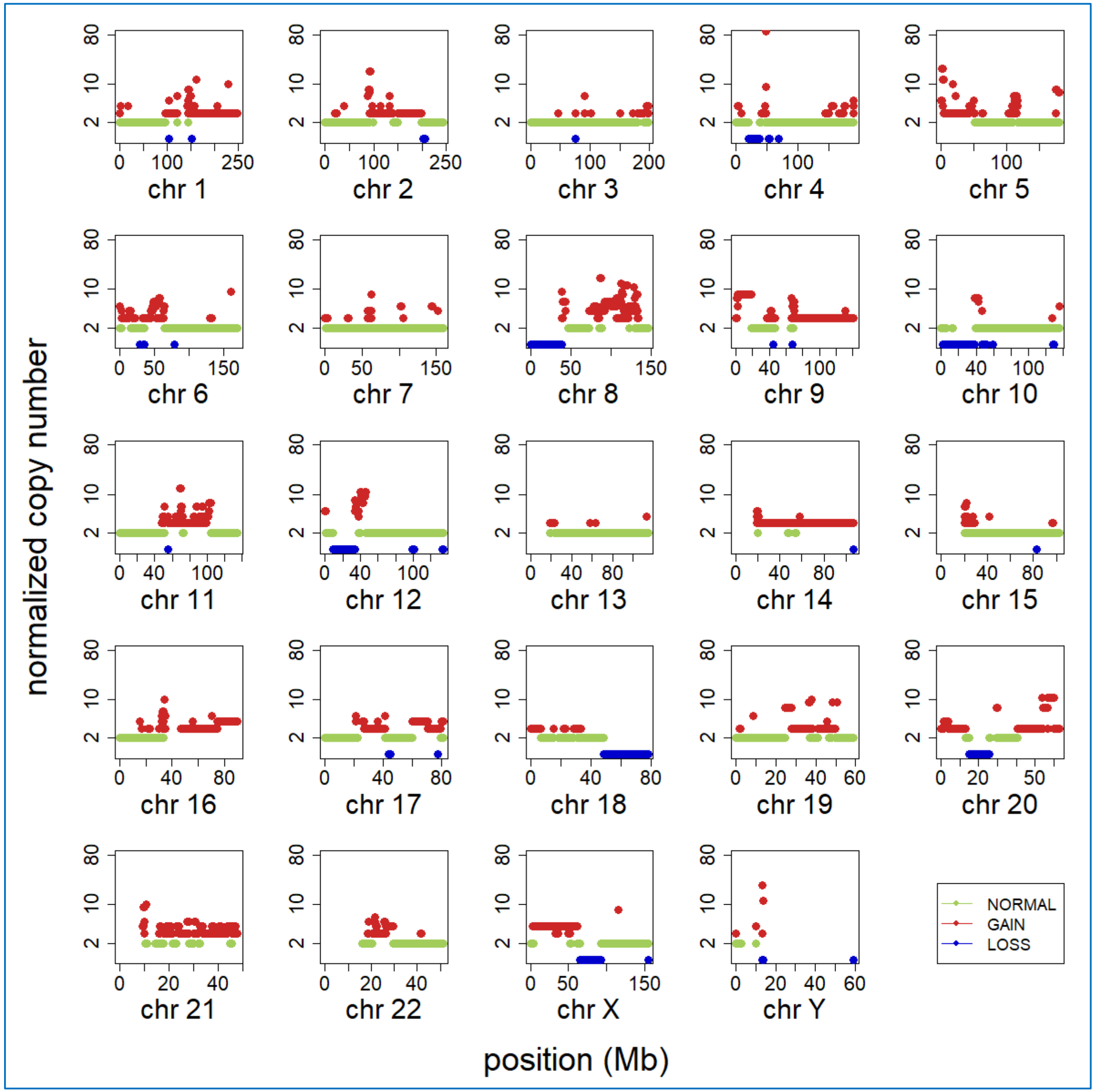

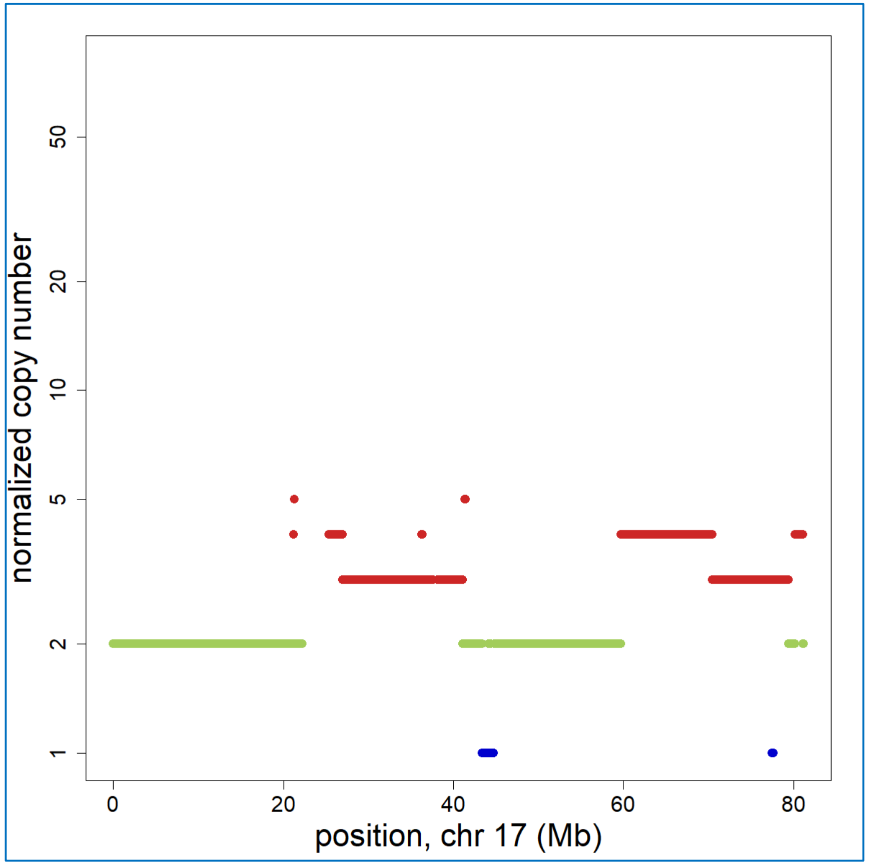

Control-FREEC

HCC1954

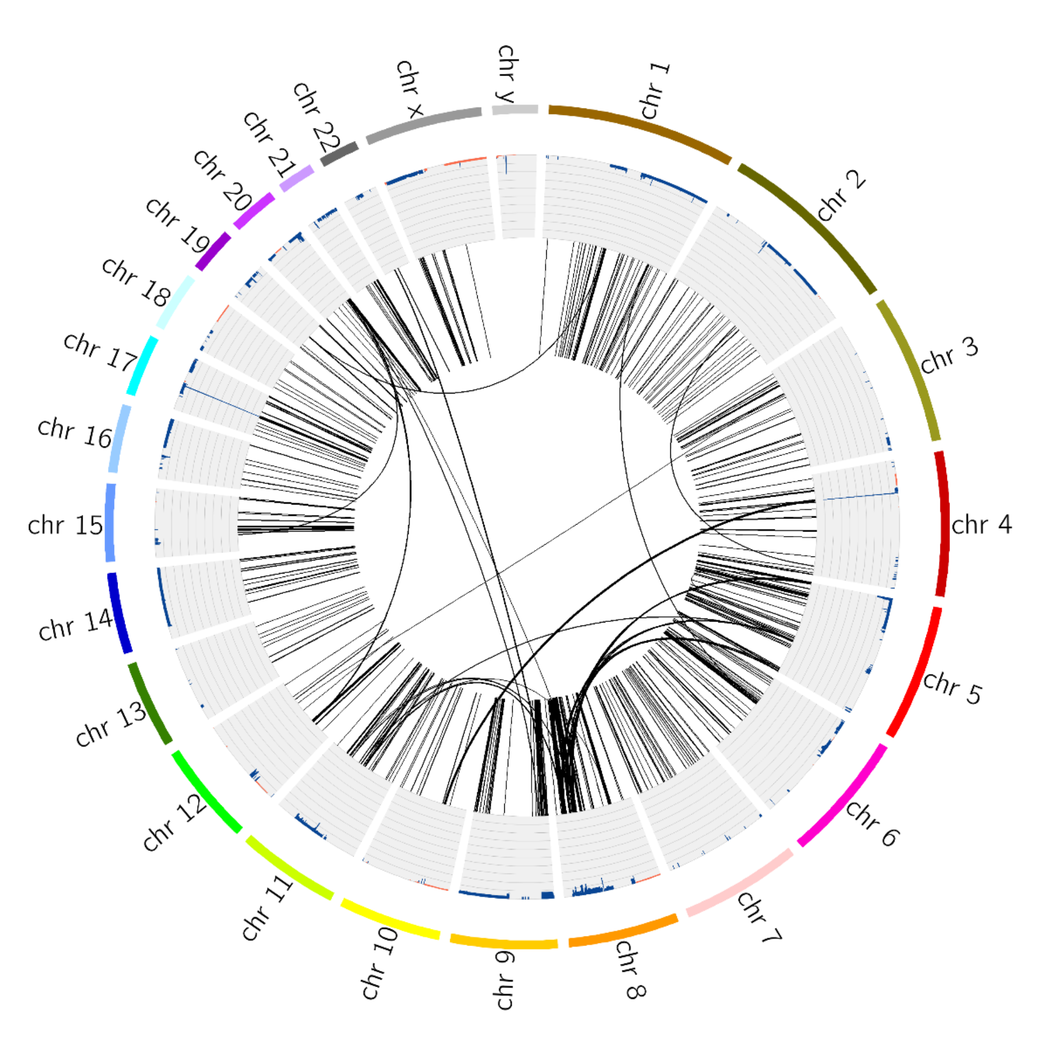

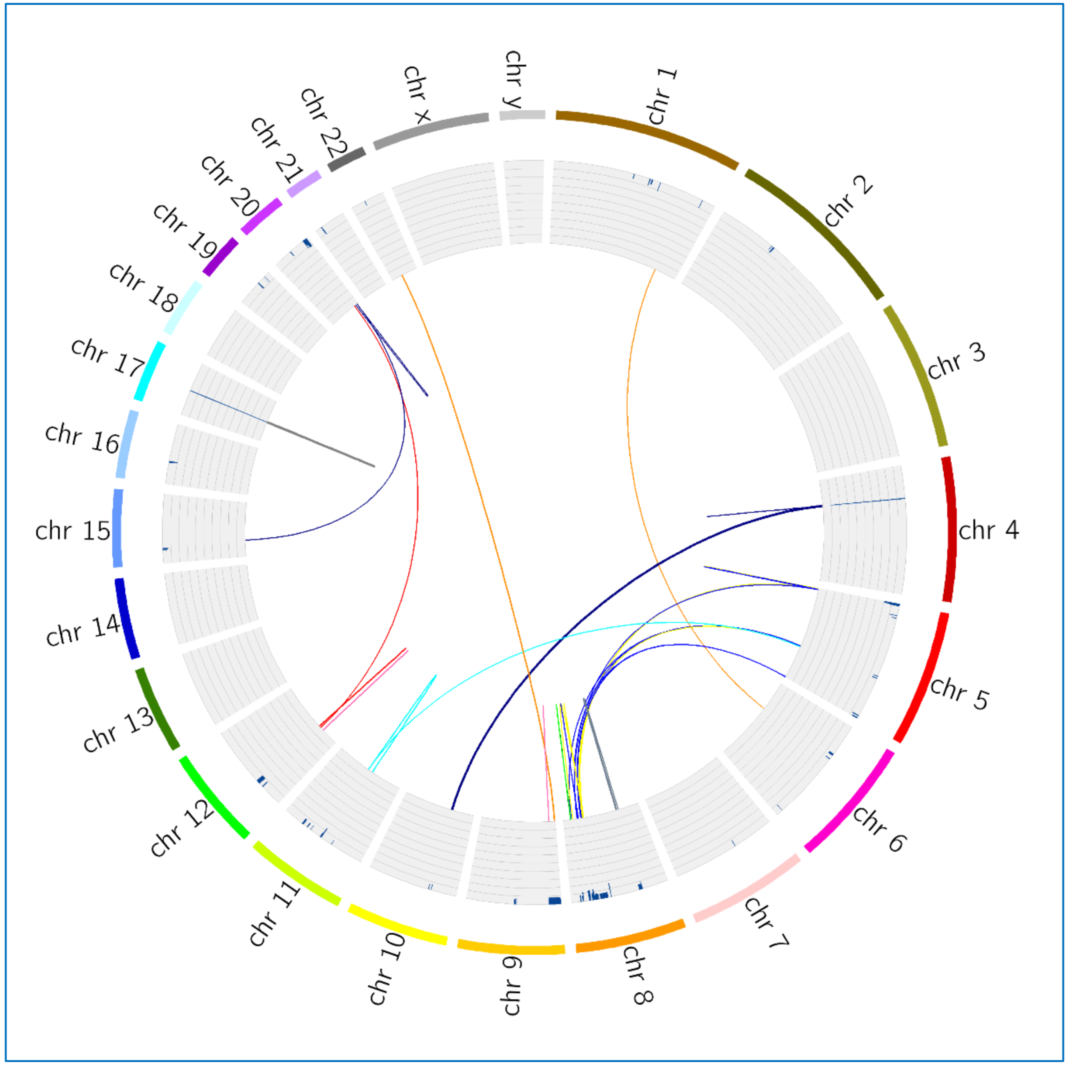

BreakDancer + Control-FREEC

FAST – Whole Genome

HCC1954

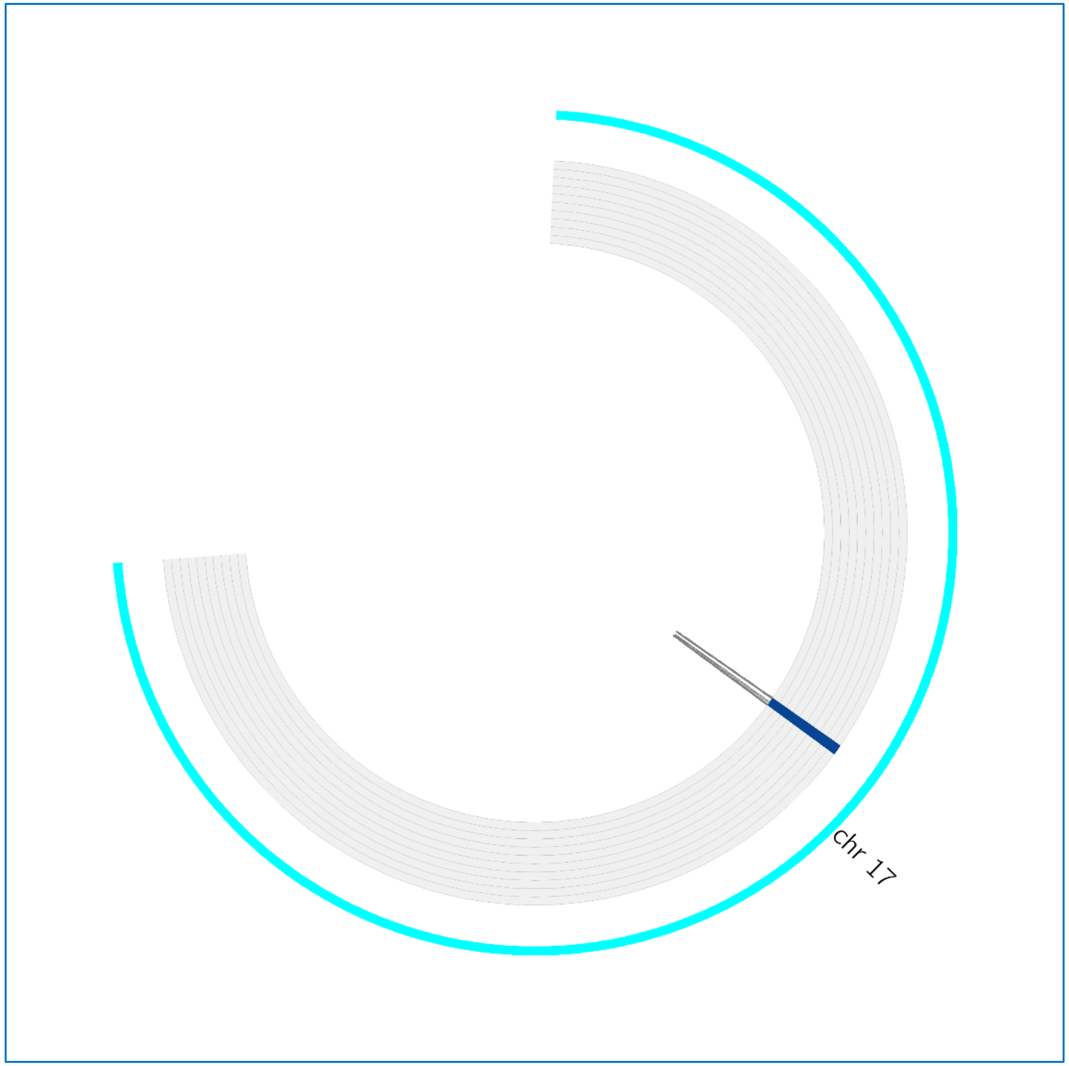

FAST – ERBB2 amplicon

MCF7

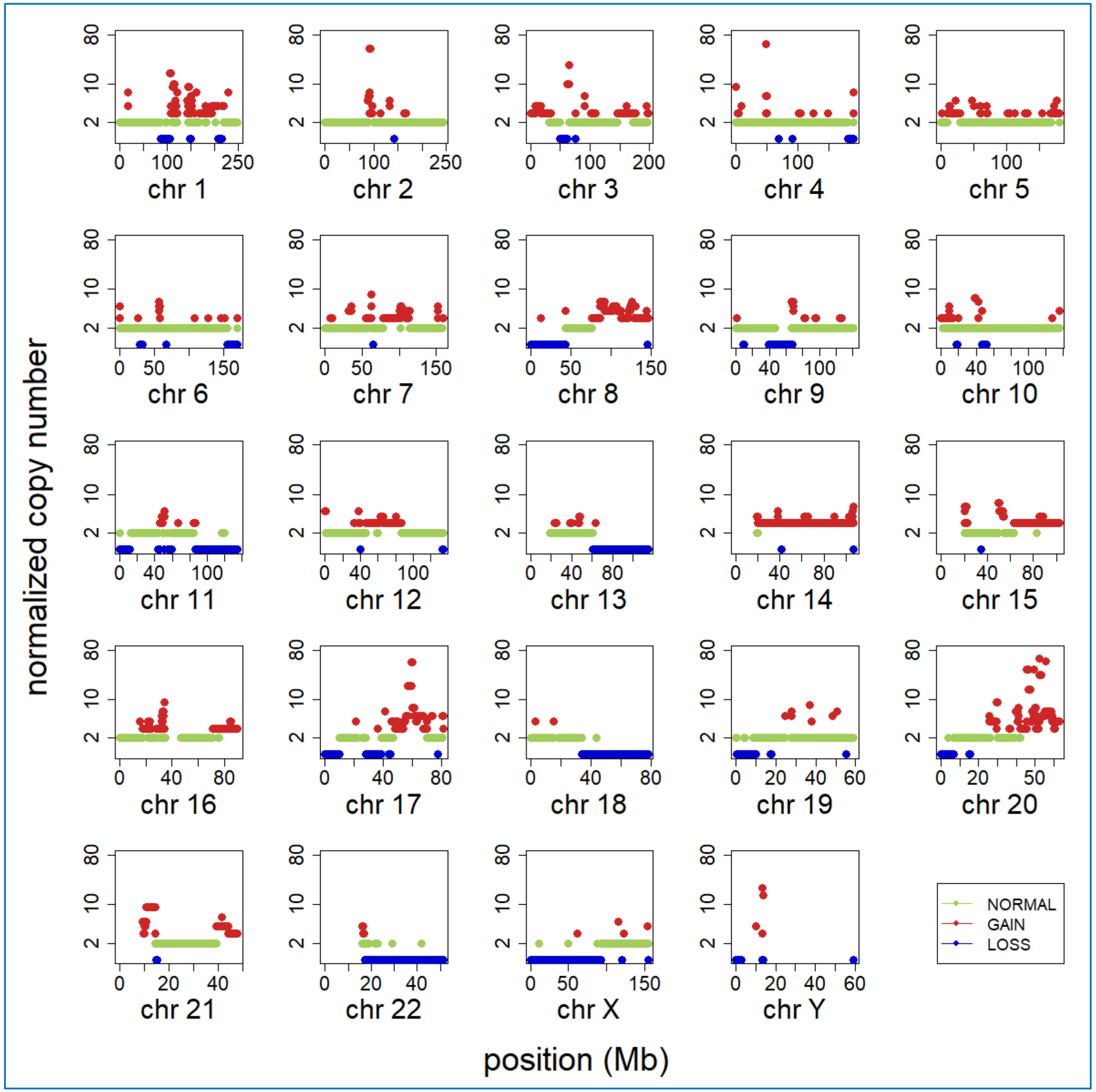

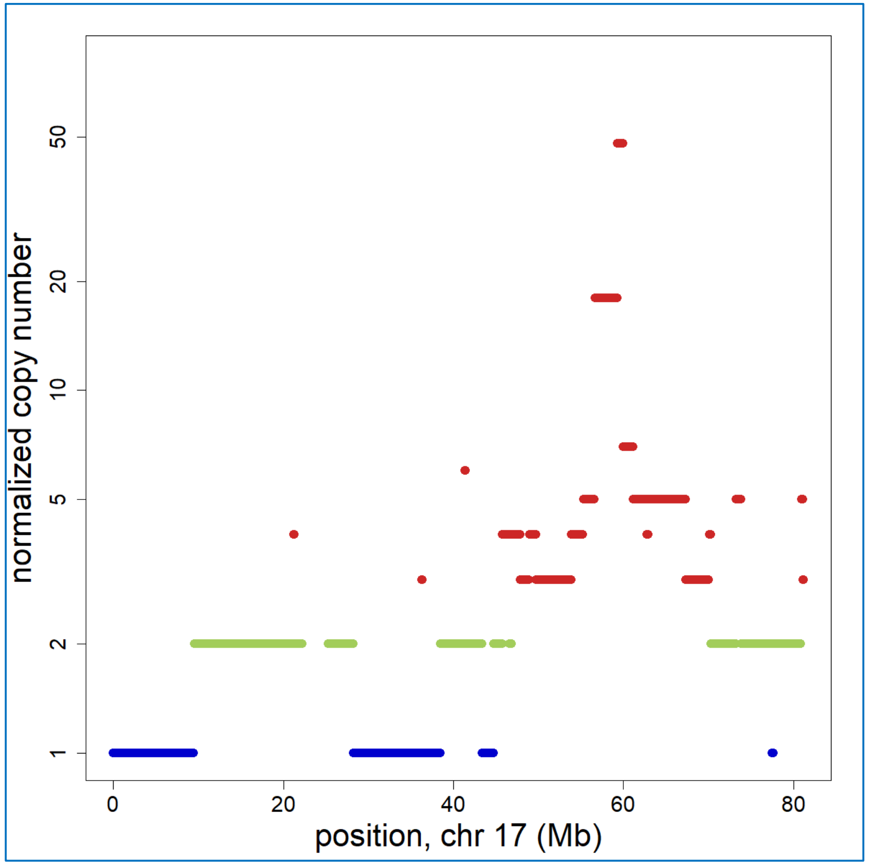

Control-FREEC

MCF7

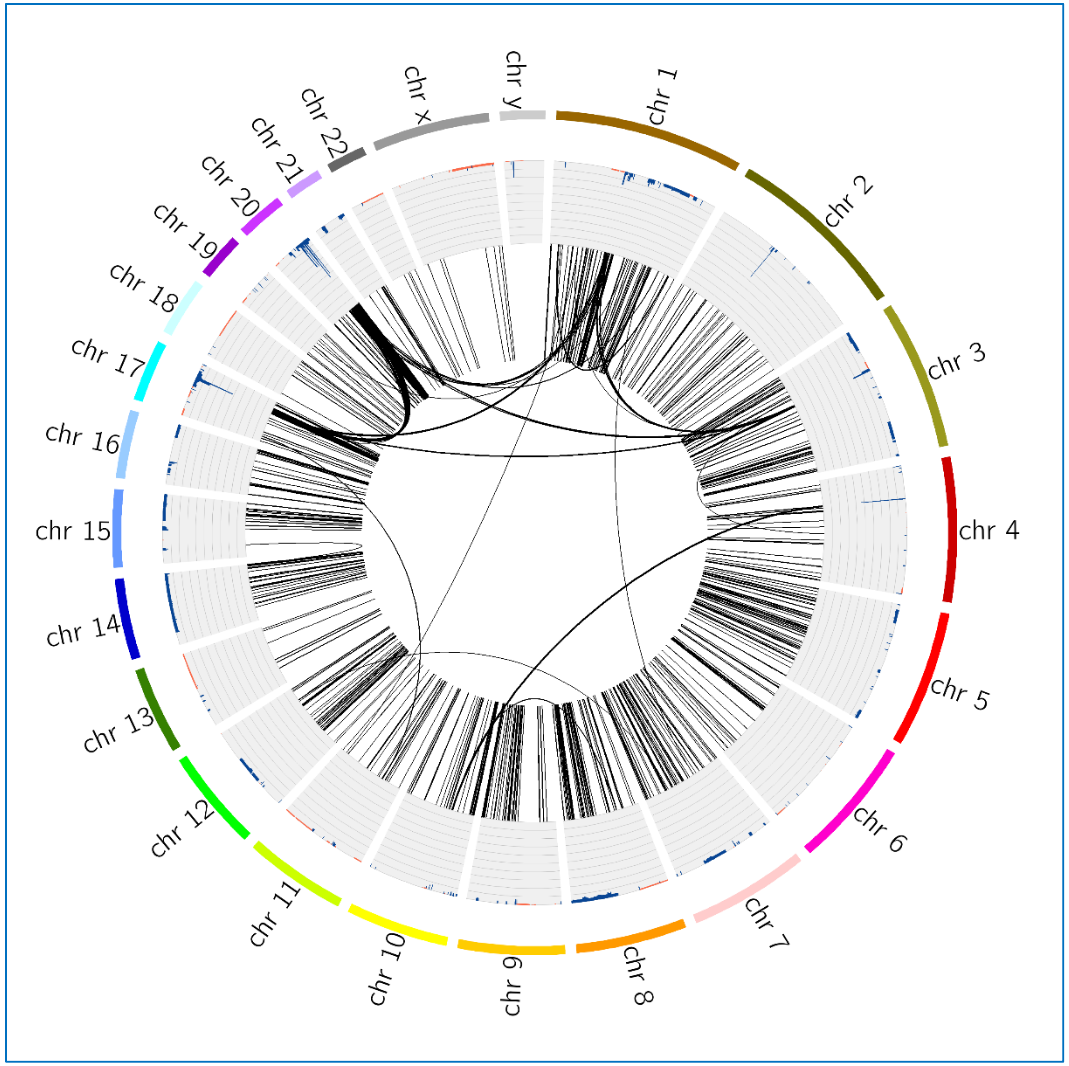

BreakDancer + Control-FREEC

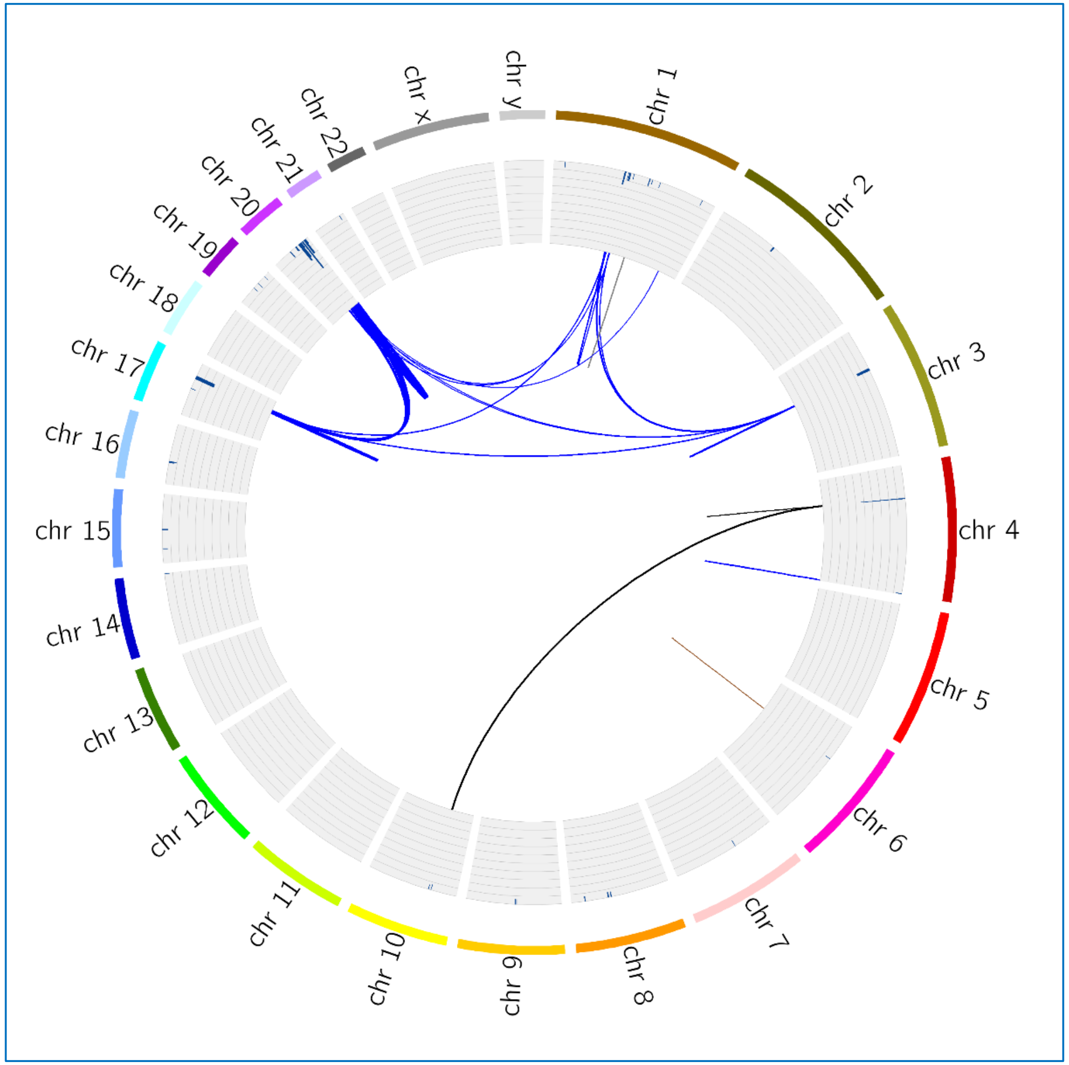

FAST – Whole Genome

MCF7

No FAST data for this sample

FAST – ERBB2 amplicon

MDA361

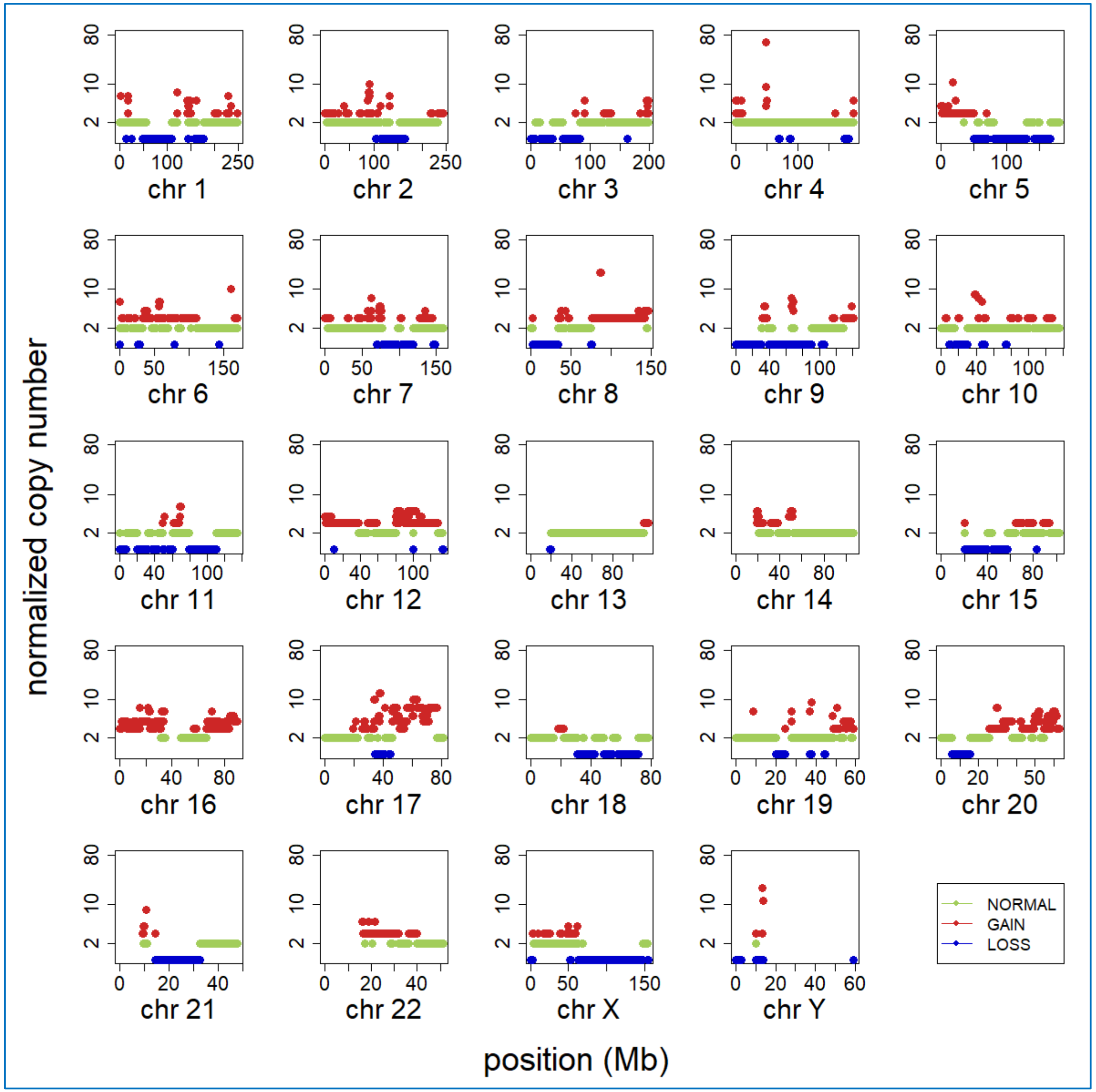

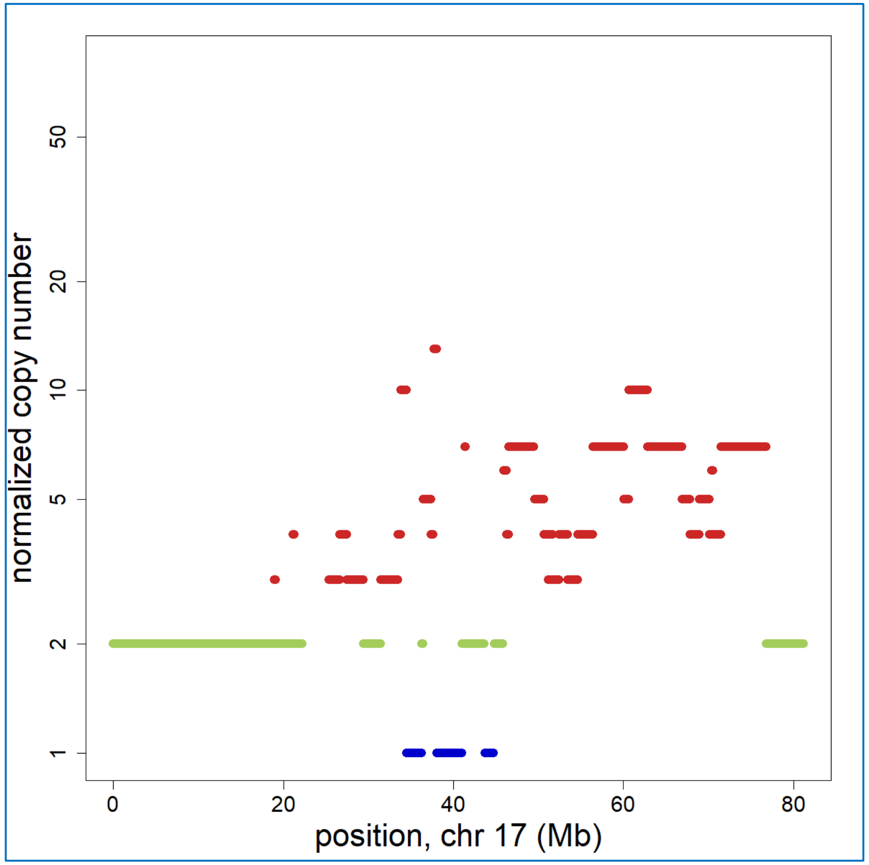

Control-FREEC

MDA361

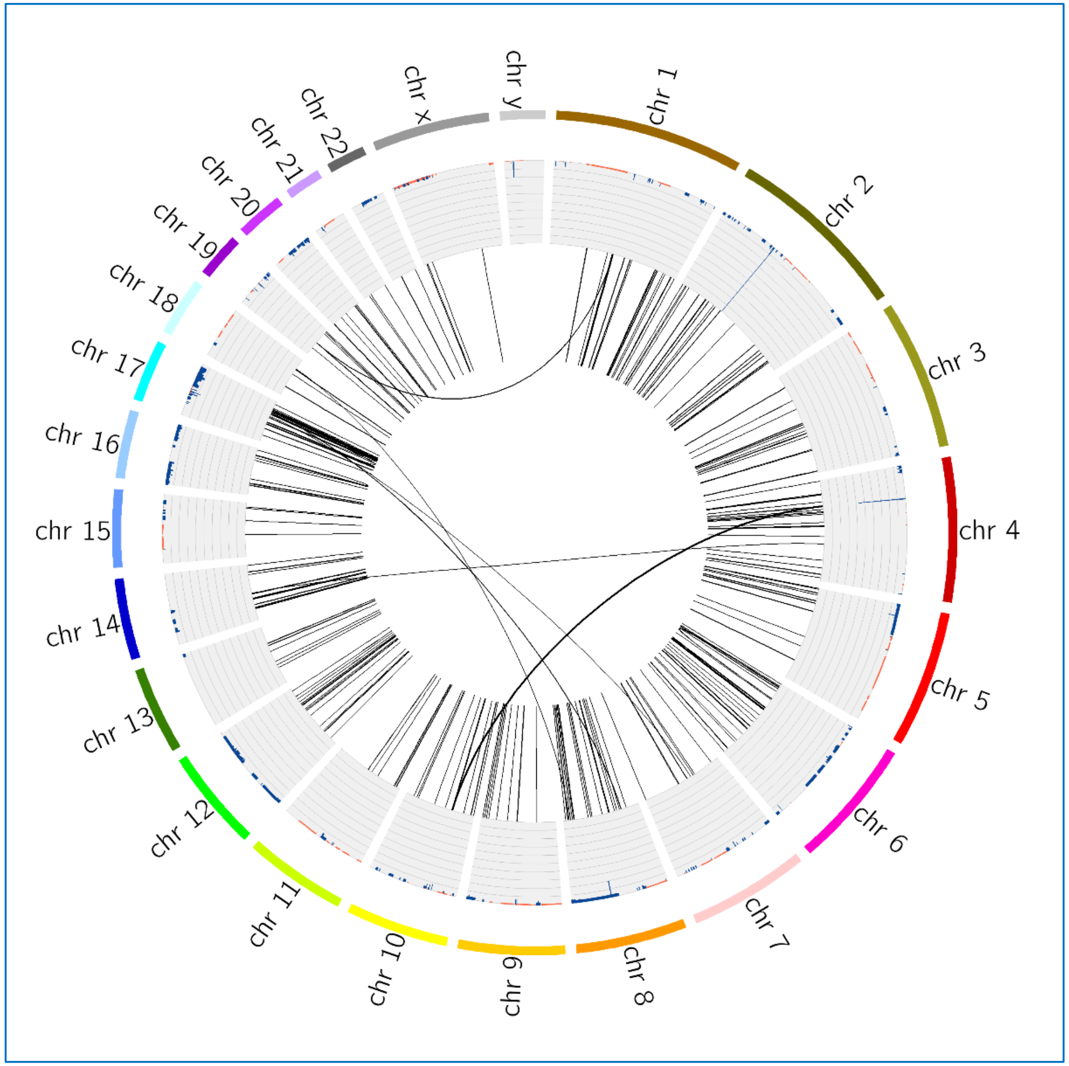

BreakDancer + Control-FREEC

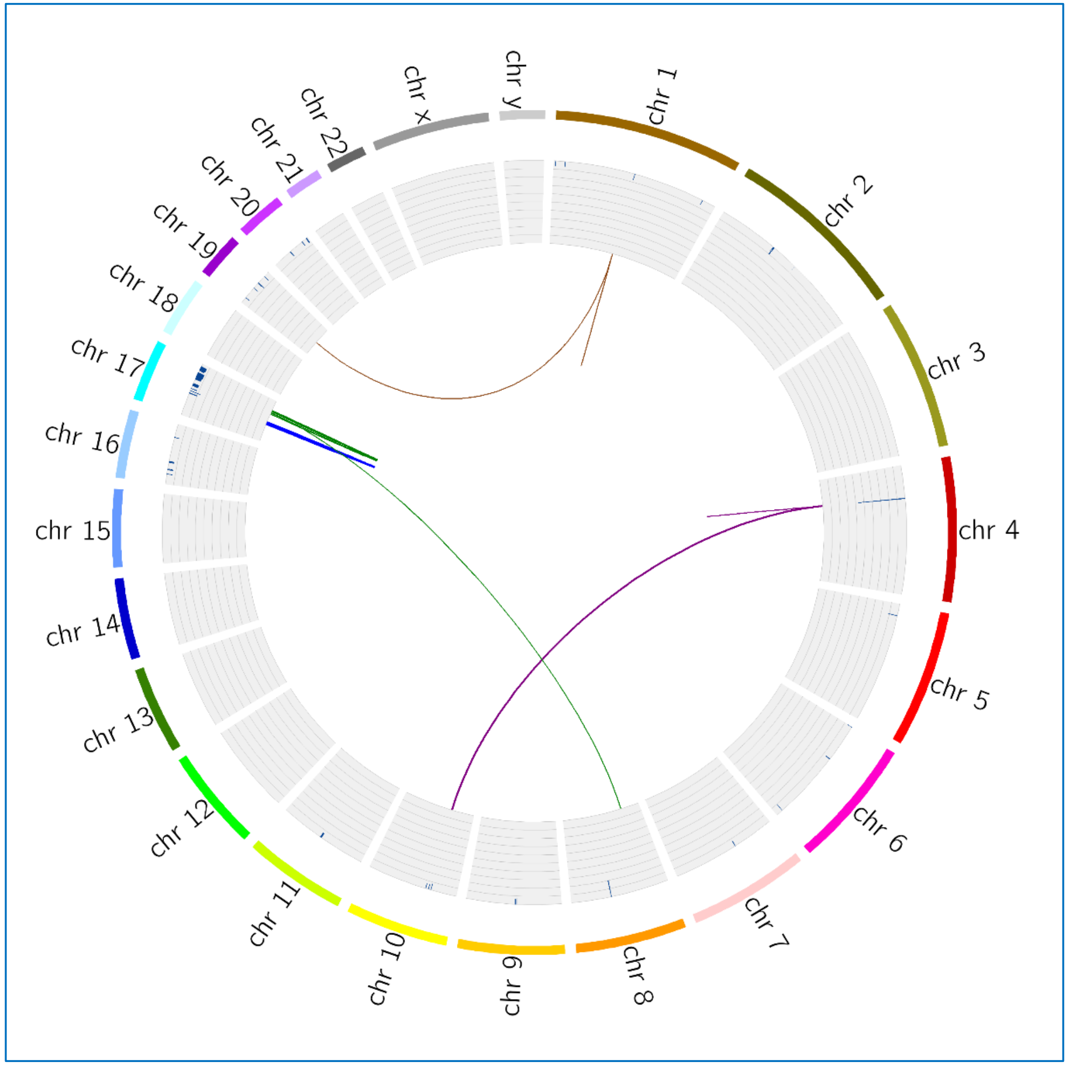

FAST – Whole Genome

MDA361

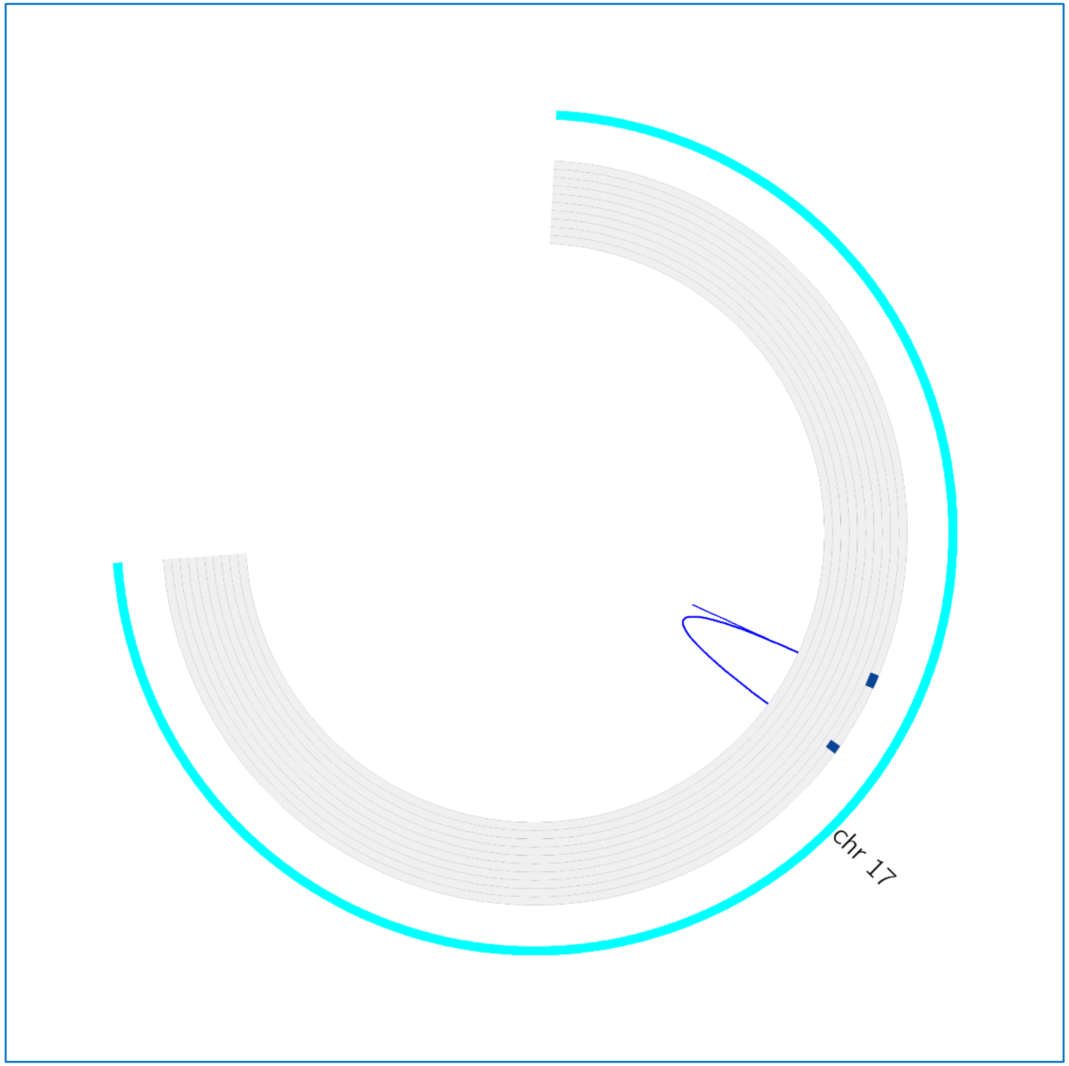

FAST – ERBB2 amplicon

SKBR3

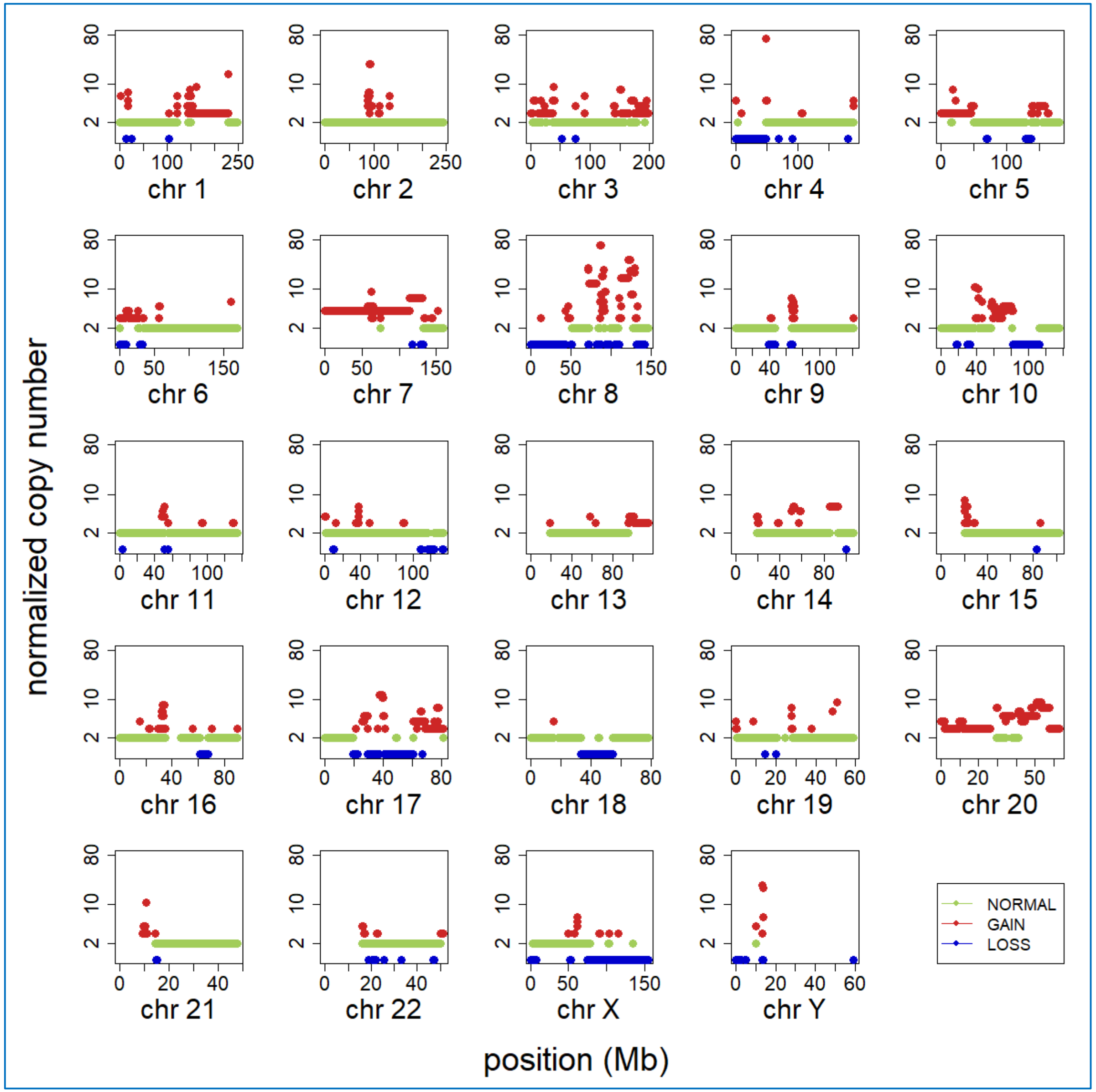

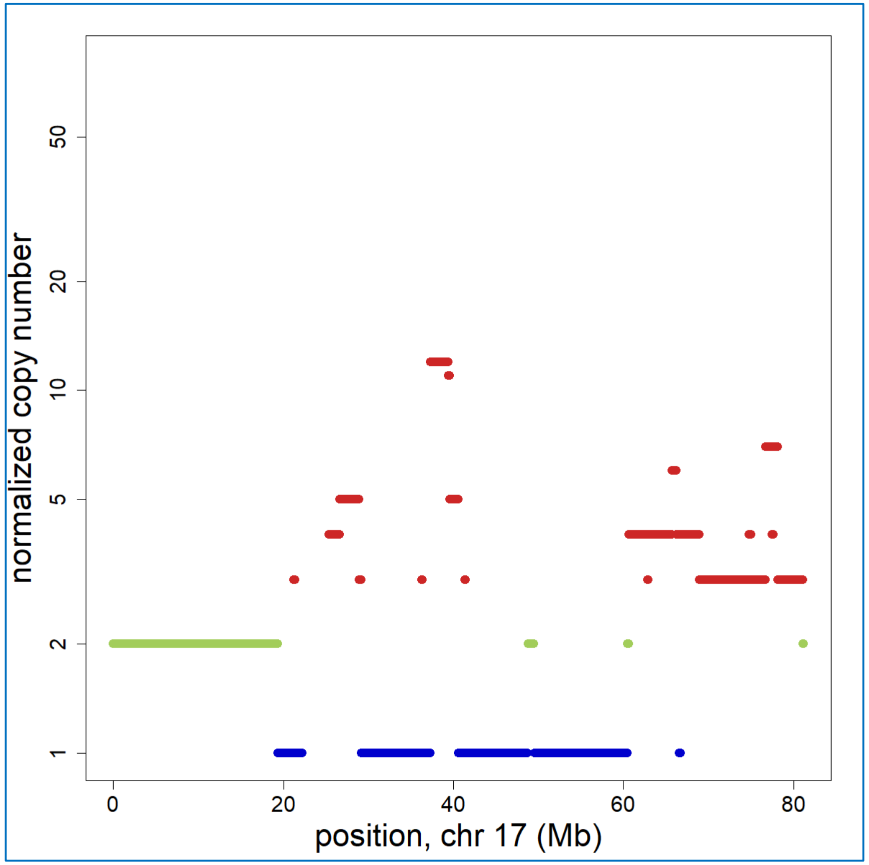

Control-FREEC

SKBR3

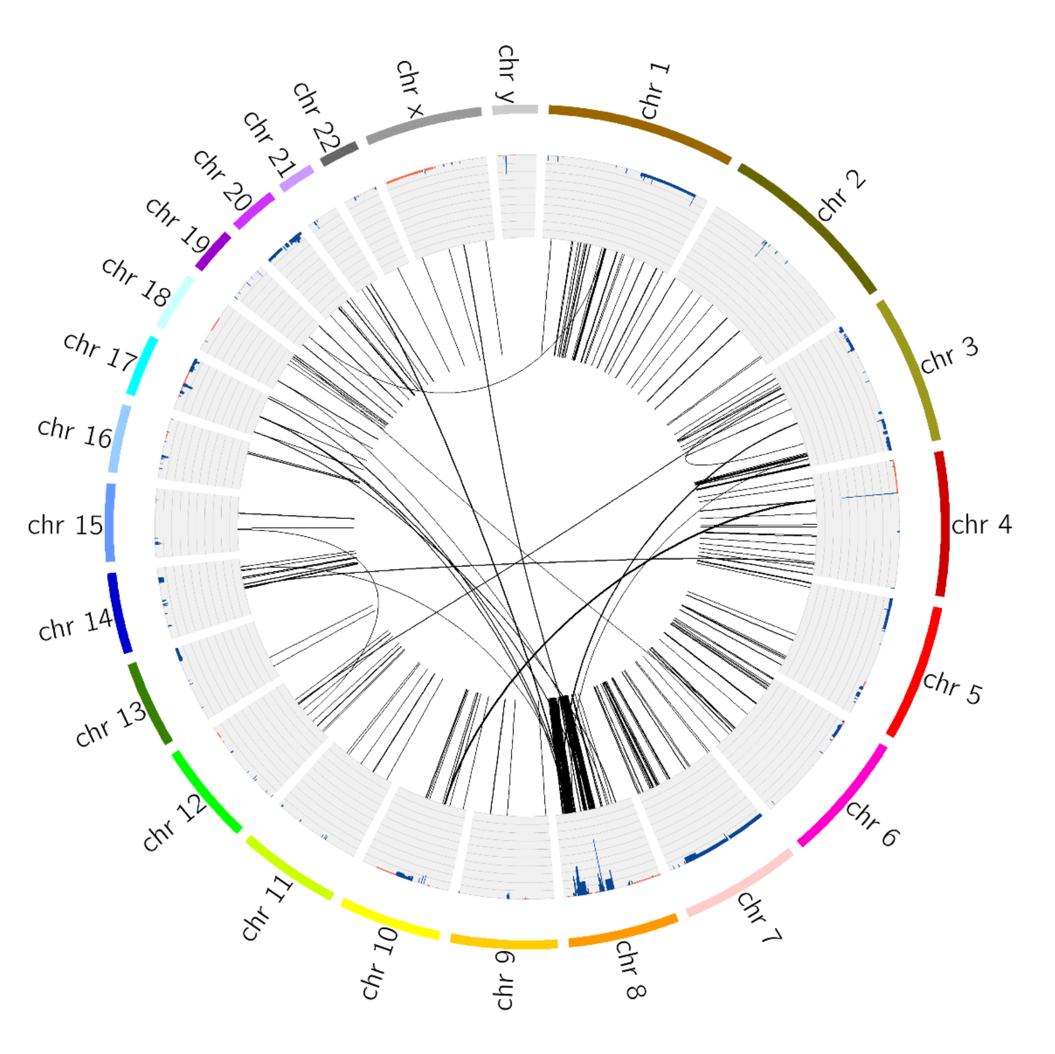

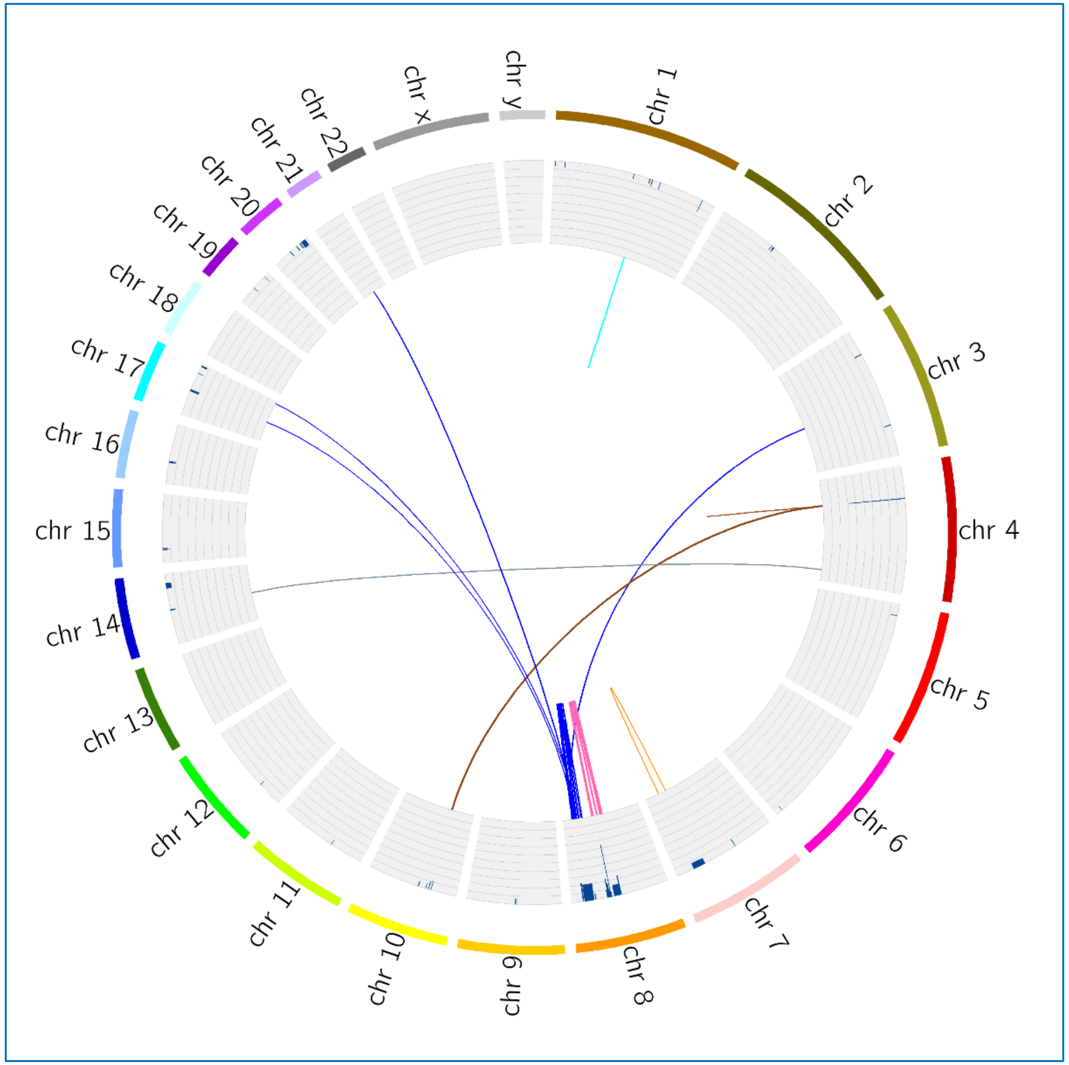

BreakDancer + Control-FREEC

FAST – Whole Genome

SKBR3

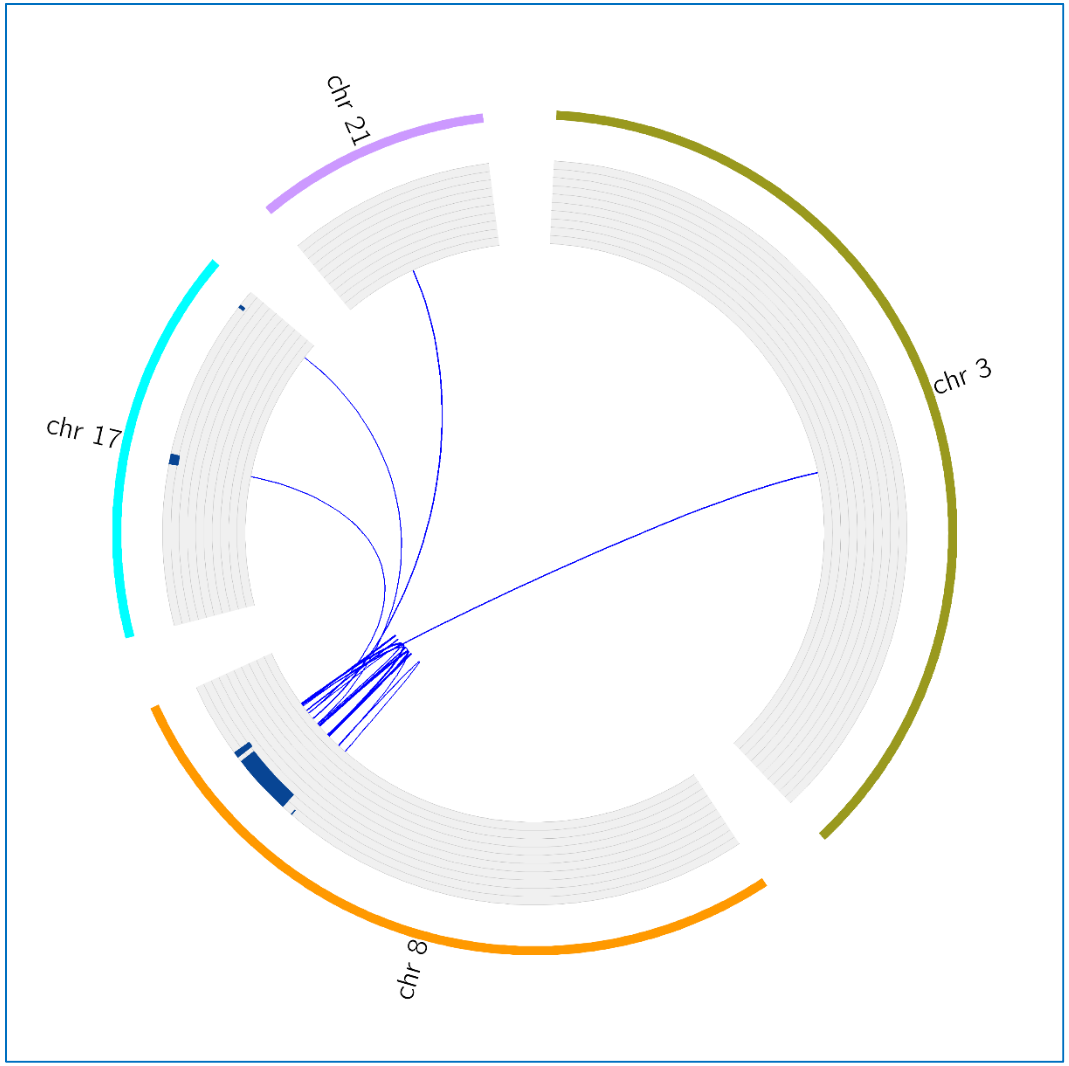

FAST – ERBB2 amplicon

ZR-75-30

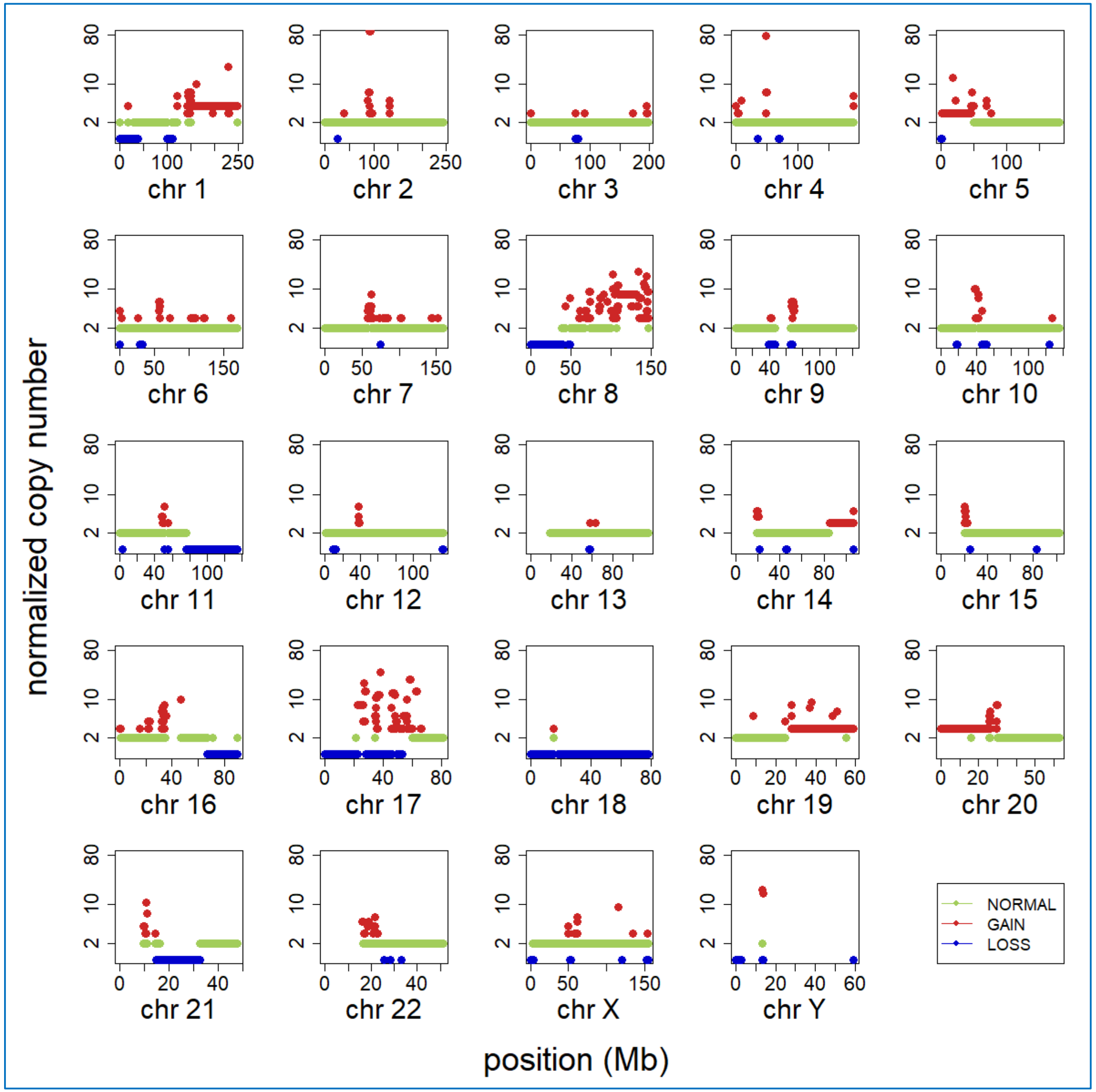

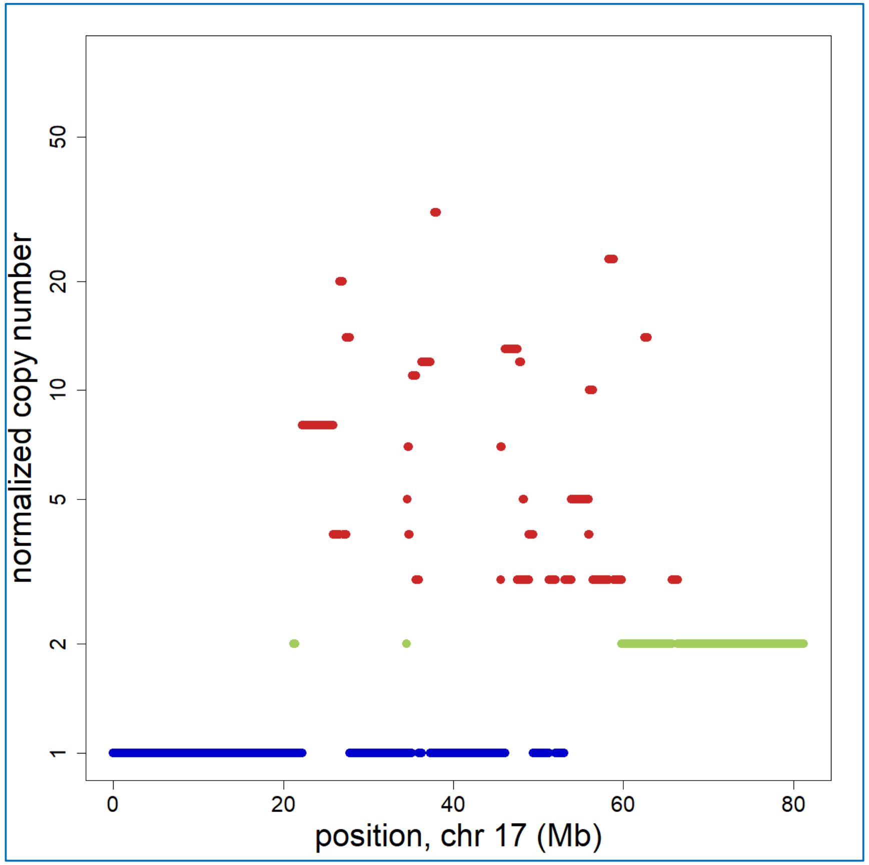

Control-FREEC

ZR-75-30

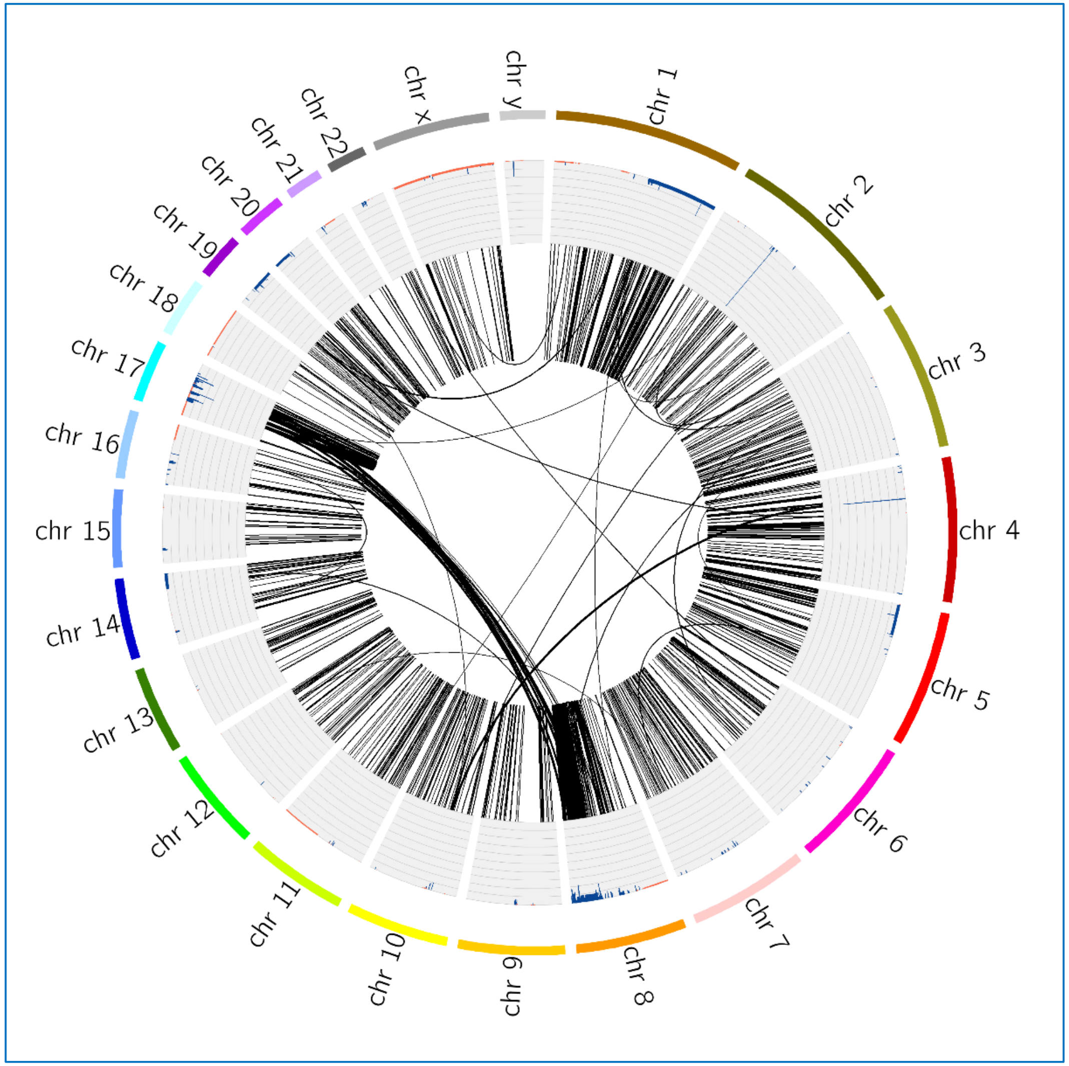

BreakDancer + Control-FREEC

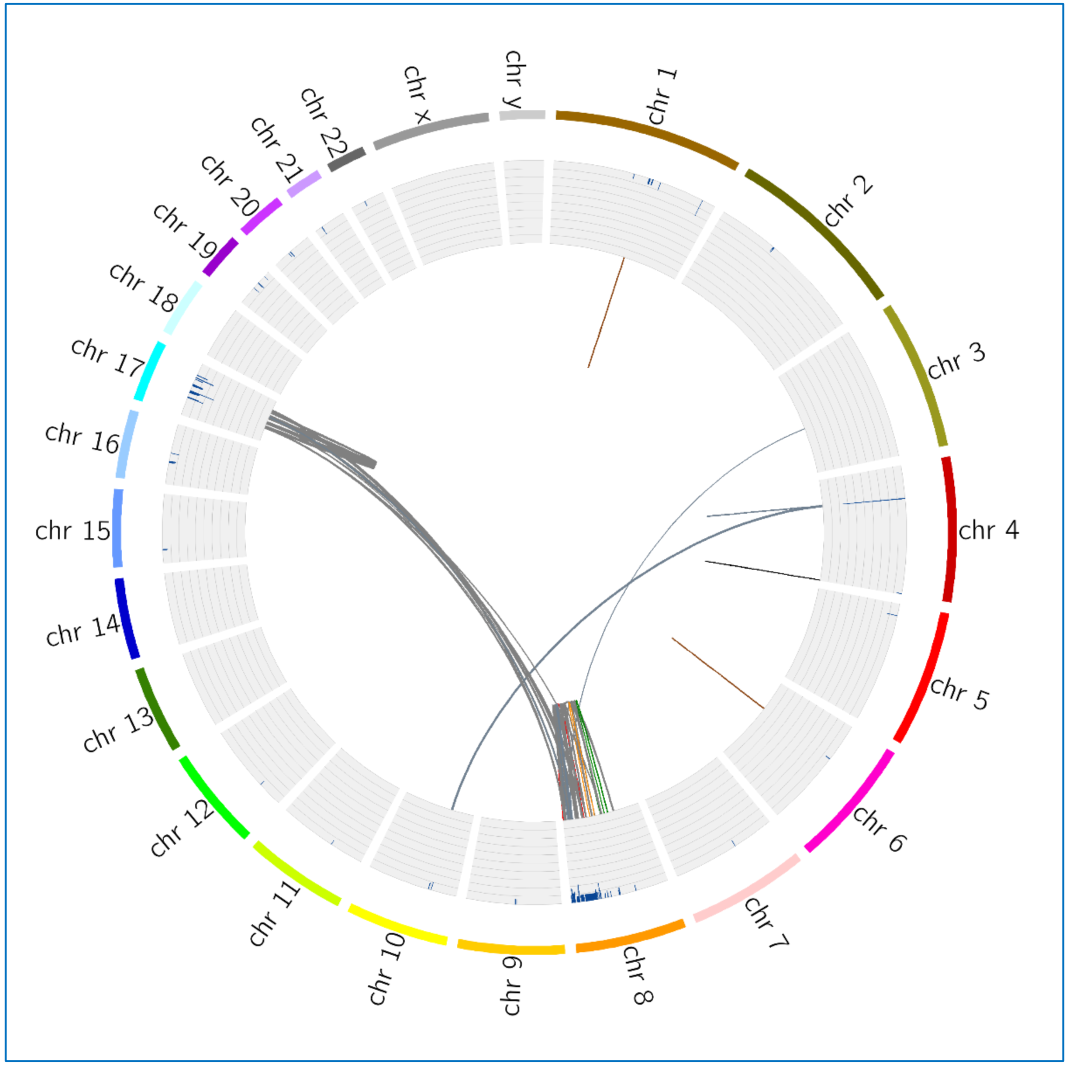

FAST – Whole Genome

ZR-75-30

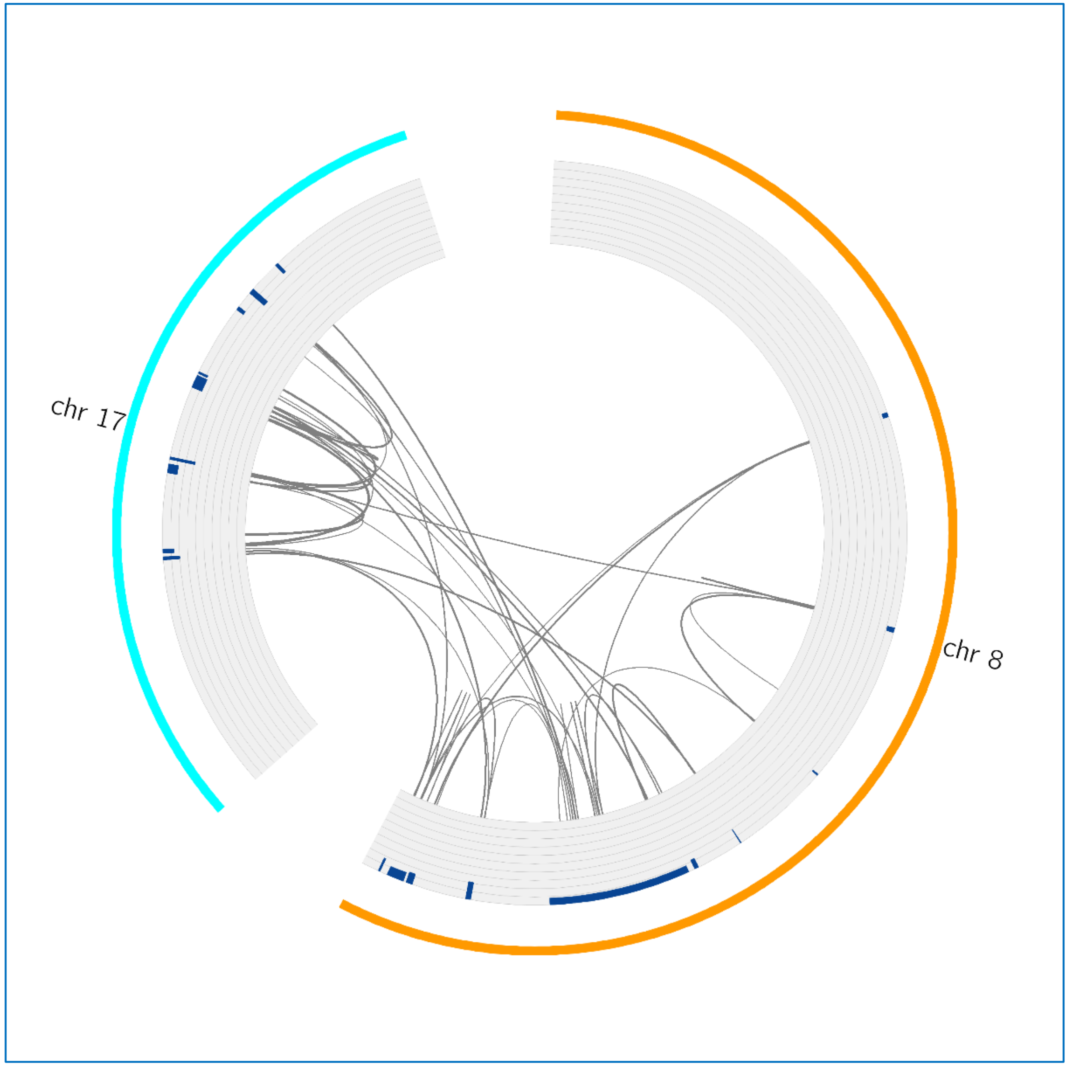

FAST – ERBB2 amplicon
